# Integrin αVβ5 regulates myoblast proliferation and differentiation in sarcopenia mice treated with FNDC5 gene delivery: Original article

**DOI:** 10.1186/s13395-026-00420-x

**Published:** 2026-03-17

**Authors:** Guoxi Gao, Qiyang Wang, Yuqiong Zhang, Minghong Shao, Yiming Liang, Shumin Zhou, Sheng Lu

**Affiliations:** 1 Graduate School, Kunming Medical University, No.1168, Chunrong West Road, Yuhua Street, Chenggong District, Kunming 650500 Yunnan, China; 2 Department of Orthopaedics, 920th Hospital of the Joint Logistics Support Force of the Chinese People’s Liberation Army, Kunming 650032 Yunnan, China; 3 Department of Orthopedic Surgery, the First People’s Hospital of Yunnan Province, the Affiliated Hospital of Kunming University of Science and Technology, Kunming 650032 Yunnan, China; 4 The Institute of Microsurgery on Extremities, Shanghai Sixth People Hospital, 200233 Shanghai, China; 5 Department of Orthopedics, Shanghai Sixth People Hospital, 200233 Shanghai, China

**Keywords:** FNDC5, Sarcopenia, Integrin αVβ5, Proliferation, Myogenic differentiation

## Abstract

**Background:**

Irisin which is encoded by *FNDC5* gene, has emerged as a promising therapeutic candidate for alleviating sarcopenia; however, its long-term therapeutic application in muscle regeneration remains insufficiently developed.

**Methods:**

Our study investigated the potential of *FNDC5* gene delivery in both cellular and animal levels via a virus vector. It was indicated that *FNDC5* overexpression promoted myoblast proliferation while inhibiting myogenic differentiation, and vice versa. Mechanistically, we confirmed that the product of *FNDC5* gene, irisin binds to integrin αVβ5 on the cell membrane of myoblasts, leading to activation of the downstream FAK/SRC complex using coimmunoprecipitation (co-IP) and confocal microscopy. RNA-Seq and qPCR analyses revealed that irisin exposure activated genes related to the extracellular matrix (ECM) and its ligands, thereby promoting the cell cycle and enhancing the proliferation of myoblast. Additionally, treatment with the integrin αVβ5 inhibitor cilengitide enhanced myogenic differentiation but suppressed cell proliferation, highlighting the crucial role of integrin αVβ5 in determining myoblasts’ cell fate. Furthermore, in sarcopenia mouse models, AAV-mediated *FNDC5* supplementation activated integrin αVβ5-related signaling as expected, resulting in increased muscle mass and alleviation of sarcopenia symptoms.

**Results:**

Our findings demonstrate that integrin αVβ5 acts as a key switch in regulating the proliferation and differentiation of myoblasts under *FNDC5* gene delivery. When integrin αVβ5 is active, *FNDC5* over-expression enhances cell proliferation rather than differentiation. Conversely, when integrin αVβ5 is inhibited, *FNDC5* over-expression promotes myogenic differentiation over proliferation. Moreover, the binding of irisin to integrin αVβ5 induces the expression of ECM proteins in myoblasts and activates the Bgn-TLR4 signaling pathway, which further enhances cell proliferation. This FNDC5-ITGAVB5-FAK-mTOR-Bgn-TLR4 signaling axis was also validated in a sarcopenia mouse model.

**Conclusion:**

Integrin αVβ5 is a critical regulator of myoblast proliferation and differentiation in response to *FNDC5* gene supplement. These findings provide new insights into *FNDC5* biological functions and suggest potential therapeutic strategies for enhancing muscle regeneration and treating sarcopenia.

**Supplementary Information:**

The online version contains supplementary material available at 10.1186/s13395-026-00420-x.

## Introduction

Aging-related diseases, particularly those involving tissue degeneration such as muscle wasting, are becoming major public health concerns [1]. Skeletal muscles, which are vital for movement, posture, and metabolic regulation. With age, imbalances in muscle protein turnover contribute to muscle loss, which is further exacerbated by reduced physical activity, malnutrition, chronic diseases, systemic inflammation, and hormonal decline [2]. Key anabolic hormones-including IGF-1, DHEA, testosterone, and estrogen-decrease with age, accelerating muscle deterioration and weakness [1, 3, 4]. While exercise and nutritional support remain central interventions, hormonal therapies are limited by their complexity and systemic effects, highlighting the need for new therapeutic approaches [5].

Sarcopenia, one of the main forms of muscle wasting characterized by the progressive loss of muscle mass and function, primarily affects aging populations or individuals with underlying diseases [3]. Additionally, older adults commonly experience metabolic dysfunctions, including insulin resistance, nonalcoholic fatty liver disease, and hepatitis [6]. Hormone replacement therapies, particularly estrogen replacement, have shown potential in preserving muscle mass and strength in postmenopausal women [7]. However, the complexities of hormone distribution and mechanisms limit widespread use, necessitating further exploration of treatment options for muscle wasting.

*FNDC5* gene, initially identified by Bostrom, has garnered attention for its multifaceted role in muscle biology [8]. In addition to metabolism, *FNDC5* plays a critical role in muscle proliferation, repair, and metabolic modulation [9]. Encoded by the *FNDC5* gene, irisin is synthesized primarily in muscle tissue in response to physical exercise. It interacts with the integrin αVβ5 in bone cells to inhibit bone loss [10] and activates the AMPK-UCP5 pathway through αVβ2 integrin receptors. Notably, the protective effect of irisin on intestinal barrier integrity depends on integrin αVβ5, AMPK, or UCP2 inhibition [11], highlighting its integrin-mediated actions in the musculoskeletal system. However, the interaction of irisin with integrin αVβ5 receptors in muscle cells and its downstream implications remains poorly understood.

In this study, we examined the effects of supplying *FNDC5* on both the proliferation and differentiation of myoblasts in a sarcopenia model. Our findings revealed that *FNDC5* delivery to myoblasts promotes cell proliferation when integrin αVβ5 pathway is activated but promotes differentiation when integrin αVβ5 pathway is inhibited. These results contribute to our understanding of *FNDC5* supplementation in treating sarcopenia by elucidating how it determines the cell fate of myoblasts and may offer insights into enhancing the management of sarcopenia, suggesting a potential therapeutic avenue for individuals affected by sarcopenia.

## Materials and methods

### Stable cell construction

C2C12 cells were transduced with lentiviruses carrying FNDC5 overexpression (OE-FNDC5) or knockdown (sh-FNDC5) constructs (Tsingke Biotechnology, Nanjing, China). For transduction, cells were seeded at 5 × 10^4^ cells/well in 24-well plates and, on the next day, were incubated with 150 µL lentiviral particles at MOI = 100. At 72 h post-transduction, cells were selected with puromycin (4µg /mL) for 144 h and then maintained in puromycin-free medium. Details regarding the vectors and sequences of full-length *FNDC5* and the shRNA against *FNDC5* are provided in Supplementary Table 1.

### Cell culture, the cell cycle and myogenic differentiation

Murine C2C12 myoblasts were acquired from BNCC, Henan, China, and cultured in complete medium (DMEM + 10% FBS and 1% penicillin-streptomycin) at 37 °C. The cells were passaged when the confluence reached 80%.

For myogenic differentiation induction, C2C12 cells were differentiated by switching to differentiation medium (DMEM + 2% horse serum and 1% penicillin-streptomycin) when the confluence reached 90%. The medium was changed every two days until myotube formation was observed.

For cell-cycle analysis, OE-FNDC5 and OE-NC, as well as sh-FNDC5 and sh-NC cells, were serum-starved overnight to synchronize the cell cycle, then cultured in complete medium for an additional 24 h before harvest for flow cytometry.

### Cell proliferation detection

The proliferation of OE-FNDC5 and OE-NC, as well as sh-FNDC5 and sh-NC cells, was monitored using the RTCA DP System (Agilent) [12]. The cell was seeded at a density of 3030 cells/cm², and the cell proliferation curves were recorded continuously for 5 days with measurements taken every 2 h. Well impedance was automatically monitored by the xCELLigence system and reported as the Cell Index value (CI). The materials and methods used in this study are provided in detail in the Supplementary Materials.

### Giemsa staining

For morphological analysis of cell differentiation, C2C12 myotubes were stained with Giemsa solution (10% Giemsa stain solution, G1010, Solarbio) [13].

### Western blot analysis and qPCR analysis

Western blot analysis was performed according to previously described methods [14]. Details of the antibodies used are provided in Supplementary Table 2.

Total RNA isolation and qPCR experiments were conducted as previously described [15]. The primers used are listed in Supplementary Table 3. Changes in gene expression were evaluated via the 2 − ΔΔCT method. The materials and methods used in this study are described in detail in the Supplementary Materials.

### Immunofluorescence staining and confocal analysis

Immunofluorescence staining was conducted as previously described [16]. Primary antibodies against various target proteins were applied overnight at 4 °C, followed by incubation with secondary antibodies for 45 min and counterstaining with DAPI (D9542, Sigma, MO, USA) for 10 min. Images were captured via a fluorescence microscope (DP72, Olympus, Japan).

For confocal microscopy analysis, the samples were incubated with a rabbit anti-*FNDC5* antibody, a mouse anti-integrin αVβ5 antibody or a mouse anti-Talin1 antibody. Details of the antibodies used are provided in Supplementary Table 2. Confocal images were captured via a confocal microscope (TCS SP8 STED 3X; Leica). The materials and methods used in this study are described in detail in the Supplementary Materials.

### Coimmunoprecipitation (co-IP)

Protein A/G PLUS-Agarose (sc‐2003; Santa Cruz Biotechnology) and a rabbit anti-*FNDC5* antibody were used for immunoprecipitation. Details of the antibodies used are provided in Supplementary Table 2. The materials and methods used in this study are described in detail in the Supplementary Materials.

### RNA-seq and analysis

Total RNA was extracted from the C2C12 (NC), sh-*FNDC5*, and OE-*FNDC5* cell lines via Trizol (Invitrogen, USA) and processed as previously described [17]. Library construction and sequencing were performed by Sinotech Genomics Co., Ltd. (Shanghai, PRC). The SRA accession number PRJNA1125111 for the RNA-seq data is provided.

### ELISA for irisin quantification

Serum irisin levels were quantified via a commercially available ELISA kit (YX-E28649G, Elabscience) according to the manufacturer’s instructions.

### Sarcopenia mouse model and therapy

All animal experiments were approved by the Institutional Animal Care and Treatment Committee of the 920th Hospital of the Joint Logistics Support Force of the PLA (Approval No. 2023 − 121(science)-01). The 32-week senescence-accelerated mouse P8 (SAMP8) strain has been established as a reliable model for studying muscular aging [18–21]. Fourteen specific-pathogen-free male sarcopenia model mice were obtained from Hangzhou Ziyuan Laboratory Animal Technology Co., Ltd. (SCXK(Zhe)2019-0004), Zhejiang, China. All the mice were housed under specific-pathogen-free conditions with controlled humidity and a 12-hour light‒dark cycle at 20 °C in the Laboratory Animal Center.

At 32 weeks of age, the mice were anesthetized, and adeno-associated virus (AAV) (1 × 10^11^ vg) carrying the mouse *FNDC5* open reading frame cloned and inserted into the pAAV-CMV-MCS-EF1-GDGreen-WPRE vector (pAAV-CMV-*FNDC5*-3×FLAG -EF1-GDGreen-WRE) was injected into the unilateral gastrocnemius muscle via a 0.5 mL insulin syringe. The injected mice were sacrificed 4 weeks postinjection, and the gastrocnemius muscle tissues were collected, fixed in 4% paraformaldehyde overnight and then embedded into paraffin. Sections were used for haematoxylin and eosin (H&E) staining following the standard protocol.

### H&E staining and Immunohistochemistry (IHC)

Gastrocnemius sections were processed for H&E staining after tissue processing [16]. Immunohistochemistry was performed on muscle sections via primary antibodies against FNDC5, p-FAK, p-mTOR, and p-S6K, as listed in Supplementary Table 2. The sections were incubated with the corresponding secondary antibodies, followed by DAB development. The quantification of the IHC staining intensities was performed via ImageJ software. The materials and methods used in this study are described in detail in the Supplementary Materials.

### Statistical analysis

Data are presented as mean ± SD. Unless otherwise stated, experiments were repeated independently *N* = 3 times. Statistical significance was assessed using a two-tailed Student’s t-test for two-group comparisons or one-way ANOVA for multiple-group comparisons, as appropriate. Statistical analyses were performed using GraphPad Prism 8.0.2. ns, *P* > 0.05; *P < 0.05; **P < 0.01; ***P < 0.001.

## Results

### Overexpression of *FNDC5* via gene delivery promoted proliferation and suppressed myogenic differentiation in vitro

Although many studies have shown that the product of *FNDC5* gene, irisin promotes myogenic differentiation of C2C12 myoblasts [8, 22], the long-term therapeutic application involving *FNDC5* gene delivery in C2C12 cells is still limited.

**Fig. 1:**
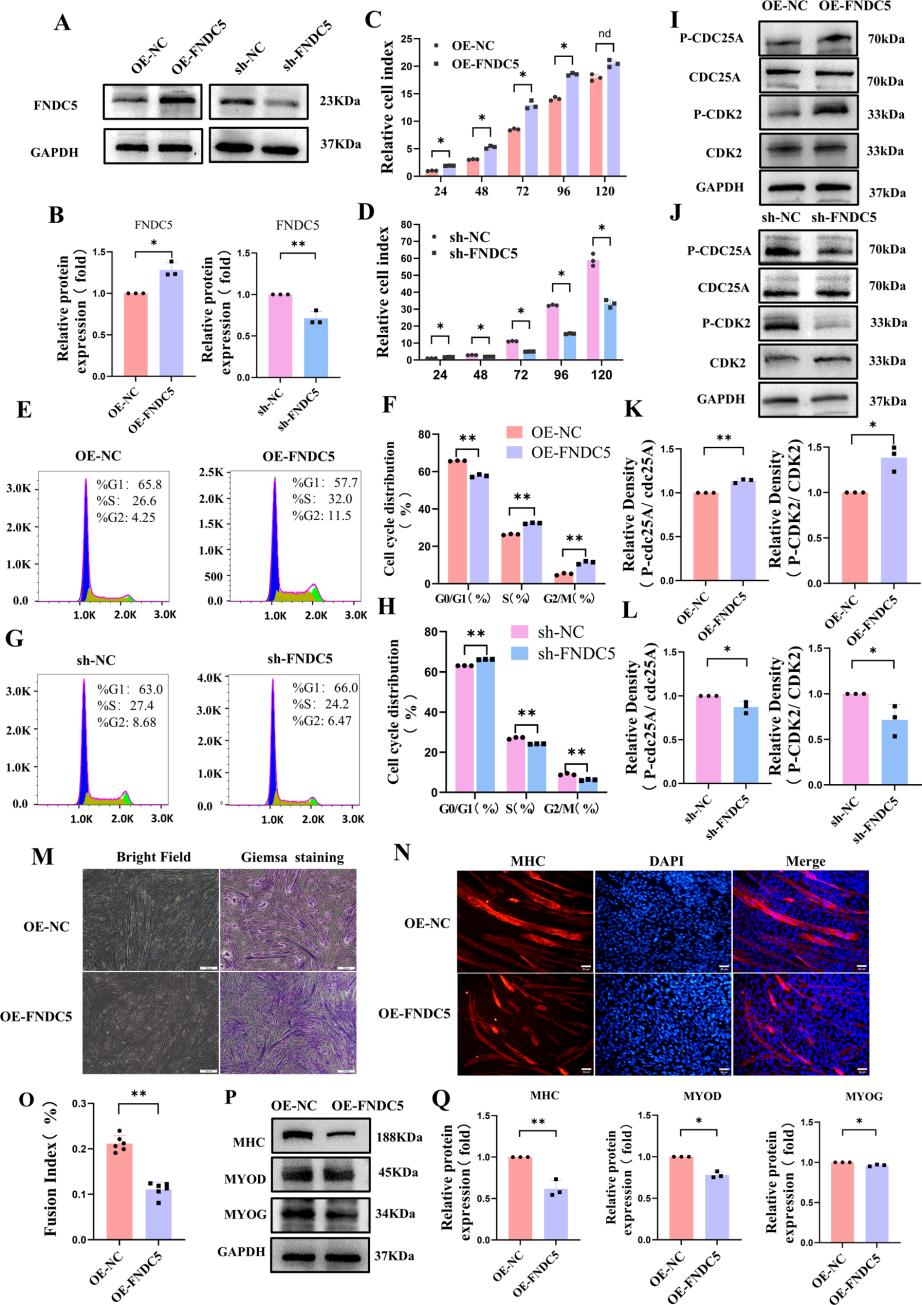
Overexpression of *FNDC5* enhances myoblasts’ proliferation and inhibits myogenic differentiation. **A**, **B** FNDC5 overexpression (OE-FNDC5) and knockdown (sh-FNDC5) in C2C12 cells were confirmed at the protein level by Western blotting (N = 3). **C**, **D** Cell prolif-eration was monitored using real-time cell analysis (RTCA). The cell index (CI) is an impedance-based readout reflecting changes in cell number and attachment/spreading (see Supplementary Materials and Methods for details). Quantitative analysis of CI at the indicated time points is shown for OE-FNDC5 vs. OE-NC and sh-FNDC5 vs. sh-NC (N = 3). **E**–**H** Cell-cycle distribution was analyzed by flow cytometry. Repre-sentative profiles and quantification of the percentages of cells in G0/G1, S, and G2/M phases are shown for OE-FNDC5 vs. OE-NC (**E**, **F**) and sh-FNDC5 vs. sh-NC (**G**, **H**) (N = 3). **I**–**L** Western blot analysis of key proteins involved in the G1/S checkpoint in OE-FNDC5 vs. OE-NC and sh-FNDC5 vs. sh-NC cells (N = 3). **M** Representative bright-field images and Giemsa staining of OE-FNDC5 and OE-NC cells after 4 days of myogenic differentiation. Scale bar, 100 μm. **N** Immunofluorescence staining of OE-FNDC5, OE-NC, sh-FNDC5, and sh-NC cells after 4 days of myogenic differenti-ation. For fusion index quantification, two randomly selected, non-overlapping fields were acquired per experiment (*N* = 3)per group（Scale bar, 50 μm）. **O** Quantifica-tion of the myotube fusion index on the basis of the MHC staining results. **P**, **Q** West-ern blotting and relative expression levels of the myogenic differentiation-related pro-teins MHC, MYOD, and MYOG in OE-FNDC5 cells compared with OE-NC cells subjected to myogenic differentiation for 4 days. (∗*P* < 0.05, ∗∗*P* < 0.01, ∗∗∗*P* < 0.001)

To comprehensively understand the role of *FNDC5* in treating patients with sarcopenia via gene therapy, we initially transduced C2C12 murine myoblast cells with the *FNDC5* gene to establish stable overexpressing cell lines (OE-*FNDC5*) and their corresponding control ones (OE-NC). Similarly, stable knockdown cell lines (sh-*FNDC5* vs. sh-NC) were generated. Verification of the expression of exogenous *FNDC5* genes was conducted at the protein level (Fig. 1A, B). Compared with control cells, OE-*FNDC5* cells presented 1.28 ± 0.09-folds greater expression at the protein level, whereas sh-*FNDC5* cells presented 0.71 ± 0.08-fold lower expression at the protein level.

To elucidate the biological functions of *FNDC5* in C2C12 cell growth, real-time cell analysis (RTCA) was employed. Compared with OE-NC cells, OE-*FNDC5* cells exhibited a significant increase in cell growth, with relative rates of the cell index being 2.09 ± 0.76, 1.79 ± 0.41, 1.50 ± 0.30, 1.32 ± 0.17, and 1.14 ± 0.07 at time points 24, 48, 72, 96, and 120 h, respectively. In contrast, sh-*FNDC5* cells exhibited the opposite results (Fig. 1C, D). Cell cycle analysis further revealed that OE-*FNDC5* promoted the progression of the cell cycle from the G0/G1 phase to the S phase (S phase: 26.33 ± 0.31% vs. 32.33 ± 0.31%), whereas sh-*FNDC5* inhibited this process (S phase: 27.1 ± 0.52% vs. 24.13 ± 0.16%) (Fig. 1E-H). The differential expression of the G1/S phase protein markers CDC25 and CDK2 under OE-*FNDC5* and sh-*FNDC5* conditions supports these findings (Fig. 1I-L), suggesting that *FNDC5* may increase DNA replication, thereby promoting cell growth.

Additionally, in the wound healing and transwell assays, OE-*FNDC5* cells displayed significantly enhanced migratory ability, whereas the migratory ability of sh-*FNDC5* cells during the same period was diminished compared with that of the sh-NC group (Supplementary Fig. S1 A, B, C, D). These results indicate a positive correlation between *FNDC5* expression and the proliferation and migration of C2C12 cells in vitro.

To further investigate the biological roles of *FNDC5* in C2C12 cells, we conducted in vitro myogenic differentiation induction assays on *FNDC5*-regulated cells and their respective controls. After 4 days of myogenic differentiation induction, morphological differences in myogenic differentiation were observed through brightfield and Giemsa staining (Fig. 1M). Immunofluorescence staining for myosin heavy chain (MHC) revealed markers of myogenic differentiation, and statistical analyses were performed to determine the fusion index in OE-*FNDC5* cells. The results revealed that the fusion indices of OE-NC and OE-*FNDC5* cells were 0.21 ± 0.02 and 0.11 ± 0.04, respectively. These results indicate slight inhibition of myogenic differentiation in OE-*FNDC5* cells compared with OE-NC cells (Fig. 1N-O). Western blotting for myogenic differentiation-related proteins, such as MHC, MYOD and MYOG, confirmed these observations (Fig. 1P, Q).

### *FNDC5* overexpressing enhances FAK/AKT signaling via integrin αVβ5

**Fig. 2:**
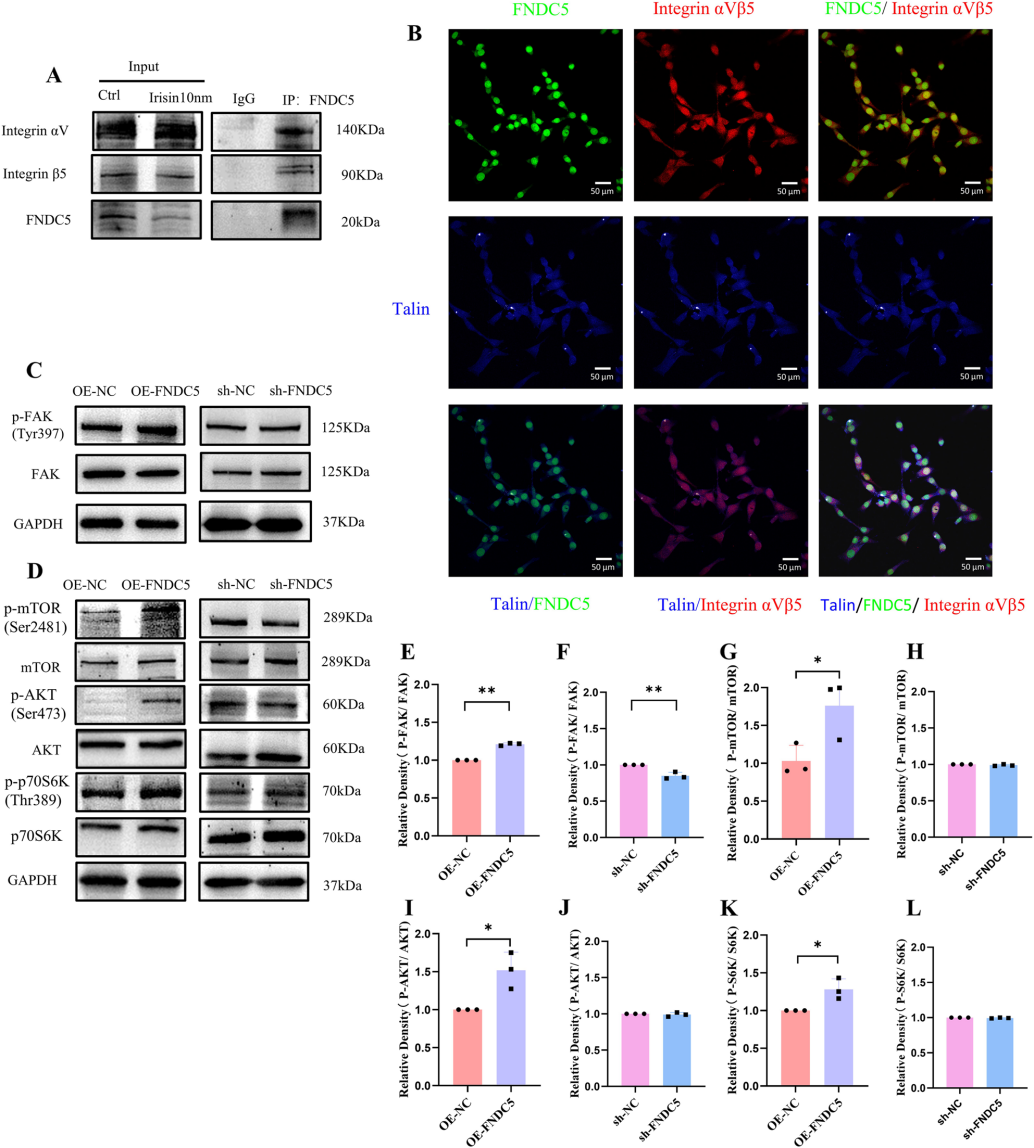
Interaction of irisin and integrin αVβ5 triggers FAK/AKT signaling in myoblast C2C12 cells. **A** Co-immunoprecipitation (co-IP) of irisin and integrin αVβ5 in C2C12 cells with or without 10 nM irisin treatment. **B** Representative immunofluorescence images showing colocalization of integrin αVβ5 and irisin/FNDC5 in C2C12 cells 2 h after treatment with 10 nM irisin. Scale bar, 50 μm. **C**, **E**, **F** Western blot analysis and relative phosphorylation levels of FAK in OE-FNDC5, OE-NC, sh-FNDC5, and sh-NC cells after 4 days of myogenic differentiation (N = 3). **D**, **G**, **I**, **K** Western blot analysis and relative phosphorylation levels of p-mTOR, p-AKT, and p-S6K in OE-FNDC5 vs. OE-NC cells after 4 days of myogenic differentiation (*N* = 3). **H**, **J**, **L** Western blot analysis and relative phosphorylation levels of p-mTOR, p-AKT, and p-S6K in sh-FNDC5 vs. sh-NC cells after 4 days of myogenic differentiation (*N* = 3). ( *P < 0.05, **P < 0.01, ***P < 0.001)

It was known that exogenous irisin interacts with integrin αVβ5 on the surface of osteoblasts. This prompted us to investigate whether endogenously produced irisin, resulting from *FNDC5* overexpression, similarly exerts its effects on myoblasts specifically, whether it promotes their proliferation in a manner analogous to its role in osteoblasts. Initially, coimmunoprecipitation assays were conducted, confirming that endogenous irisin produced by overexpressed *FNDC5* gene directly interacts with integrin αVβ5 under both native and exogenous conditions (Fig. 2A). To further validate this interaction, we analyzed the colocalization of these proteins via confocal microscopy with immunofluorescence staining. The colocalization of endogenous irisin, integrin αVβ5 and the focal adhesion marker Talin, which is used as a membrane marker, was clearly observed on the cell surface (Fig. 2B).

To explore the downstream consequences of this interaction, we performed semiquantitative western blots of key signaling molecules. We detected significant upregulation of p-FAK (Tyr397) in OE-*FNDC5* cells compared with control cells (Fig. 2C). Moreover, we detected significant downregulation of p-FAK (Tyr397) in sh-*FNDC5* cells (Fig. 2C). Specifically, the level of p-FAK (Tyr397) in OE-*FNDC5* cells was 1.21 ± 0.018-fold greater than that in control cells. In contrast, the level of p-FAK (Tyr397) in sh-*FNDC5* cells was 0.85 ± 0.05-fold greater than that in control cells (Fig. 2E, F).

Given our previous findings linking *FNDC5* to muscle cell proliferation, we further investigated proteins associated with cell proliferation and protein synthesis, namely, AKT and mTOR. The activation state of the mTOR/AKT pathway was assessed, revealing that the phosphorylation levels of mTOR (p-mTOR Ser2481), AKT (p-AKT Ser473), and S6K (p-S6K Thr389) were 1.72 ± 0.37, 1.52 ± 0.24 and 1.21 ± 0.05-fold greater, respectively, in OE-*FNDC5* cells than in control cells (Fig. 2D, G, I, K). In contrast, the relative phosphorylation levels of mTOR (p-mTOR Ser2481), AKT (p-AKT Ser473) and S6K (p-S6K Thr389) in sh-*FNDC5* cells were 0.99 ± 0.03, 0.99 ± 0.01 and 0.99-fold greater, respectively, than those in control cells (Fig. 2D, H, J, L).

### Integrin αVβ5 inhibition reverses the effects of *FNDC5* overexpression on C2C12 myoblasts

**Fig. 3:**
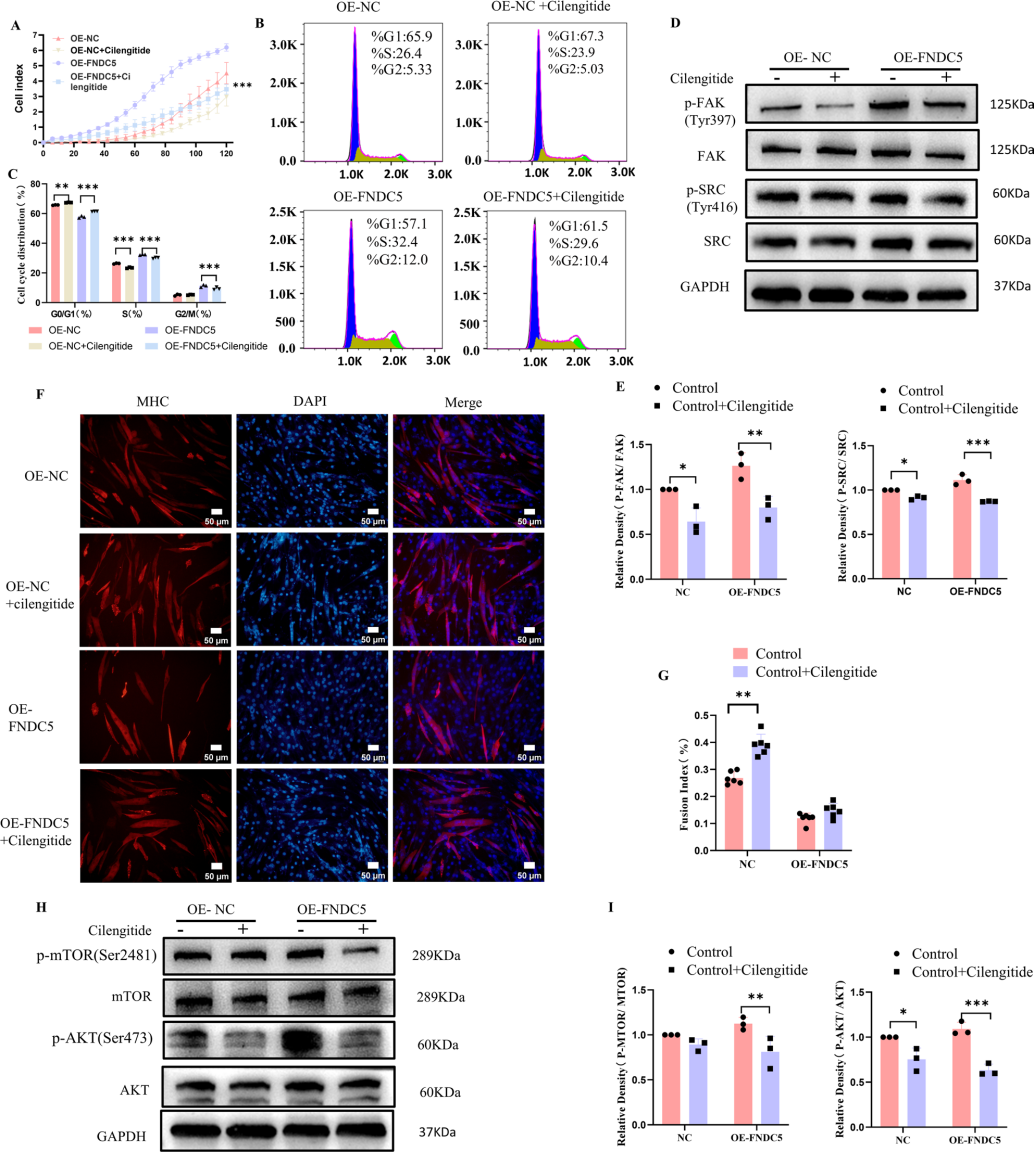
The inhibitor cilengitide reverses *FNDC5* functioning on myoblasts. **A** Cell proliferation was monitored using real-time cell analysis (RTCA). Representative cell index (CI) readouts and quantification of CI at the indicated time points are shown for OE-FNDC5 vs. OE-NC (*N* = 3). **B**, **C** Cell-cycle distribution of OE-NC and OE-FNDC5 cells treated with or without 0.5 µM cilengitide. Representative profiles (**B**) and quantification of the percentages of cells in each phase (**C**) are shown (N = 3). **D**, **E** Western blot analysis and relative phosphorylation levels of FAK and SRC in OE-FNDC5 vs. OE-NC cells (N = 3). **F** Immunofluorescence staining of OE-FNDC5, OE-NC cells treated with or without cilengitide for 4 days during myogenic differentiation. For fusion index quantification, two randomly selected, non-overlapping fields were acquired per experiment (N = 3)per group(Scale bar, 50 μm). **G** Quantification of the myotube fusion index based on the MHC staining results. **H**, **I** Western blot analysis and relative phosphorylation levels of mTOR and AKT in OE-NC and OE-FNDC5 cells treated with or without cilengitide for 4 days during myogenic differentiation (*N* = 3). ( *P < 0.05, **P < 0.01, ***P < 0.001)

To further investigate whether the integrin αVβ5/FAK axis is crucial for promoting cell proliferation in OE-*FNDC5* cell lines, cilengitide, a well-known integrin αVβ3/5 inhibitor, was added to cultured OE-*FNDC5* and NC cells. As expected, significant suppression of cell growth was observed in both OE-*FNDC5* and OE-NC cells; the inhibition rates at the 120-hour time point were 23.00 ± 5.31% and 43.95 ± 6.13%, respectively. These findings indicate that the inhibition rate in OE-*FNDC5* cells was 1.91 ± 0.52-fold greater than that in OE-NC cells, possibly due to increased binding of irisin and integrin αVβ5 (Fig. 3A). Correspondingly, cell cycle assays revealed a block from the G0/G1 to the S phase in both the OE-NC and OE-*FNDC5* groups: 57.77 ± 0.71% vs. 61.43 ± 0.12% and 65.77 ± 0.16% vs. 67.43 ± 0.16%, respectively (Fig. 3B, C).

To corroborate the cellular functional results described above, the key proteins involved in the FAK‒mTOR signaling pathway related to cell proliferation were analyzed under cilengitide treatment. As expected, the relative phosphorylation levels of p-FAK(Tyr397) and p- SRC (Tyr416) were decreased in both the OE-NC and OE-*FNDC5* groups: 1VS 0.64 ± 0.15 and 1.26 ± 0.14 VS 0.80 ± 0.12; 1 VS 0.91 ± 0.02 and 1.11 ± 0.05 VS 0.87 (Fig. 3D, E).

Furthermore, bright field and Giemsa staining (Supplementary Fig. S2A), as well as immunofluorescence staining for MHC and analysis of the fusion index (Fig. 3F, G), confirmed that cilengitide improved myotube formation. The expression levels of key myogenic differentiation markers (MHC, MYOD, MYOG) further validated this effect in both OE-NC and OE-*FNDC5* cells (Supplementary Fig. S2B). Additionally, we assessed the expression of proliferation-related marker proteins. Western blotting revealed that cilengitide inhibited the expression of the proliferation-related markers mTOR (p-mTOR Ser2481) and AKT (p-AKT Ser473) in both the OE-NC and OE-*FNDC5* groups: 1 vs. 0.89 ± 0.06 and 1.12 ± 0.07 vs. 0.81 ± 0.17; 1 vs. 0.75 ± 0.13 and 1.09 ± 0.07 vs. 0.63 ± 0.07 (Fig. 3H, I).

### *FNDC5* overexpression promotes extracellular matrix protein expression

**Fig. 4:**
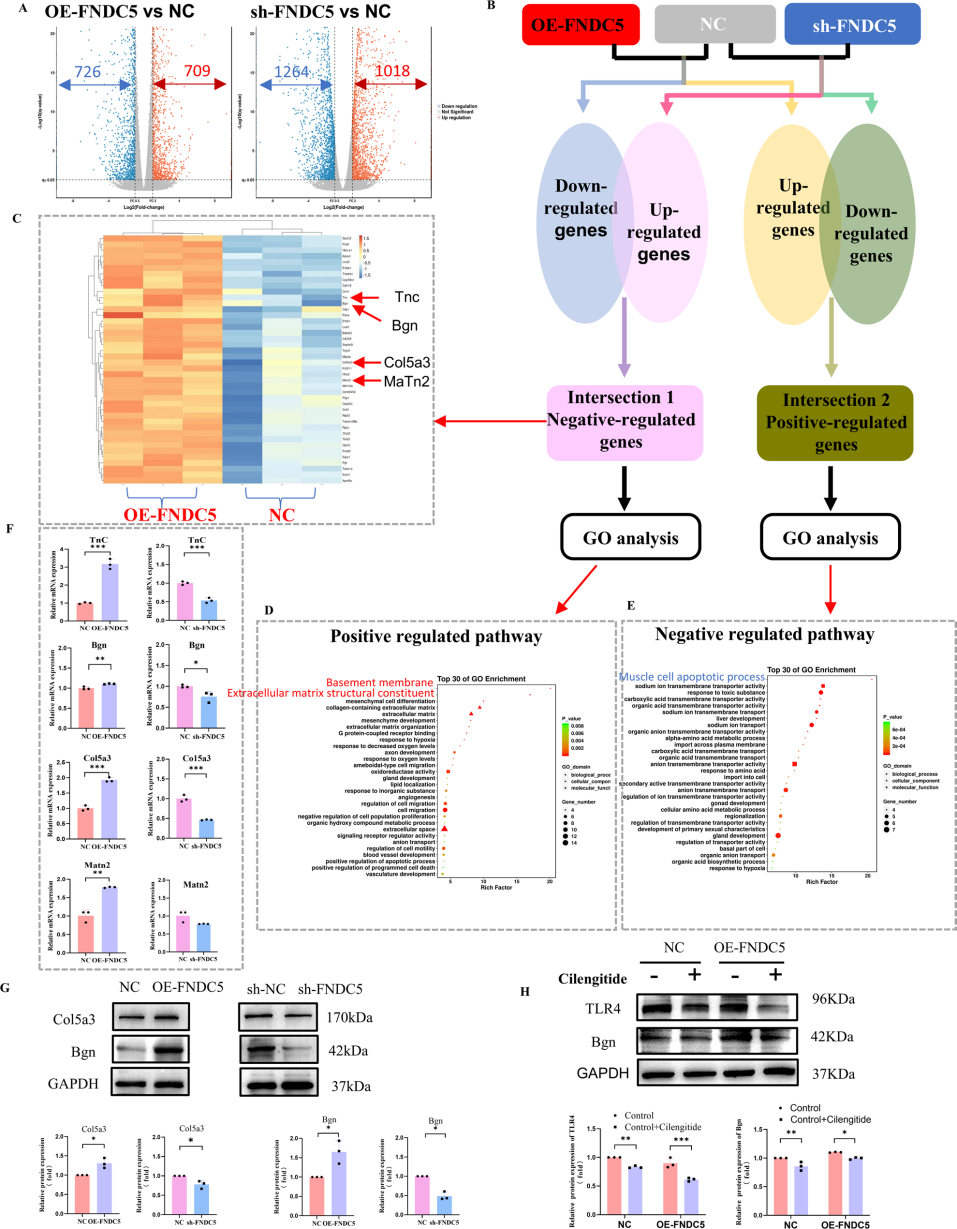
Over-expressing of *FNDC5* enhances ECM protein expression in myoblasts. **A** RNA-seq was performed to compare OE-FNDC5, sh-FNDC5, and corresponding negative control (NC) cells (biological replicates as described in the Methods). Differentially expressed genes (DEGs) were defined using fold-change cutoffs of > 1.5 or < 0.67 and an adjusted *P* value (FDR) < 0.05.**B** Workflow for selecting positively and negatively regulated genes. **C**–**E** Gene Ontology (GO) enrichment analysis of positively and negatively regulated gene sets. Enriched terms/pathways were considered significant at adjusted P (FDR) < 0.05. **F** qPCR validation of TNC, BGN, COL5A3, and MATN2 expression in OE-FNDC5 vs. OE-NC and sh-FNDC5 vs. sh-NC (N = 3). **G** Western blot analysis of COL5A3 and Bgn in OE-FNDC5 vs. OE-NC and sh-FNDC5 vs. sh-NC (N = 3). **H** Western blot analysis of Bgn and its receptor TLR4 in OE-FNDC5 and OE-NC cells treated with or without cilengitide (*N* = 3). ( *P < 0.05, **P < 0.01, ***P < 0.001)

To elucidate the mechanism by which *FNDC5* over-expression promotes cell proliferation, we conducted RNA-Seq to identify differentially expressed genes (DEGs) between *FNDC5* gene regulated C2C12 cells (OE-*FNDC5* and sh-*FNDC5*) and control C2C12 cells (NC). We identified 1435 DEGs (709 upregulated and 726 downregulated) between OE-*FNDC5* and NC and 2282 DEGs (1018 upregulated and 1264 downregulated) between sh-*FNDC5* and NC (Fig. 4A). DEGs were selected using a cutoff of 1.5-fold higher or 0.67-fold lower expression than that of the controls. The positively regulated genes were highly expressed in OE-*FNDC5* vs. NC (fold change > 1.5) and expressed at low levels in sh-*FNDC5* vs. NC (fold change < 0.67), whereas the negatively regulated genes presented the opposite pattern (Fig. 4A).

GO analyses of the identified DEGs revealed that *FNDC5* overexpression enhances pathways related to the “basement membrane” and “extracellular matrix structural constituent,” suggesting increased cell adhesion (Fig. 4C, D, E). qPCR assays validated these RNA-Seq results, with a focus on genes involved in these pathways, such as *BGN*,* TNC*,* COL5A3*,* and MATN2* (Fig. 4F). Western blot assays confirmed the expression of BGN and COL5A3, which was consistent with the RNA-Seq findings (Fig. 4G).

Previous studies have indicated that Bgn expression is regulated by the mTOR/AKT axis, influencing cell adhesion and proliferation [23]. Given that TLR4 acts as a receptor for BGN, we investigated BGN/TLR4 signaling activation. The protein expression of Bgn and TLR4 was inhibited by cilengitide treatment (Fig. 4H). Thus, the *FNDC5* product, irisin, binds to integrin αVβ5 on C2C12 cells, triggering the FAK‒mTOR axis and promoting cell proliferation by enhancing cell adhesion during C2C12 myogenic differentiation. This process can be blocked by the integrin αVβ5 inhibitor cilengitide.

### *FNDC5* gene delivery alleviates sarcopenia in SAMP8 mouse model

**Fig. 5:**
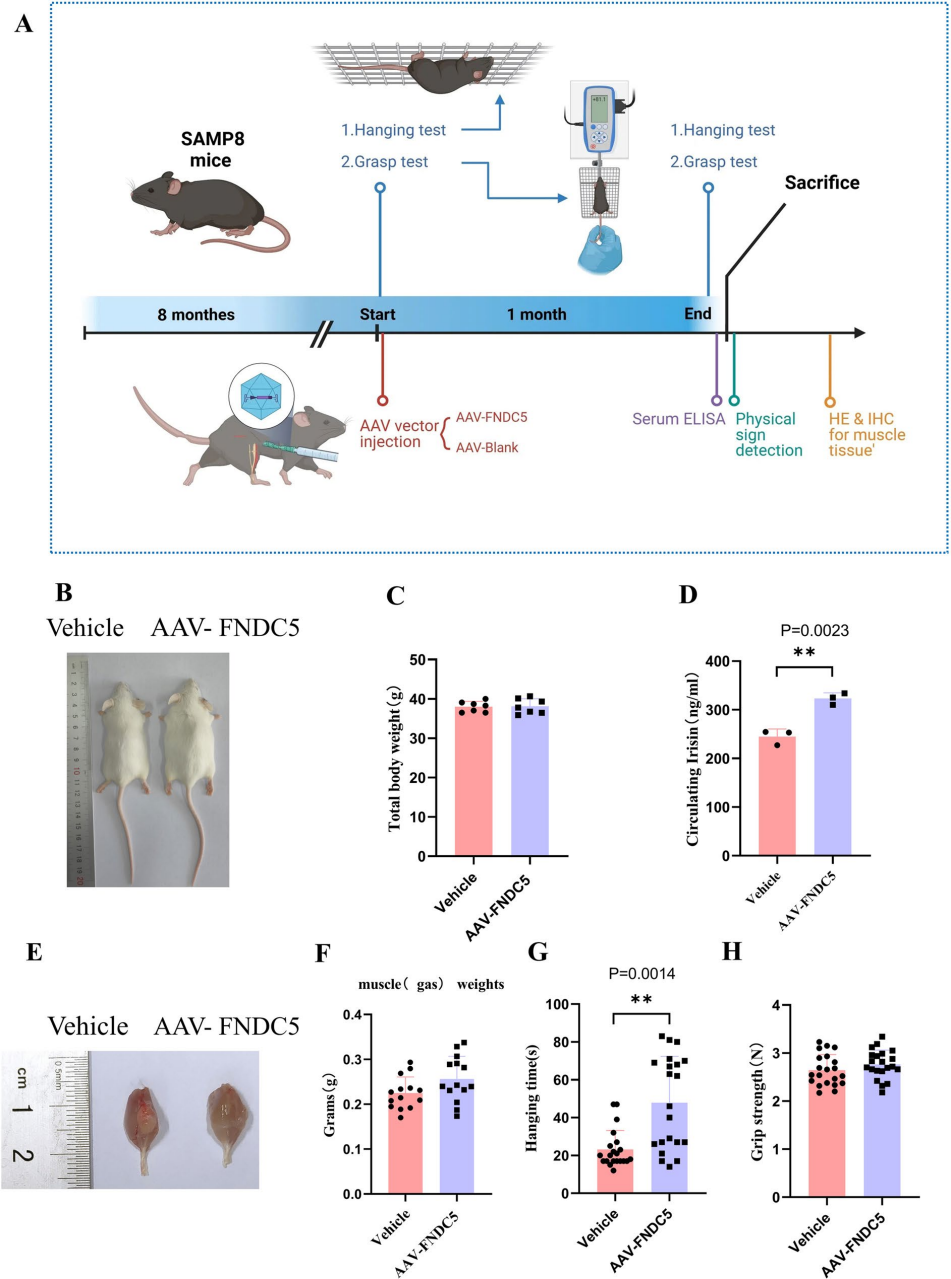
Application of AAV-*FNDC5* delivery attenuates the phenotype in sarcopenia mice model. **A** Experimental timeline schematic. AAV-vehicle or AAV-FNDC5 was injected into the gastrocnemius muscle of sarcopenic mice (N = 7 mice per group). Mice were euthanized 4 weeks after AAV injection; blood was collected for ELISA, and tissues were processed for H&E staining and IHC analysis. **B**, **C** Representative general appearance (**B**) and body weight (**C**) of sarcopenic mice prior to euthanasia. **D** ELISA analysis of circulating irisin levels in sarcopenic mice 4 weeks after AAV injection. **E**, **F** Representative images (**E**) and weights (**F**) of gastrocnemius muscles. Gastrocnemius weight was recorded bilaterally ( *N* = 14). **G**, **H** Hanging time (**G**) and grip strength (**H**) of sarcopenic mice 4 weeks after AAV injection ( *N* = 21). ( *P < 0.05, **P < 0.01, ***P < 0.001)

Our previous results demonstrated that increasing *FNDC5* expression in vitro enhances its binding to integrin αVβ5 on the cell surface, thereby activating the mTOR pathway and promoting cell proliferation. To validate these findings in vivo, we tested the effects on sarcopenic sarcopenia model mice via an AAV vector (Fig. 5A). Seven 8-month-old sarcopenia model mice in each group received a single dose of either AAV-*FNDC5* or AAV-Blank. No notable changes in body morphology (Fig. 5B) or body weight (Fig. 5C) were observed between the vehicle and AAV-*FNDC5* groups. ELISA confirmed higher serum irisin levels in the AAV-*FNDC5* delivery group than in the control group at 1month postinjection (Fig. 5D).

Both groups underwent muscle function tests, including hanging and grip strength assays, before and 1 month after injection. Upon sacrifice, gastrocnemius muscle tissues were isolated, quantified, and subjected to IHC staining for specific proteins. Gastrocnemius muscle mass was slightly greater in the AAV-*FNDC5* group than in the control group (Fig. 5E, F). Muscle function assays revealed that mice receiving AAV-*FNDC5* treatment presented approximately 2.06-folds longer hanging times (47.86 ± 24.51 s vs. 23.19 ± 9.98 s), indicating improved muscle strength, whereas no significant change in grip strength was observed (Fig. 5G, H).

### *FNDC5* gene delivery promotes muscle bundle formation through FAK/AKT pathway activation

**Fig. 6:**
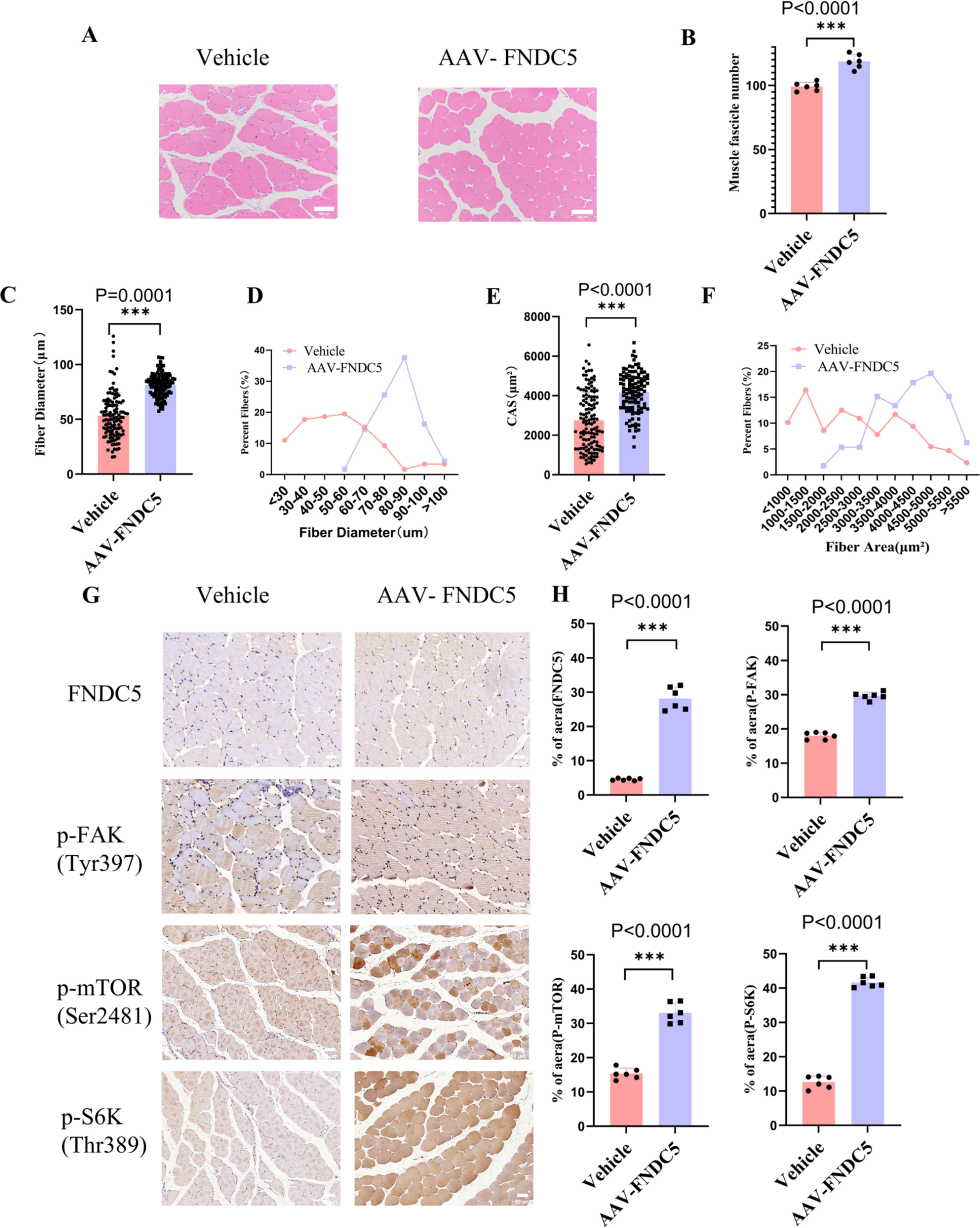
*FNDC5* introduction activates the FAK/AKT cascade in muscle cells and increases muscle bundles. **A** Representative H&E-stained sections of gastrocnemius muscles from the AAV-vehicle and AAV-FNDC5 groups, with corresponding quantification. **B** Frequency distribution of the number of muscle fascicles in gastrocnemius muscle sections (*N* = 6). **C**–**F** Frequency distributions of gastrocnemius muscle fiber diameter and cross-sectional area (CSA) in the AAV-vehicle and AAV-FNDC5 groups (*N* = 3). **G**, **H** Representative immunohistochemical staining and quantification of staining intensities for irisin, p-FAK, p-mTOR, and p-S6K in gastrocnemius muscles from the AAV-vehicle and AAV-FNDC5 groups (N = 6). Scale bar, 50 μm. (*P < 0.05, **P < 0.01, ***P < 0.001)

**Fig. 7:**
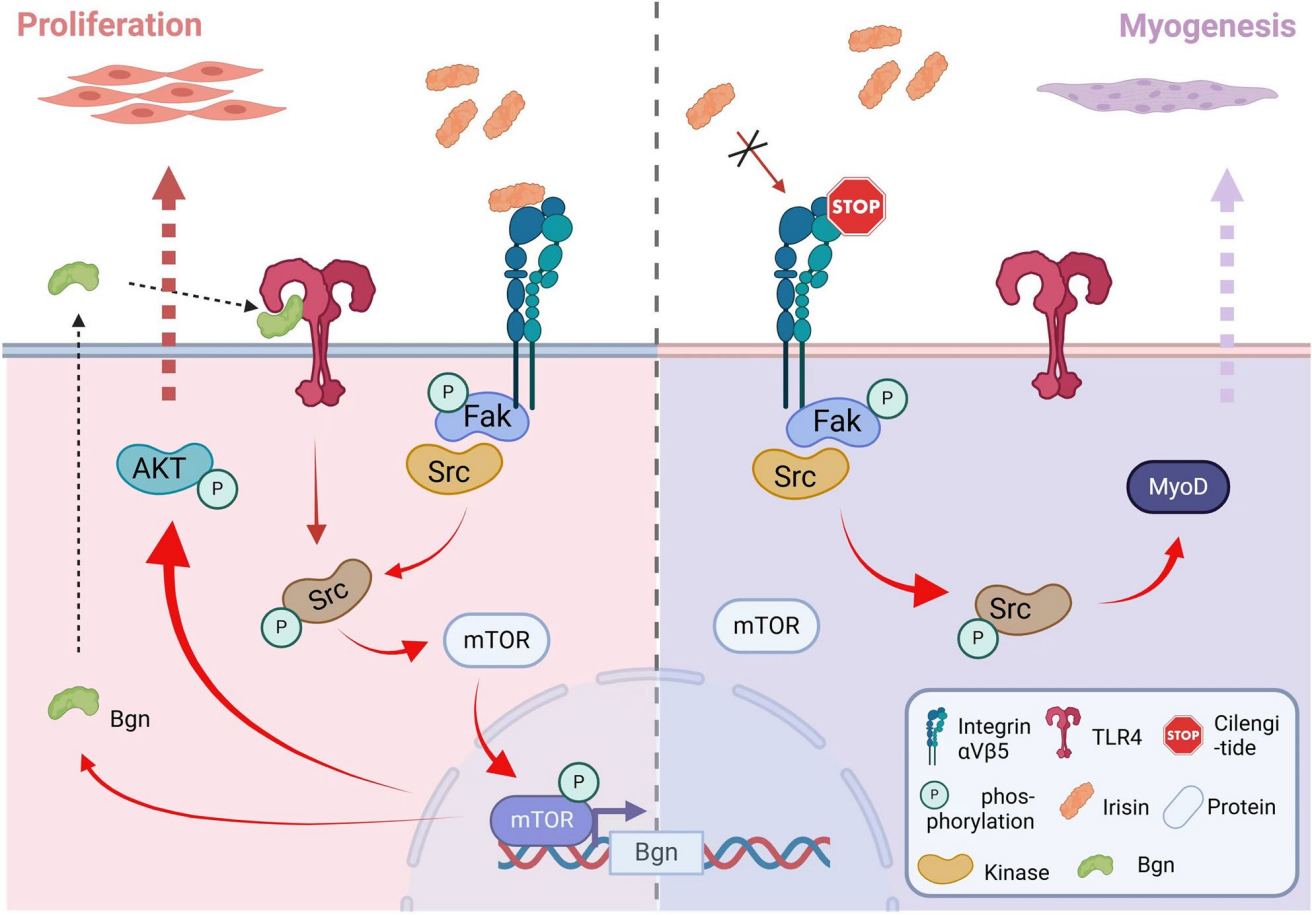
Integrin αVβ5 as a switch regulating the proliferation and differentiation of Myoblasts under FNDC5 treatment. Diagram illustrating the role of integrin αVβ5 in regulating C2C12 cell fate under irisin supplementation: When integrin αVβ5 is unblocked, C2C12 cells exhibit increased proliferation compared with differentiation during irisin treatment. When integrin αVβ5 is blocked, C2C12 cells exhibit greater myogenic differentiation than proliferation under irisin treatment

To further investigate the observed changes in the mouse model, HE and IHC staining for proteins related to cell proliferation were conducted on muscle tissue from both the AAV-vehicle and AAV-*FNDC5* groups. *FNDC5* introduction resulted in an approximately 20% greater number of muscle fascicles (99.17 ± 3.31 vs. 118.83 ± 5.56) (Fig. 6A, B). Additionally, compared with the vehicle group, the AAV-*FNDC5* group presented significant increases in muscle fiber diameter and cross-sectional area (CSA) (Fig. 6C-F) (Fig. 7).

Immunohistochemical staining revealed elevated expression levels of irisin, p-FAK, p-mTOR and p-S6K within muscle fibers in the AAV-*FNDC5* group than in the control group (Fig. 6G). Quantitative analysis revealed that the positive rates of irisin, p-FAK (Tyr397), p-mTOR (Ser2481) and p-S6K (Thr389) staining were 6.17 ± 0.87, 1.65 ± 0.11, 2.15 ± 0.61, and 3.31 ± 0.73 times greater than those in the control group, respectively (Fig. 6H). These results confirm the findings from the in vitro assays, which revealed significant upregulation of myoblast proliferation and subsequent increases in muscle bundles.

## Discussion

Growing evidence implicates FNDC5/irisin as a mechanistically relevant, exercise-responsive axis in age-related muscle loss (sarcopenia). In humans, skeletal muscle PGC-1α and FNDC5 transcription can increase following structured training programs; however, responses vary across cohorts and exercise regimens, and circulating irisin often exhibits a transient post-exercise increase rather than a sustained elevation [24]. In aged models, FNDC5/irisin declines with aging, and genetic or pharmacologic evidence supports a functional role in sarcopenia: aerobic training improves sarcopenic phenotypes in aged mice, but these benefits are attenuated when FNDC5 is disrupted, supporting a causal contribution of this pathway to exercise-mediated muscle maintenance [25]. Consistently, multiple in vivo and in vitro studies report that exogenous irisin can counteract muscle atrophy by suppressing canonical catabolic programs (e.g., MURF1/ATROGIN-1) and supporting anabolic signaling, including mTORC1-associated protein synthesis and mitochondrial homeostasis [26].

Mechanistically, the long-sought irisin receptor has been identified as αV-class integrins, with biophysical and functional data supporting interaction interfaces involving αV/β5. This mechanism was initially established in osteocytes and adipocytes, where integrin blockade abrogates irisin-induced signaling and biological effects [10]. Because integrin engagement canonically couples to focal adhesion signaling (FAK) and downstream growth/metabolic nodes (including AKT–mTOR and AMPK modules), this receptor framework provides a plausible route through which FNDC5/irisin may influence myoblast behavior and muscle remodeling in sarcopenia, while acknowledging that tissue-specific receptor utilization and context-dependent signaling in skeletal muscle remain active areas of investigation [10].

Here, we report a previously unrecognized role for integrin αVβ5 in regulating myoblast fate under FNDC5/irisin supplementation in a sarcopenia-relevant setting. We demonstrate that FNDC5 overexpression enhances myoblast proliferation through interaction with integrin αVβ5, leading to activation of the FAK/SRC complex and downstream mTOR signaling. This activation increases the expression of extracellular matrix (ECM) components, including BGN, TNC, COL5A3, and MATN2. Importantly, BGN–TLR4 engagement further amplifies the AKT/mTOR cascade, accelerating cell-cycle progression and promoting myoblast proliferation. Pharmacologic blockade of integrin αVβ5 with cilengitide diminishes the pro-proliferative effect of FNDC5 overexpression, while promoting myogenic differentiation. Collectively, these findings identify integrin αVβ5 as a pivotal determinant of myoblast fate during FNDC5/irisin supplementation and suggest that the timing of FNDC5/irisin administration may critically influence therapeutic efficacy in senile sarcopenia.

Consistent with our observations, satellite cell activation and proliferation are widely recognized as key determinants of skeletal muscle hypertrophy and regenerative capacity. Notably, experimental suppression of satellite-cell proliferation markedly blunts the hypertrophic response to chronic overload, underscoring the requirement for proliferative expansion and subsequent myonuclear accretion during muscle growth and repair [27]. In this context, irisin has been reported to act as a pro-myogenic cue: recombinant irisin increases myoblast number and enhances fusion/myotube formation in vitro, with mechanistic studies implicating pathways such as ERK signaling and transcriptional upregulation of proliferation-associated markers [8]. Moreover, the identification of αV-class integrins (including αVβ5) as irisin receptors in multiple tissues—where inhibition of αV integrins suppresses irisin-triggered signaling—supports a receptor paradigm compatible with integrin-coupled FAK/SRC-centered signaling in muscle [10]. Finally, from an independent signaling perspective, dasatinib, a clinically used SRC-family kinase inhibitor, has been reported to perturb myogenic programs and impair normal muscle regeneration, further reinforcing the functional importance of SRC/FAK-associated signaling modules in efficient muscle repair [28].

In conclusion, our study demonstrates that FNDC5 overexpression promotes myoblast proliferation by binding to integrin αVβ5 and activating a FAK/BGN/TLR4–AKT/mTOR signaling cascade. This pathway accelerates cell-cycle progression and enhances proliferative capacity, at least in part by strengthening cell–ECM interactions. Disrupting this interaction with cilengitide attenuates proliferation while favoring myogenic differentiation. To our knowledge, this is the first report establishing integrin αVβ5 as a key regulator of myoblast fate under FNDC5/irisin supplementation. These findings provide a mechanistic rationale for exploring FNDC5 augmentation strategies, potentially including gene therapy-based approaches, as a therapeutic avenue for sarcopenia.

## Supplementary Information

Supplementary Material 1.

Supplementary Material 2.

Supplementary Material 3.

Supplementary Material 4.

Supplementary Material 5.

Supplementary Material 6.

## Data Availability

The processed raw data needed to replicate these findings are available upon request.

## Abbreviations

AAV: Adeno–associated virus
AKT: Protein kinase B
AMPK: AMP–activated protein kinase
ANOVA: Analysis of variance
BGN: Biglycan
CDK2: Cyclin–dependent kinase 2
CDC25: Cell division cycle 25
Co: IP–Co–immunoprecipitation
COL5A3: Collagen type V alpha 3 chain
CSA: Cross–sectional area
CT: Cycle threshold
DAPI: 4′,6–diamidino–2–phenylindole
DEG: Differentially expressed gene
ECM: Extracellular matrix
ELISA: Enzyme–linked immunosorbent assay
FAK: Focal adhesion kinase
FNDC5: Fibronectin type III domain–containing protein 5
GAPDH: Glyceraldehyde–3–phosphate dehydrogenase
GO: Gene Ontology
H&E: Hematoxylin and eosin
IHC: Immunohistochemistry
MHC: Myosin heavy chain
mTOR: Mammalian target of rapamycin
MYOD: Myogenic differentiation 1
MYOG: Myogenin
NC: Negative control
OE: Overexpression
PI3K: Phosphoinositide 3–kinase
qPCR: Quantitative polymerase chain reaction
RNA: seq–RNA sequencing
RTCA: Real–time cell analysis
S6K: Ribosomal protein S6 kinase
SPF: Specific–pathogen–free
SRC: Proto–oncogene tyrosine–protein kinase Src
TNC: Tenascin C
TLR4: Toll–like receptor 4
UCP: Uncoupling protein
WPRE: Woodchuck hepatitis virus posttranscriptional regulatory element
