# Supplementary material for: Integrin αVβ5 regulates myoblast proliferation and differentiation in sarcopenia mice treated with FNDC5 gene delivery: Original article
Source: Skelet Muscle. 2026 Mar 17;16:28. doi: 10.1186/s13395-026-00420-x (PMC13347998; doi:10.1186/s13395-026-00420-x)
Supplement: Supplementary file 1 — Supplementary Material 1. [file 13395_2026_420_MOESM1_ESM.docx]

**Figure Legends**

**Fig. S1 *FNDC5/*irisin's Influence on C2C12 Cell Migration**

(A, B) Migration of C2C12 cells in OE-FNDC5, sh-FNDC5, and control groups at 20 h. Microscopic images show representative migration in each group. (Scale bar, 100μm). Quantification of cell migration in C2C12 cells. (C, D) Migration of OE-FNDC5, sh-FNDC5, and control groups using transwell assays (Scale bar, 100μm) and quantification of results. (∗P<0.05，∗∗P<0.01，∗∗∗P<0.001)

**Fig.S2 Effects of *FNDC5* Overexpression and Cilengitide Inhibition on C2C12 Myoblasts**

(A) Bright-field and Giemsa staining images depicting the myogenesis of C2C12 cells treated with Cilengitide for 4 days (Scale bar,100μm). (B, C) Western blotting results and statistical analysis of MHC, MYOD, MYOG intensities. (∗P<0.05，∗∗P<0.01，∗∗∗P<0.001)
