# Supplementary material for: Integrin αVβ5 regulates myoblast proliferation and differentiation in sarcopenia mice treated with FNDC5 gene delivery: Original article
Source: Skelet Muscle. 2026 Mar 17;16:28. doi: 10.1186/s13395-026-00420-x (PMC13347998; doi:10.1186/s13395-026-00420-x)
Supplement: Supplementary file 2 — Supplementary Material 2. [file 13395_2026_420_MOESM2_ESM.docx]

**Materials and Methods**

**1. Stable cell construction**

C2C12 cells were transduced with lentiviruses carrying either *FNDC5* overexpression (OE‑FNDC5) or shRNA‑mediated knockdown (sh‑FNDC5) constructs (Tsingke Biotechnology, Nanjing, China).

For *FNDC5* knockdown, three independent shRNA‑expressing lentiviral constructs targeting *FNDC5* were generated in the pLKO.5 backbone; their sequences are provided in Supplementary Table 1. Each construct was transiently transfected into C2C12 cells to assess knockdown efficiency. Based on preliminary qPCR validation, shFNDC5‑1 (target sequence: GAGGAGGATACGGAGTACATA) was selected for subsequent functional experiments, with sh‑NC serving as the negative control.

For transduction, C2C12 cells were seeded at 5×10⁴ cells/well in 24‑well plates. The following day, cells were incubated with 150 μL lentiviral particles at an MOI of 100. At 72 h post‑transduction, selection was performed using puromycin (4 μg /mL) for 144 h, after which cells were maintained in puromycin‑free medium. The vector maps for OE‑FNDC5 and sh‑FNDC5 lentiviral plasmids are shown in Supplementary Fig. S1, and detailed vector information is summarized in Supplementary Table 1.

**2. Cell culture, cell cycle analysis and differentiation induction**

Murine C2C12 myoblasts were acquired from BNCC, Henan, China, and cultured in complete medium (DMEM + 10% FBS and 1% penicillin-streptomycin) at 37°C. Cells were passaged when confluence reached 80%.

OE-FNDC5, OE-NC, sh-FNDC5 and sh-NC cells were seeded in 6-well culture plate. Cells were serum-starved overnight to synchronize, then cultured in complete medium for an additional 24 h before harvesting for flow cytometry analysis. Then cells were washed three times with PBS and fixed with 70% ethanol overnight at -20°C followed by being treated with 1 mg/mL RNase A at 37 °C for 40 min, and stained with 50 ug/mL propidium iodide (PI) at 4℃ for 1 h. Samples were detected with a FACS Calibur Flow Cytometry (Becton Dickinson, Franklin Lakes, NJ, USA). The proliferative index shows the ratio of cells in S and G2/M phases among 20,000 cells examined.

For myogenesis induction, C2C12 cells were differentiated by switching to differentiation medium (DMEM + 2% horse serum and 1% penicillin-streptomycin) when confluence reached 90%. Medium was refreshed every two days until myotube formation was observed.

**3.** **Wound healing and transwell assays**

Cells were seeded in a 6-well plate with complete medium (DMEM with 10% FBS). Then, cells were starved in serum-free medium for 6–8 h, and a 200 µl pipette tip was used to create a straight wound in the cell monolayers when the cells had reached ~100% confluence. The cells were washed once with PBS. The scratch was observed and imaged under a light microscope at 0 and 20 h, and the wound width was measured. All experiments were performed in triplicate.

For migration assay, C2C12 cells were seeded in 24-well transwell chambers (Corning, USA). Cell suspension was added to the top compartment and treated with PBS. After incubation at 37 °C for 24 h, the chambers were removed and the cells migrating to the lower surface were stained with 0.1% crystal violet and pictured (Solarbio, China). Data were expressed as an average of cells per field that migrated through micropores.

**4. Cell proliferation detection**

The proliferation of OE-FNDC5 and OE-NC, as well as sh-FNDC5 and sh-NC cells was monitored using the RTCA DP System (Agilent).

Experiments were carried out using the xCELLigence RTCA DP instrument (Agilent ) which was placed in a humidified incubator at 37°C and 5% CO2.

Cell proliferation experiments were performed using modified 16-well plates (E-plate, (Agilent)). Microelectrodes were attached at the bottom of the wells for impedance-based detection of attachment, spreading and proliferation of the cells. Initially, 100 µL of cell-free growth medium (10% FBS) was added to the wells. After leaving the devices at room temperature for 30 min, the background impedance for each well was measured. Cells were harvested from exponential phase cultures by a standardized detachment procedure using 0.05% Trypsin-EDTA (Invitrogen NV/SA, Merelbeke, Belgium) and counted automatically with a Scepter 2.0 device (Merck Millipore SA/NV, Overijse, Belgium) .Then,50 µL of the cell suspension was seeded into the wells (The cell seeding density was 3030 cells per cm²). After leaving the plates at room temperature for 30 min to allow cell attachment, in accordance with the manufacturer's guidelines, they were locked in the RTCA DP device in the incubator and the impedance value of each well was automatically monitored by the xCELLigence system and expressed as a Cell Index value (CI). Water was added to the space surrounding the wells of the E-plate to avoid interference from evaporation. For proliferation assays, the cells were incubated during five days in growth medium (10% FBS) and CI was monitored every two hours for the period. Three replicates of each cell concentration were used in each test.

**5. Giemsa Staining**

Giemsa staining of OE-FNDC5 cells and control cells undergoing myogenesis for 4 days. Cells were first washed with PBS twice and fixed in 4% paraformaldehyde for 10 min, after which the myotubes were stained with 10% Giemsa solution (G1010, Solarbio) for 40 min and then observed by an optical microscope (Leica).

**6. Western Blot Analysis and qPCR analysis**

Proteins were extracted in cooled RIPA buffer containing protease inhibitor mix (Sigma-Aldrich) and phosphatase inhibitors. Protein lysates were then sonicated and centrifuged at 12000 rpm for 15 minutes at 4°C and supernatants were collected. Protein concentration was calculated by using a BSA standard curved and Bradford assay (Bio-Rad). Proteins were resolved under reducing conditions by SDS-PAGE (sodium dodecyl sulfate polyacrylamide gel electrophoresis) in 9% polyacrylamide gels. Subsequently, proteins were transferred to PVDF Immobilon-FL membranes typically for 60-90 minutes at 100V. Membranes were then blocked with 5% BSA for 1 hour at room temperature and incubated at 4°C overnight with the corresponding primary antibodies. Next, membranes were washed and then incubated for 1 hour at room temperature with HRP-coupled secondary antibodies. Protein detection was performed with short incubation with the ECL reagent Luminata Classico Western HRP substrate. Details of antibodies used are provided in Supplementary Table 2.

EZ-press RNA Purification Kit (EZBioscience, Cat#B0004DP) was used to obtain total RNA from samples according to manufacturer^，^s instruction. Complementary DNA synthesis was performed using a reverse transcription kit (EZBioscience , Cat# A0010CGQ). The expression of genes were analyzed by ABI Prism 7500 Real‐Time PCR System with 2× SYBR Green qPCR Master Mix (EZBioscience , Cat# A0001-R1). The comparative Ct method was used to analyze the relative expression of genes. Relative gene expression was analyzed using the 2^−ΔΔCt method, with GAPDH as the internal control. Primers used are listed in Supplementary Table 3.

**7. Immunofluorescence Staining and Confocal Analysis**

The C2C12 cells were rinsed three times in PBS and washed in 0.3% Triton X-100 for 30 min, followed by blocking for 1 h at room temperature. Subsequently, these cells were incubated overnight in mouse anti-MHC(MF-20) at 4 ℃. The next day, the C2C12 cells were incubated with Alexa Fluor® 488 secondary antibody (ab150113; Abcam) for 1 h, and the cell nucleus was stained by DAPI (D9542, Sigma, MO, USA).

Myotubes were defined as MHC-positive tubular structures containing two or more nuclei to exclude dividing myoblasts. In each experiment, images from two randomly selected, non-overlapping fields were acquired across each dish using an Olympus microscope equipped with a DP72 digital camera (Olympus, Tokyo, Japan) with cellSens Standard software (v1.16, Olympus, Tokyo, Japan). Image analysis was performed in ImageJ. The fusion index was calculated as the percentage of all nuclei that were located within myotubes containing two or more nuclei. Three independent experiments were performed (N = 3). In each experiment, cells were cultured in an independent dish per group, and the fusion index was calculated separately for each field. For confocal analysis, samples were incubated with rabbit anti-FNDC5 antibody and either mouse anti-integrin αVβ5 antibody or mouse anti-Talin1 antibody. Details of the antibodies used are provided in Supplementary Table 2. Confocal images were captured using a confocal microscope(TCS SP8 STED 3X; Leica).

**8.Coimmunoprecipitation (CO-IP)**

C2C12 cells were treated with or without 10 nM irisin for 20 min. The cells were lysed with RIPA buffer containing multi-function protease/phosphatase inhibitors. After protein concentration was quantified, the samples were balanced to the same concentration, and then divided into two parts. Anti-*FNDC5* antibody or anti-IgG antibody was incubated with the protein samples overnight for immunoprecipitation.. Then , the samples were incubated with protein A agarose beads at 4℃ for 4 h. Finally, western blot was used to detect αV, β5 and FNDC5 proteins. The antibodies used in this experiment and their working dilution were listed in Supplementary Table 2.

**9. RNA-seq and analysis**

Total RNA was extracted from C2C12（NC）, sh-*FNDC5*, and OE-*FNDC5* cell lines using Trizol (Invitrogen, USA) and processed as previously described. Library construction and sequencing were performed by Sinotech Genomics Co., Ltd. (Shanghai, PRC). The SRA accession number PRJNA1125111 for the RNA-seq data is provided.

**10. ELISA for Irisin Quantification**

At 4 weeks post-injection, mice were anesthetized, blood was collected, and the mice were then sacrificed. Serum Irisin levels were quantified using a commercially available ELISA kit (YX-E28649G, Elabscience) according to the manufacturer's instructions.

**11. Sarcopenia mouse model and Therapy**

All animal experiments were approved by the Institutional Animal Care and Treatment Committee of the 920th Hospital of the Joint Logistics Support Force of PLA (Approval No. 2023-121(science)-01). The 32-week-old senescence-accelerated mouse P8(SAMP8) has been established as a reliable model for studying muscular aging[1-4]. Fourteen specific-pathogen-free male sarcopenia mice were obtained from Hangzhou Ziyuan Laboratory Animal Technology Co., Ltd (SCXK(Zhe)2019-0004), Zhejiang, China. All mice were housed under specific-pathogen-free conditions with controlled humidity and a 12-hour light-dark cycle at 20°C in the Laboratory Animal Center.

At 32 weeks of age, mice were anesthetized, and adeno-associated virus (AAV) (1×10^11^ vg) carrying mouse *FNDC5* open reading frame cloned into the pAAV-CMV-MCS-EF1-GDGreen-WPRE vector (pAAV-CMV-*FNDC5*-3×FLAG-EF1-GDGreen-WPRE) was injected into the unilateral gastrocnemius muscle using a 0.5mL insulin syringe. The injected mice were sacrificed 4 weeks post-injection and gastrocnemius muscle tissues were collected and fixed in 4% paraformaldehyde. .

**12.Grip strength test**

The muscle strength was measured by a grip strength device (DB025X, zhishuduobao biological technology, Beijing, China). The muscle strength of both forelimb and hindlimb was recorded. The grip strength test of each mouse was repeated three times.

**13.Hanging grid test**

In this assay, inverted hanging time was measured. A 45 × 45 cm grid (bar thickness, 2 mm; mesh, 18 mm) was placed on a 55-cm-high frame, and a 5-cm-thick cushion was placed under the grid. The distance between the grid and the cushion was 50 cm. We placed each mouse at the center of the grid and then turned the grid upside down with the mouse head declining first. Hanging time was recorded as the time until the mice fell. Each mouse was tested thrice with a >30 min interval between tests, and the hanging time was recorded and averaged.

**14. HE staining and Immunohistochemistry (IHC)**

Hematoxylin and eosin (HE) staining was carried out for the gastrocnemius muscle. The collected muscle tissue was fixed with 4% paraformaldehyde and dehydrated. The tissues were then sequentially soaked in xylene for transparency. The tissue was then soaked in paraffin. The wax-impregnated tissue blocks were sliced after the embedding treatment. The sections were dewaxed and hydrated with xylene and gradient ethanol, haematoxylin staining, hydrochloric acid differentiation, running water washing, eosin staining, running water washing, gradient ethanol and xylene dehydration and transparency, drying at room temperature, dropping neutral gum for sealing, observing under a microscope, and taking pictures.

Immunohistochemistry (IHC) was performed using an IHC Kit (Sigma-Aldrich, St. Louis, MO, USA), according to the manufacturer's instructions. It was performed on muscle sections using primary antibodies against FNDC5, p-FAK, p-mTOR, p-S6K listed in Supplementary Table 2. Sections were incubated with corresponding secondary antibodies followed by DAB development. Quantification of IHC staining intensities was performed using Image J software.

[1] Deng Z, Song C, Chen L, Zhang R, Yang L, Zhang P, et al. Inhibition of CILP2 Improves Glucose Metabolism and Mitochondrial Dysfunction in Sarcopenia via the Wnt Signalling Pathway. J Cachexia Sarcopenia Muscle 2024;15(6):2544-58. [eng].

[2] Wang C, Zhao B, Zhai J, Wang A, Cao N, Liao T, et al. Clinical-grade human umbilical cord-derived mesenchymal stem cells improved skeletal muscle dysfunction in age-associated sarcopenia mice. Cell Death Dis 2023;14(5):321. [eng].

[3] Liu C, Wong PY, Wang Q, Wong HY, Huang T, Cui C, et al. Short-chain fatty acids enhance muscle mass and function through the activation of mTOR signalling pathways in sarcopenic mice. J Cachexia Sarcopenia Muscle 2024;15(6):2387-401. [eng].

[4] Long Y-F, Cui C, Wang Q, Xu Z, Chow SK-H, Zhang N, et al. Low-Magnitude High-Frequency Vibration Attenuates Sarcopenia by Modulating Mitochondrial Quality Control via Inhibiting miR-378. J Cachexia Sarcopenia Muscle 2025;16(1):e13740. [eng].
