# Supplementary figures and images for: Integrin αVβ5 regulates myoblast proliferation and differentiation in sarcopenia mice treated with FNDC5 gene delivery: Original article (part 1 of 2)
Source: Skelet Muscle. 2026 Mar 17;16:28. doi: 10.1186/s13395-026-00420-x (PMC13347998; doi:10.1186/s13395-026-00420-x)

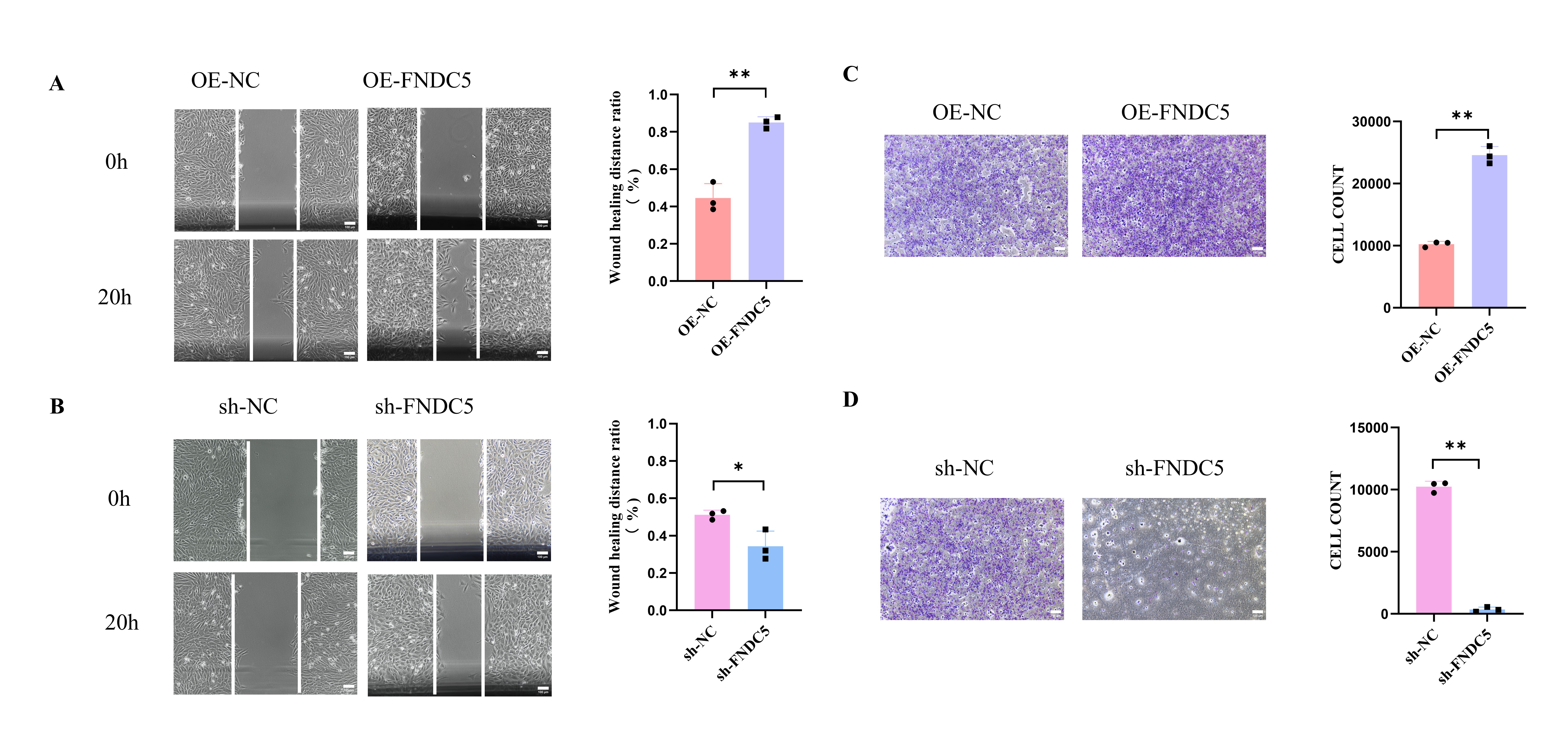

Supplement: Supplementary file 3 — Supplementary Material 3. [file 13395_2026_420_MOESM3_ESM.jpg]

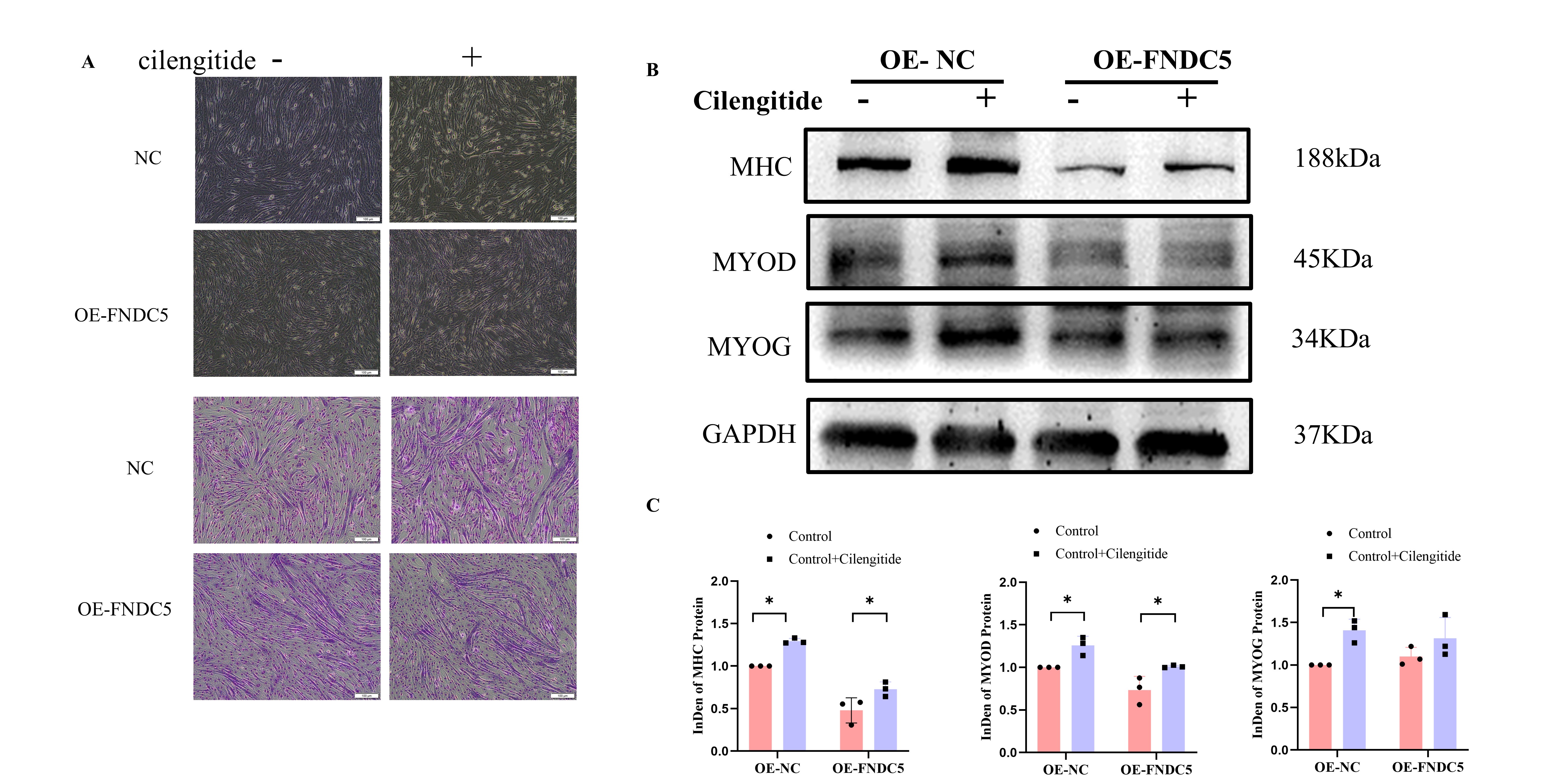

Supplement: Supplementary file 4 — Supplementary Material 4. [file 13395_2026_420_MOESM4_ESM.jpg]

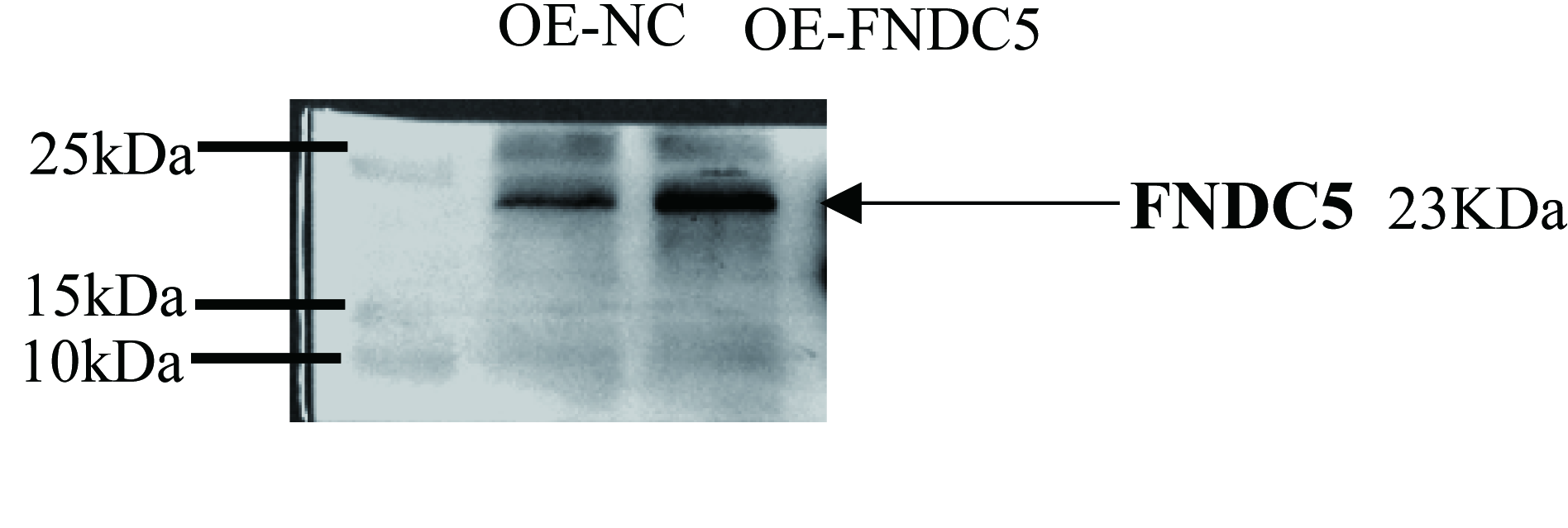

Supplement: Supplementary file 5 — Supplementary Material 5. [file 13395_2026_420_MOESM5_ESM.zip › Supplementary Material 5/Fig1/Fig1A/OE-FNDC5/FNDC5/FNDC5-1.tif]

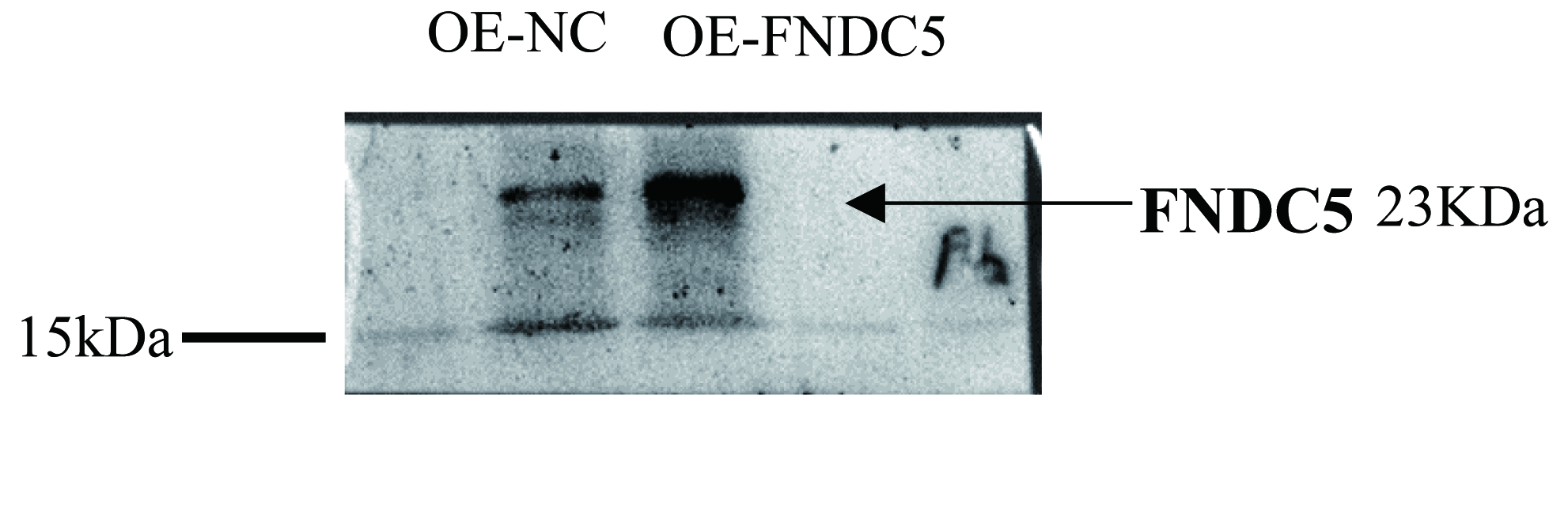

Supplement: Supplementary file 5 — Supplementary Material 5. [file 13395_2026_420_MOESM5_ESM.zip › Supplementary Material 5/Fig1/Fig1A/OE-FNDC5/FNDC5/FNDC5-2.tif]

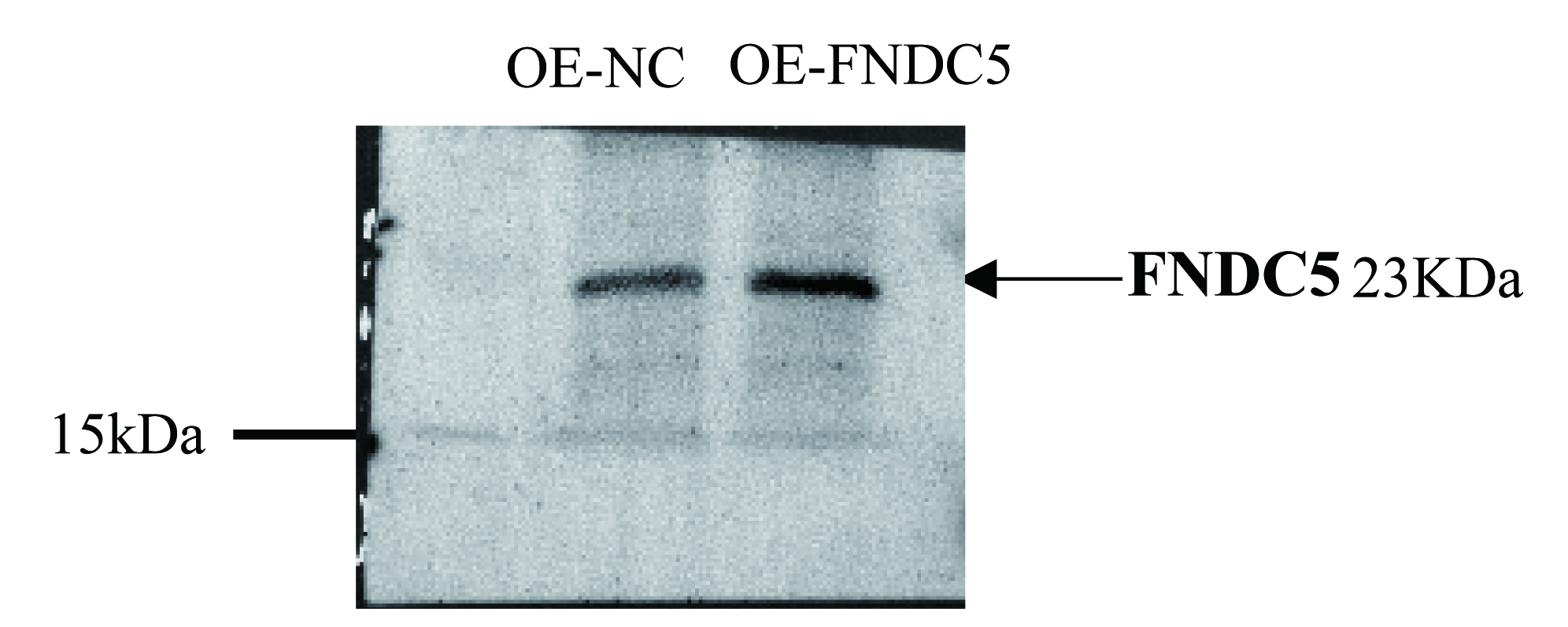

Supplement: Supplementary file 5 — Supplementary Material 5. [file 13395_2026_420_MOESM5_ESM.zip › Supplementary Material 5/Fig1/Fig1A/OE-FNDC5/FNDC5/FNDC5-3.tif]

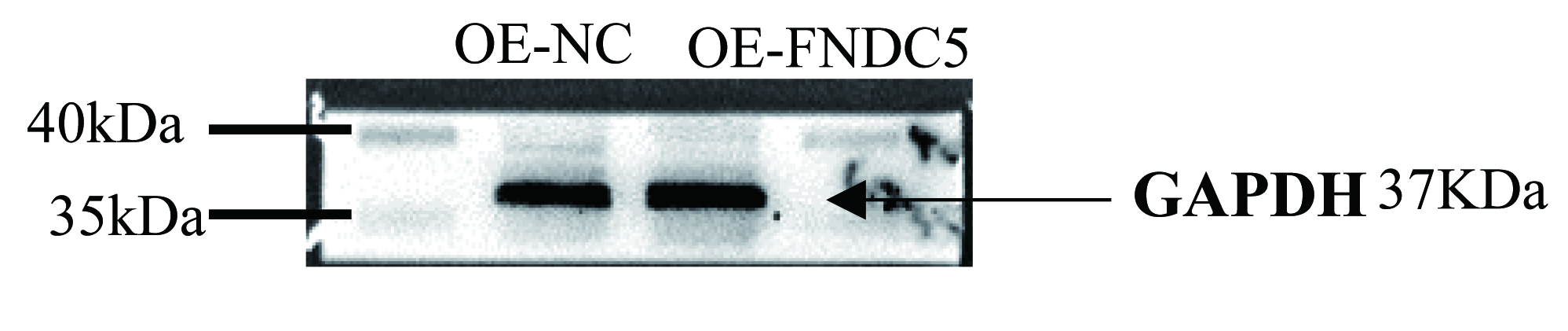

Supplement: Supplementary file 5 — Supplementary Material 5. [file 13395_2026_420_MOESM5_ESM.zip › Supplementary Material 5/Fig1/Fig1A/OE-FNDC5/GAPDH/GAPDH-1.tif]

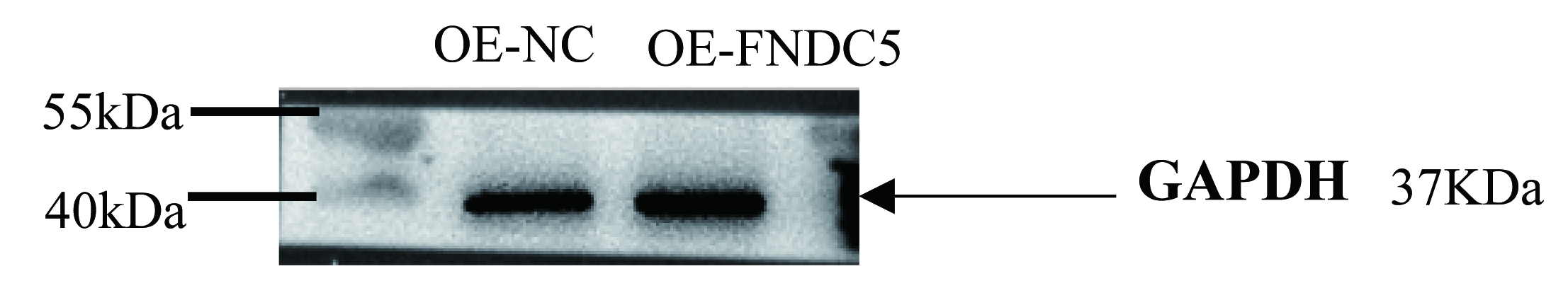

Supplement: Supplementary file 5 — Supplementary Material 5. [file 13395_2026_420_MOESM5_ESM.zip › Supplementary Material 5/Fig1/Fig1A/OE-FNDC5/GAPDH/GAPDH-2.tif]

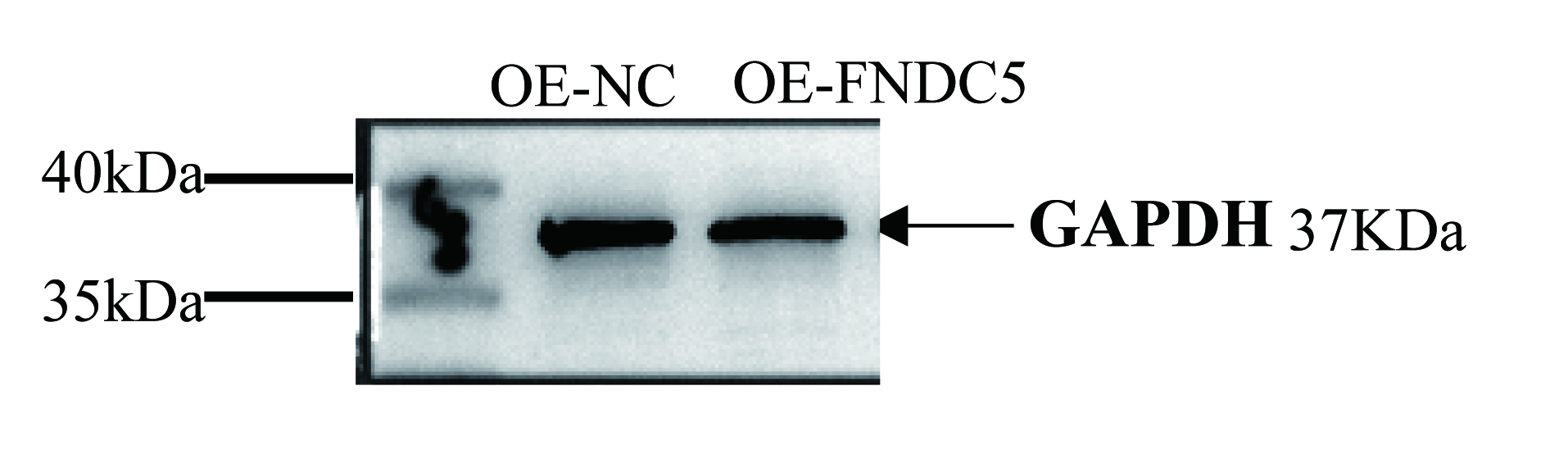

Supplement: Supplementary file 5 — Supplementary Material 5. [file 13395_2026_420_MOESM5_ESM.zip › Supplementary Material 5/Fig1/Fig1A/OE-FNDC5/GAPDH/GAPDH-3.tif]

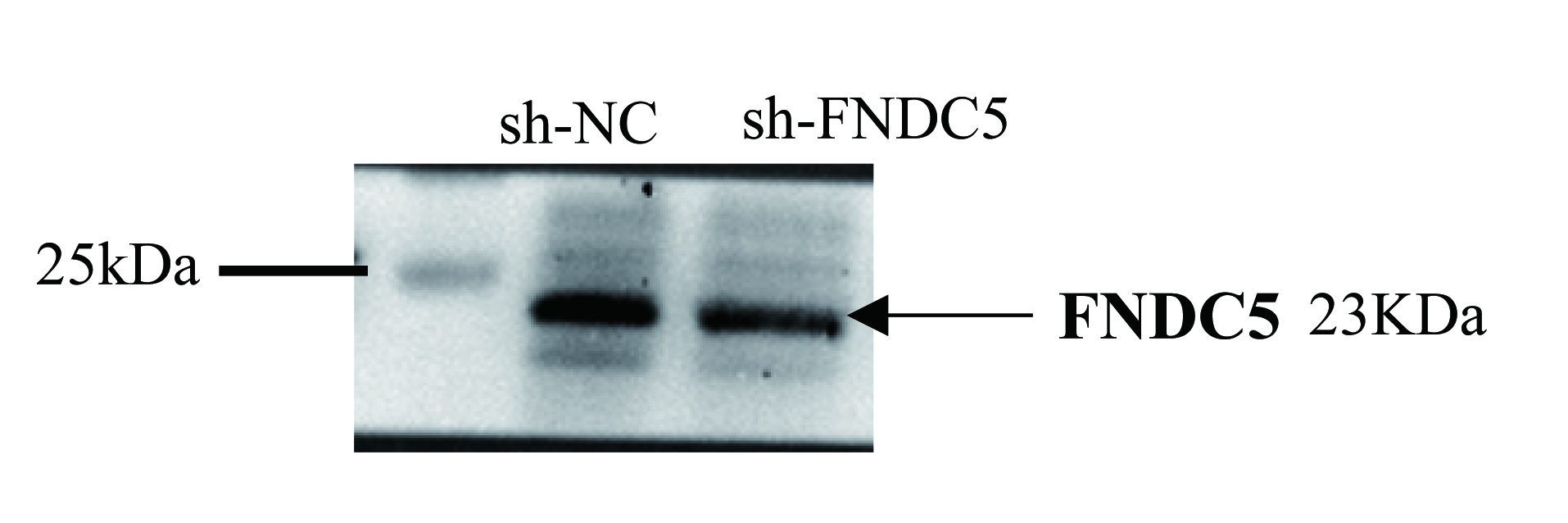

Supplement: Supplementary file 5 — Supplementary Material 5. [file 13395_2026_420_MOESM5_ESM.zip › Supplementary Material 5/Fig1/Fig1A/sh-FNDC5/FNDC5/FNDC5-1.tif]

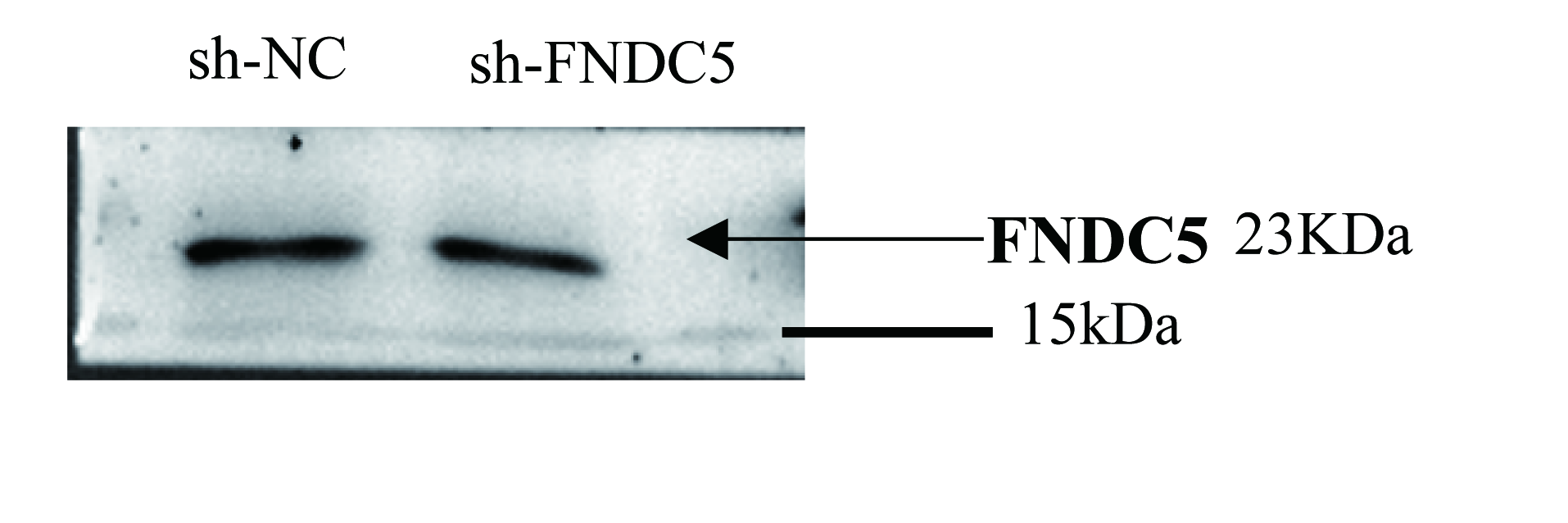

Supplement: Supplementary file 5 — Supplementary Material 5. [file 13395_2026_420_MOESM5_ESM.zip › Supplementary Material 5/Fig1/Fig1A/sh-FNDC5/FNDC5/FNDC5-2.tif]

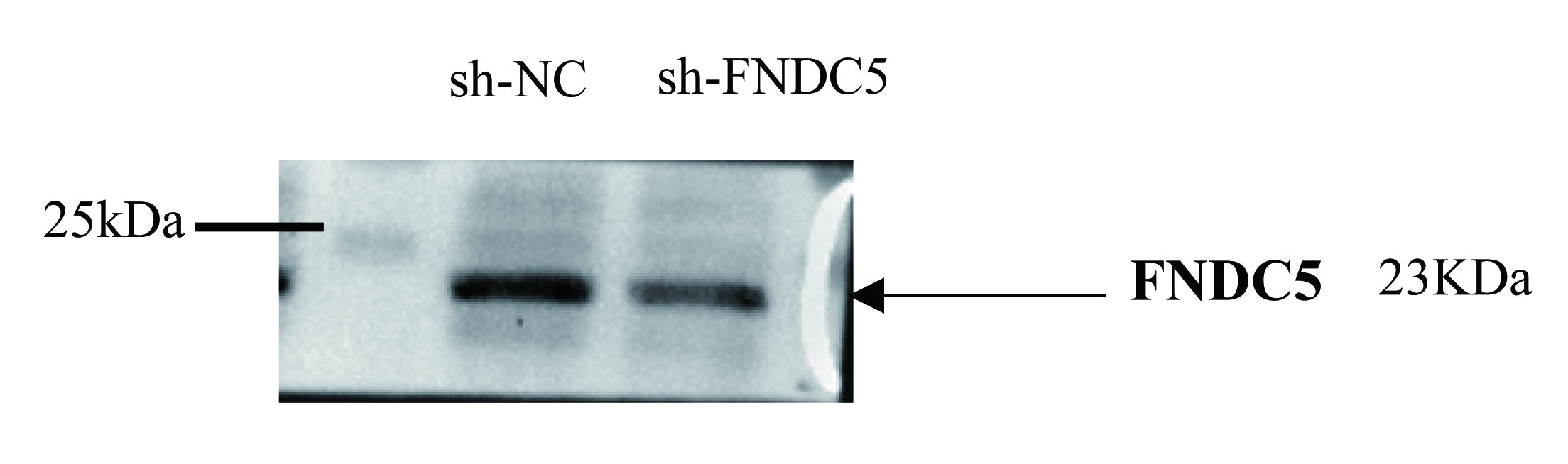

Supplement: Supplementary file 5 — Supplementary Material 5. [file 13395_2026_420_MOESM5_ESM.zip › Supplementary Material 5/Fig1/Fig1A/sh-FNDC5/FNDC5/FNDC5-3.tif]

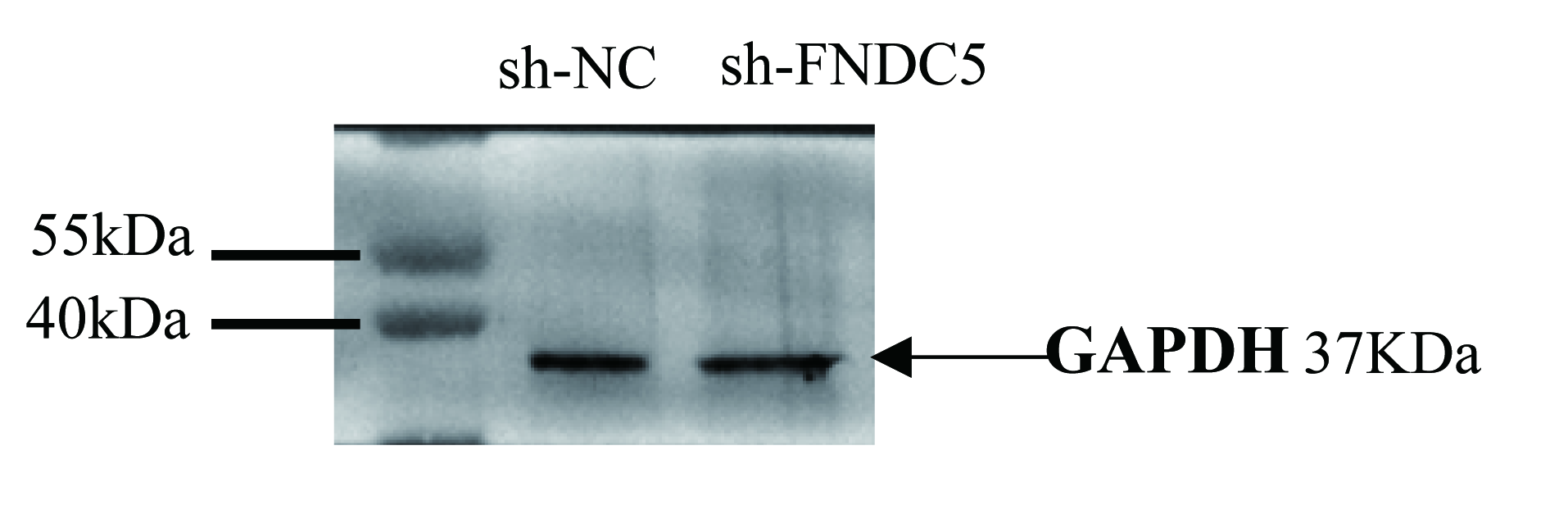

Supplement: Supplementary file 5 — Supplementary Material 5. [file 13395_2026_420_MOESM5_ESM.zip › Supplementary Material 5/Fig1/Fig1A/sh-FNDC5/GAPDH/GAPDH-1.tif]

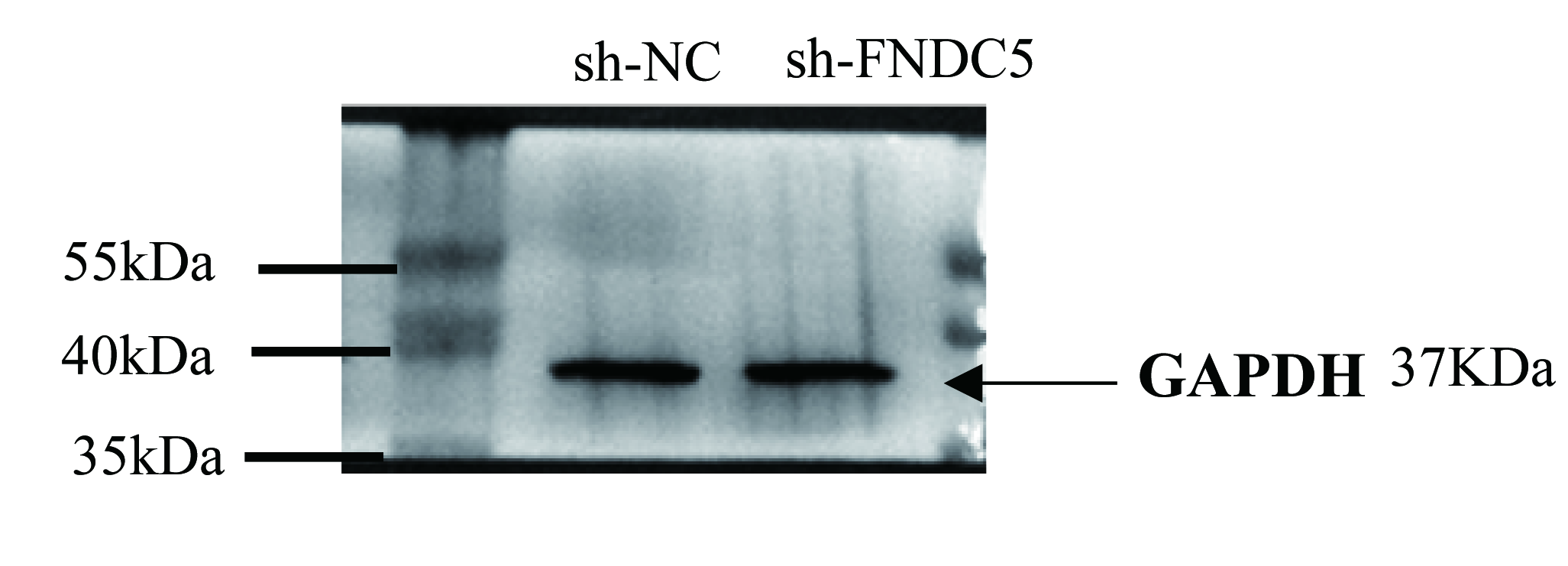

Supplement: Supplementary file 5 — Supplementary Material 5. [file 13395_2026_420_MOESM5_ESM.zip › Supplementary Material 5/Fig1/Fig1A/sh-FNDC5/GAPDH/GAPDH-2.tif]

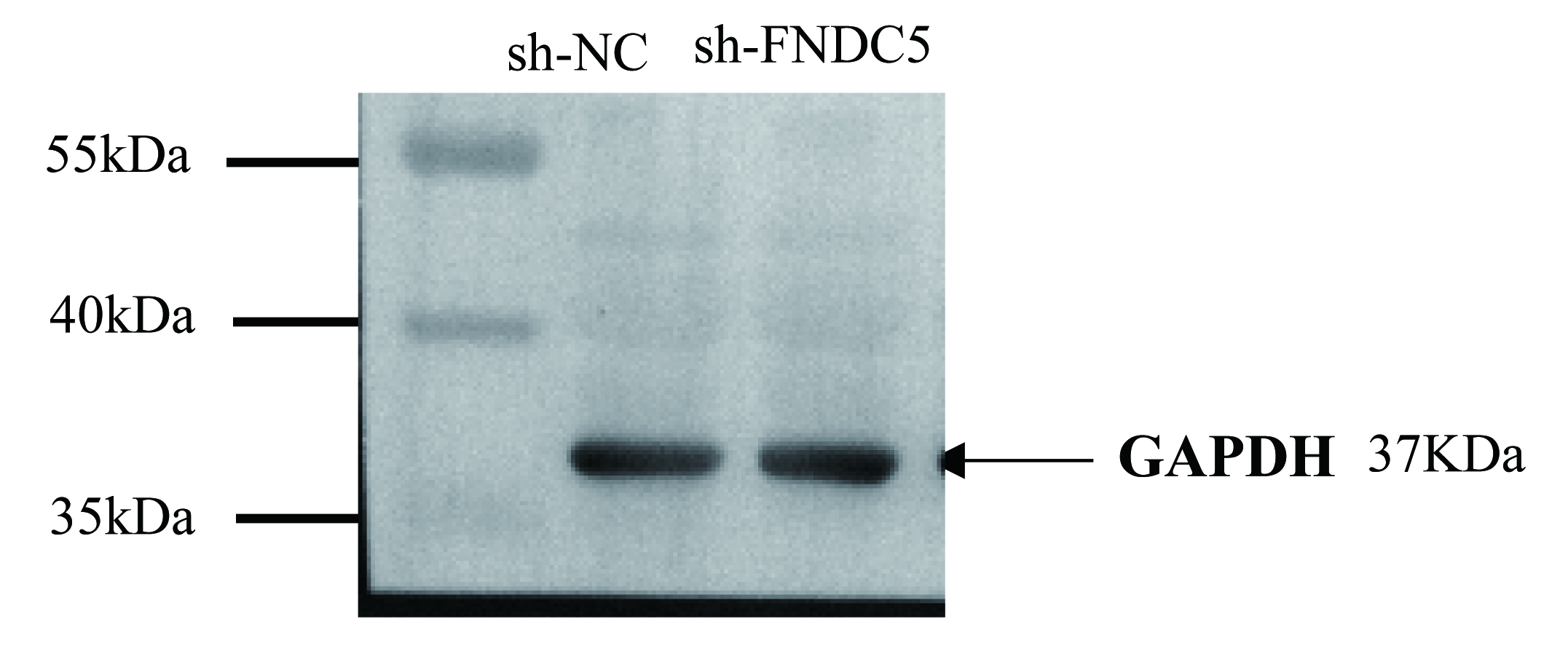

Supplement: Supplementary file 5 — Supplementary Material 5. [file 13395_2026_420_MOESM5_ESM.zip › Supplementary Material 5/Fig1/Fig1A/sh-FNDC5/GAPDH/GAPDH-3.tif]

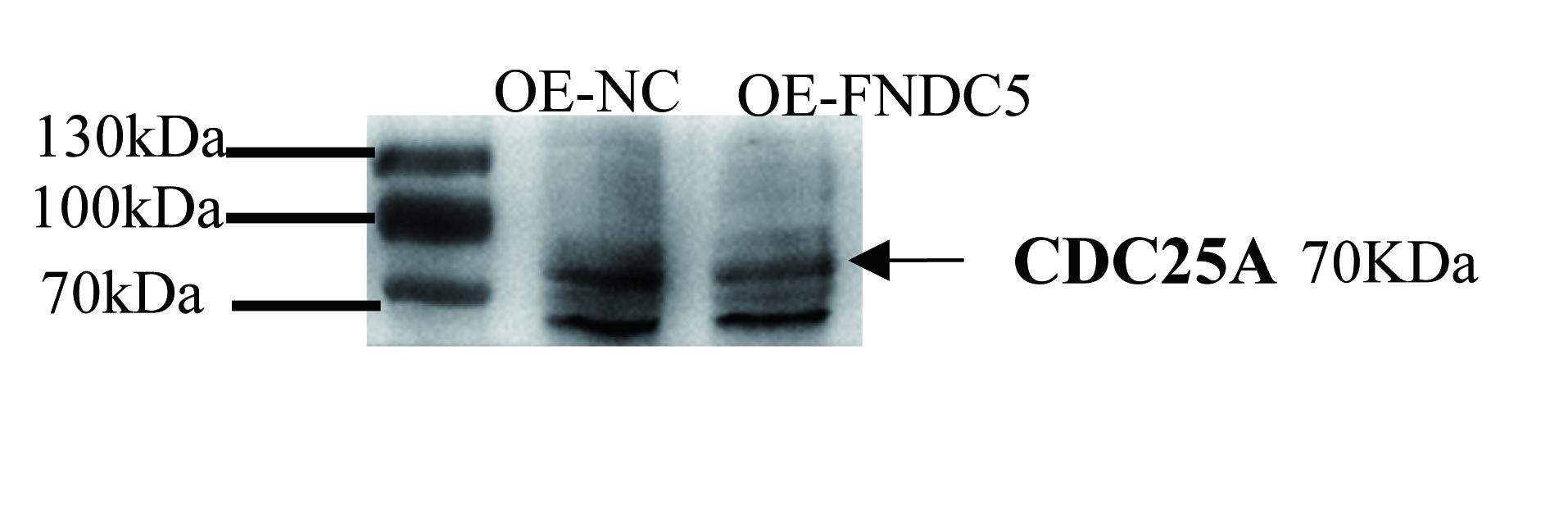

Supplement: Supplementary file 5 — Supplementary Material 5. [file 13395_2026_420_MOESM5_ESM.zip › Supplementary Material 5/Fig1/Fig1I/CDC25A/CDC25A-1.tif]

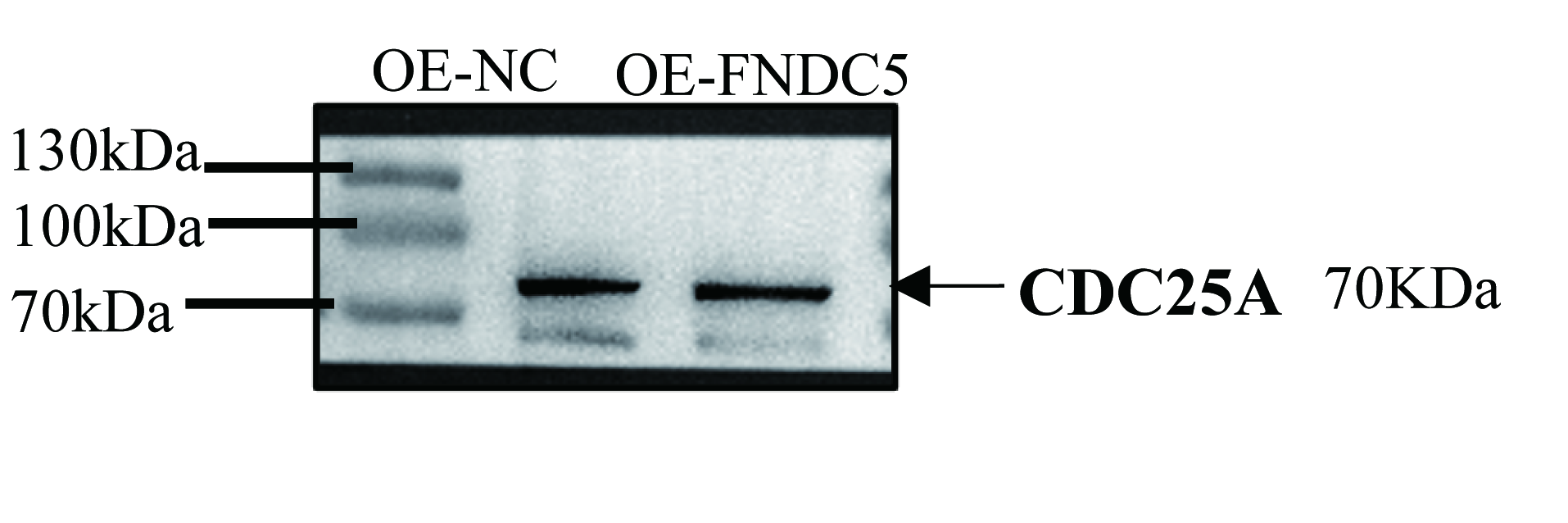

Supplement: Supplementary file 5 — Supplementary Material 5. [file 13395_2026_420_MOESM5_ESM.zip › Supplementary Material 5/Fig1/Fig1I/CDC25A/CDC25A-2.tif]

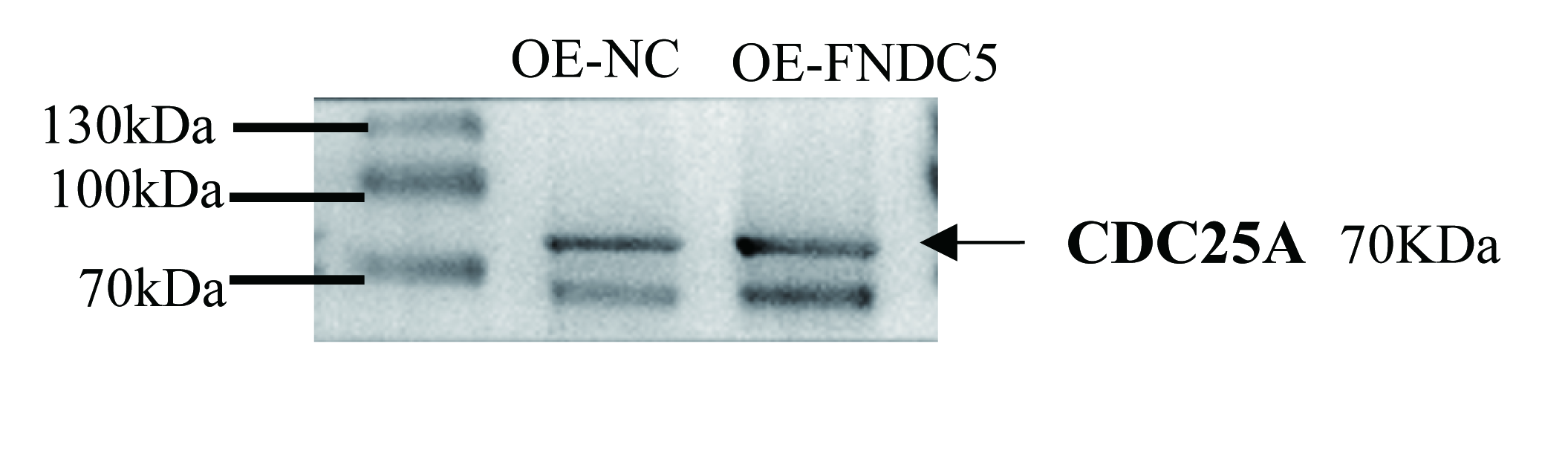

Supplement: Supplementary file 5 — Supplementary Material 5. [file 13395_2026_420_MOESM5_ESM.zip › Supplementary Material 5/Fig1/Fig1I/CDC25A/CDC25A-3.tif]

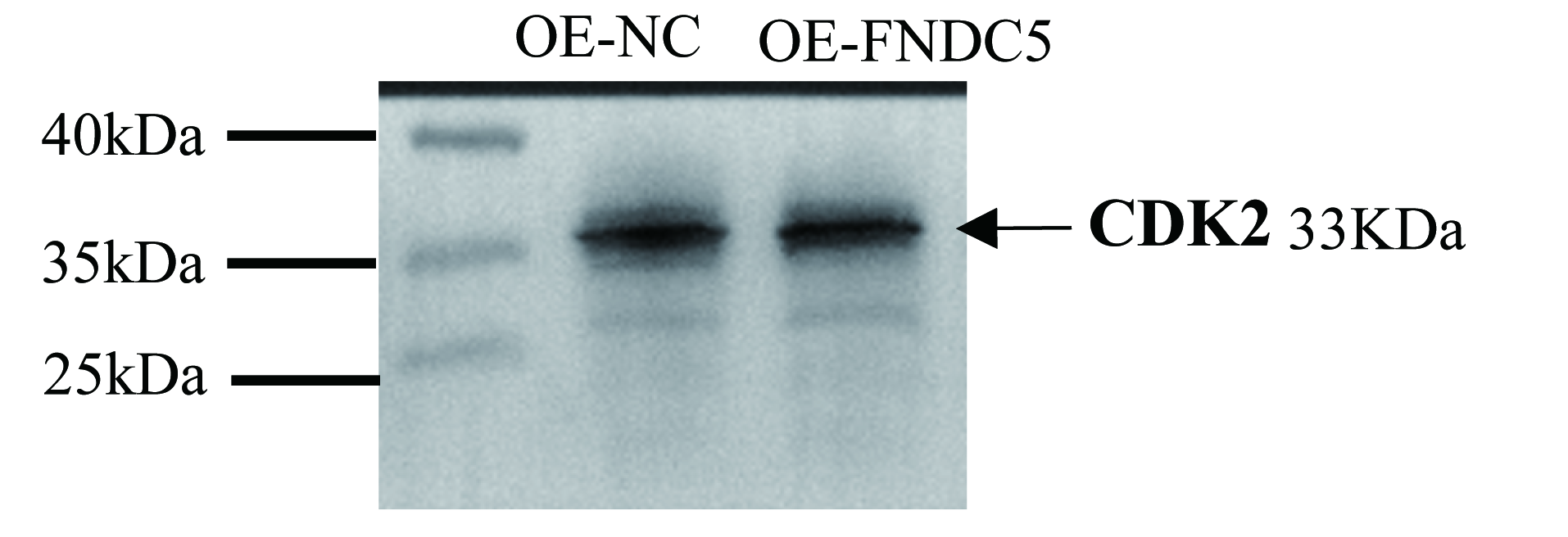

Supplement: Supplementary file 5 — Supplementary Material 5. [file 13395_2026_420_MOESM5_ESM.zip › Supplementary Material 5/Fig1/Fig1I/CDK2/CDK2-1.tif]

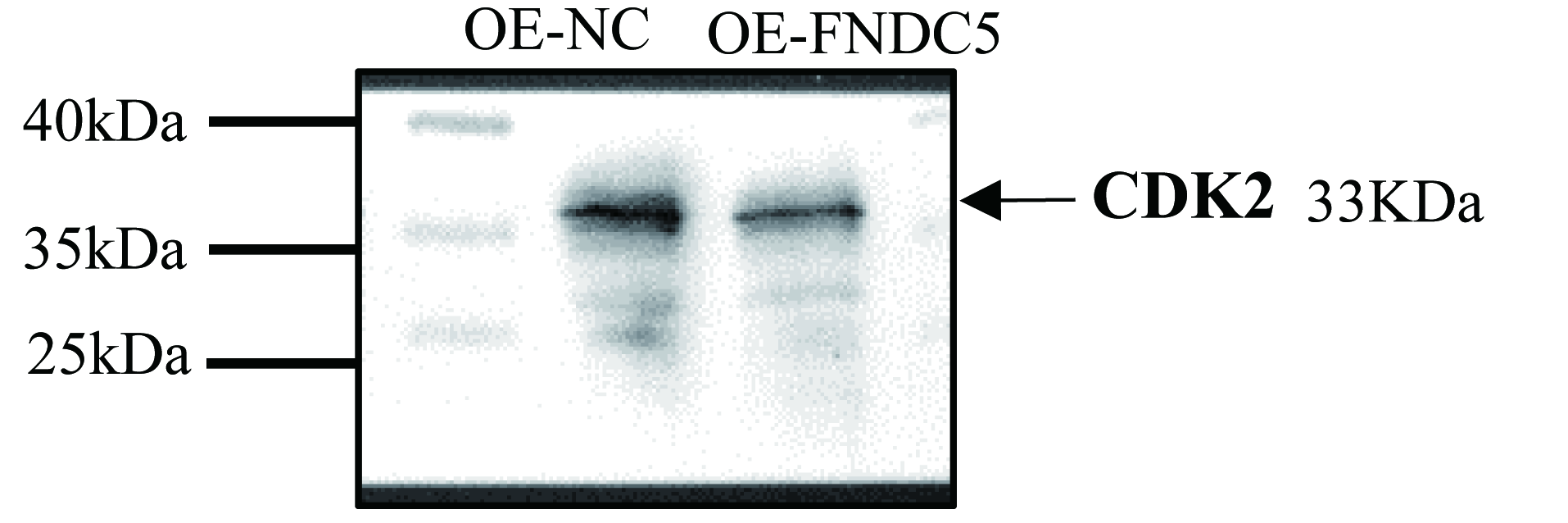

Supplement: Supplementary file 5 — Supplementary Material 5. [file 13395_2026_420_MOESM5_ESM.zip › Supplementary Material 5/Fig1/Fig1I/CDK2/CDK2-2.tif]

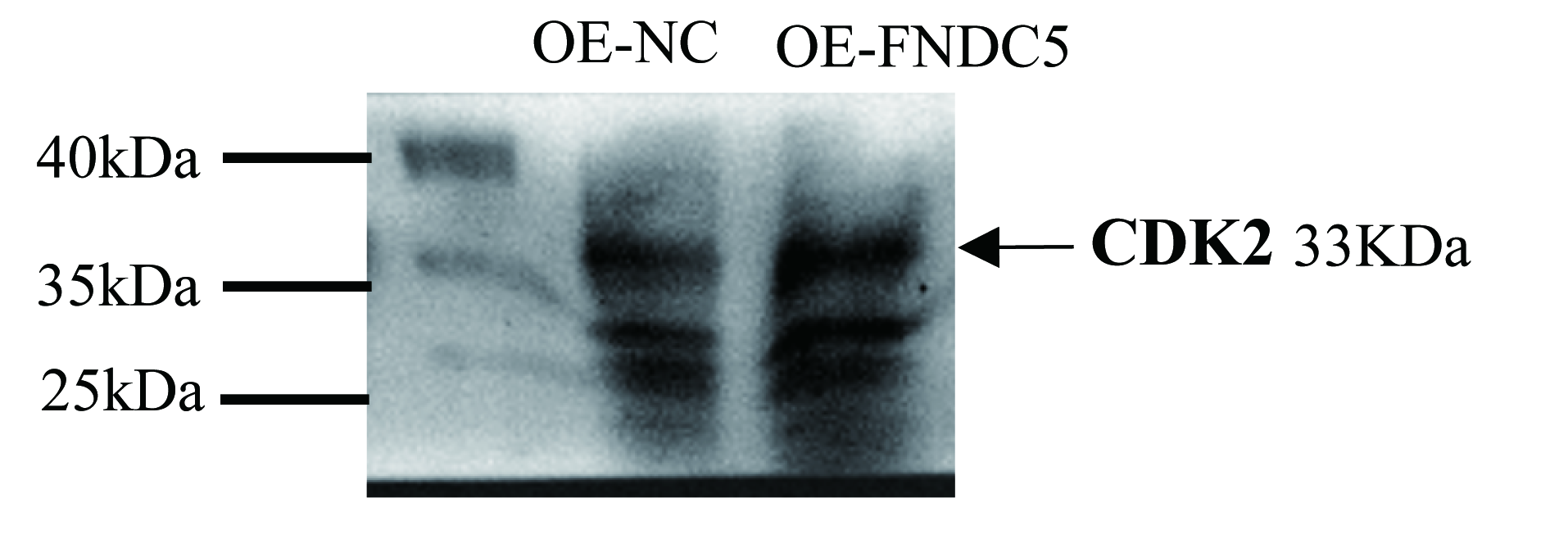

Supplement: Supplementary file 5 — Supplementary Material 5. [file 13395_2026_420_MOESM5_ESM.zip › Supplementary Material 5/Fig1/Fig1I/CDK2/CDK2-3.tif]

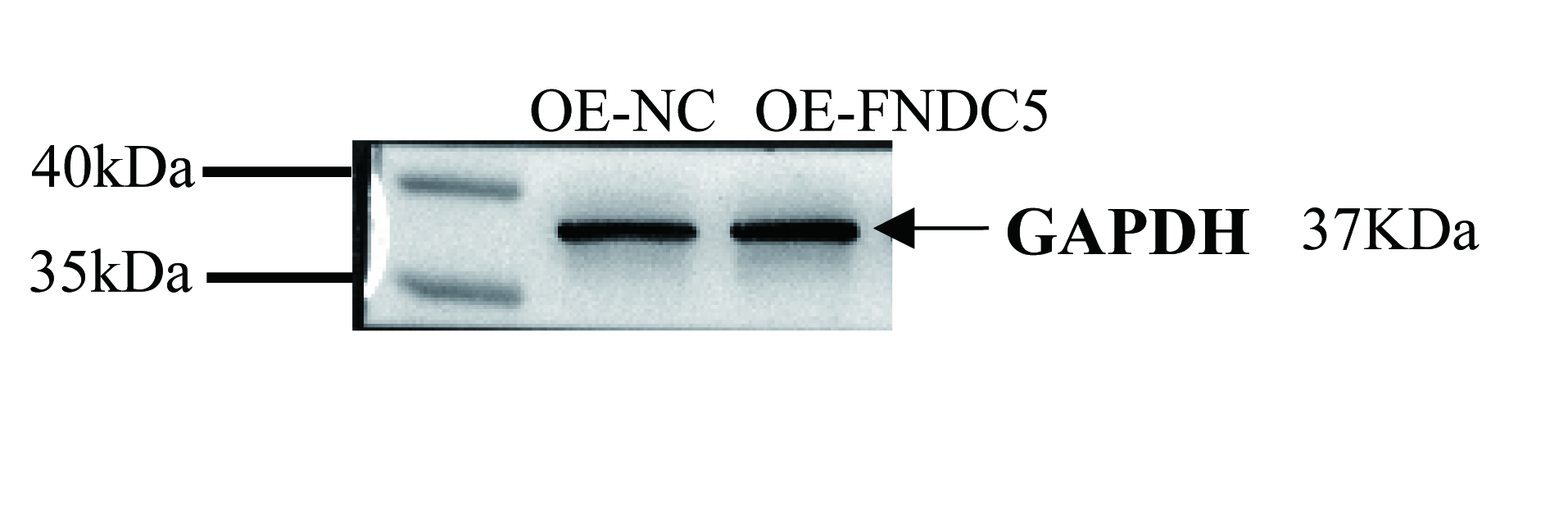

Supplement: Supplementary file 5 — Supplementary Material 5. [file 13395_2026_420_MOESM5_ESM.zip › Supplementary Material 5/Fig1/Fig1I/GAPDH/GAPDH-1.tif]

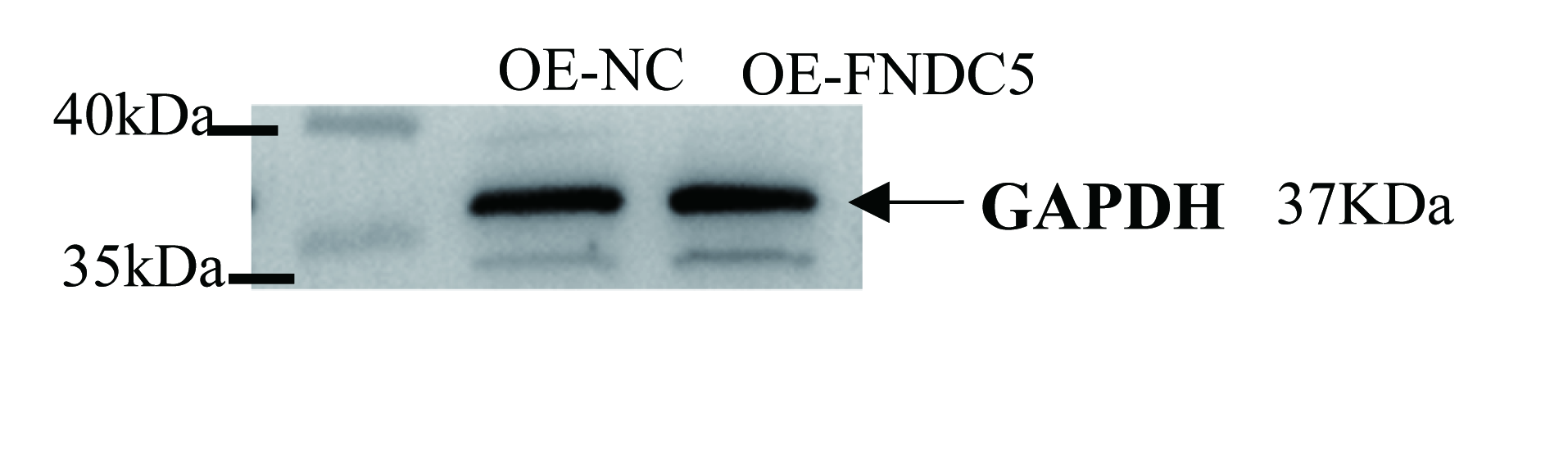

Supplement: Supplementary file 5 — Supplementary Material 5. [file 13395_2026_420_MOESM5_ESM.zip › Supplementary Material 5/Fig1/Fig1I/GAPDH/GAPDH-2.tif]

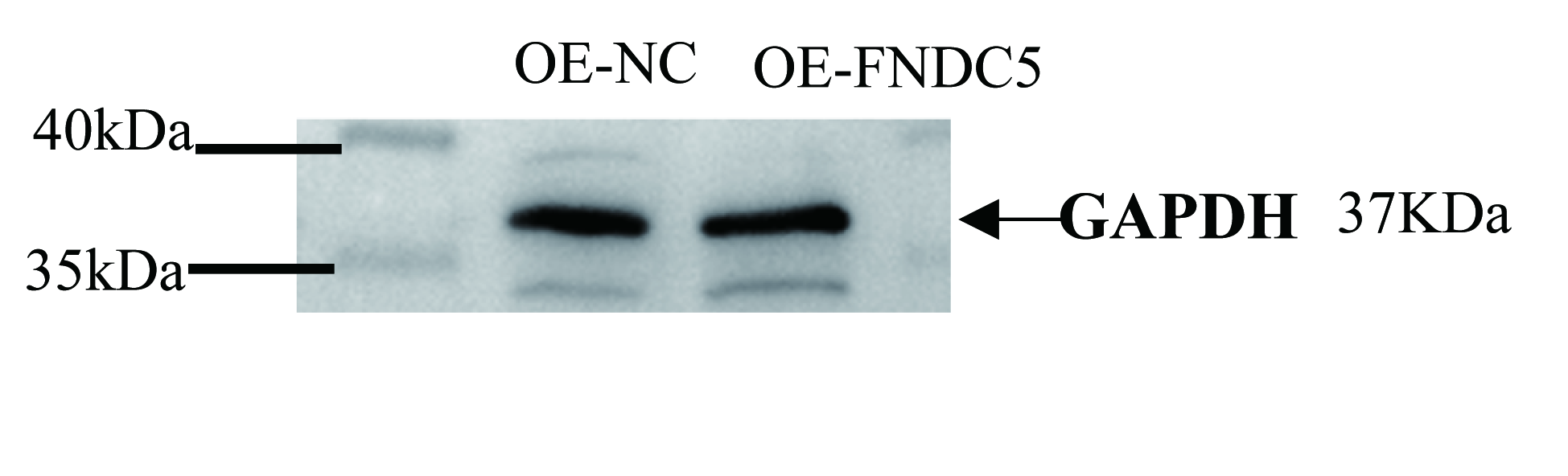

Supplement: Supplementary file 5 — Supplementary Material 5. [file 13395_2026_420_MOESM5_ESM.zip › Supplementary Material 5/Fig1/Fig1I/GAPDH/GAPDH-3.tif]

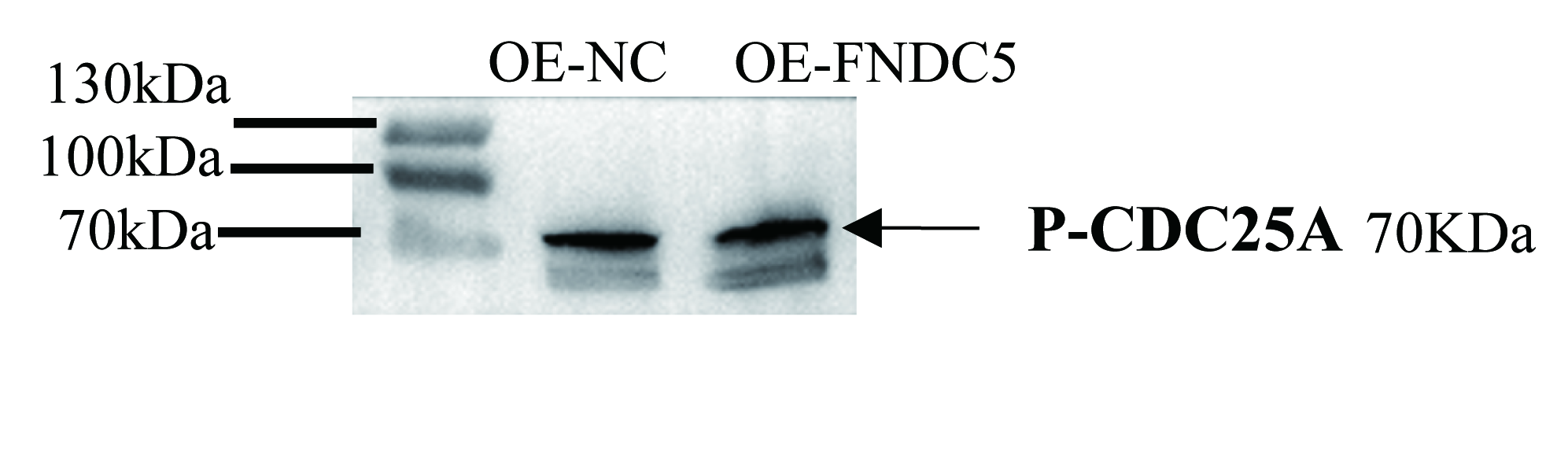

Supplement: Supplementary file 5 — Supplementary Material 5. [file 13395_2026_420_MOESM5_ESM.zip › Supplementary Material 5/Fig1/Fig1I/P-CDC25A/P-CDC25A-1.tif]

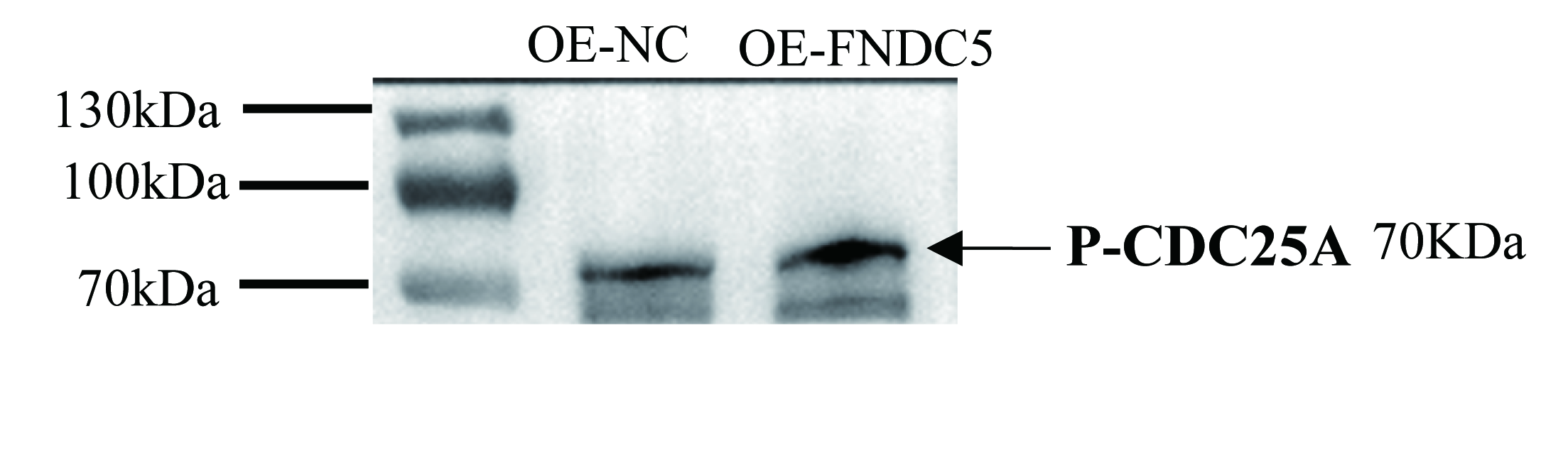

Supplement: Supplementary file 5 — Supplementary Material 5. [file 13395_2026_420_MOESM5_ESM.zip › Supplementary Material 5/Fig1/Fig1I/P-CDC25A/P-CDC25A-2.tif]

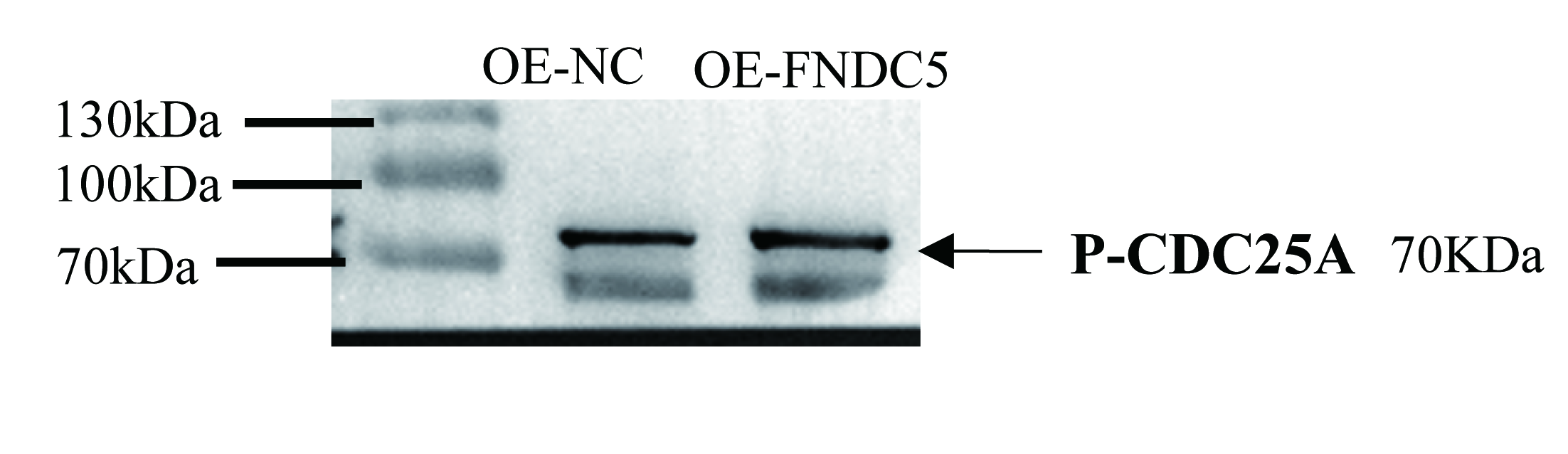

Supplement: Supplementary file 5 — Supplementary Material 5. [file 13395_2026_420_MOESM5_ESM.zip › Supplementary Material 5/Fig1/Fig1I/P-CDC25A/P-CDC25A-3.tif]

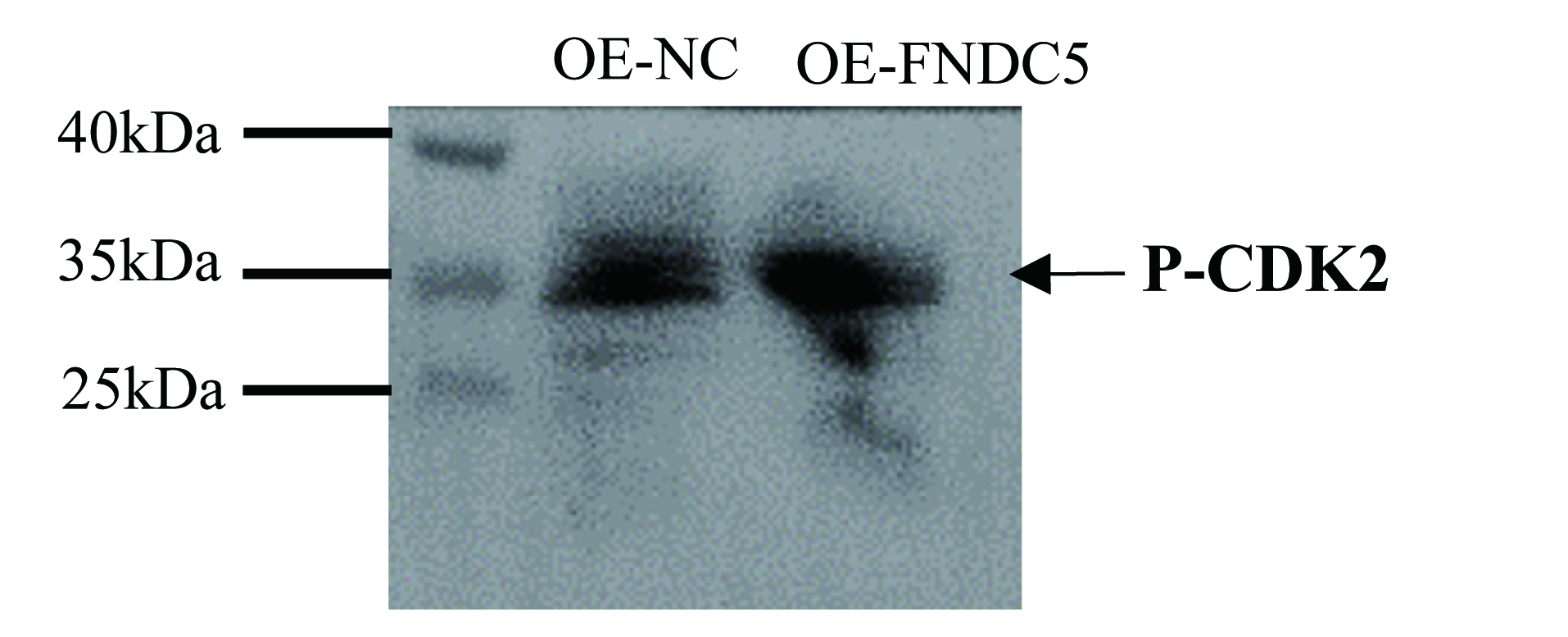

Supplement: Supplementary file 5 — Supplementary Material 5. [file 13395_2026_420_MOESM5_ESM.zip › Supplementary Material 5/Fig1/Fig1I/P-CDK2/PCDK2-1.tif]

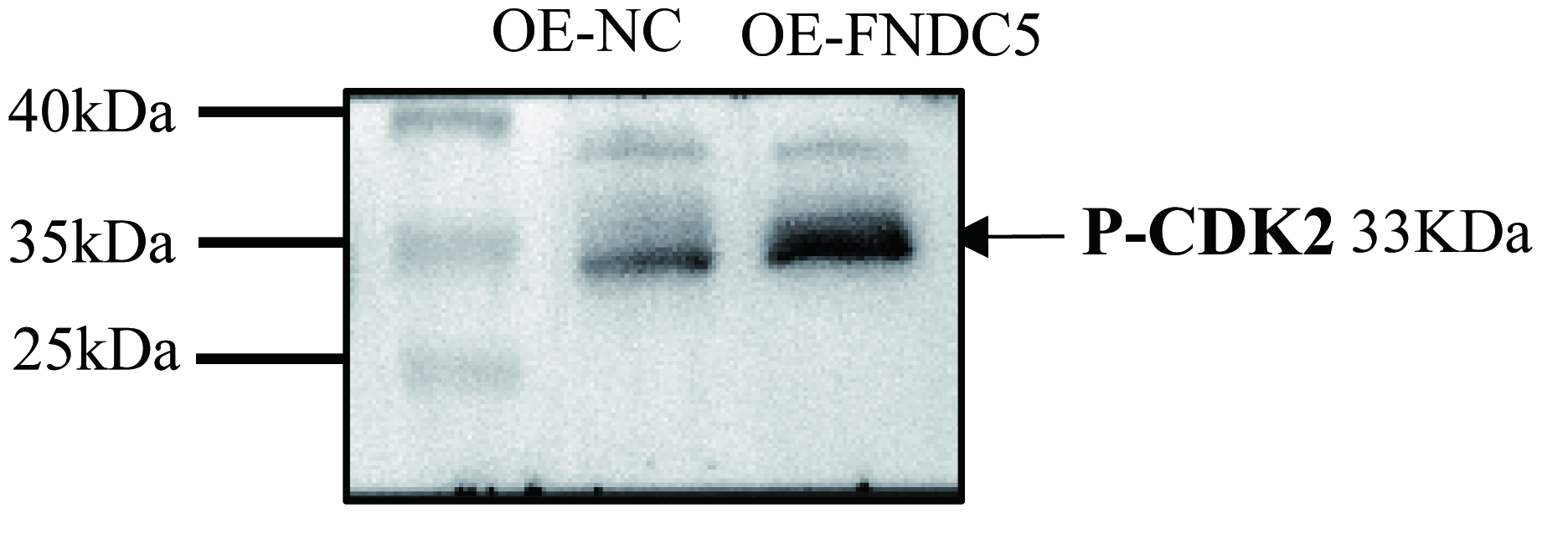

Supplement: Supplementary file 5 — Supplementary Material 5. [file 13395_2026_420_MOESM5_ESM.zip › Supplementary Material 5/Fig1/Fig1I/P-CDK2/PCDK2-2.tif]

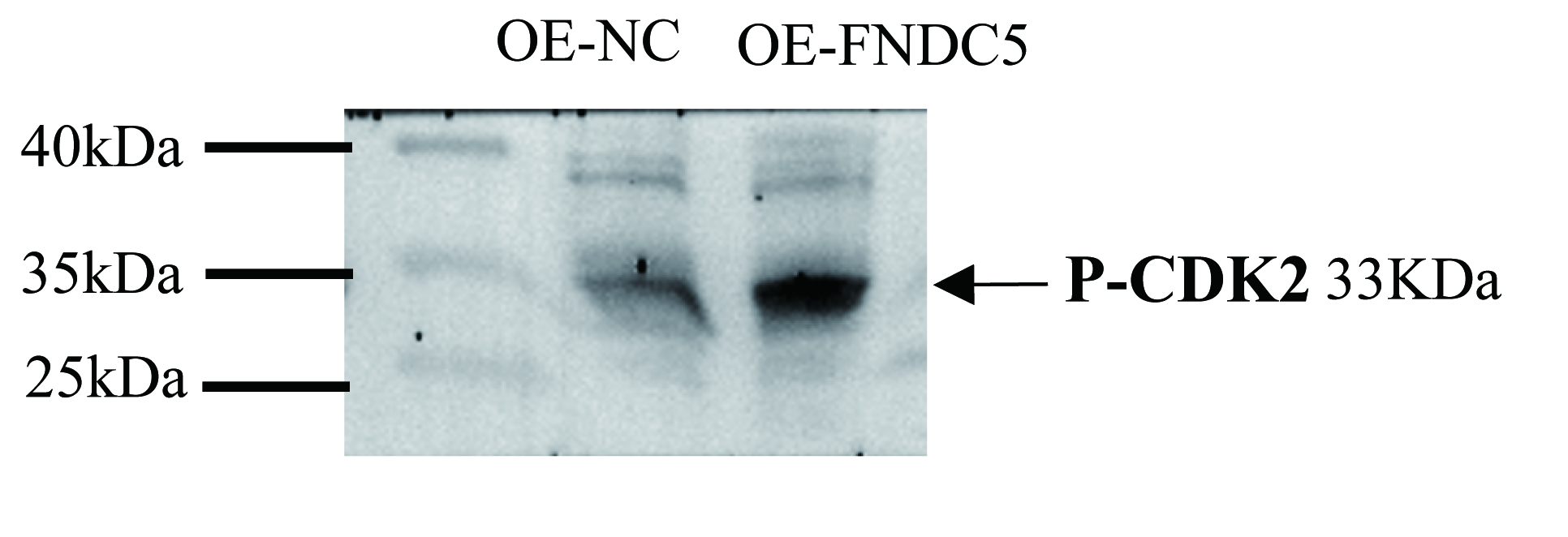

Supplement: Supplementary file 5 — Supplementary Material 5. [file 13395_2026_420_MOESM5_ESM.zip › Supplementary Material 5/Fig1/Fig1I/P-CDK2/PCDK2-3.tif]

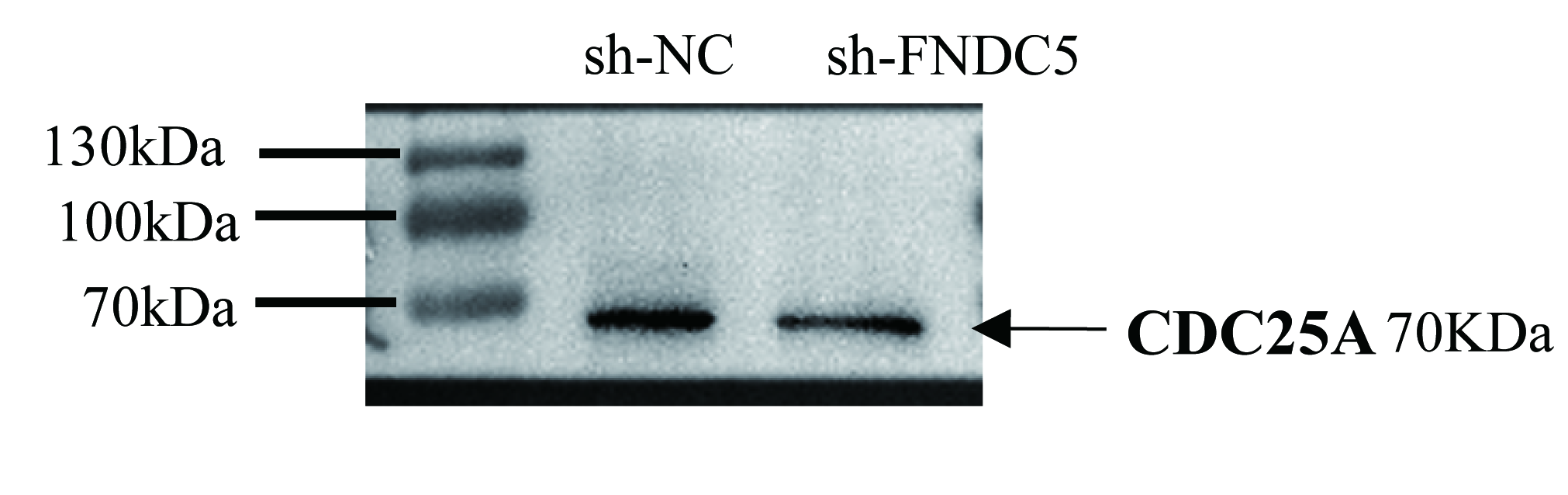

Supplement: Supplementary file 5 — Supplementary Material 5. [file 13395_2026_420_MOESM5_ESM.zip › Supplementary Material 5/Fig1/Fig1J/CDC25A/CDC25A-1.tif]

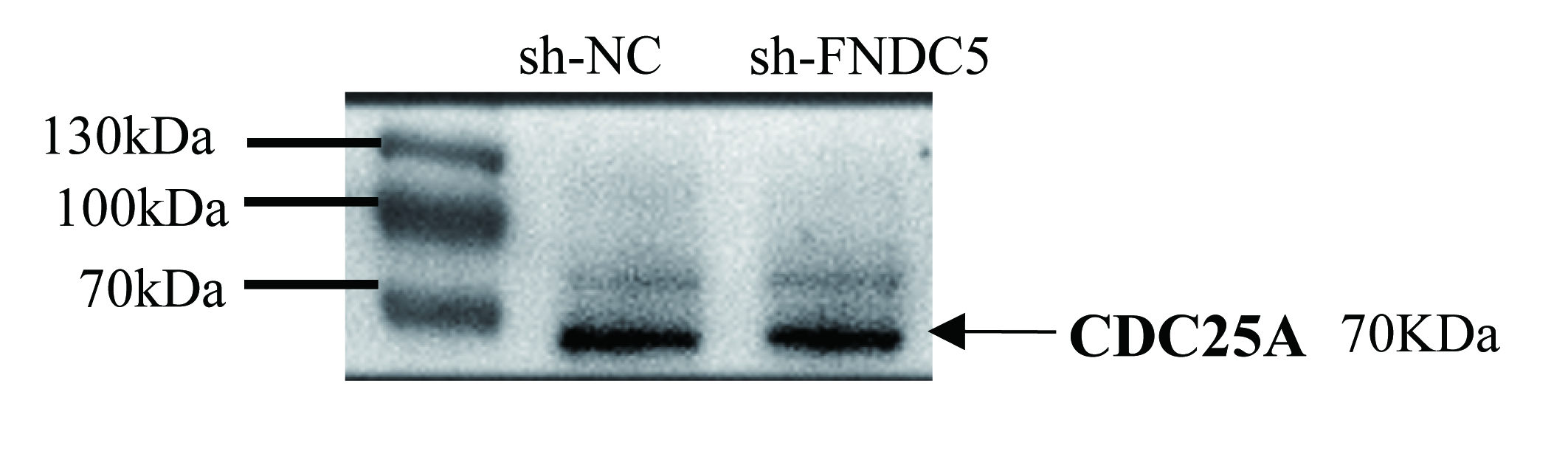

Supplement: Supplementary file 5 — Supplementary Material 5. [file 13395_2026_420_MOESM5_ESM.zip › Supplementary Material 5/Fig1/Fig1J/CDC25A/CDC25A-2.tif]

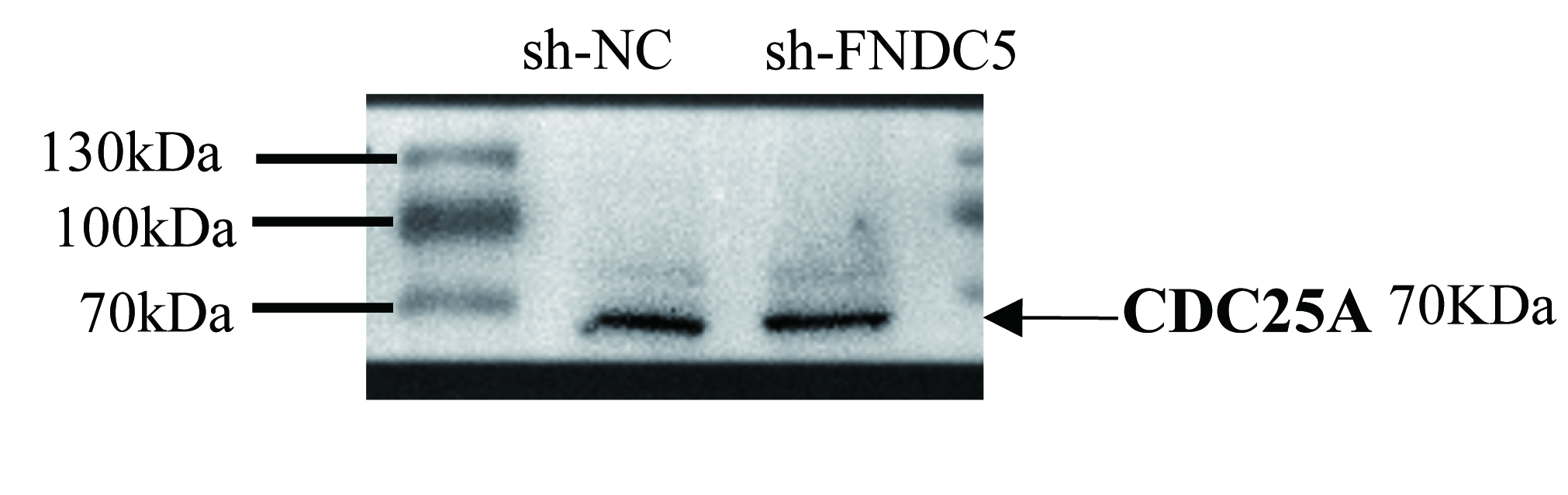

Supplement: Supplementary file 5 — Supplementary Material 5. [file 13395_2026_420_MOESM5_ESM.zip › Supplementary Material 5/Fig1/Fig1J/CDC25A/CDC25A-3.tif]

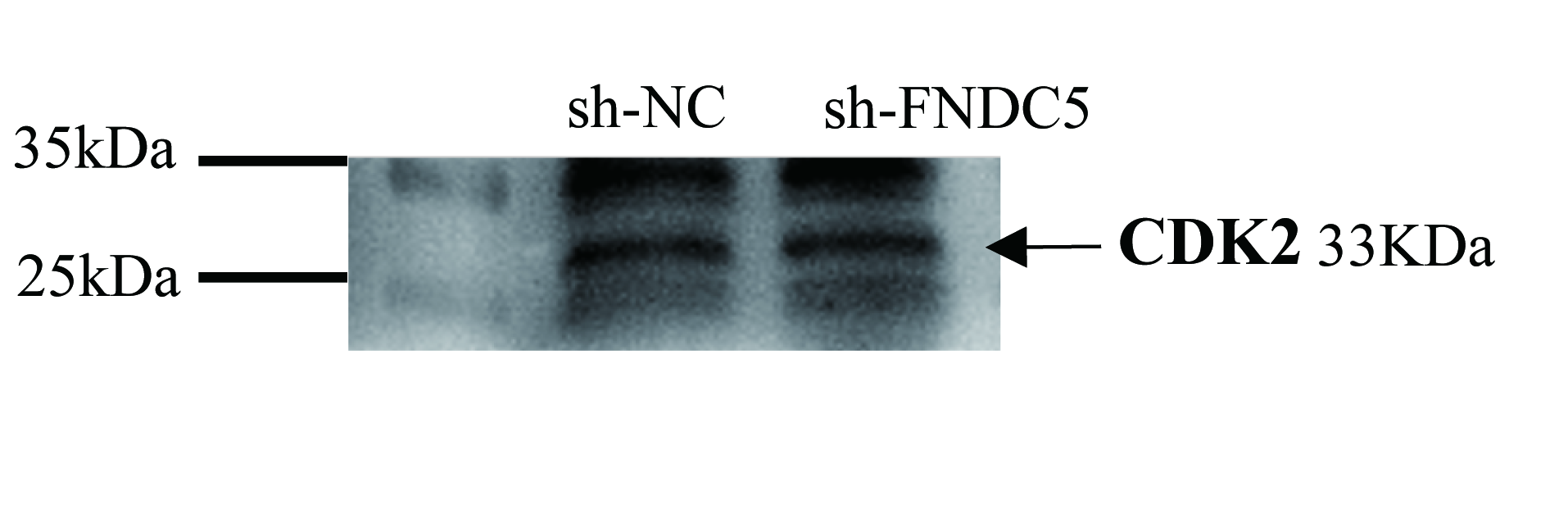

Supplement: Supplementary file 5 — Supplementary Material 5. [file 13395_2026_420_MOESM5_ESM.zip › Supplementary Material 5/Fig1/Fig1J/CDK2/CDK2-1.tif]

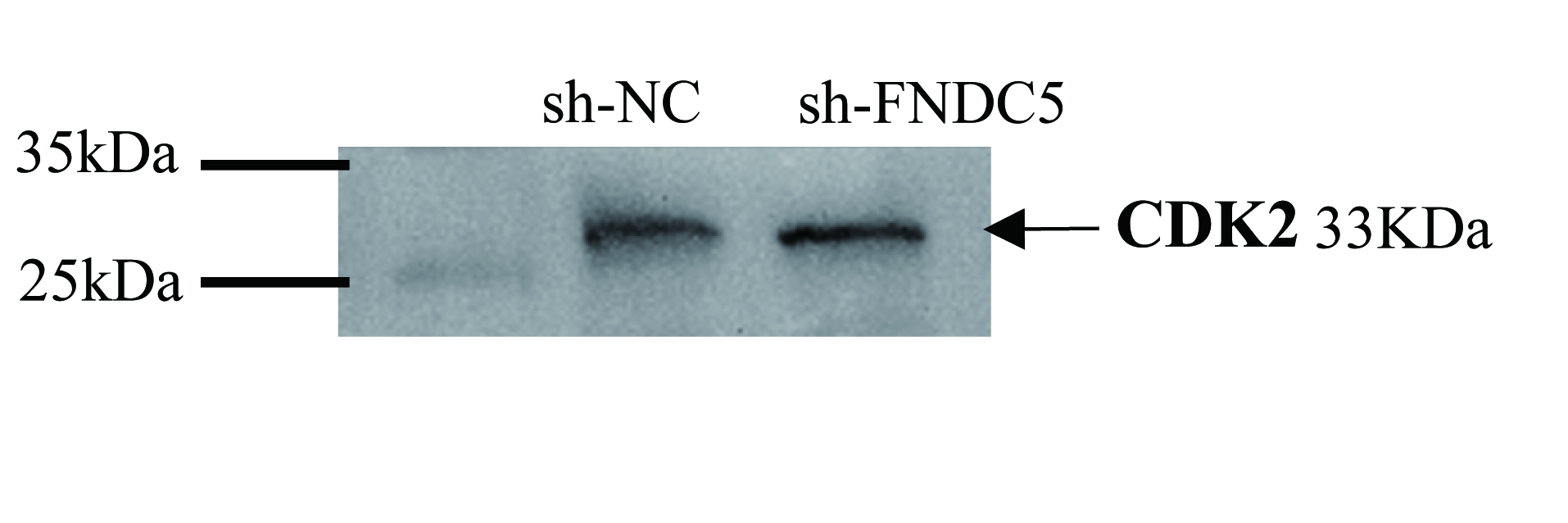

Supplement: Supplementary file 5 — Supplementary Material 5. [file 13395_2026_420_MOESM5_ESM.zip › Supplementary Material 5/Fig1/Fig1J/CDK2/CDK2-2.tif]

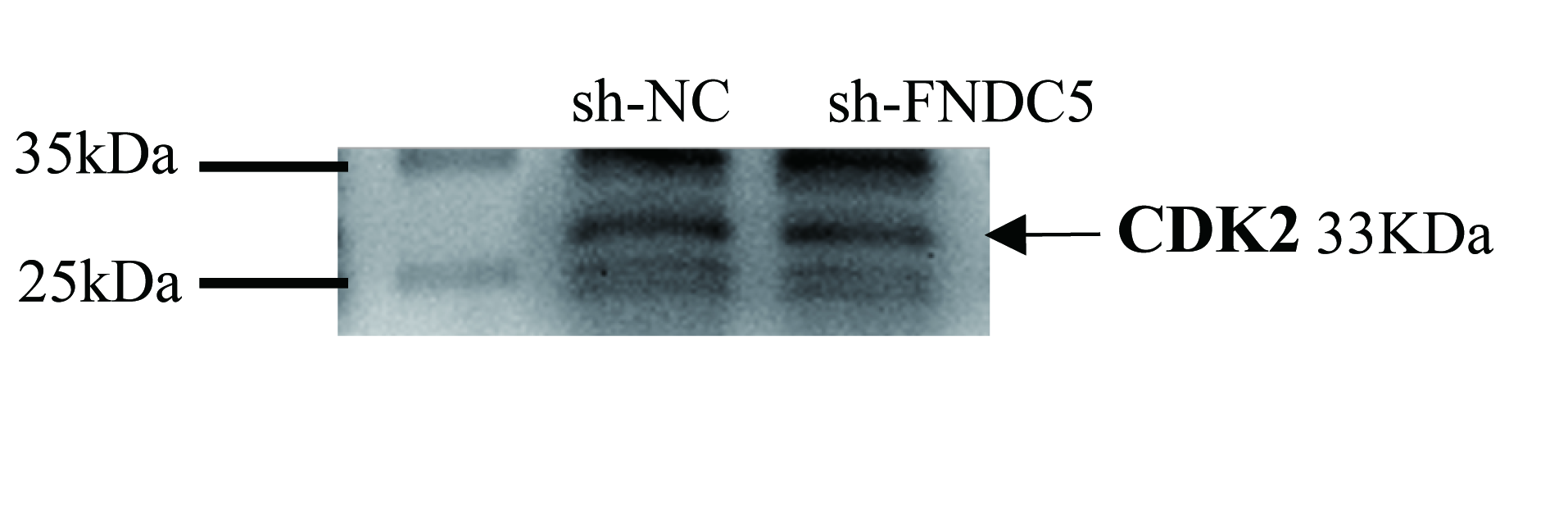

Supplement: Supplementary file 5 — Supplementary Material 5. [file 13395_2026_420_MOESM5_ESM.zip › Supplementary Material 5/Fig1/Fig1J/CDK2/CDK2-3.tif]

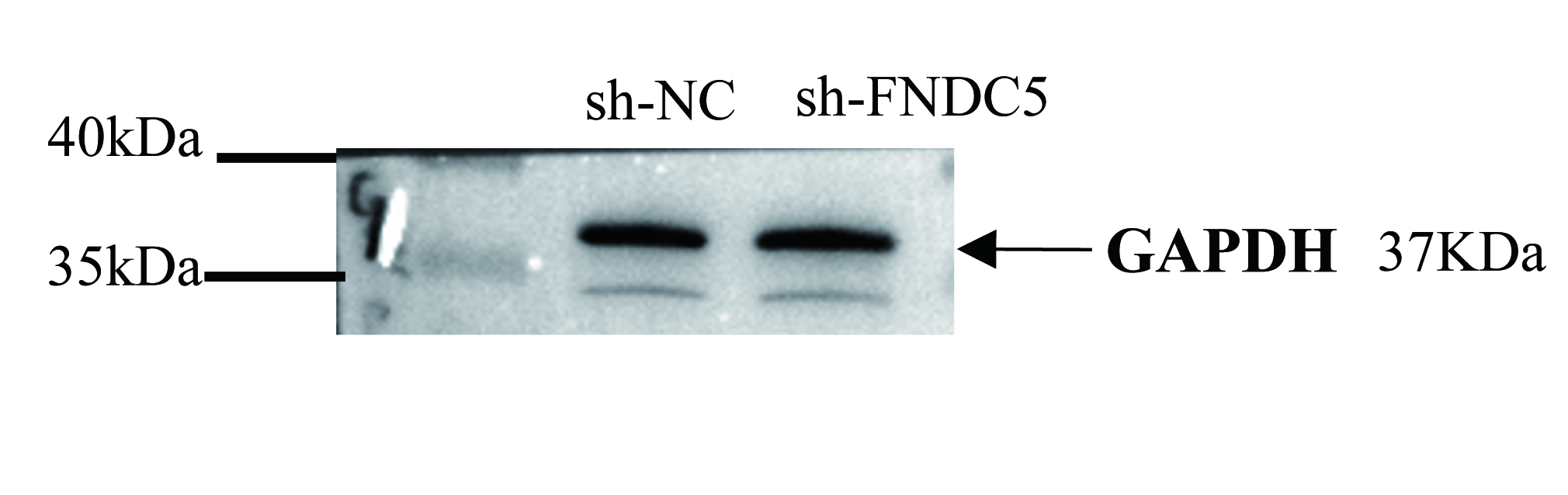

Supplement: Supplementary file 5 — Supplementary Material 5. [file 13395_2026_420_MOESM5_ESM.zip › Supplementary Material 5/Fig1/Fig1J/GAPDH/GAPDH-1.tif]

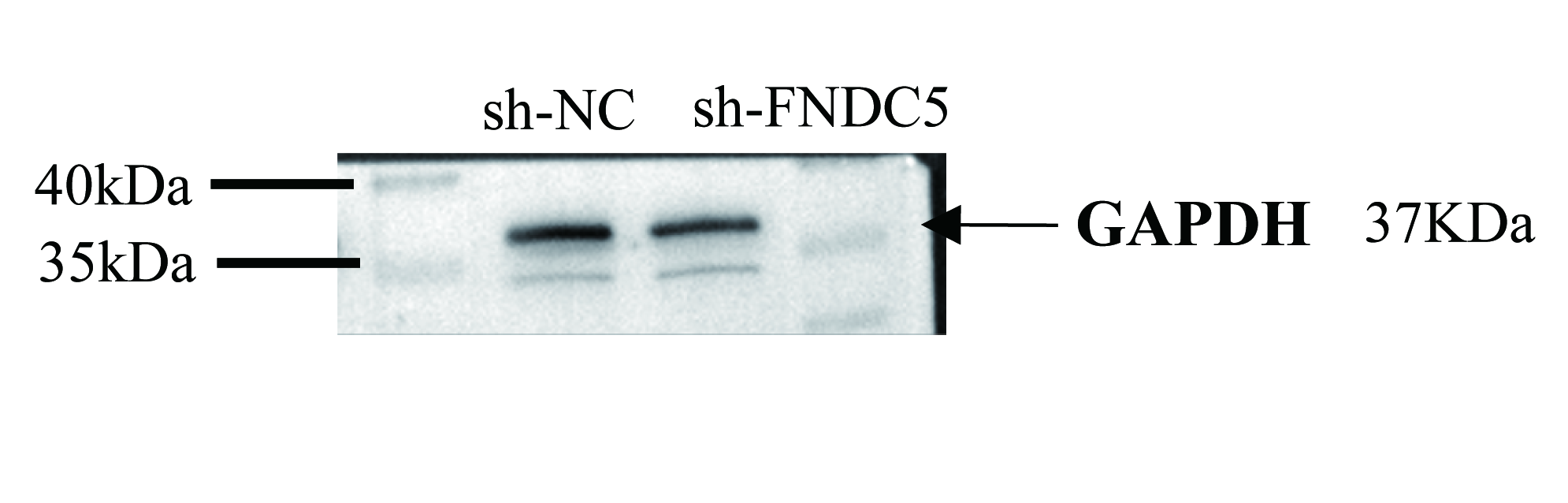

Supplement: Supplementary file 5 — Supplementary Material 5. [file 13395_2026_420_MOESM5_ESM.zip › Supplementary Material 5/Fig1/Fig1J/GAPDH/GAPDH-2.tif]

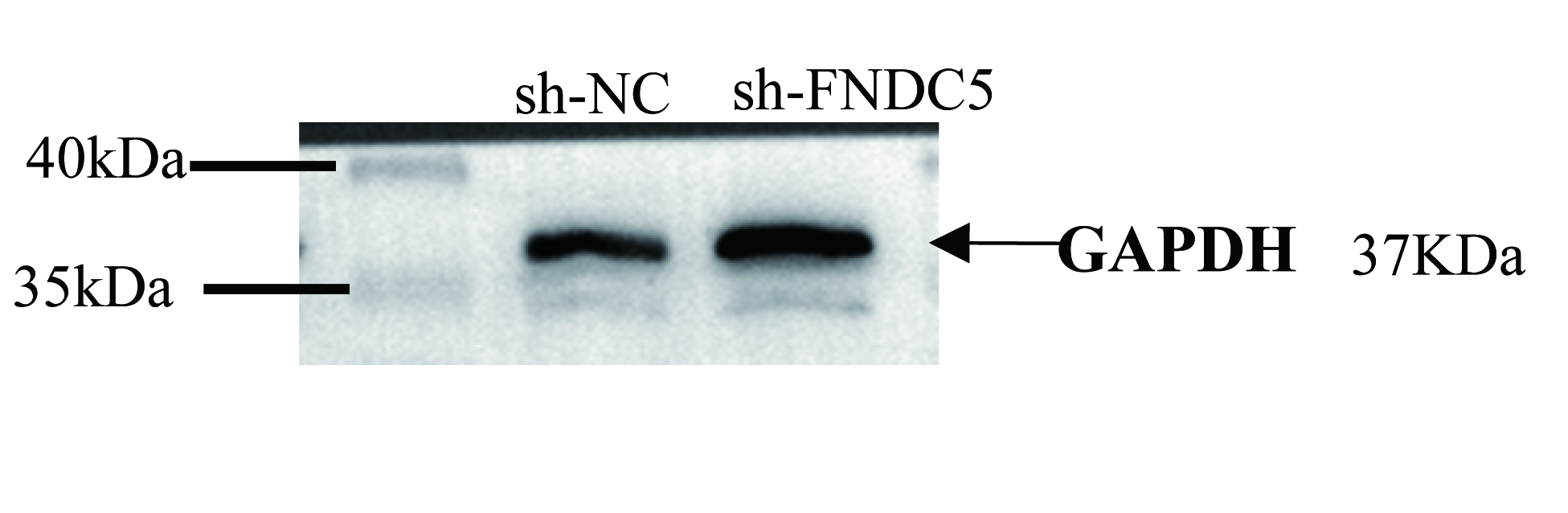

Supplement: Supplementary file 5 — Supplementary Material 5. [file 13395_2026_420_MOESM5_ESM.zip › Supplementary Material 5/Fig1/Fig1J/GAPDH/GAPDH-3.tif]

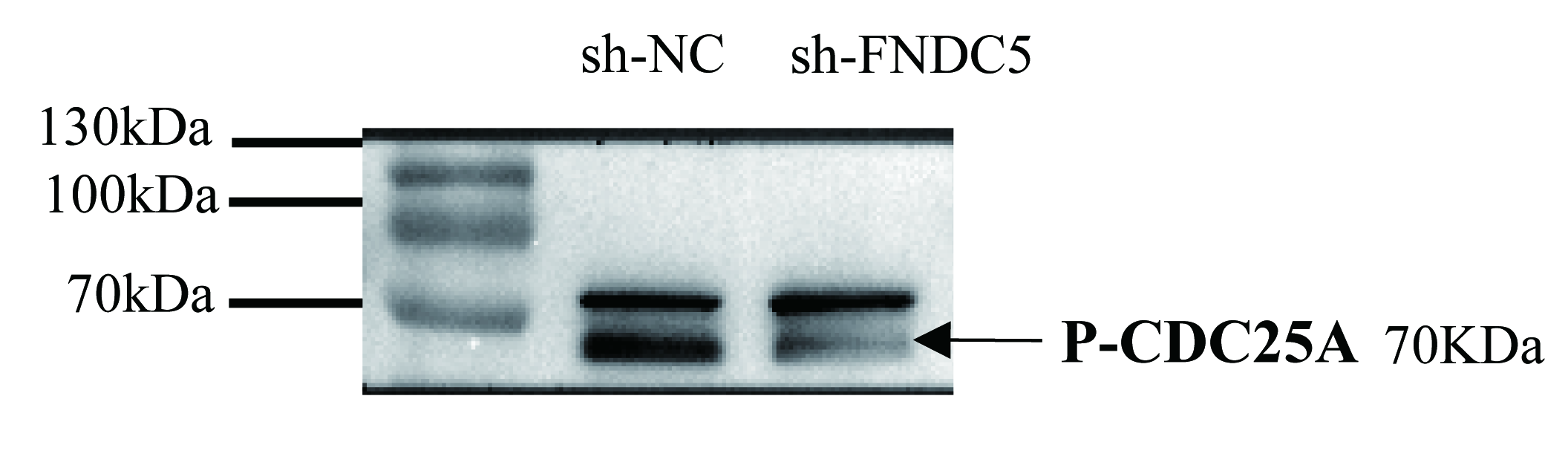

Supplement: Supplementary file 5 — Supplementary Material 5. [file 13395_2026_420_MOESM5_ESM.zip › Supplementary Material 5/Fig1/Fig1J/P-CDC25A/P-CDC25A-1.tif]

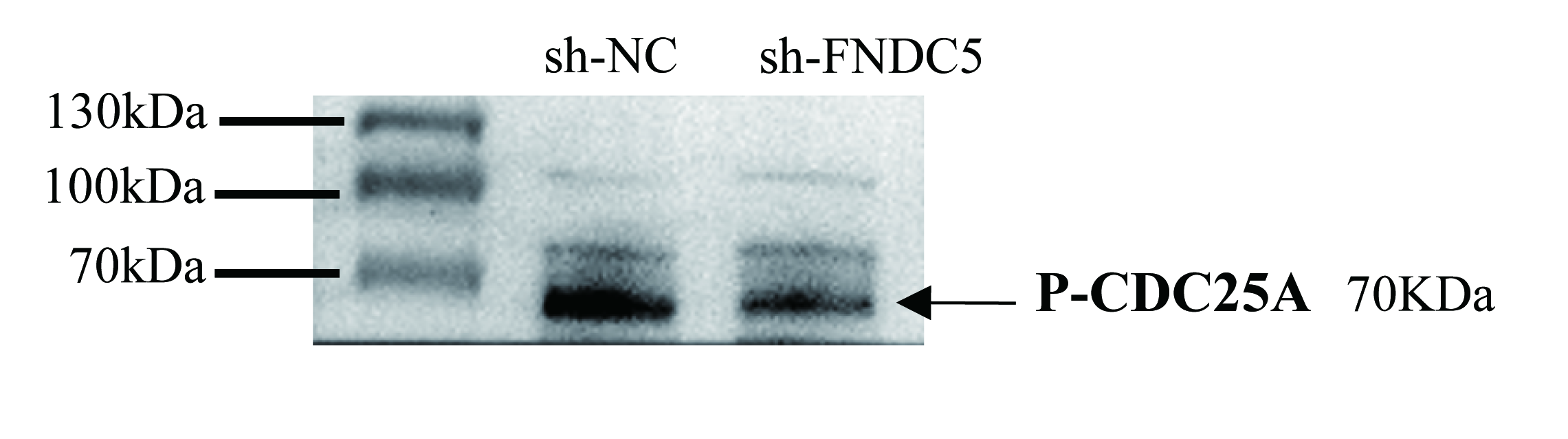

Supplement: Supplementary file 5 — Supplementary Material 5. [file 13395_2026_420_MOESM5_ESM.zip › Supplementary Material 5/Fig1/Fig1J/P-CDC25A/P-CDC25A-2.tif]

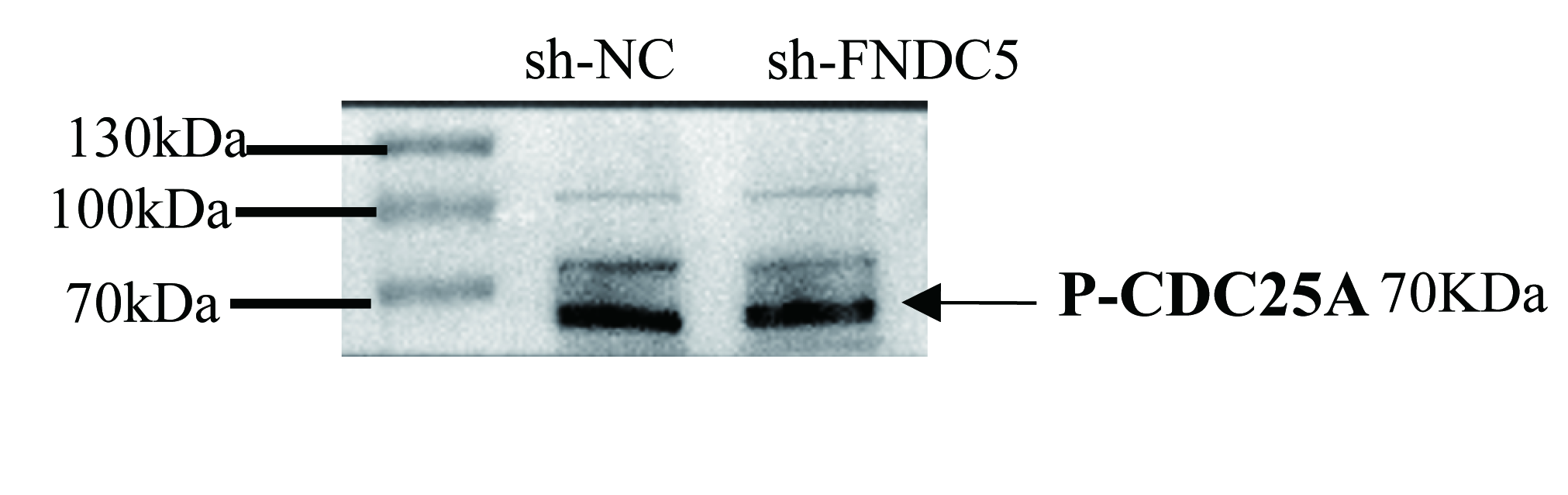

Supplement: Supplementary file 5 — Supplementary Material 5. [file 13395_2026_420_MOESM5_ESM.zip › Supplementary Material 5/Fig1/Fig1J/P-CDC25A/P-CDC25A-3.tif]

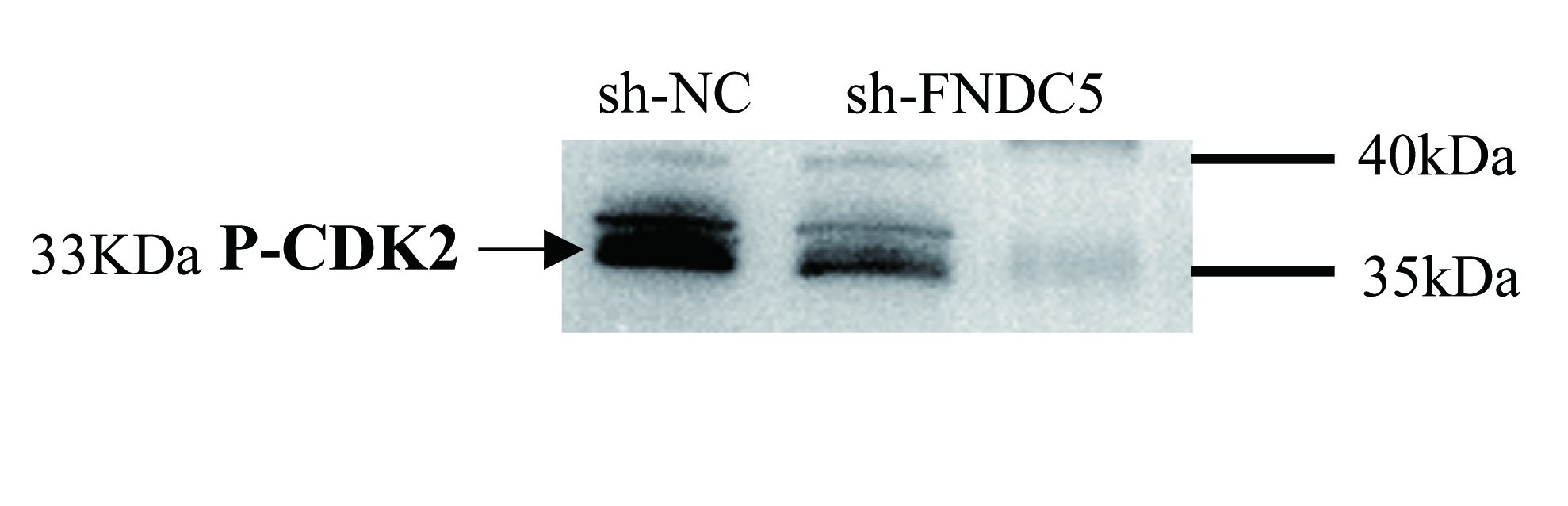

Supplement: Supplementary file 5 — Supplementary Material 5. [file 13395_2026_420_MOESM5_ESM.zip › Supplementary Material 5/Fig1/Fig1J/P-CDK2/P-CDK2-1.tif]

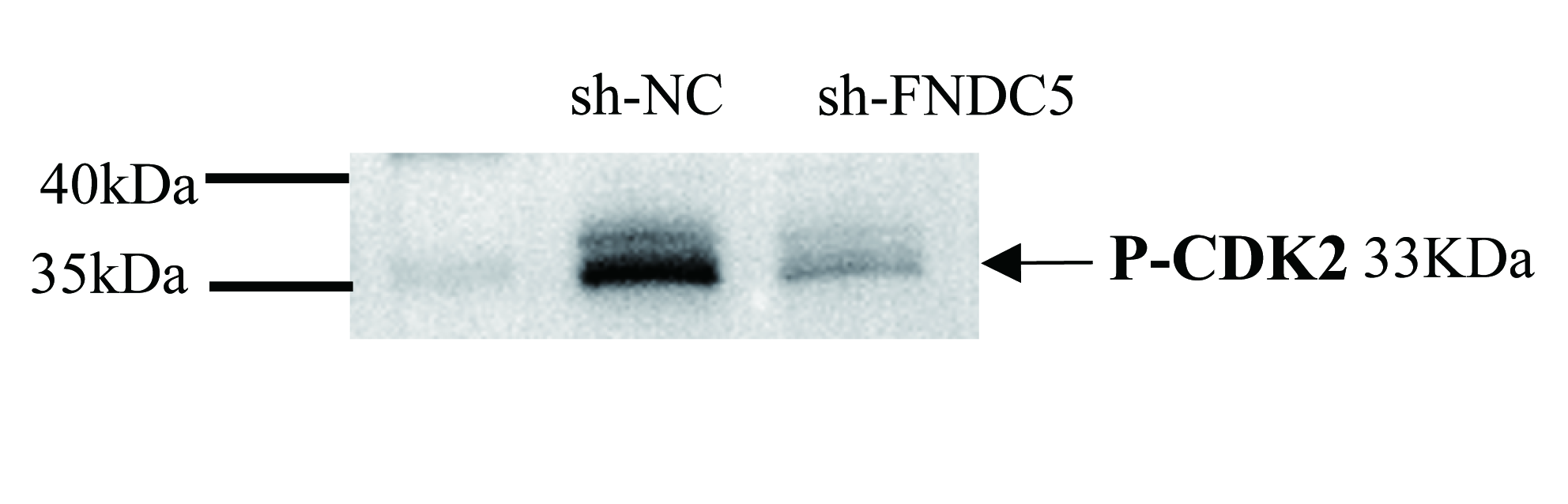

Supplement: Supplementary file 5 — Supplementary Material 5. [file 13395_2026_420_MOESM5_ESM.zip › Supplementary Material 5/Fig1/Fig1J/P-CDK2/P-CDK2-2.tif]

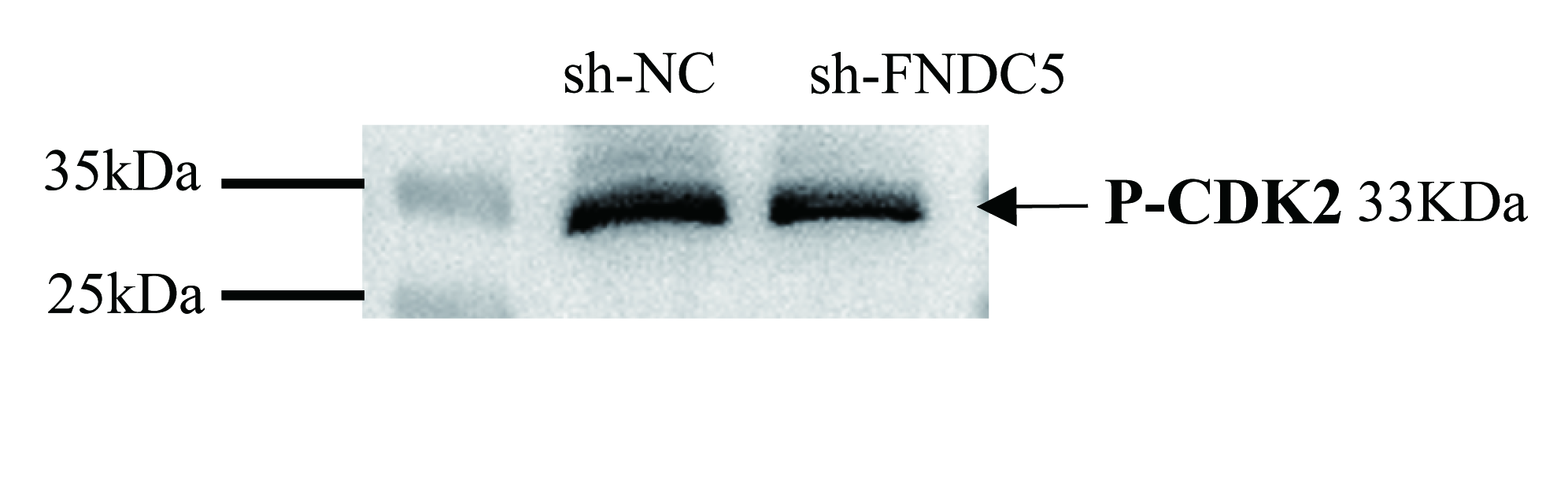

Supplement: Supplementary file 5 — Supplementary Material 5. [file 13395_2026_420_MOESM5_ESM.zip › Supplementary Material 5/Fig1/Fig1J/P-CDK2/P-CDK2-3.tif]

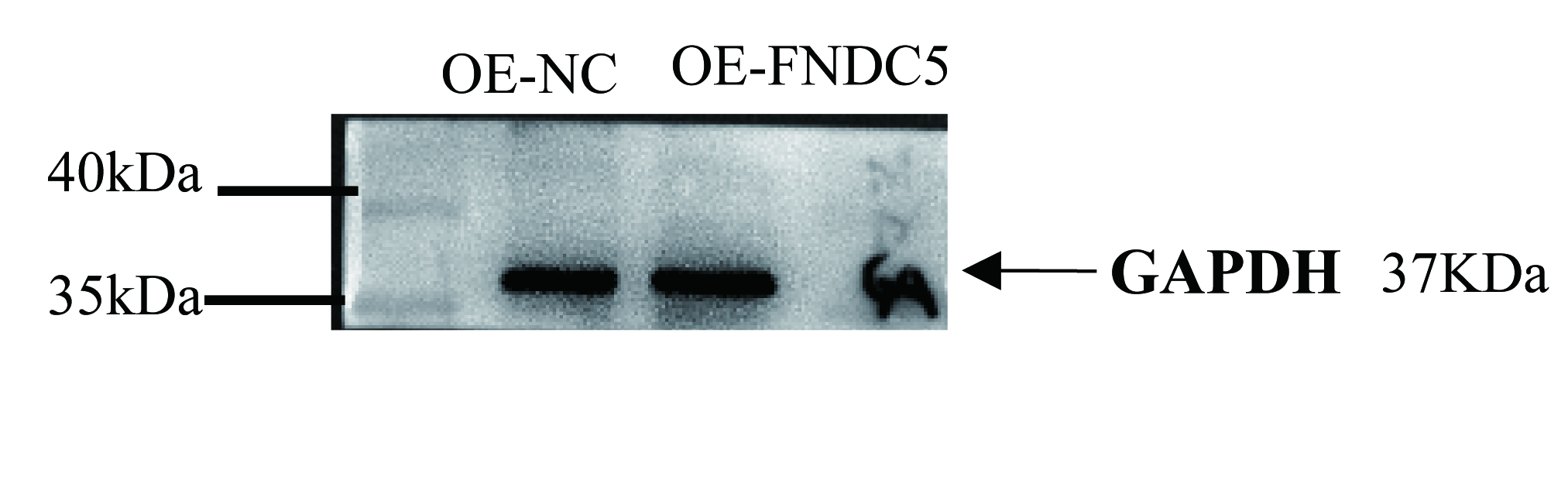

Supplement: Supplementary file 5 — Supplementary Material 5. [file 13395_2026_420_MOESM5_ESM.zip › Supplementary Material 5/Fig1/Fig1P/GAPDH/GAPDH-1.tif]

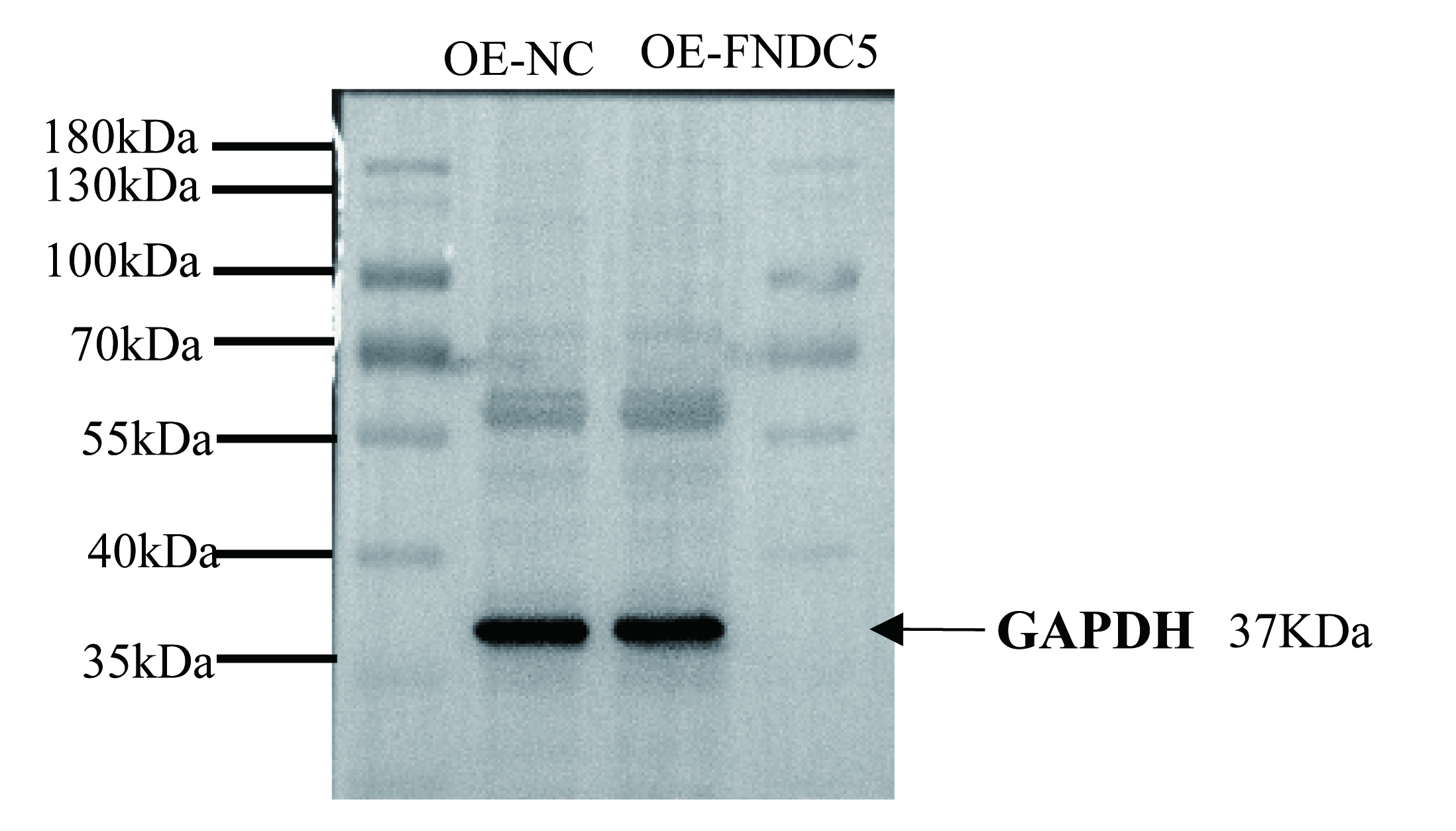

Supplement: Supplementary file 5 — Supplementary Material 5. [file 13395_2026_420_MOESM5_ESM.zip › Supplementary Material 5/Fig1/Fig1P/GAPDH/GAPDH-2.tif]

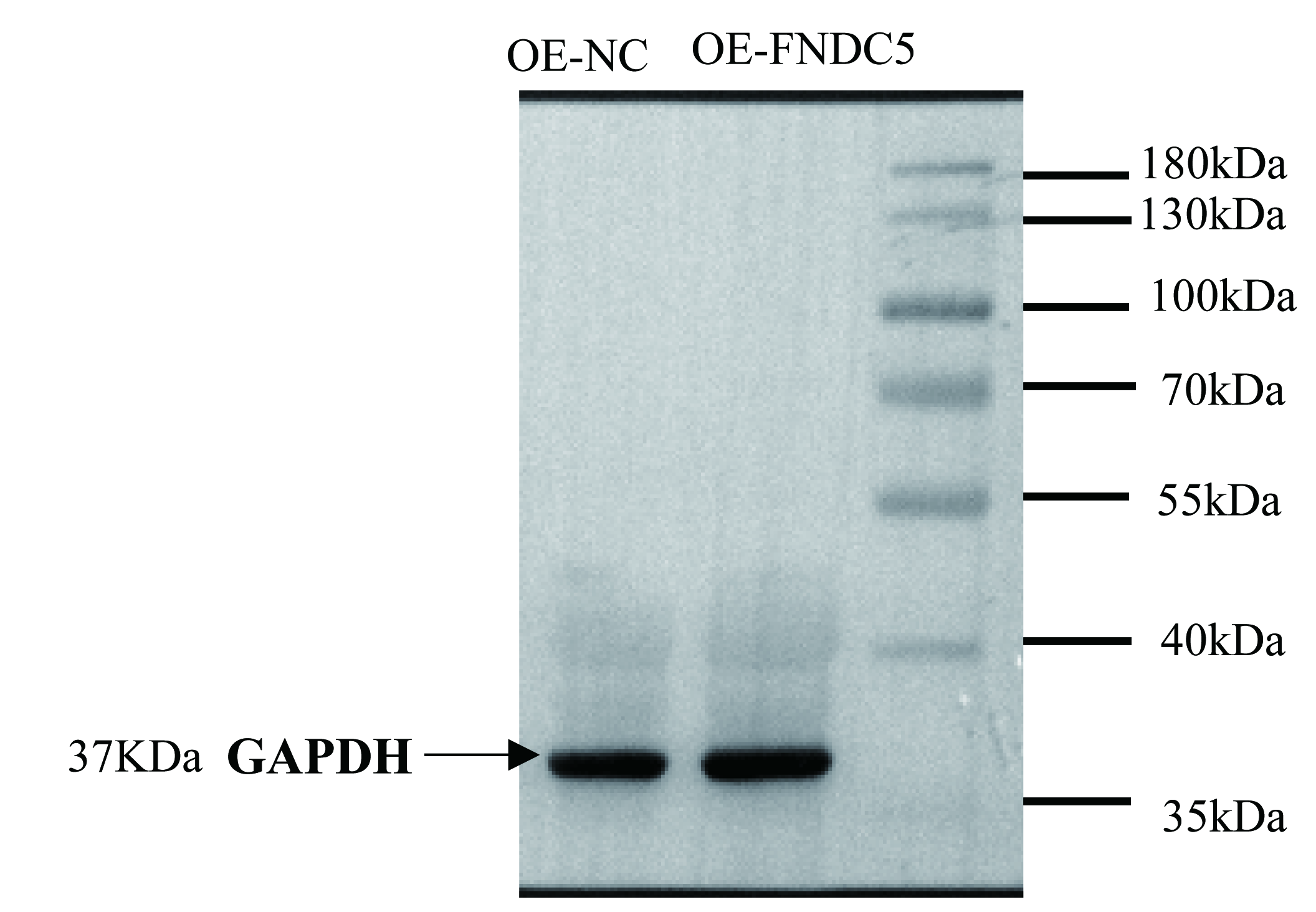

Supplement: Supplementary file 5 — Supplementary Material 5. [file 13395_2026_420_MOESM5_ESM.zip › Supplementary Material 5/Fig1/Fig1P/GAPDH/GAPDH-3.tif]

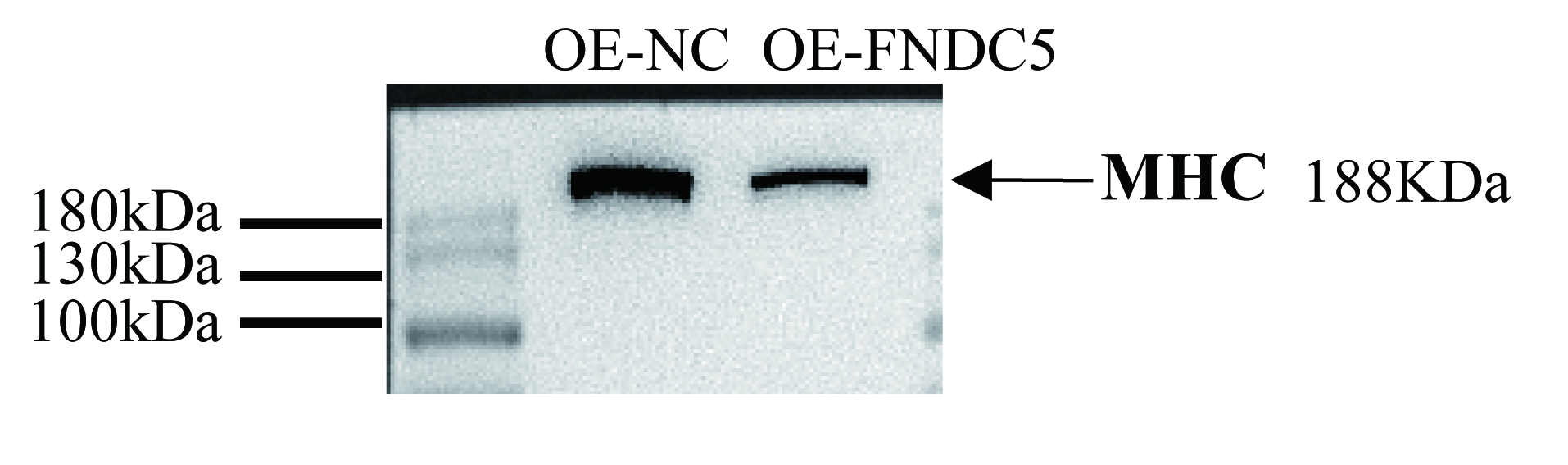

Supplement: Supplementary file 5 — Supplementary Material 5. [file 13395_2026_420_MOESM5_ESM.zip › Supplementary Material 5/Fig1/Fig1P/MHC/MHC-1.tif]

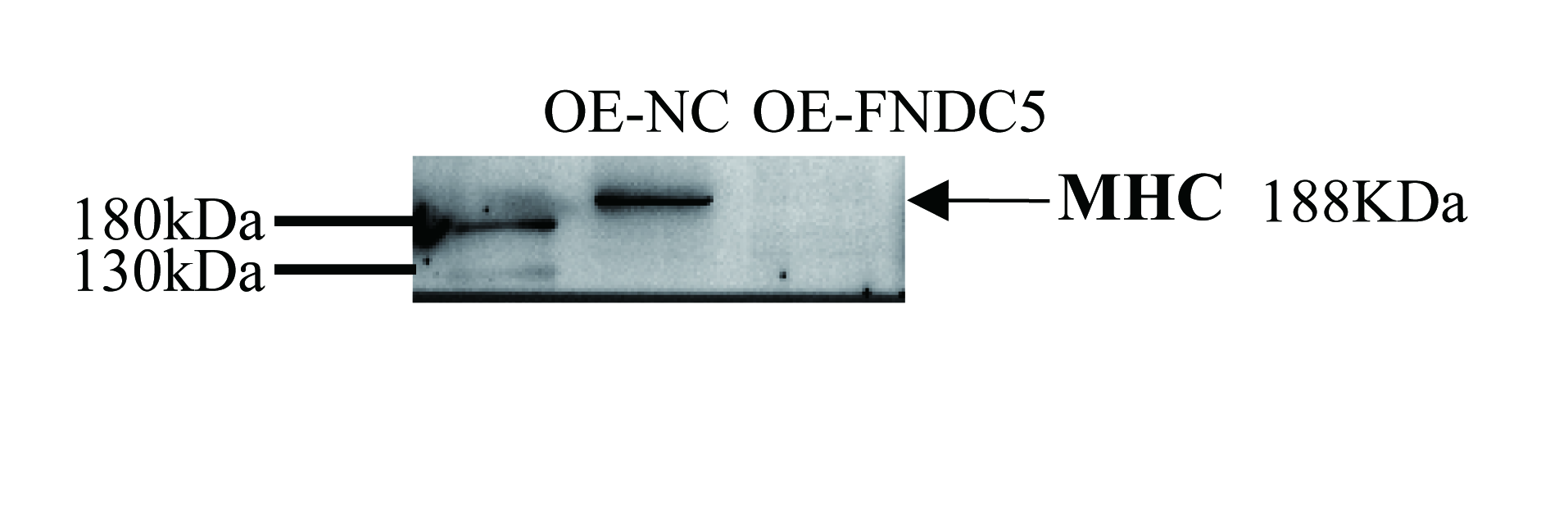

Supplement: Supplementary file 5 — Supplementary Material 5. [file 13395_2026_420_MOESM5_ESM.zip › Supplementary Material 5/Fig1/Fig1P/MHC/MHC-2.tif]

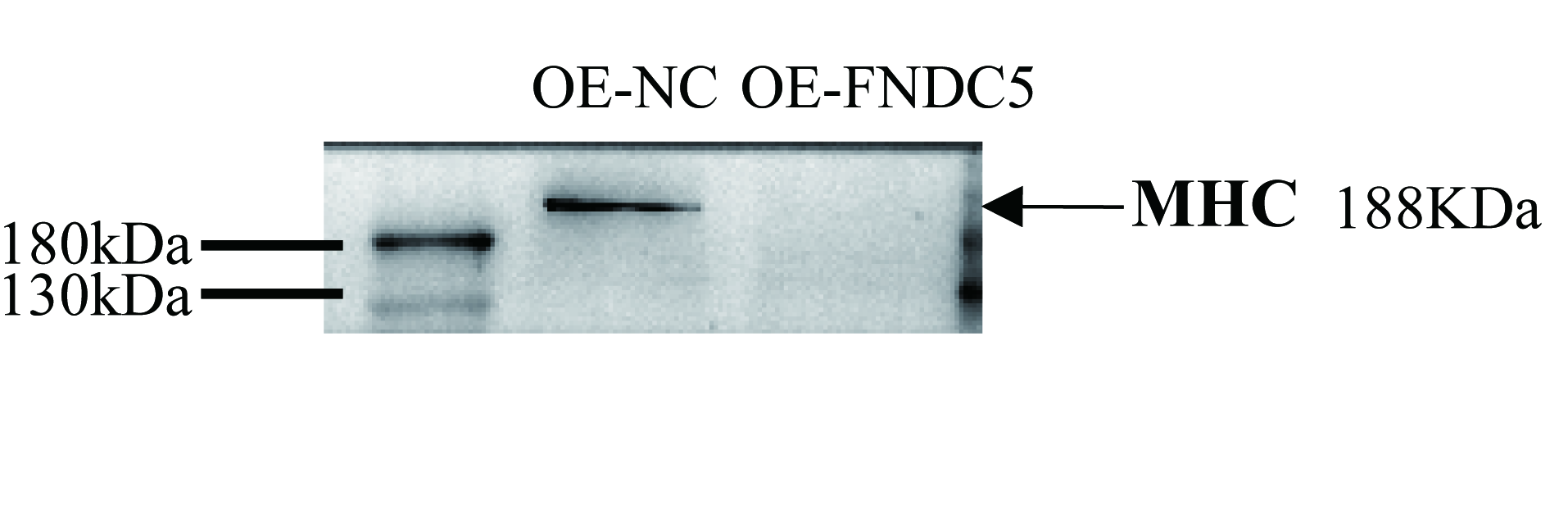

Supplement: Supplementary file 5 — Supplementary Material 5. [file 13395_2026_420_MOESM5_ESM.zip › Supplementary Material 5/Fig1/Fig1P/MHC/MHC-3.tif]

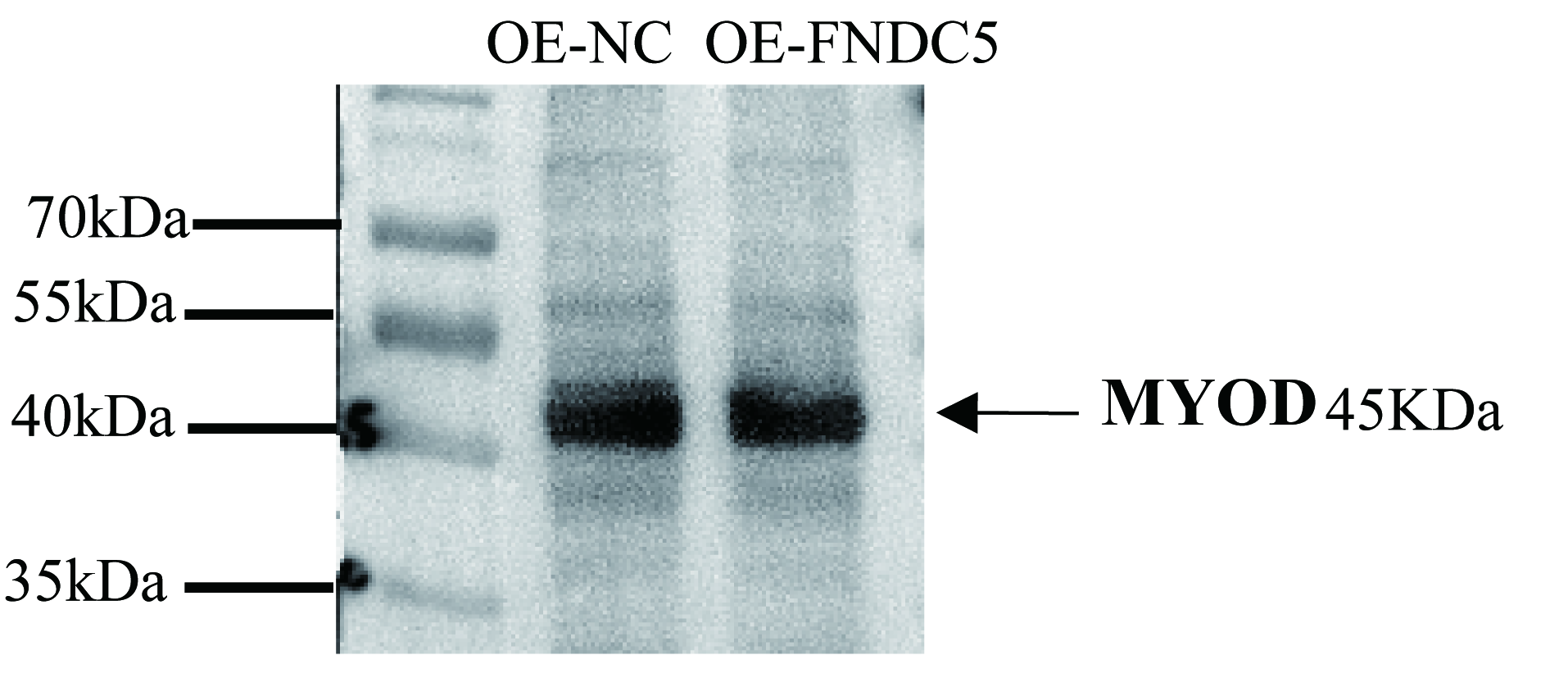

Supplement: Supplementary file 5 — Supplementary Material 5. [file 13395_2026_420_MOESM5_ESM.zip › Supplementary Material 5/Fig1/Fig1P/MYOD/MYOD-1.tif]

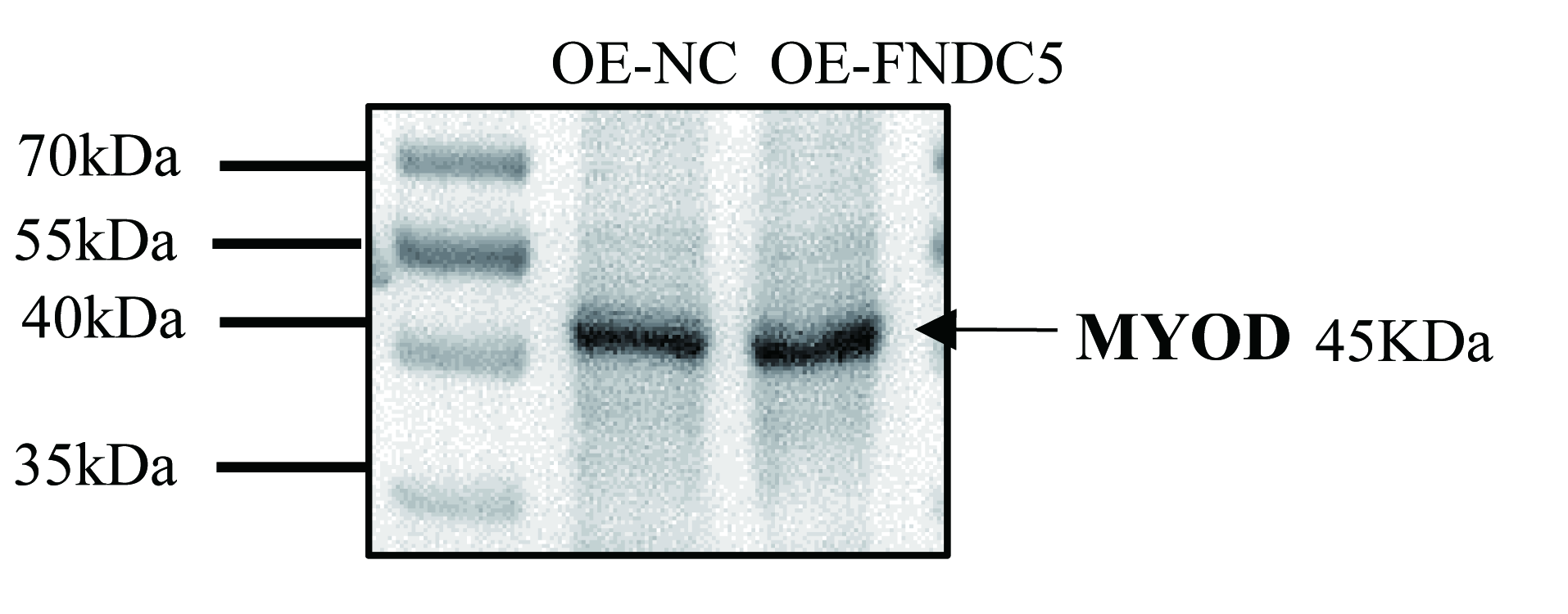

Supplement: Supplementary file 5 — Supplementary Material 5. [file 13395_2026_420_MOESM5_ESM.zip › Supplementary Material 5/Fig1/Fig1P/MYOD/MYOD-2.tif]

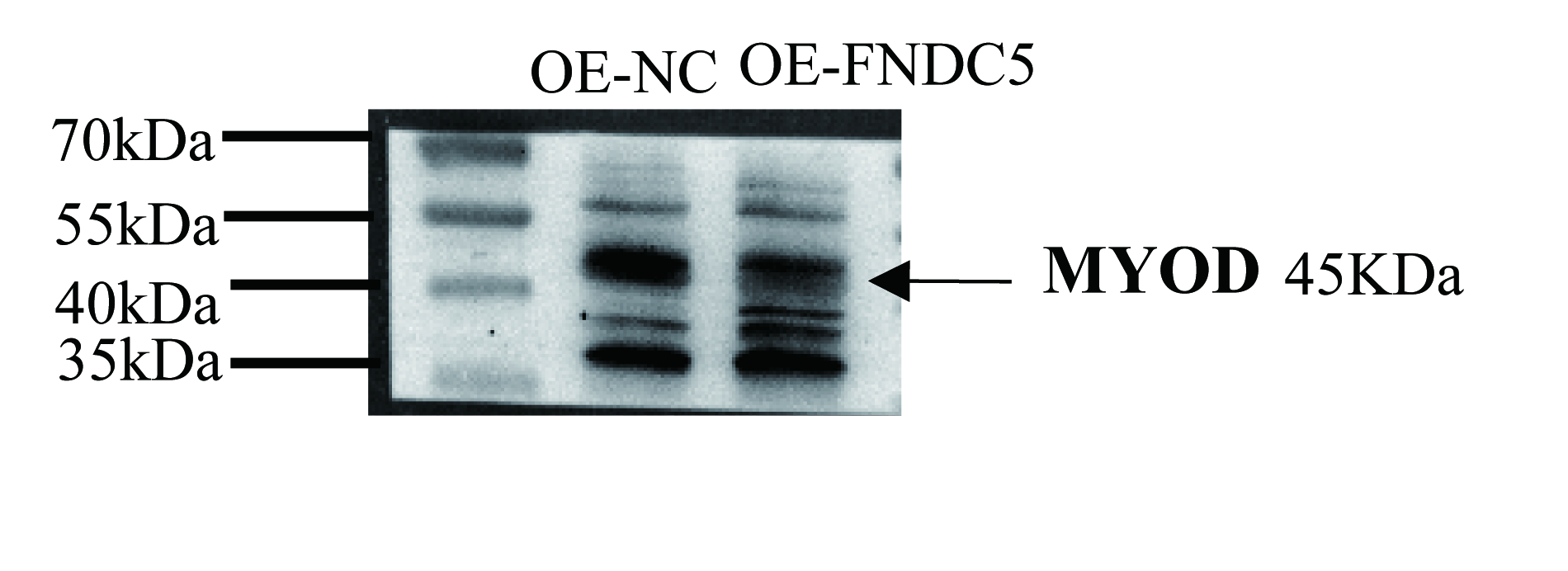

Supplement: Supplementary file 5 — Supplementary Material 5. [file 13395_2026_420_MOESM5_ESM.zip › Supplementary Material 5/Fig1/Fig1P/MYOD/MYOD-3.tif]

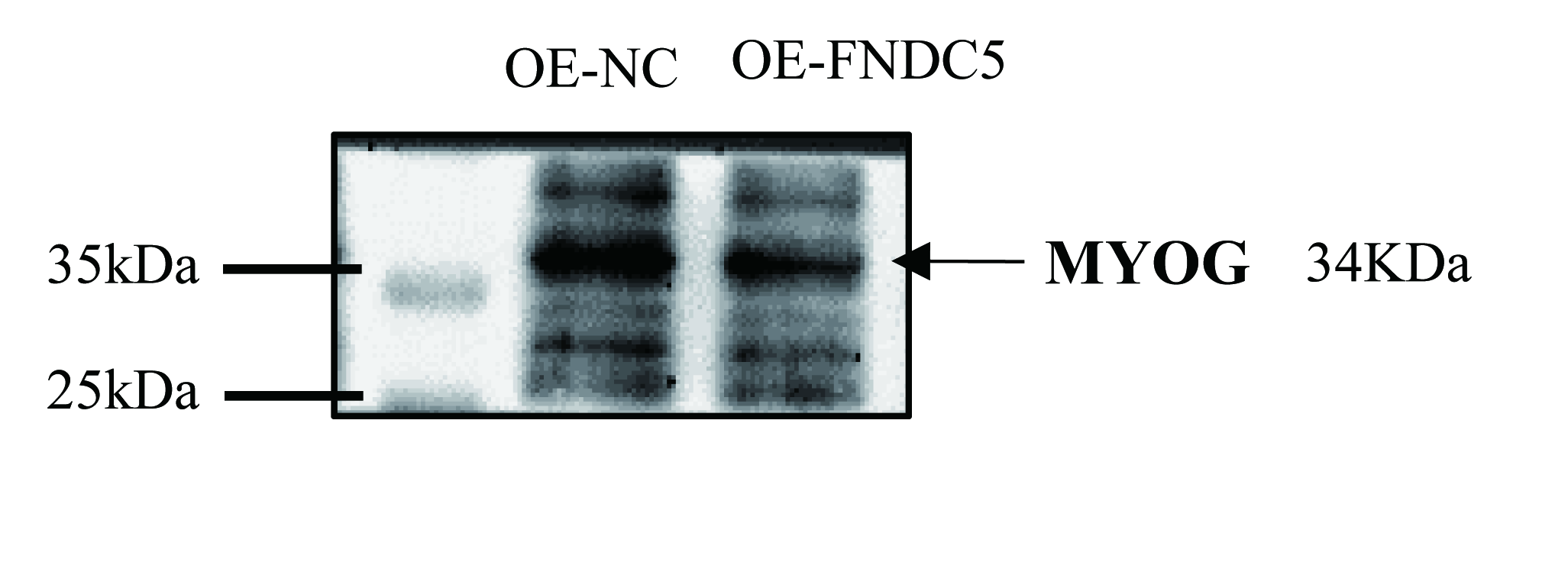

Supplement: Supplementary file 5 — Supplementary Material 5. [file 13395_2026_420_MOESM5_ESM.zip › Supplementary Material 5/Fig1/Fig1P/MYOG/MYOG-1.tif]

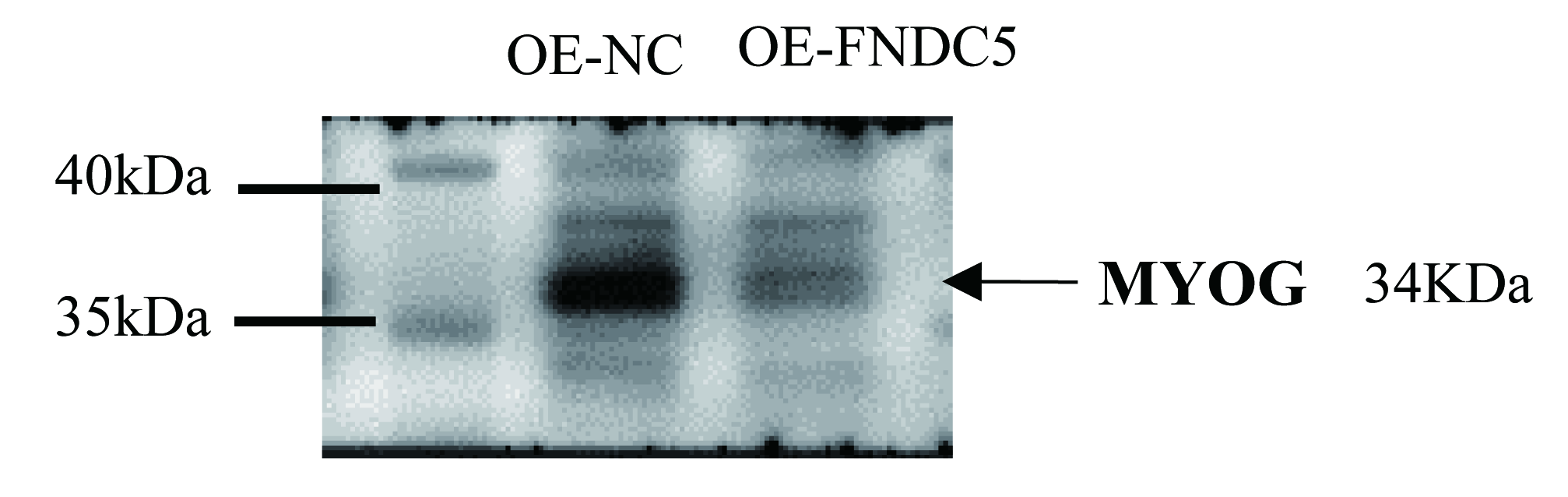

Supplement: Supplementary file 5 — Supplementary Material 5. [file 13395_2026_420_MOESM5_ESM.zip › Supplementary Material 5/Fig1/Fig1P/MYOG/MYOG-2.tif]

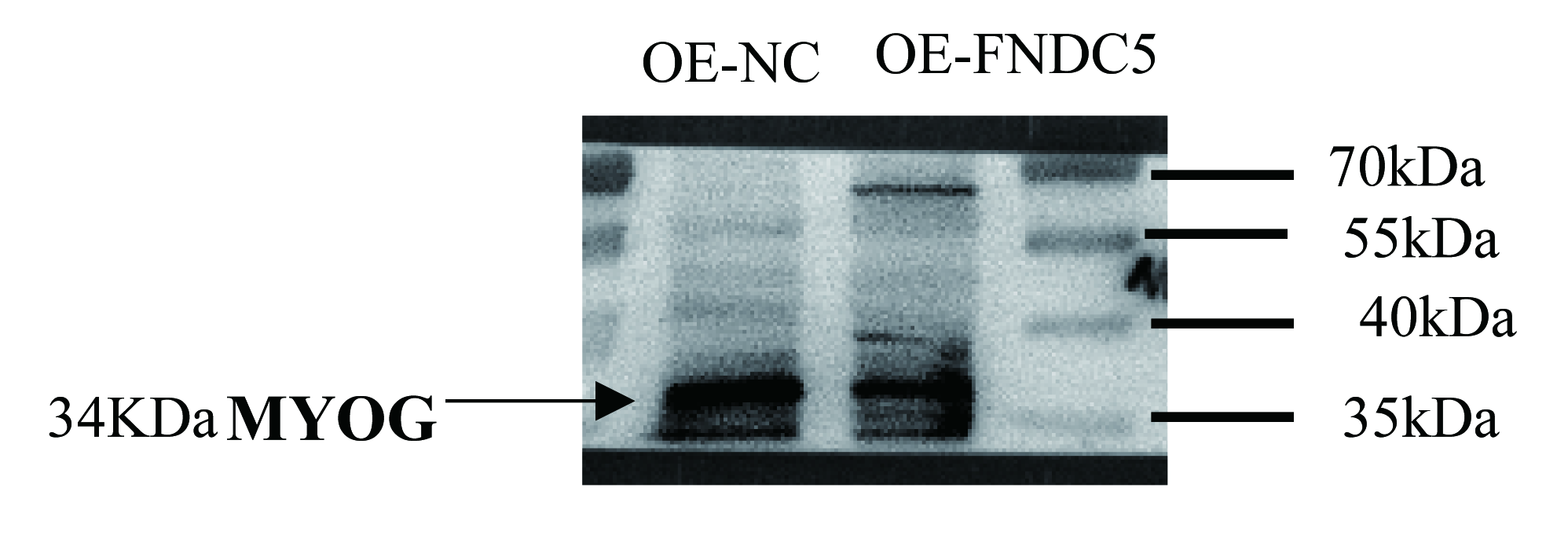

Supplement: Supplementary file 5 — Supplementary Material 5. [file 13395_2026_420_MOESM5_ESM.zip › Supplementary Material 5/Fig1/Fig1P/MYOG/MYOG-3.tif]

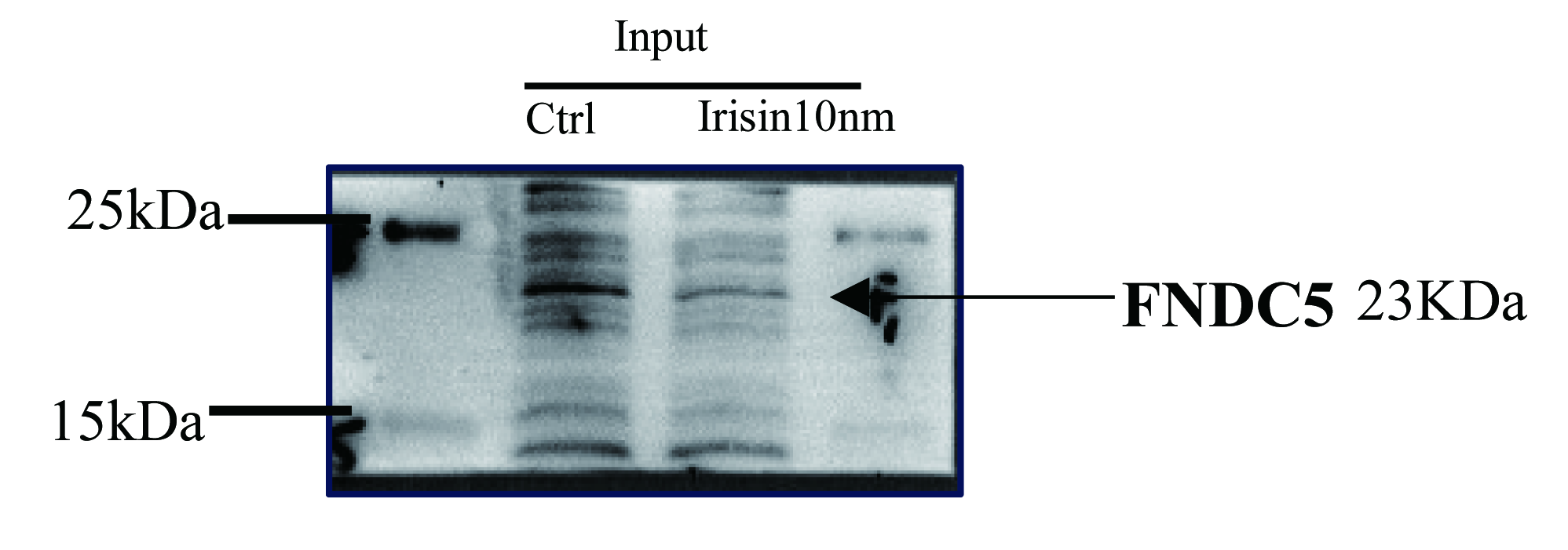

Supplement: Supplementary file 5 — Supplementary Material 5. [file 13395_2026_420_MOESM5_ESM.zip › Supplementary Material 5/Fig2/Fig2A/FNDC5-input.tif]

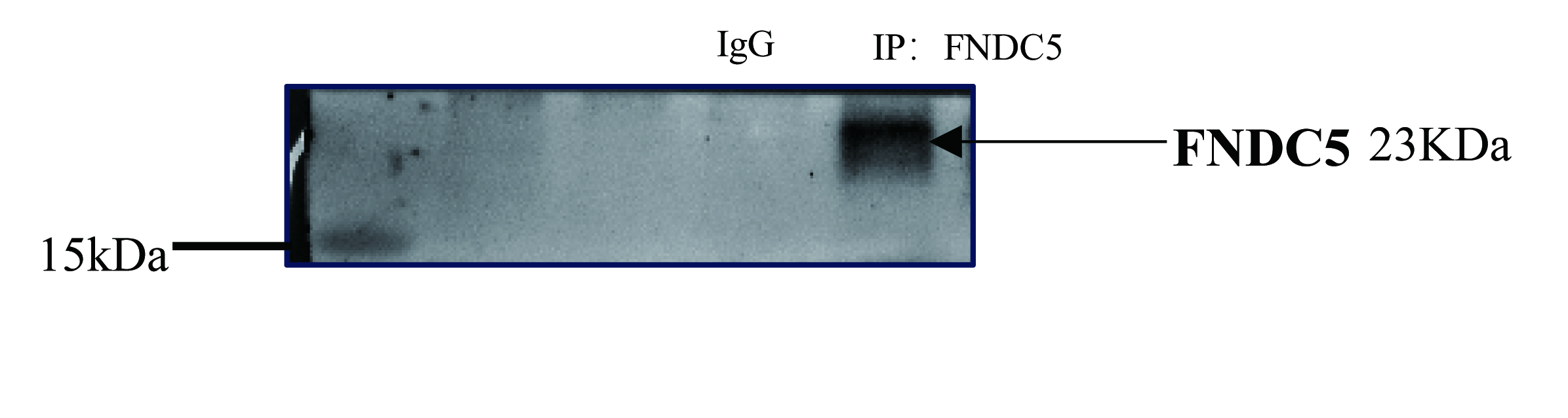

Supplement: Supplementary file 5 — Supplementary Material 5. [file 13395_2026_420_MOESM5_ESM.zip › Supplementary Material 5/Fig2/Fig2A/IgG+IP-FNDC5.tif]

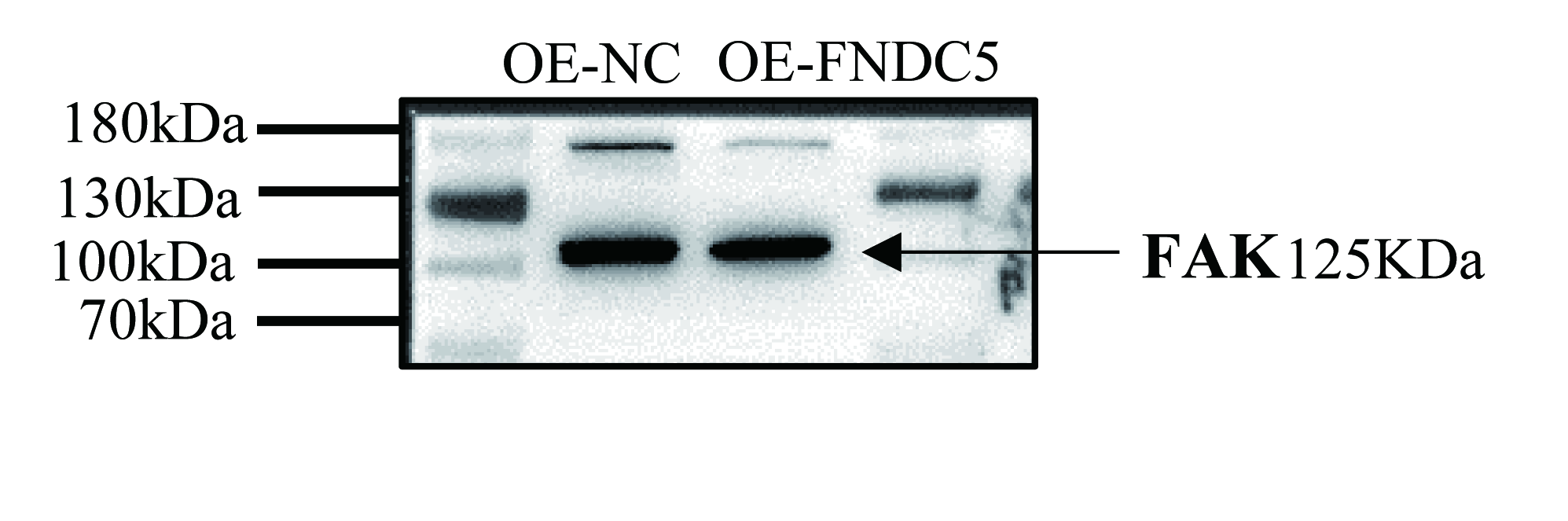

Supplement: Supplementary file 5 — Supplementary Material 5. [file 13395_2026_420_MOESM5_ESM.zip › Supplementary Material 5/Fig2/Fig2C/OE-FNDC5/FAK/FAK-1.tif]

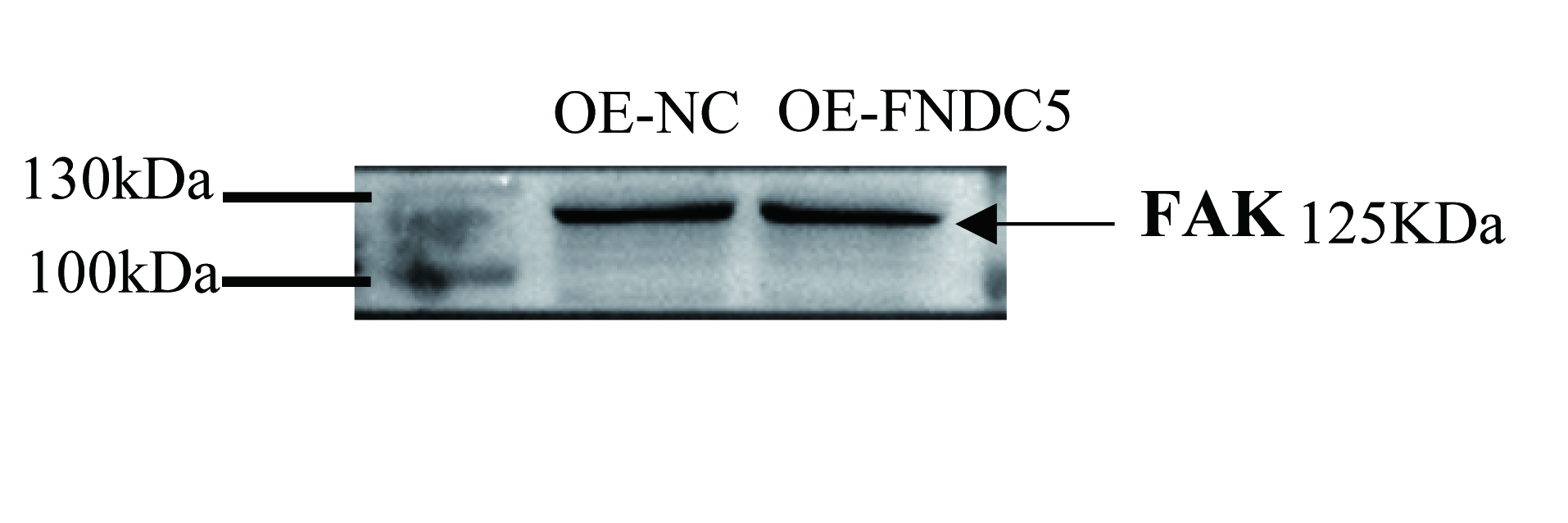

Supplement: Supplementary file 5 — Supplementary Material 5. [file 13395_2026_420_MOESM5_ESM.zip › Supplementary Material 5/Fig2/Fig2C/OE-FNDC5/FAK/FAK-2.tif]

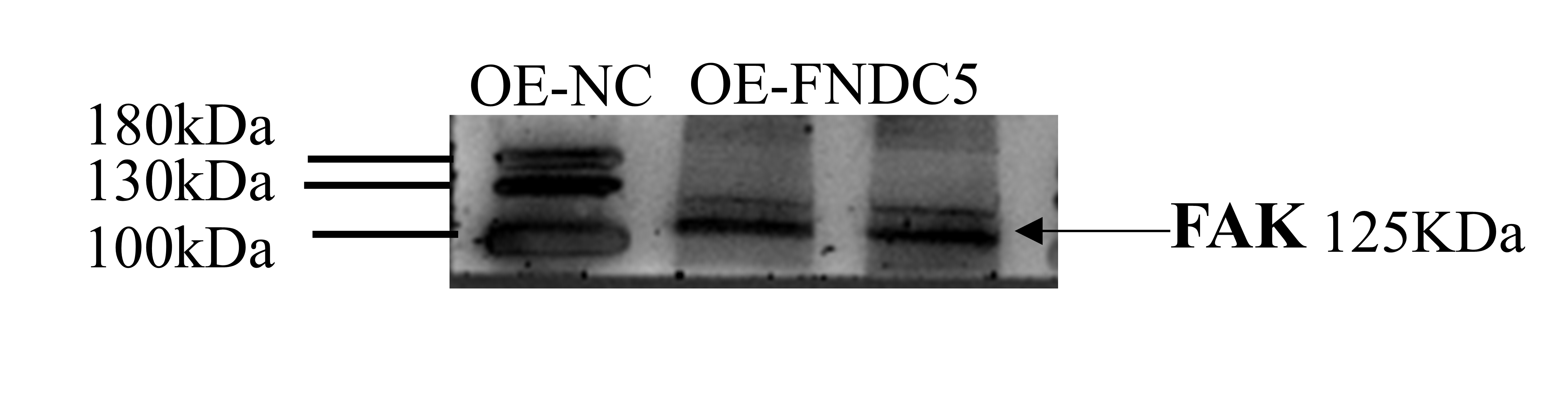

Supplement: Supplementary file 5 — Supplementary Material 5. [file 13395_2026_420_MOESM5_ESM.zip › Supplementary Material 5/Fig2/Fig2C/OE-FNDC5/FAK/FAK-3.tif]

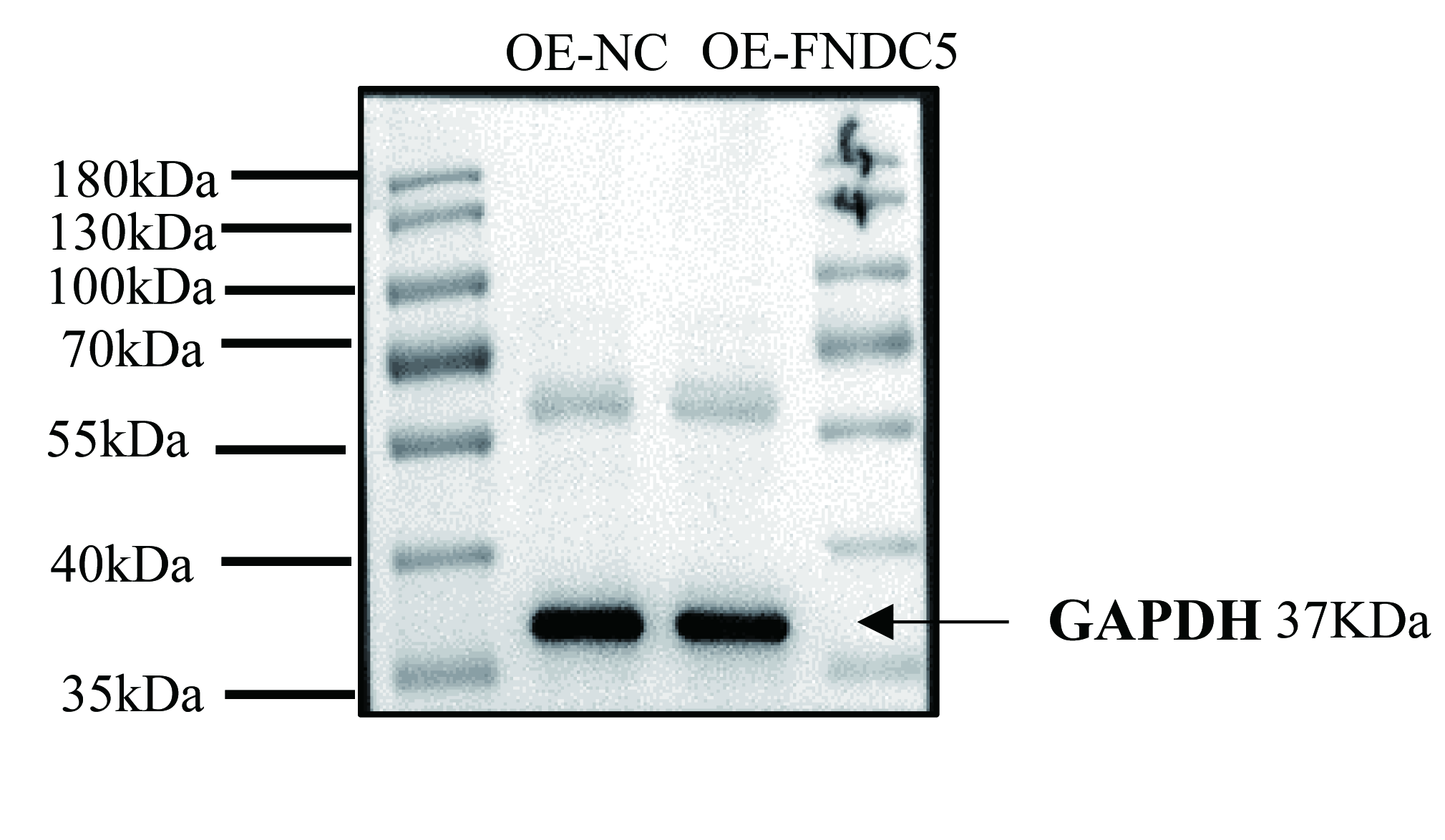

Supplement: Supplementary file 5 — Supplementary Material 5. [file 13395_2026_420_MOESM5_ESM.zip › Supplementary Material 5/Fig2/Fig2C/OE-FNDC5/GAPDH/GAPDH-1.tif]

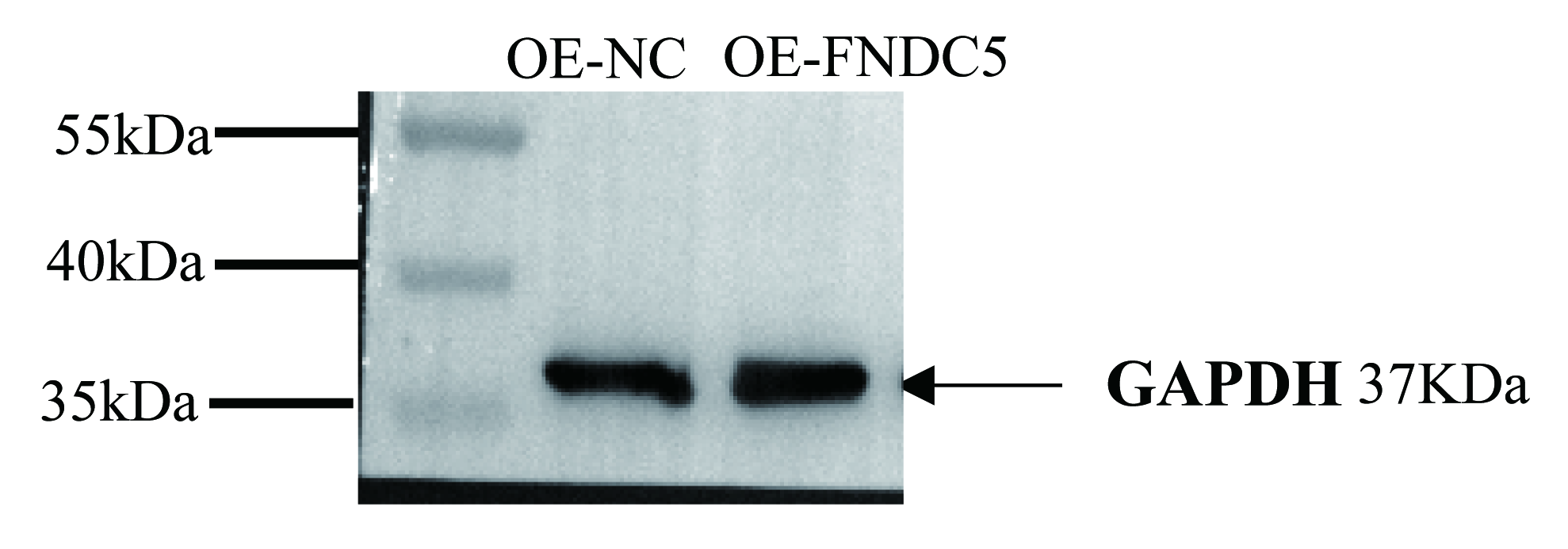

Supplement: Supplementary file 5 — Supplementary Material 5. [file 13395_2026_420_MOESM5_ESM.zip › Supplementary Material 5/Fig2/Fig2C/OE-FNDC5/GAPDH/GAPDH-2.tif]

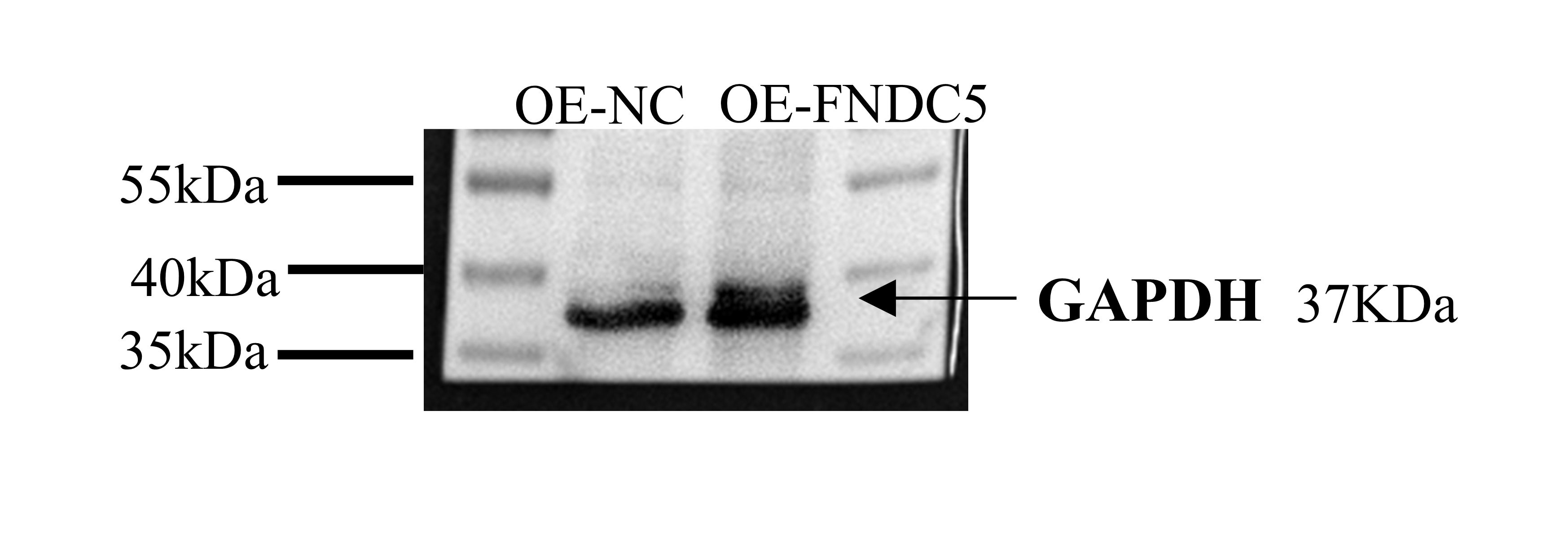

Supplement: Supplementary file 5 — Supplementary Material 5. [file 13395_2026_420_MOESM5_ESM.zip › Supplementary Material 5/Fig2/Fig2C/OE-FNDC5/GAPDH/GAPDH-3.tif]

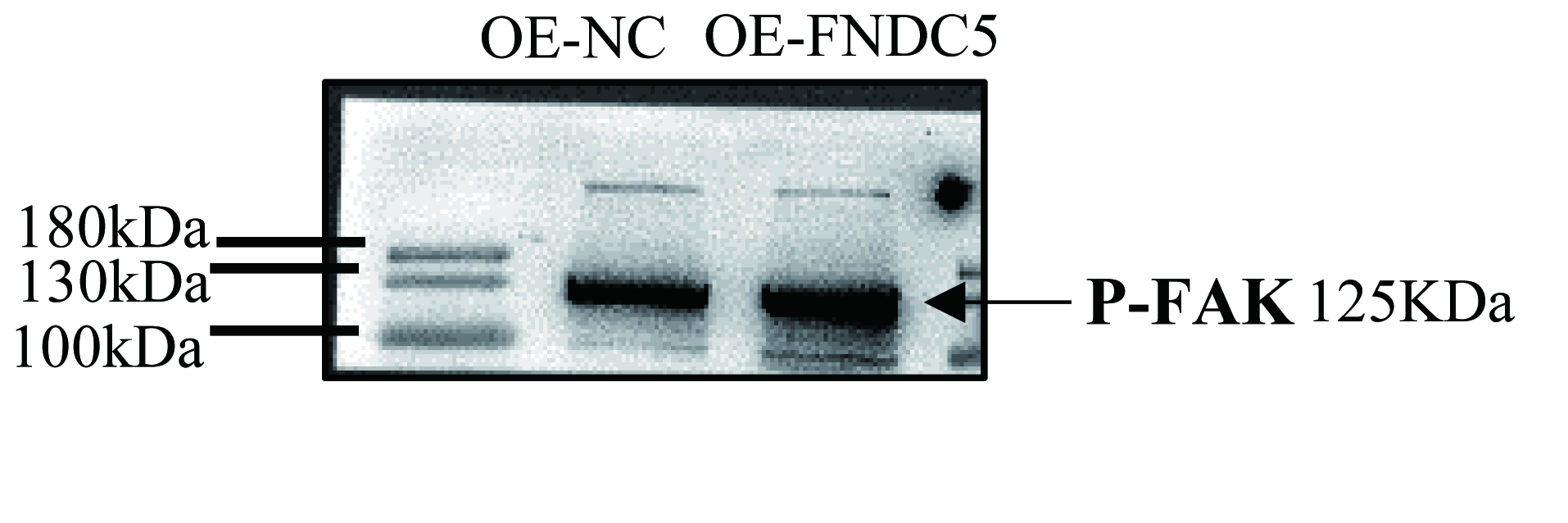

Supplement: Supplementary file 5 — Supplementary Material 5. [file 13395_2026_420_MOESM5_ESM.zip › Supplementary Material 5/Fig2/Fig2C/OE-FNDC5/P-FAK/P-FAK-1.tif]

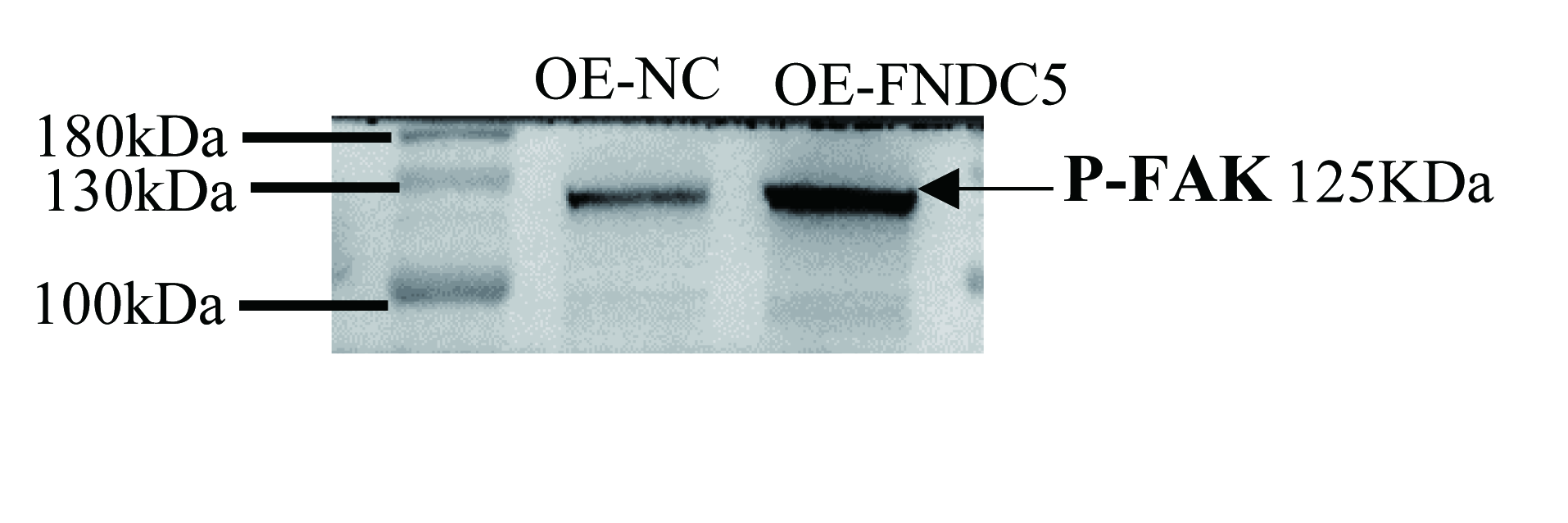

Supplement: Supplementary file 5 — Supplementary Material 5. [file 13395_2026_420_MOESM5_ESM.zip › Supplementary Material 5/Fig2/Fig2C/OE-FNDC5/P-FAK/P-FAK-2.tif]

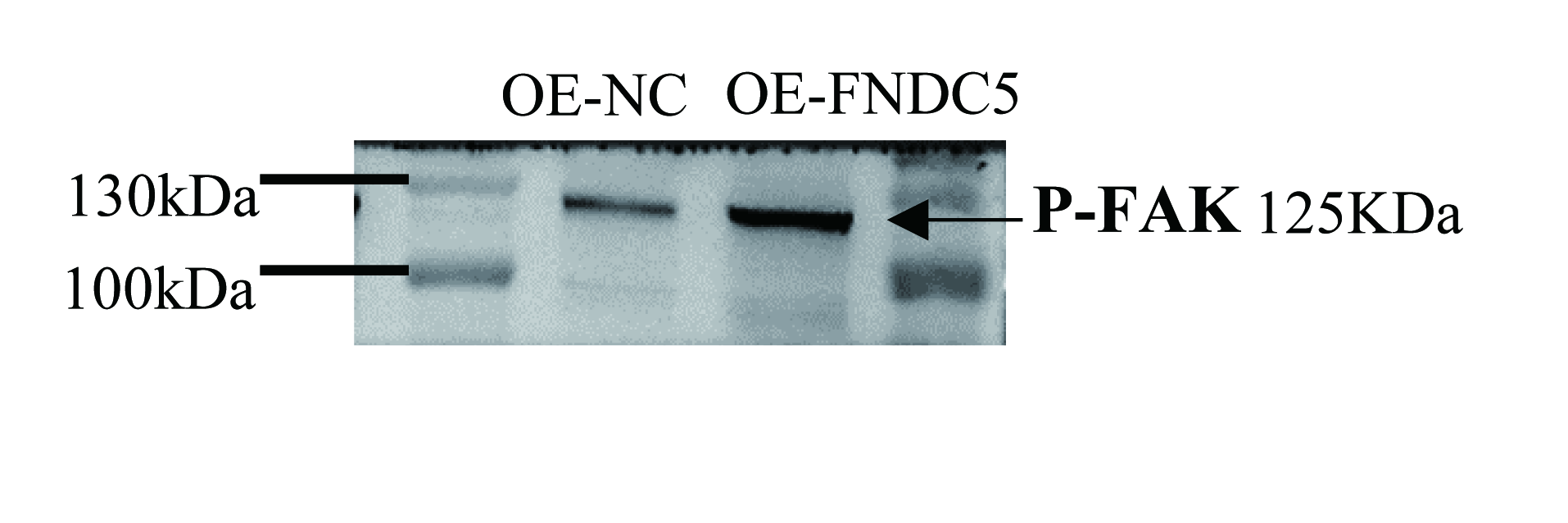

Supplement: Supplementary file 5 — Supplementary Material 5. [file 13395_2026_420_MOESM5_ESM.zip › Supplementary Material 5/Fig2/Fig2C/OE-FNDC5/P-FAK/P-FAK-3.tif]

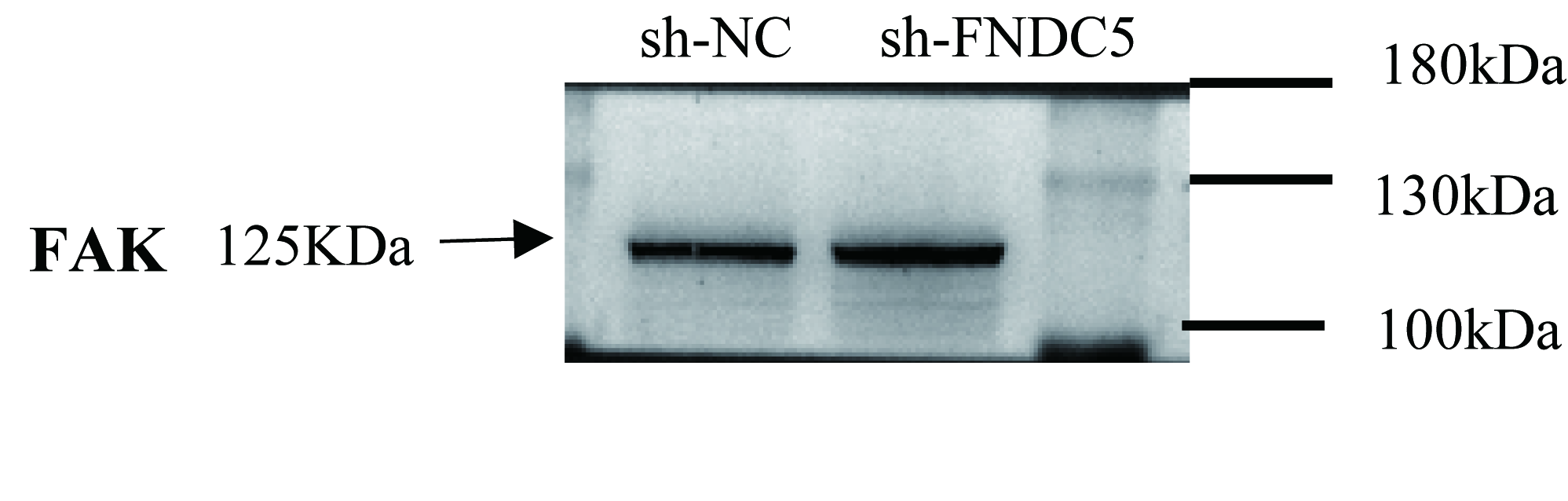

Supplement: Supplementary file 5 — Supplementary Material 5. [file 13395_2026_420_MOESM5_ESM.zip › Supplementary Material 5/Fig2/Fig2C/sh-FNDC5/FAK/FAK-1.tif]

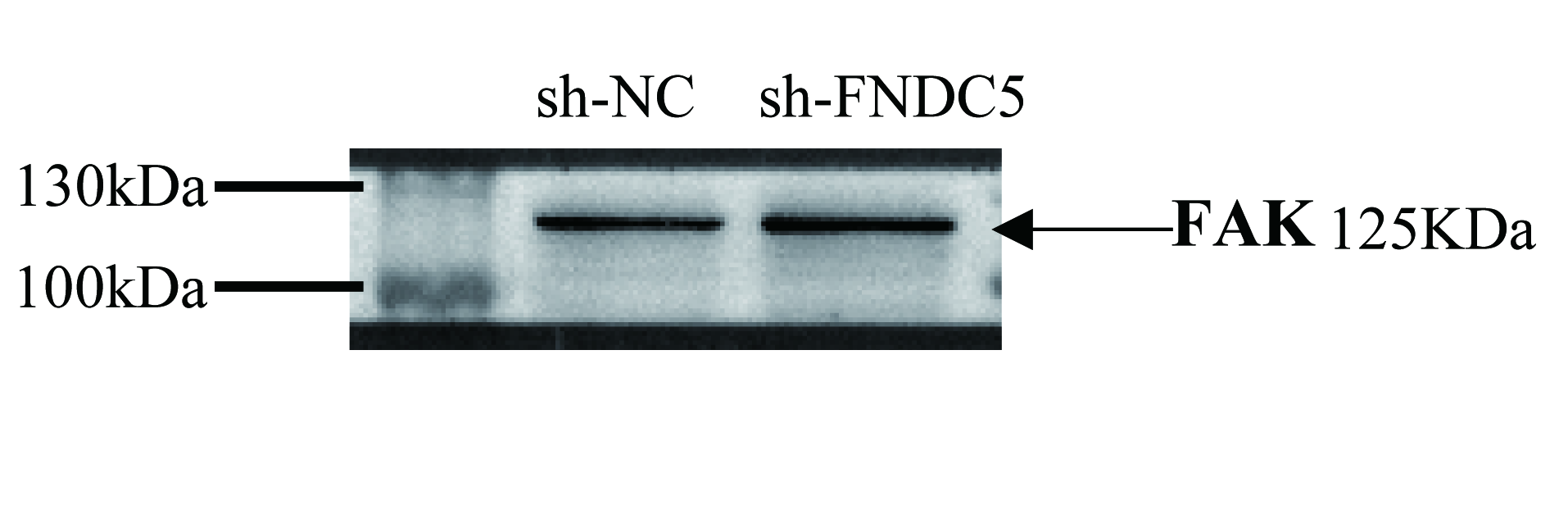

Supplement: Supplementary file 5 — Supplementary Material 5. [file 13395_2026_420_MOESM5_ESM.zip › Supplementary Material 5/Fig2/Fig2C/sh-FNDC5/FAK/FAK-2.tif]

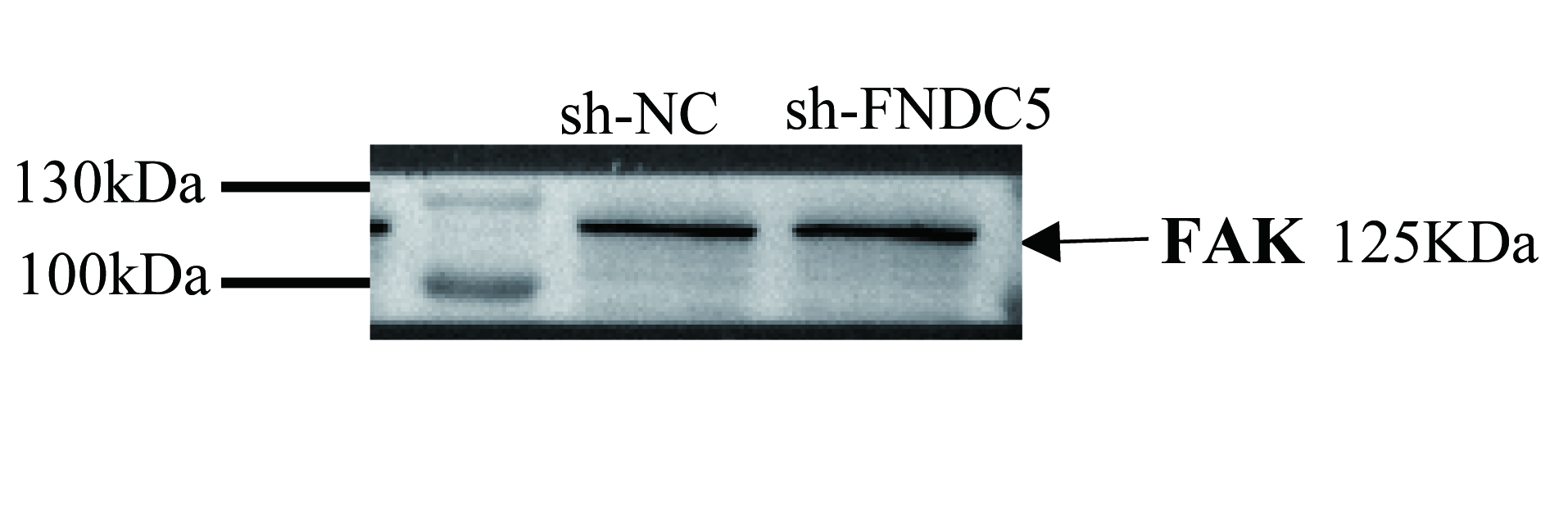

Supplement: Supplementary file 5 — Supplementary Material 5. [file 13395_2026_420_MOESM5_ESM.zip › Supplementary Material 5/Fig2/Fig2C/sh-FNDC5/FAK/FAK-3.tif]

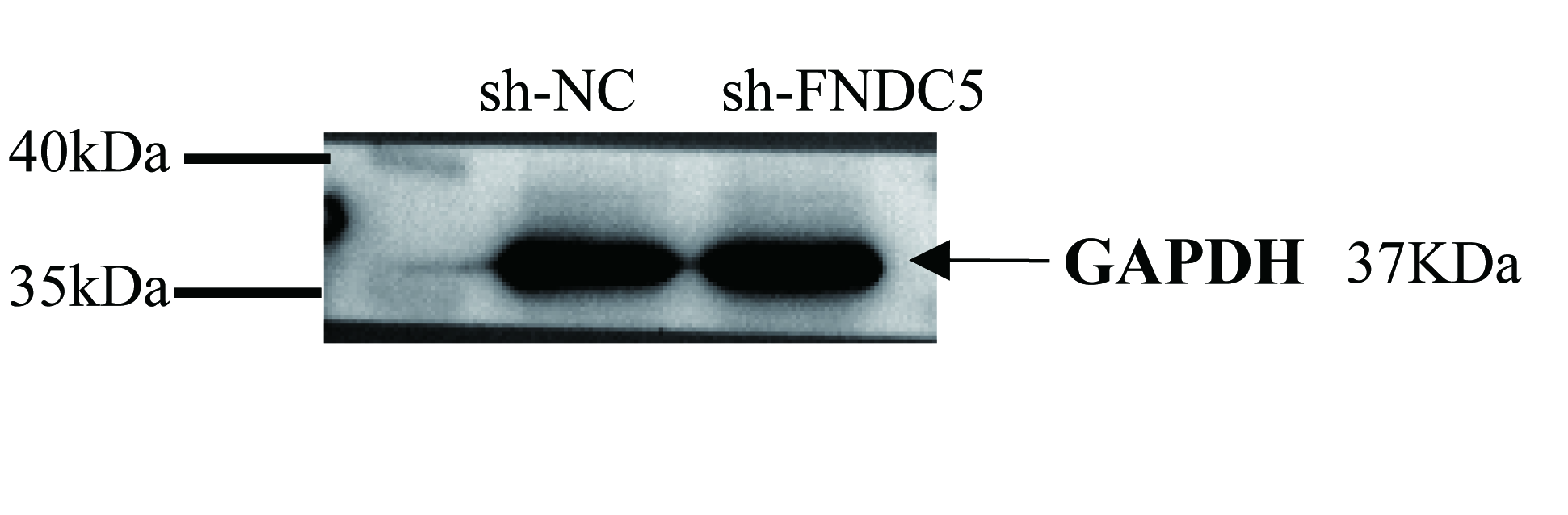

Supplement: Supplementary file 5 — Supplementary Material 5. [file 13395_2026_420_MOESM5_ESM.zip › Supplementary Material 5/Fig2/Fig2C/sh-FNDC5/GAPDH/GAPDH-1.tif]

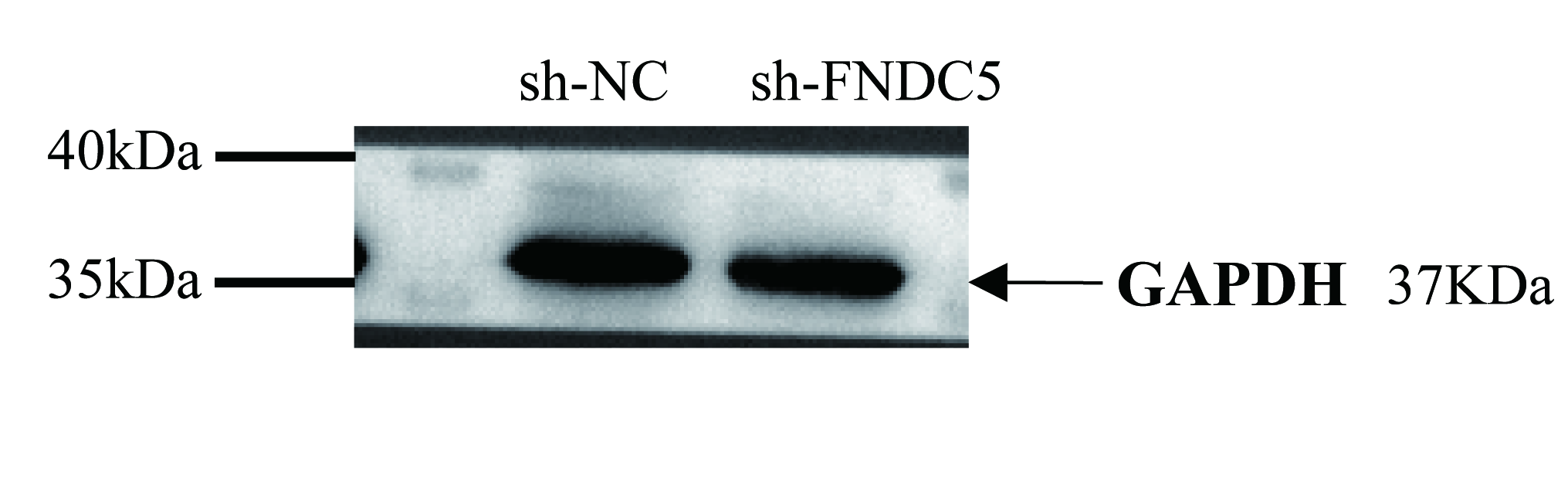

Supplement: Supplementary file 5 — Supplementary Material 5. [file 13395_2026_420_MOESM5_ESM.zip › Supplementary Material 5/Fig2/Fig2C/sh-FNDC5/GAPDH/GAPDH-2.tif]

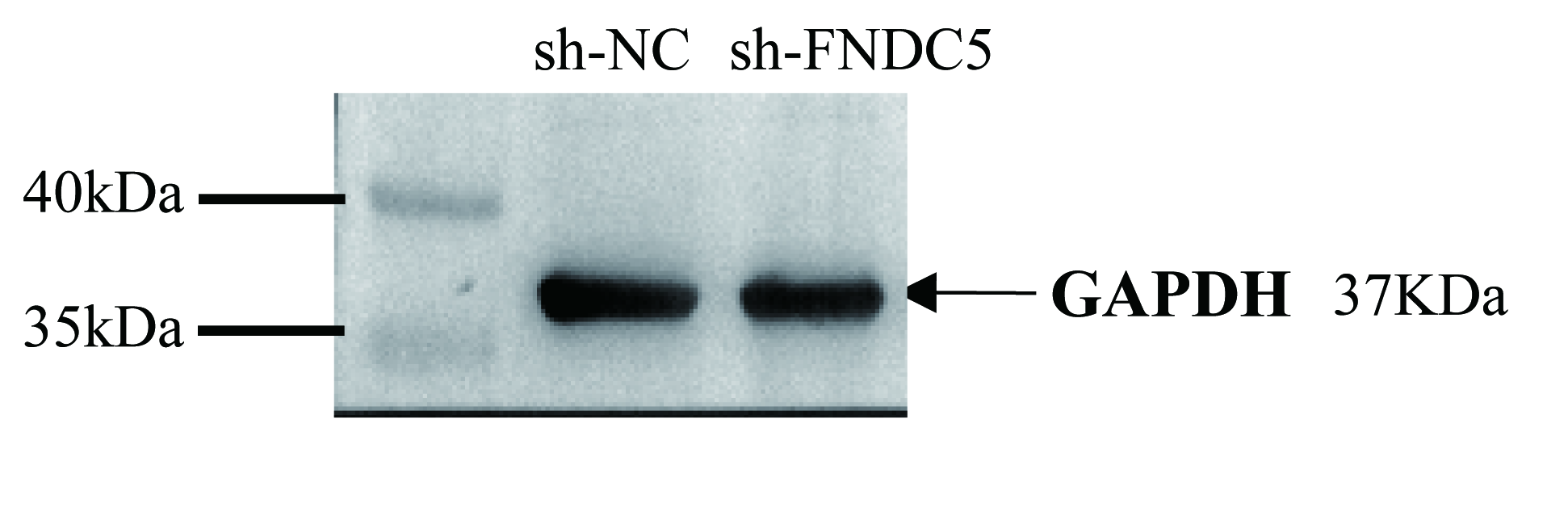

Supplement: Supplementary file 5 — Supplementary Material 5. [file 13395_2026_420_MOESM5_ESM.zip › Supplementary Material 5/Fig2/Fig2C/sh-FNDC5/GAPDH/GAPDH-3.tif]

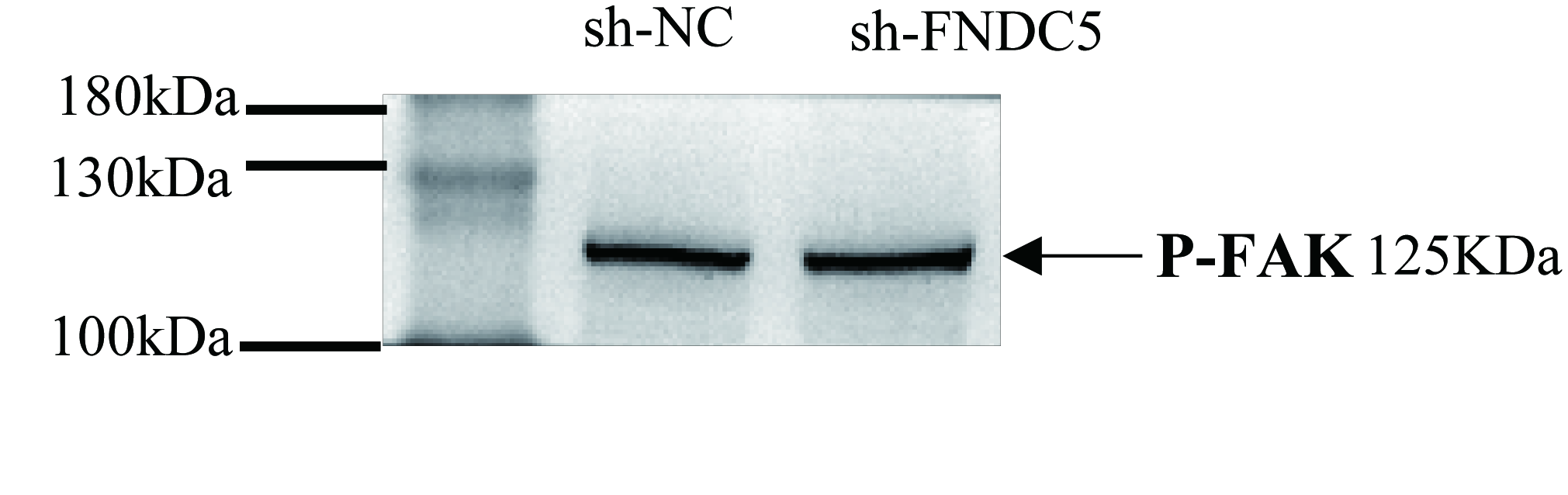

Supplement: Supplementary file 5 — Supplementary Material 5. [file 13395_2026_420_MOESM5_ESM.zip › Supplementary Material 5/Fig2/Fig2C/sh-FNDC5/P-FAK/P-FAK-1.tif]

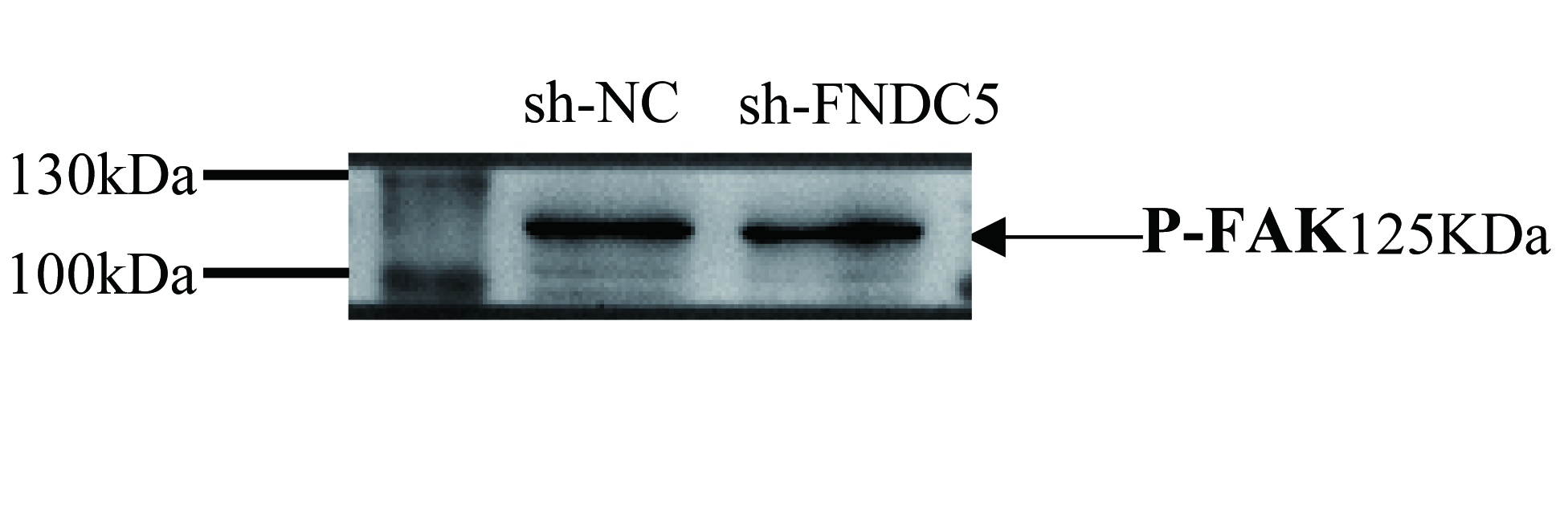

Supplement: Supplementary file 5 — Supplementary Material 5. [file 13395_2026_420_MOESM5_ESM.zip › Supplementary Material 5/Fig2/Fig2C/sh-FNDC5/P-FAK/P-FAK-2.tif]

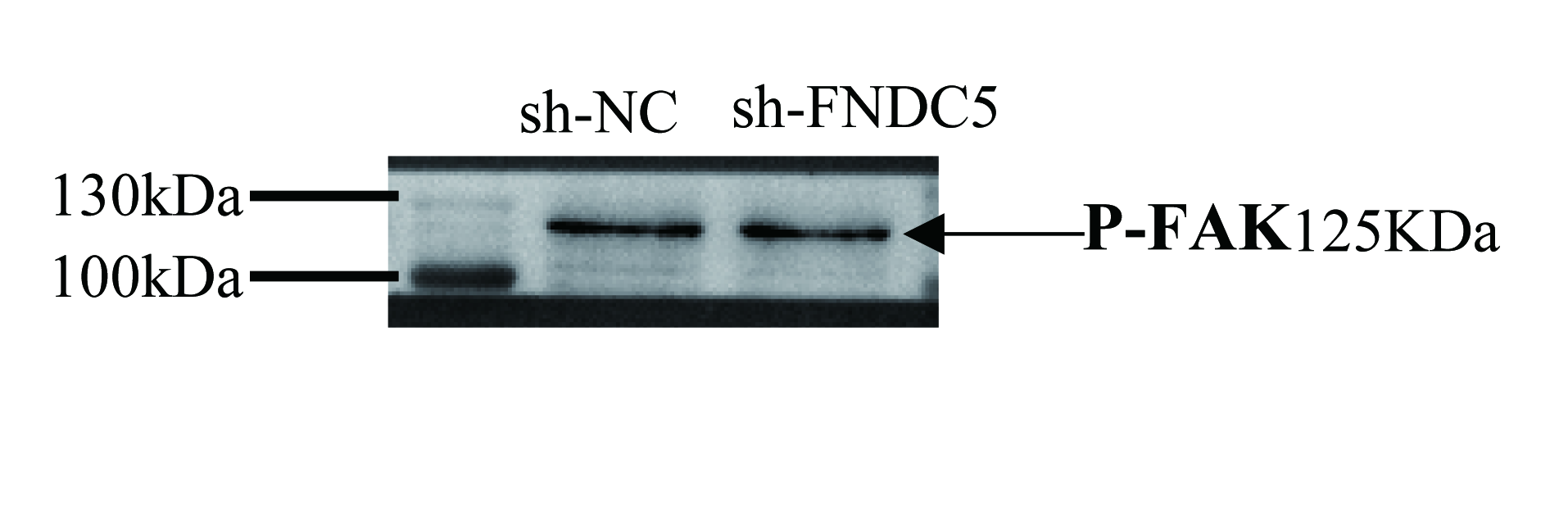

Supplement: Supplementary file 5 — Supplementary Material 5. [file 13395_2026_420_MOESM5_ESM.zip › Supplementary Material 5/Fig2/Fig2C/sh-FNDC5/P-FAK/P-FAK-3.tif]

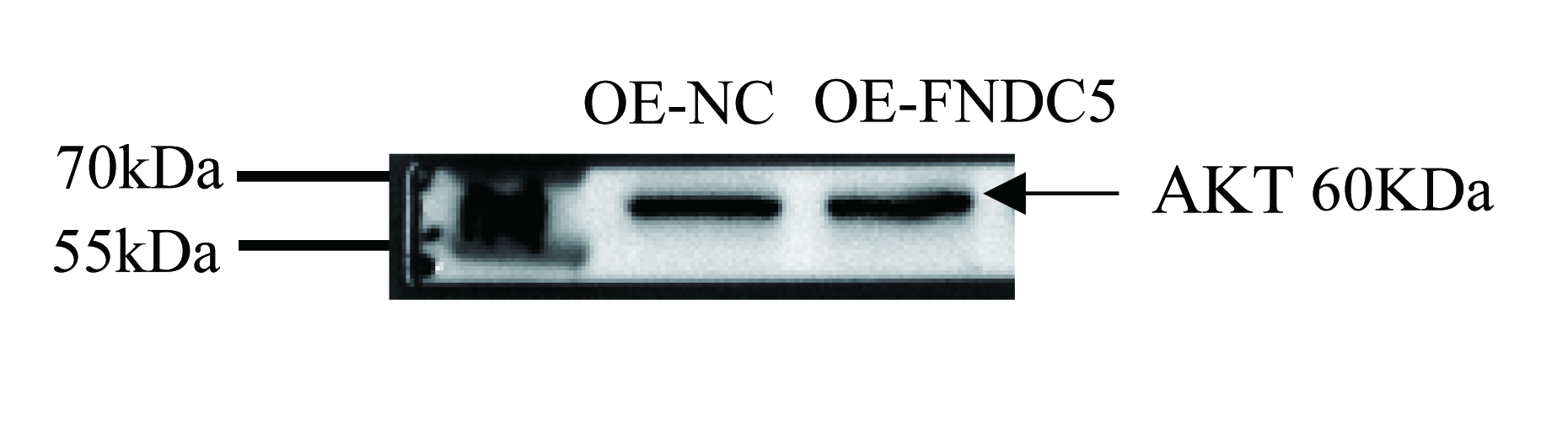

Supplement: Supplementary file 5 — Supplementary Material 5. [file 13395_2026_420_MOESM5_ESM.zip › Supplementary Material 5/Fig2/Fig2D/OE-FNDC5/AKT/AKT-1.tif]

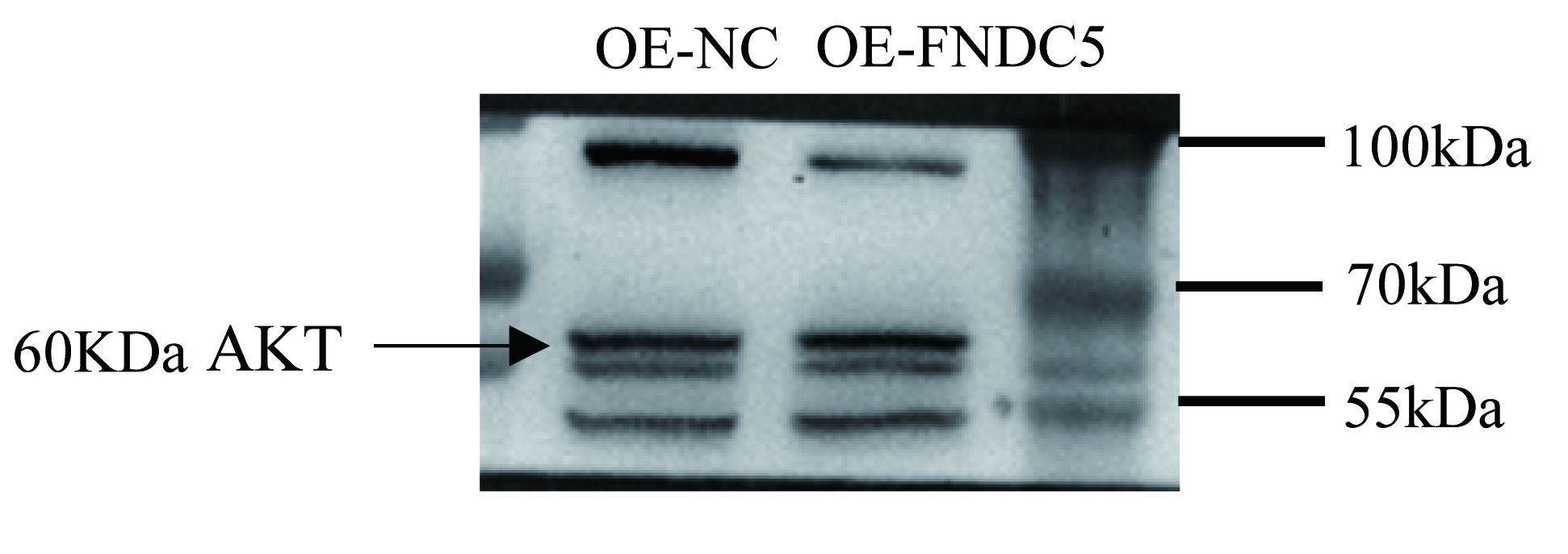

Supplement: Supplementary file 5 — Supplementary Material 5. [file 13395_2026_420_MOESM5_ESM.zip › Supplementary Material 5/Fig2/Fig2D/OE-FNDC5/AKT/AKT-2.tif]

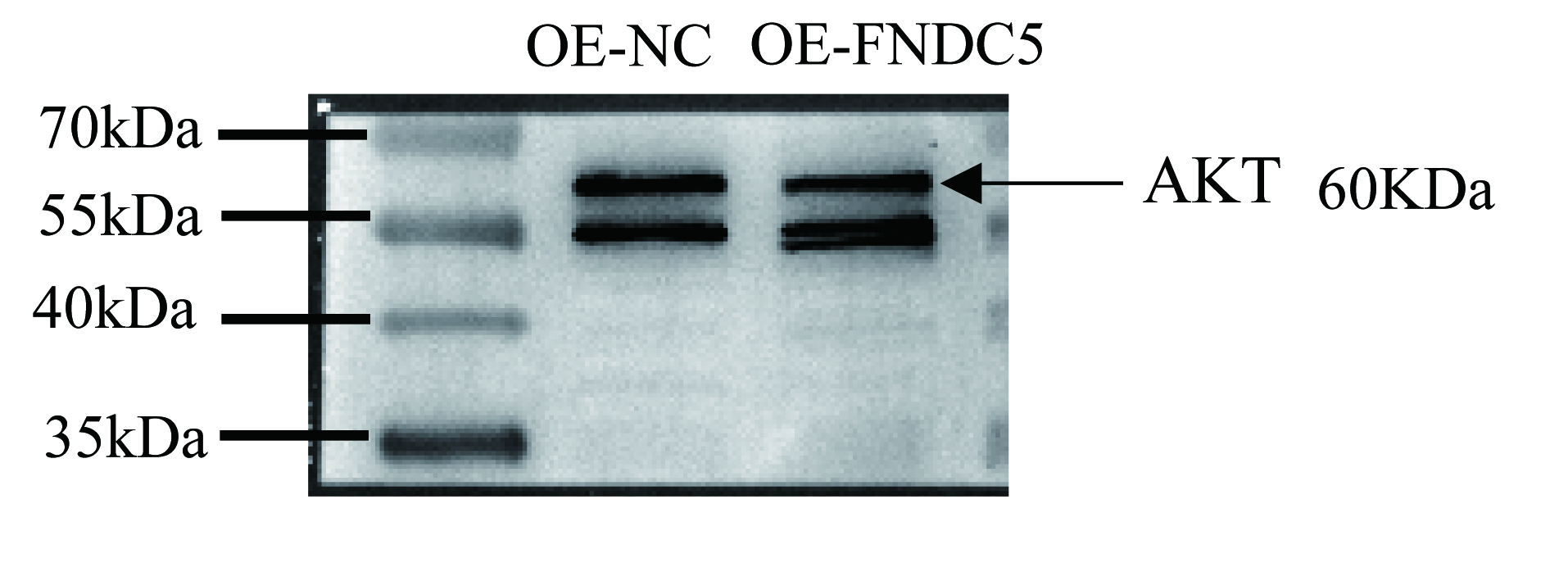

Supplement: Supplementary file 5 — Supplementary Material 5. [file 13395_2026_420_MOESM5_ESM.zip › Supplementary Material 5/Fig2/Fig2D/OE-FNDC5/AKT/AKT-3.tif]

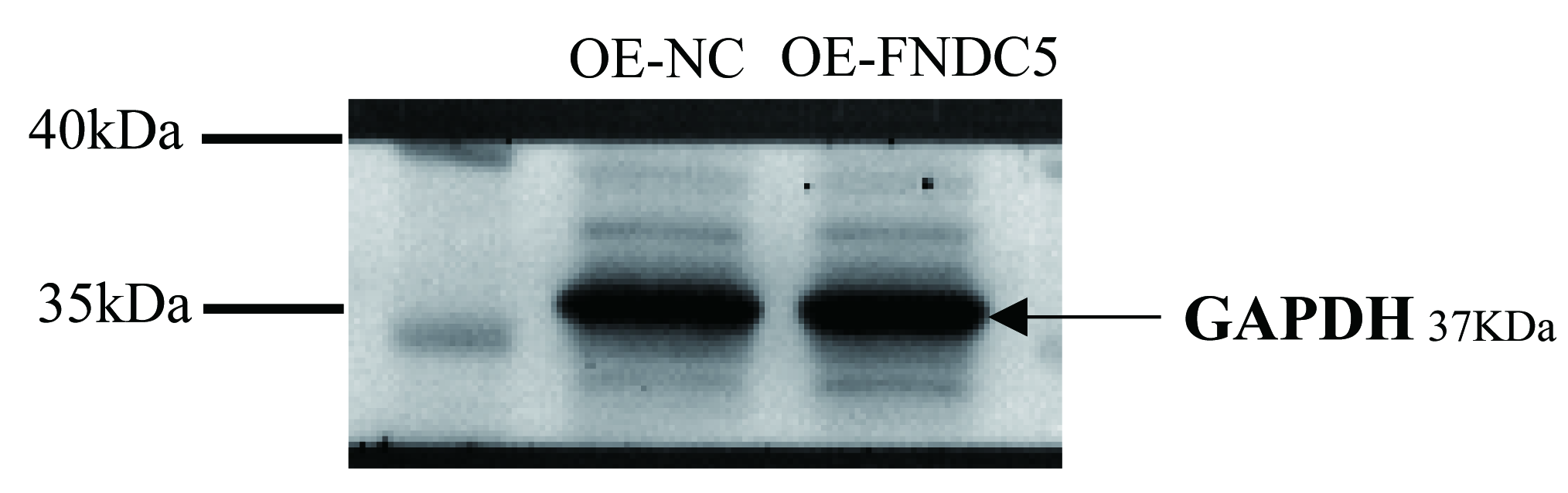

Supplement: Supplementary file 5 — Supplementary Material 5. [file 13395_2026_420_MOESM5_ESM.zip › Supplementary Material 5/Fig2/Fig2D/OE-FNDC5/GAPDH/GAPDH-1.tif]

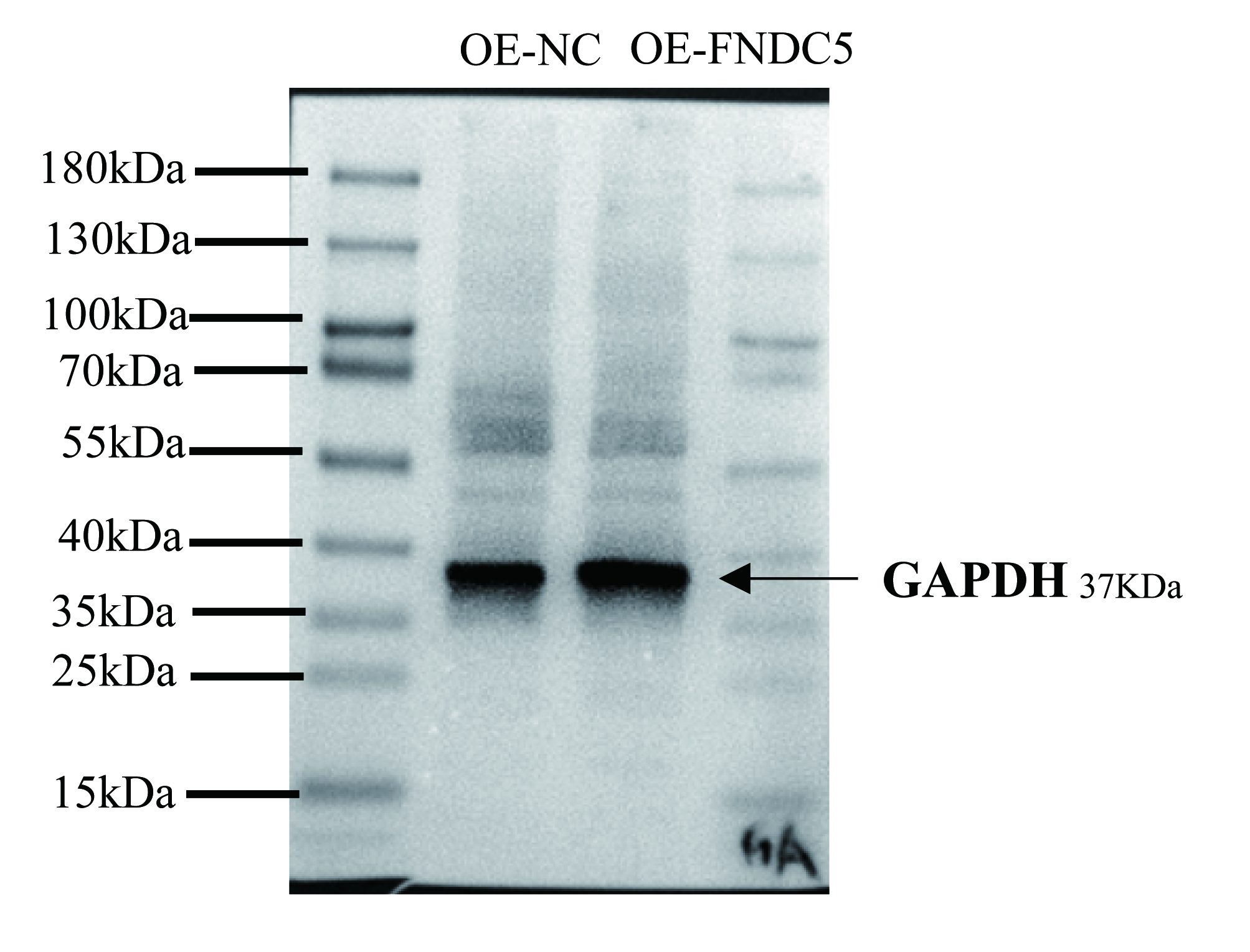

Supplement: Supplementary file 5 — Supplementary Material 5. [file 13395_2026_420_MOESM5_ESM.zip › Supplementary Material 5/Fig2/Fig2D/OE-FNDC5/GAPDH/GAPDH-2.tif]

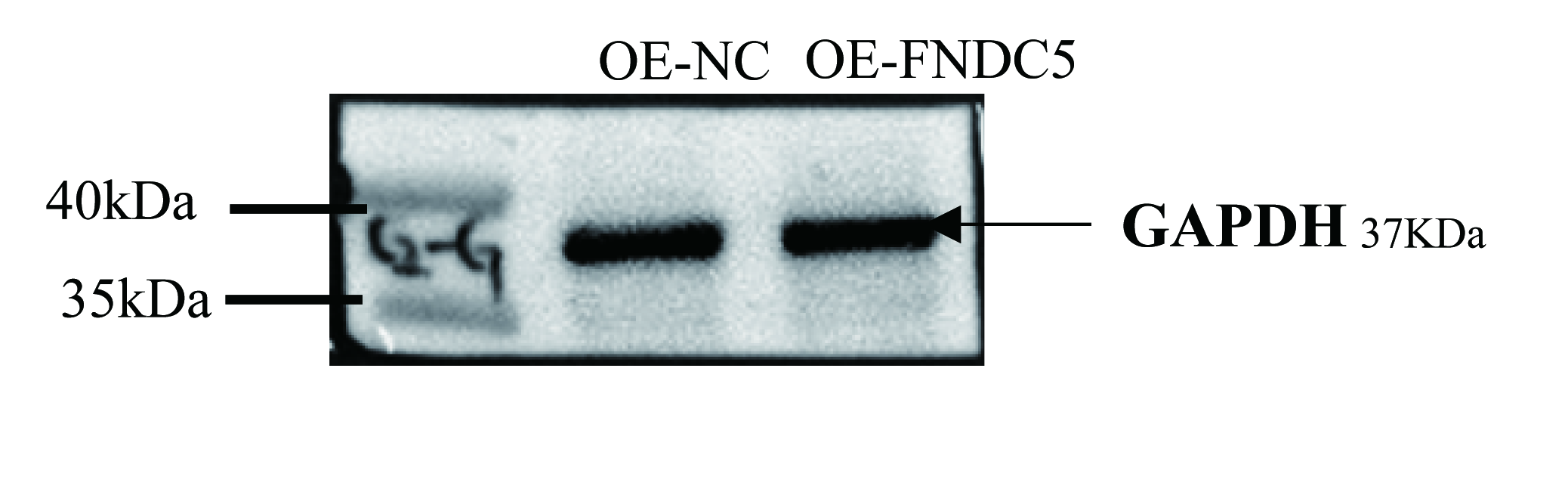

Supplement: Supplementary file 5 — Supplementary Material 5. [file 13395_2026_420_MOESM5_ESM.zip › Supplementary Material 5/Fig2/Fig2D/OE-FNDC5/GAPDH/GAPDH-3.tif]

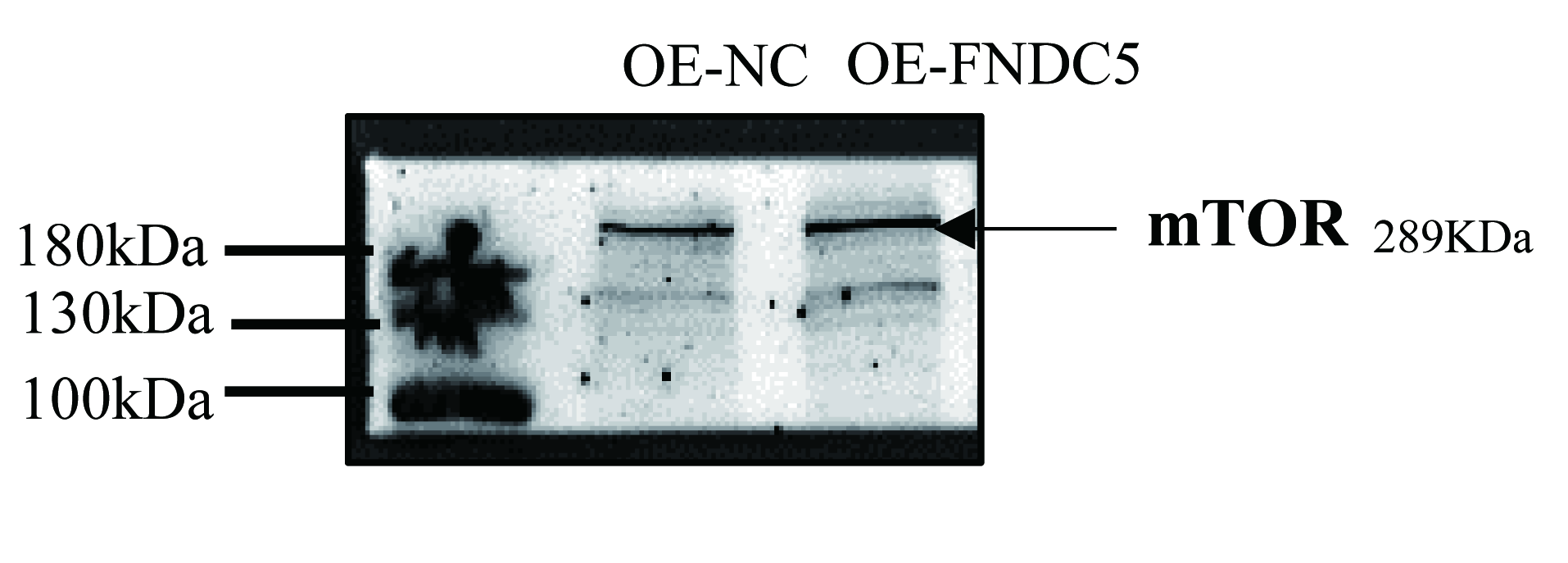

Supplement: Supplementary file 5 — Supplementary Material 5. [file 13395_2026_420_MOESM5_ESM.zip › Supplementary Material 5/Fig2/Fig2D/OE-FNDC5/mTOR/mTOR-1.tif]

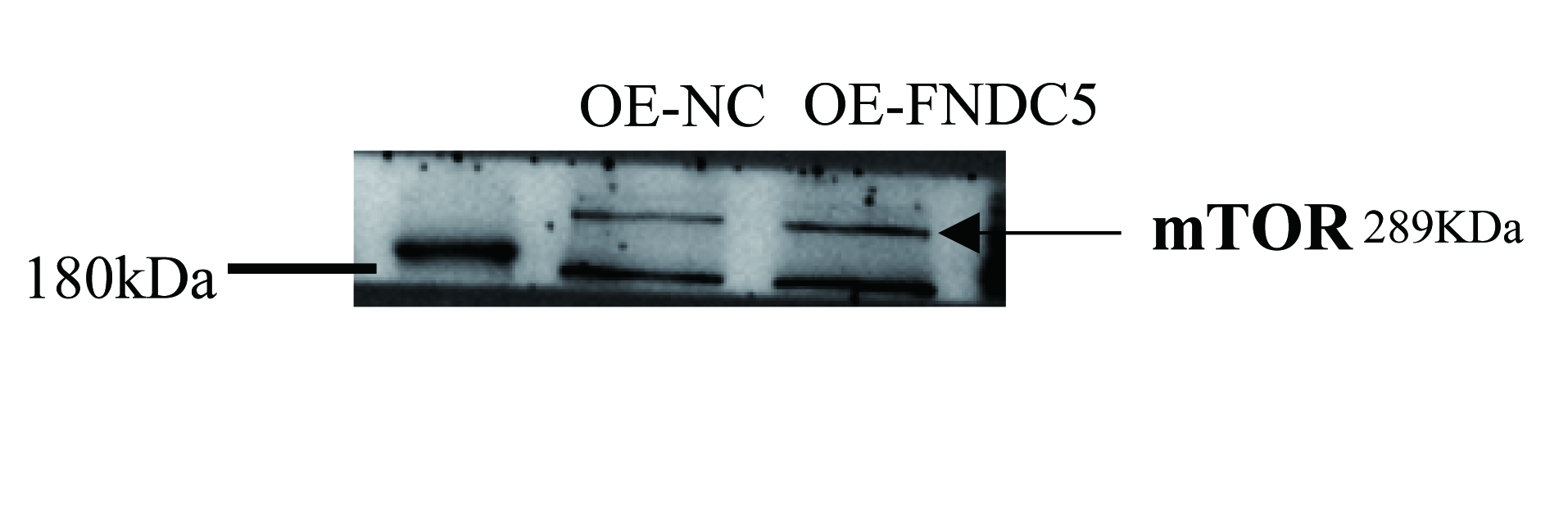

Supplement: Supplementary file 5 — Supplementary Material 5. [file 13395_2026_420_MOESM5_ESM.zip › Supplementary Material 5/Fig2/Fig2D/OE-FNDC5/mTOR/mTOR-2.tif]

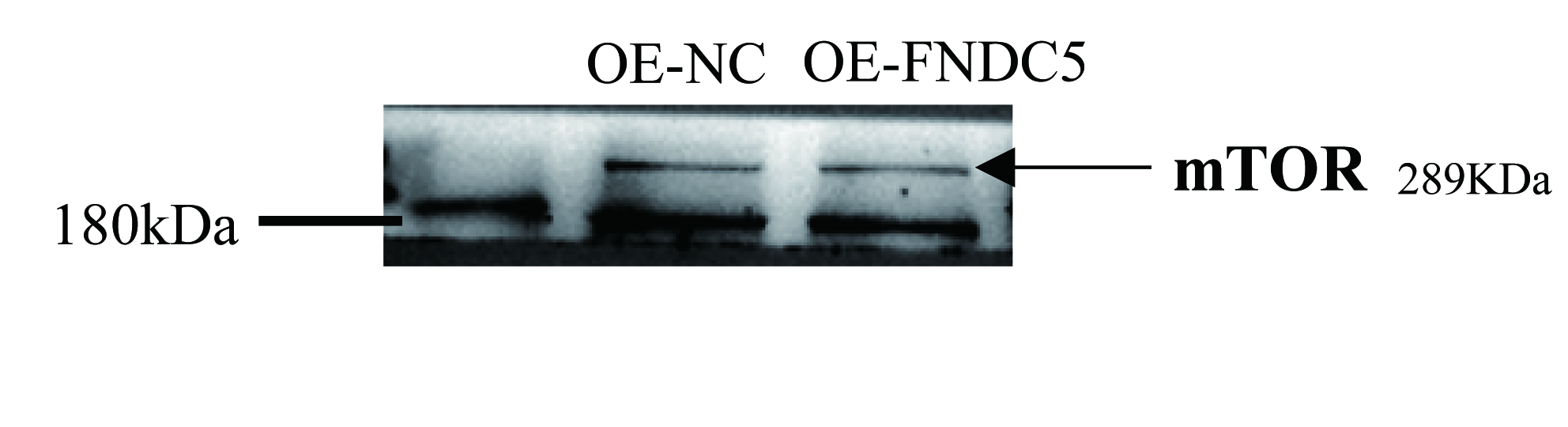

Supplement: Supplementary file 5 — Supplementary Material 5. [file 13395_2026_420_MOESM5_ESM.zip › Supplementary Material 5/Fig2/Fig2D/OE-FNDC5/mTOR/mTOR-3.tif]

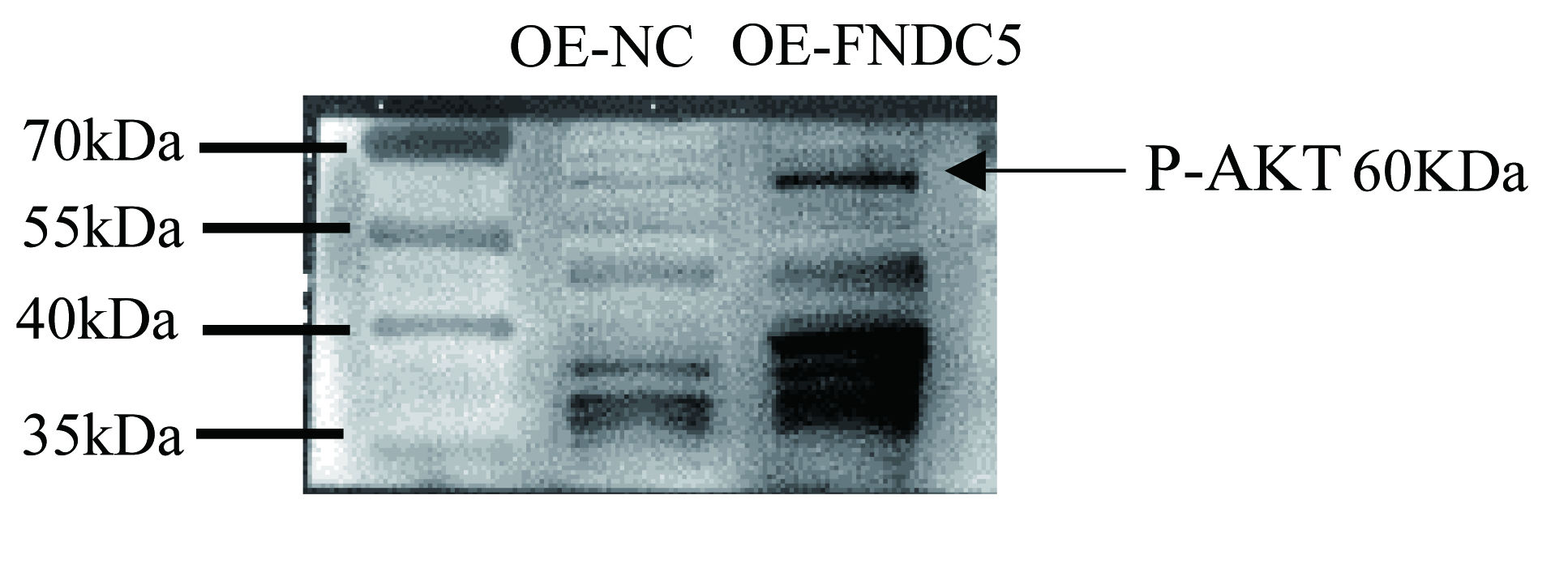

Supplement: Supplementary file 5 — Supplementary Material 5. [file 13395_2026_420_MOESM5_ESM.zip › Supplementary Material 5/Fig2/Fig2D/OE-FNDC5/P-AKT/P-AKT-1.tif]

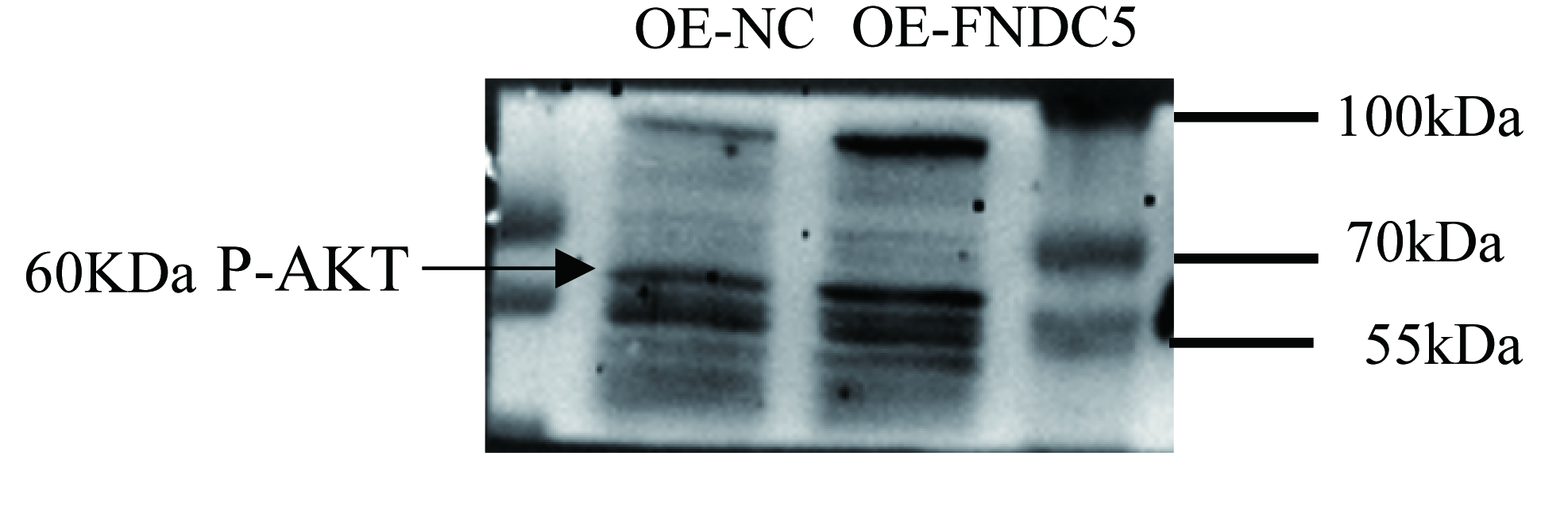

Supplement: Supplementary file 5 — Supplementary Material 5. [file 13395_2026_420_MOESM5_ESM.zip › Supplementary Material 5/Fig2/Fig2D/OE-FNDC5/P-AKT/P-AKT-2.tif]

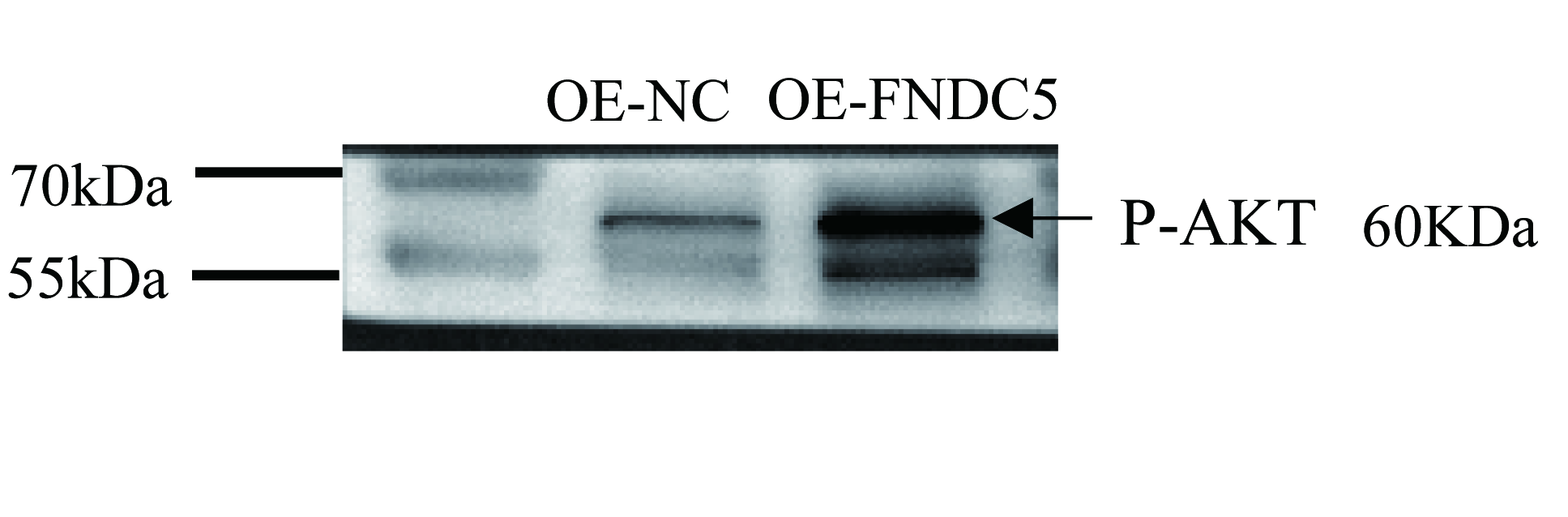

Supplement: Supplementary file 5 — Supplementary Material 5. [file 13395_2026_420_MOESM5_ESM.zip › Supplementary Material 5/Fig2/Fig2D/OE-FNDC5/P-AKT/P-AKT-3.tif]

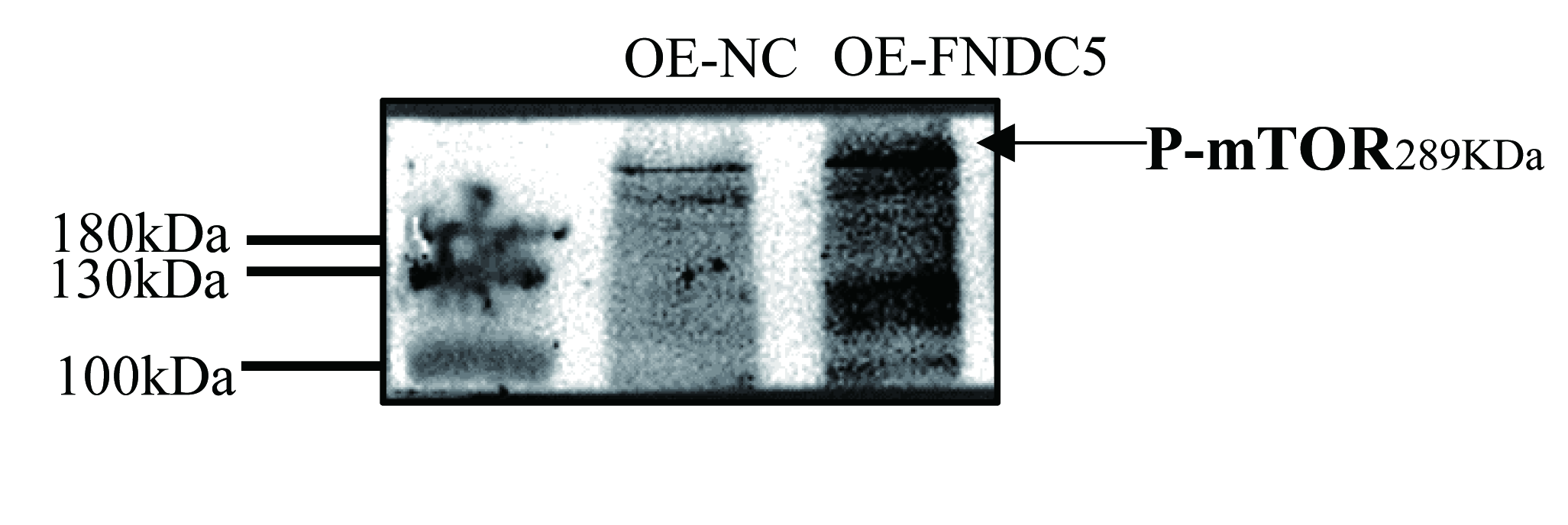

Supplement: Supplementary file 5 — Supplementary Material 5. [file 13395_2026_420_MOESM5_ESM.zip › Supplementary Material 5/Fig2/Fig2D/OE-FNDC5/P-mTOR/P-mTOR-1.tif]

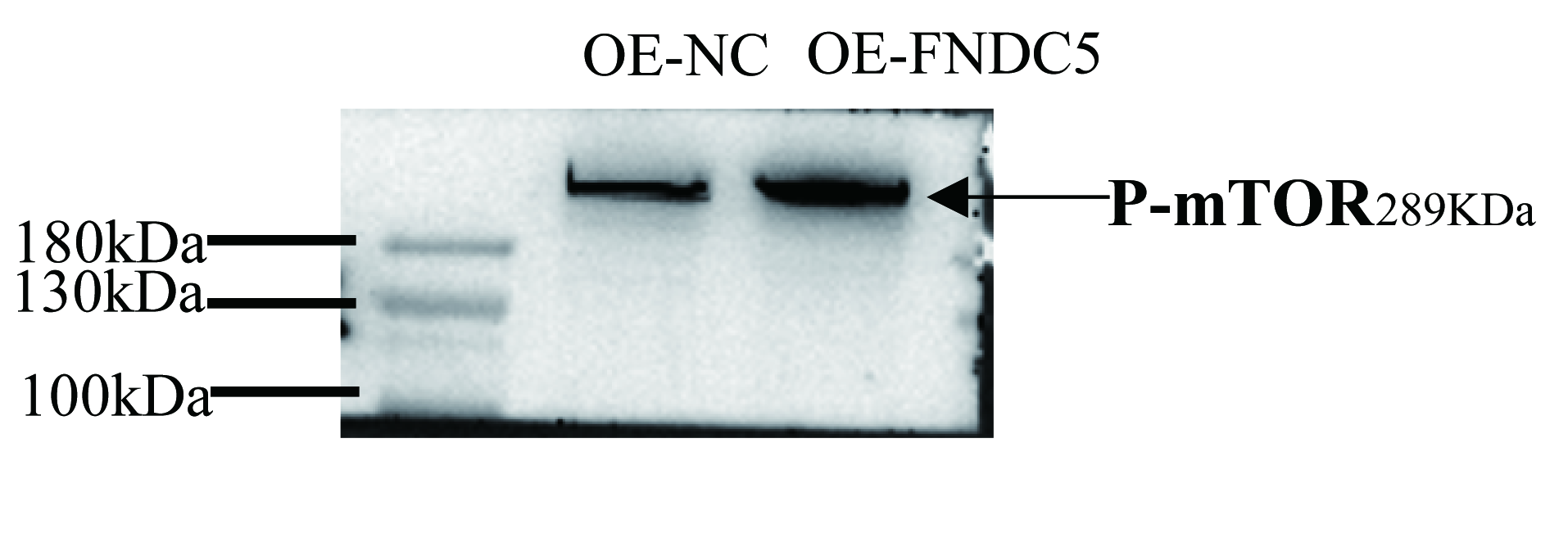

Supplement: Supplementary file 5 — Supplementary Material 5. [file 13395_2026_420_MOESM5_ESM.zip › Supplementary Material 5/Fig2/Fig2D/OE-FNDC5/P-mTOR/P-mTOR-2.tif]

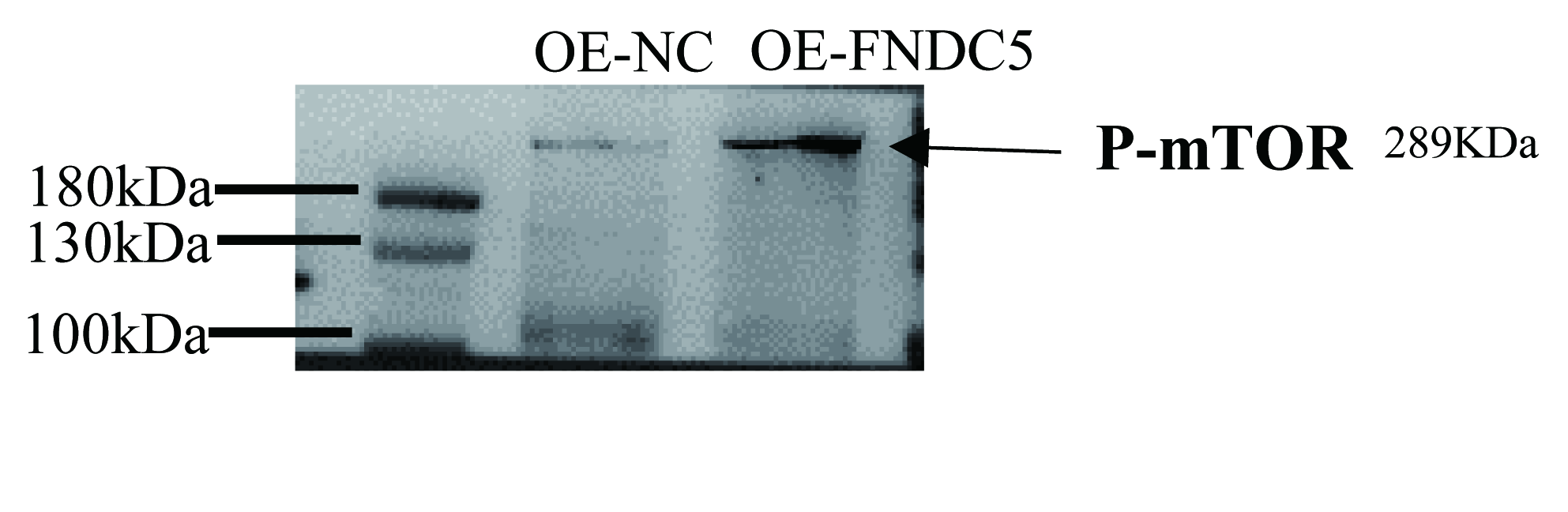

Supplement: Supplementary file 5 — Supplementary Material 5. [file 13395_2026_420_MOESM5_ESM.zip › Supplementary Material 5/Fig2/Fig2D/OE-FNDC5/P-mTOR/P-mTOR-3.tif]

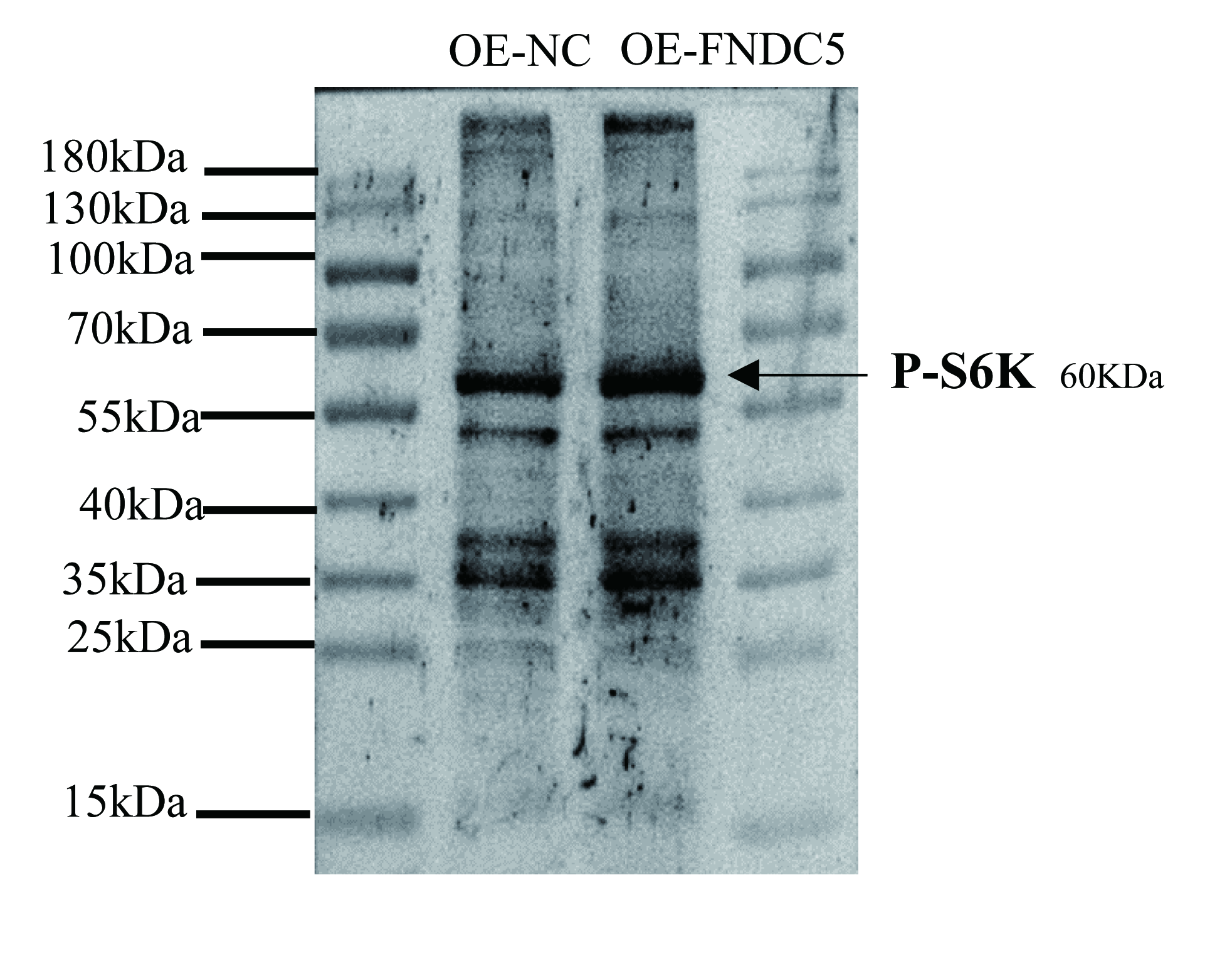

Supplement: Supplementary file 5 — Supplementary Material 5. [file 13395_2026_420_MOESM5_ESM.zip › Supplementary Material 5/Fig2/Fig2D/OE-FNDC5/P-S6K/P-S6K-1.tif]

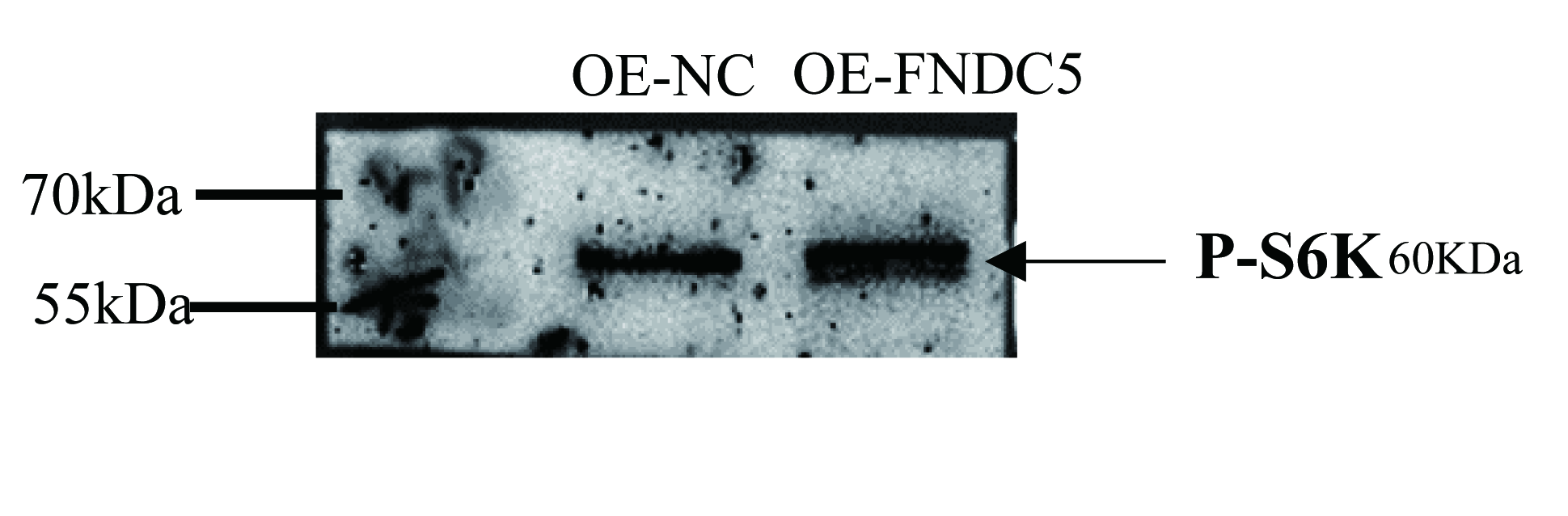

Supplement: Supplementary file 5 — Supplementary Material 5. [file 13395_2026_420_MOESM5_ESM.zip › Supplementary Material 5/Fig2/Fig2D/OE-FNDC5/P-S6K/P-S6K-2.tif]

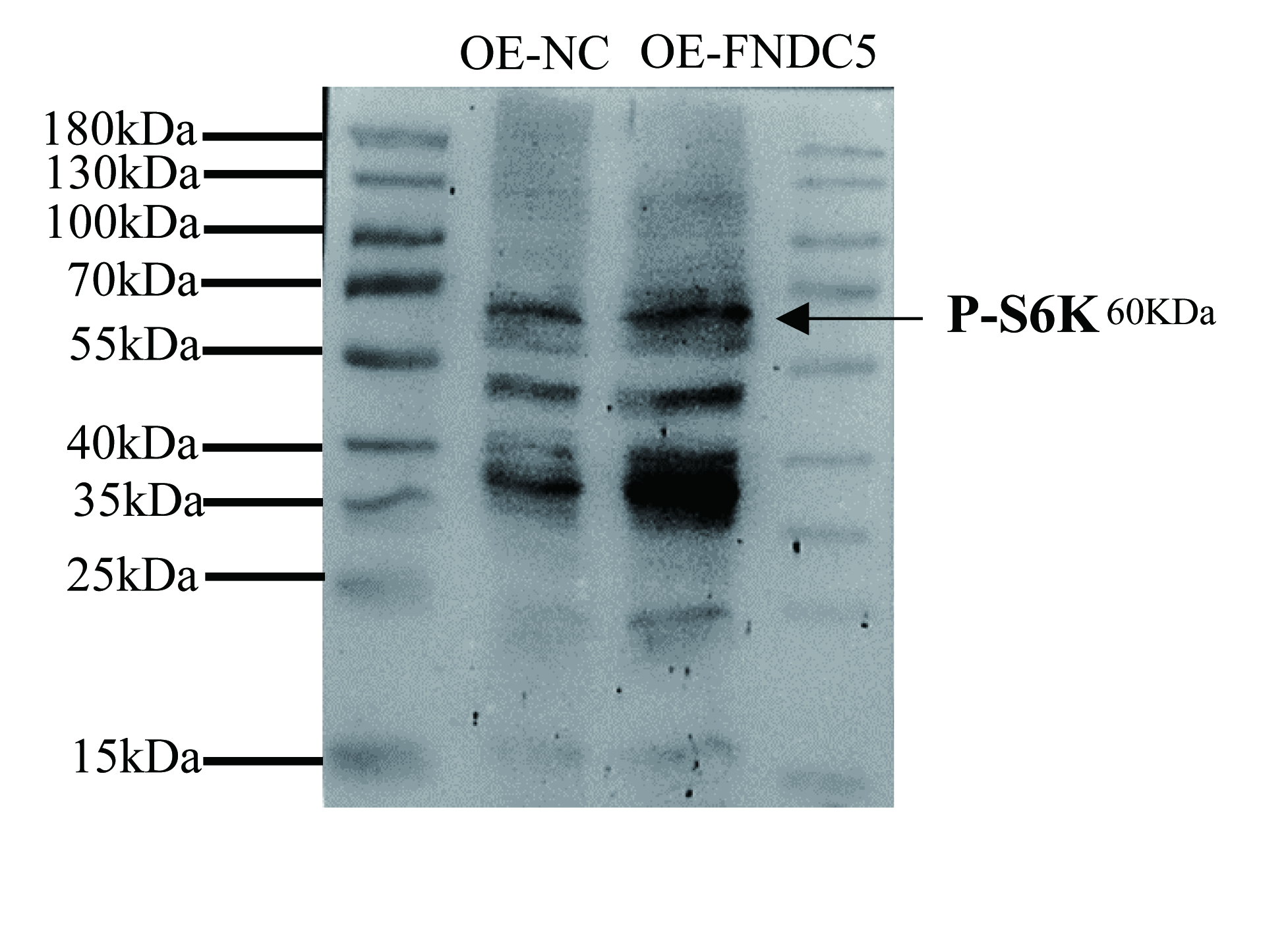

Supplement: Supplementary file 5 — Supplementary Material 5. [file 13395_2026_420_MOESM5_ESM.zip › Supplementary Material 5/Fig2/Fig2D/OE-FNDC5/P-S6K/P-S6K-3.tif]

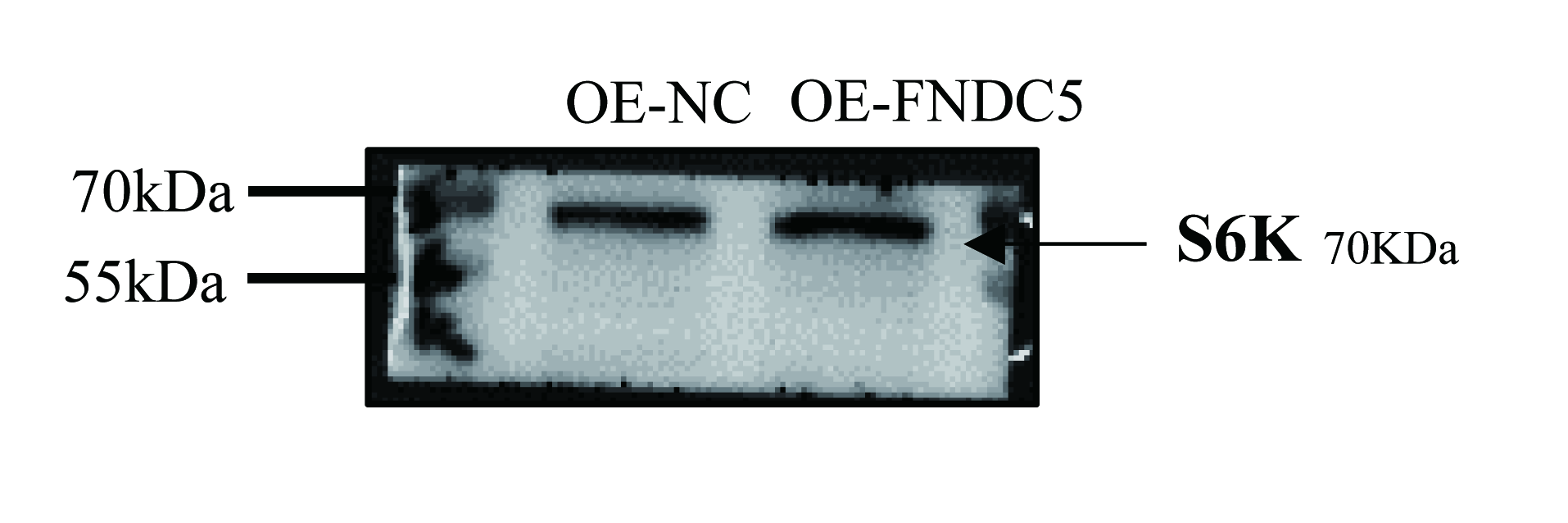

Supplement: Supplementary file 5 — Supplementary Material 5. [file 13395_2026_420_MOESM5_ESM.zip › Supplementary Material 5/Fig2/Fig2D/OE-FNDC5/S6K/S6K-1.tif]

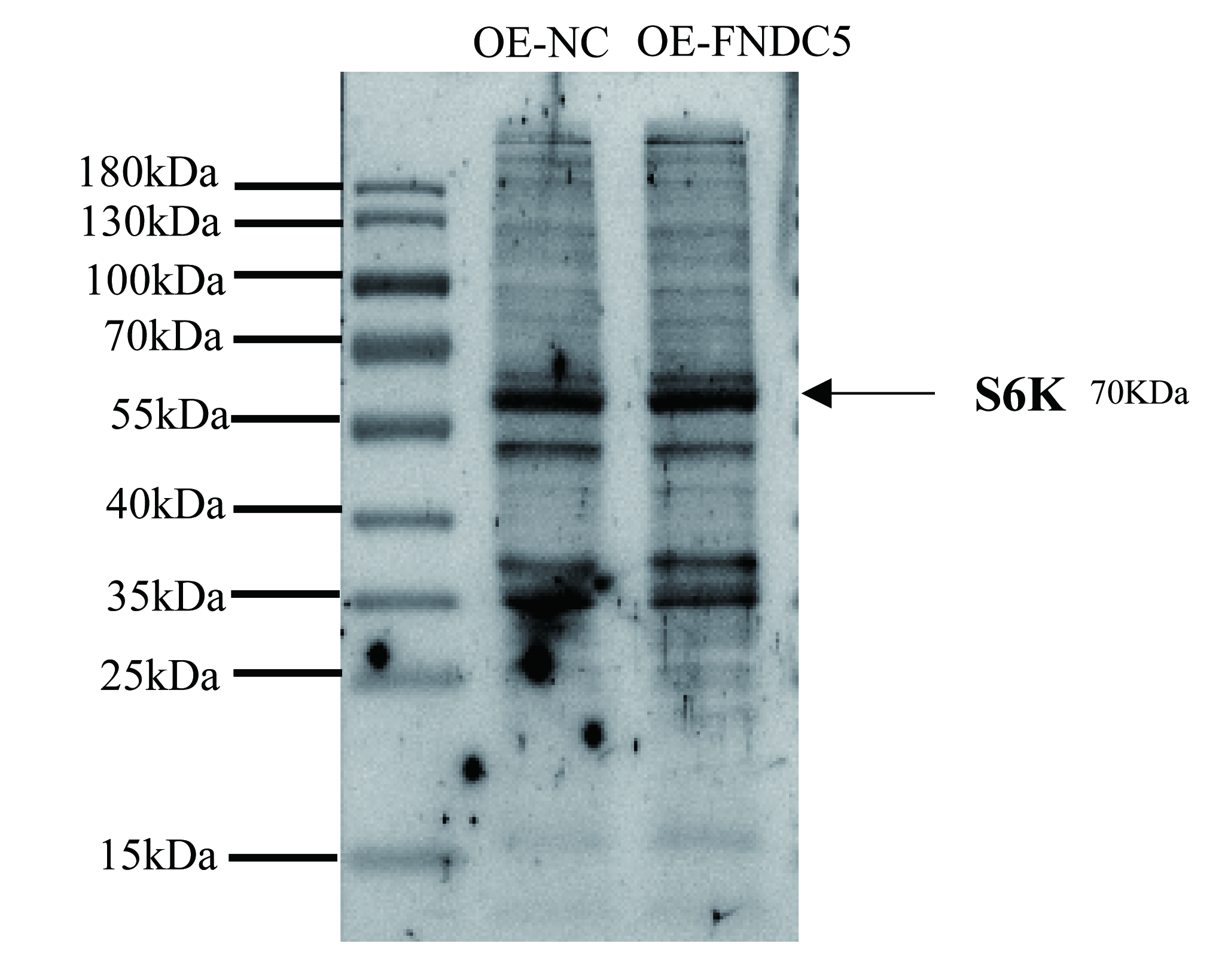

Supplement: Supplementary file 5 — Supplementary Material 5. [file 13395_2026_420_MOESM5_ESM.zip › Supplementary Material 5/Fig2/Fig2D/OE-FNDC5/S6K/S6K-2.tif]

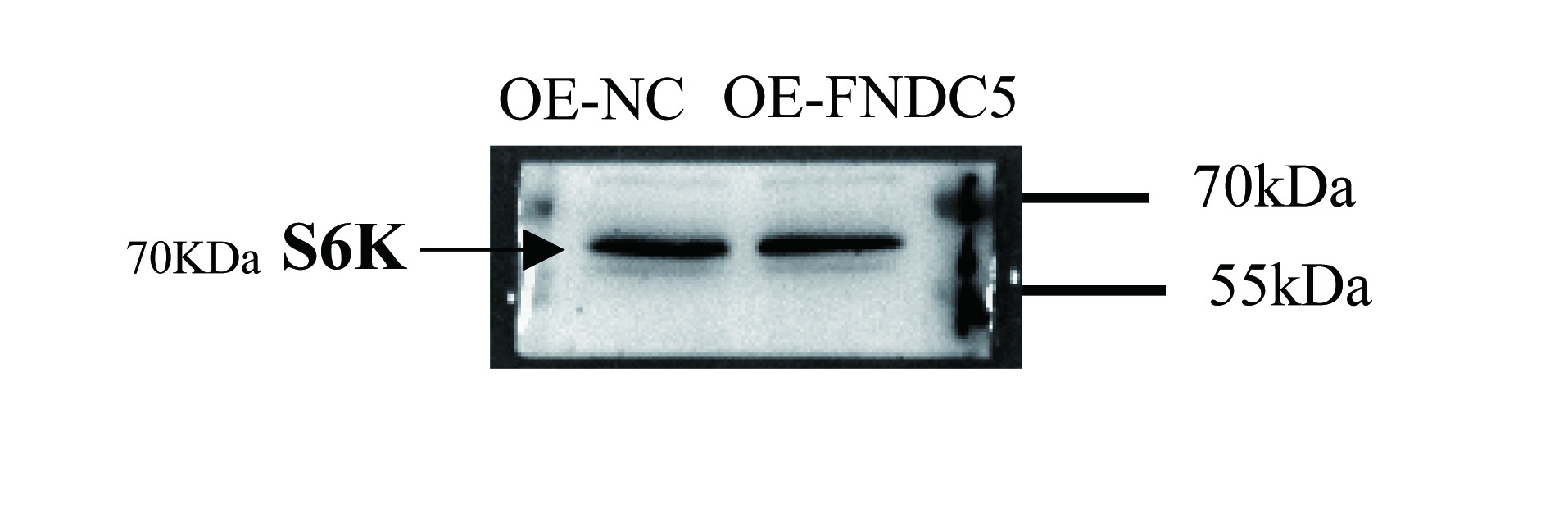

Supplement: Supplementary file 5 — Supplementary Material 5. [file 13395_2026_420_MOESM5_ESM.zip › Supplementary Material 5/Fig2/Fig2D/OE-FNDC5/S6K/S6K-3.tif]

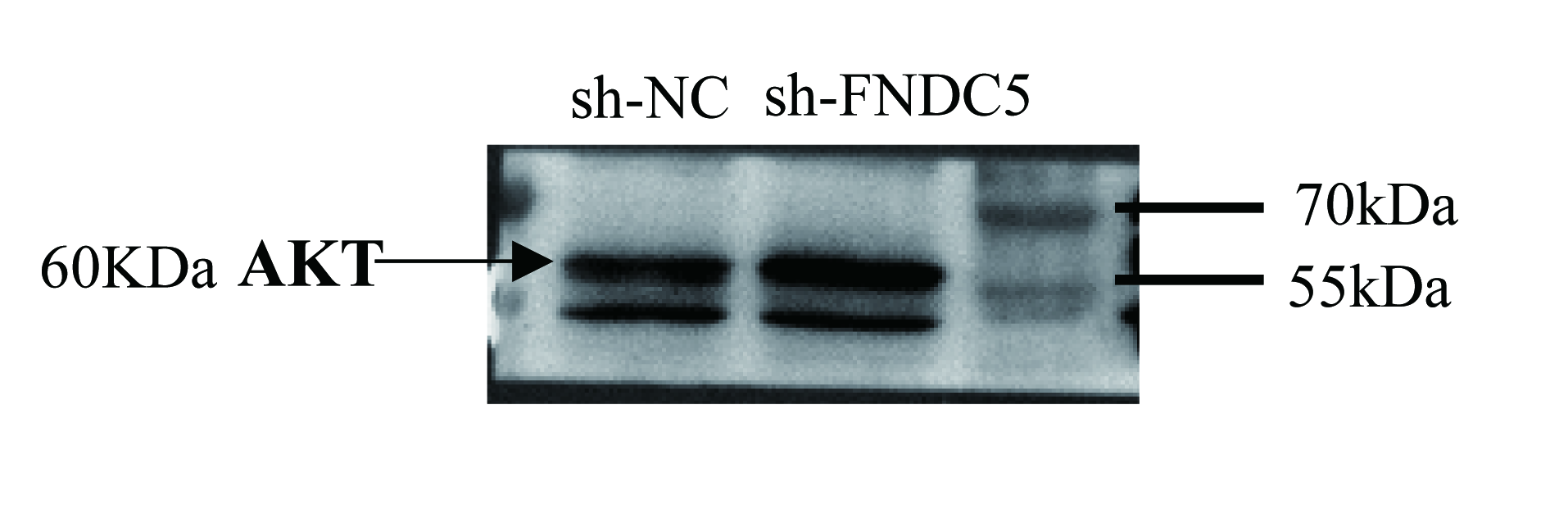

Supplement: Supplementary file 5 — Supplementary Material 5. [file 13395_2026_420_MOESM5_ESM.zip › Supplementary Material 5/Fig2/Fig2D/sh-FNDC5/AKT/AKT-1.tif]

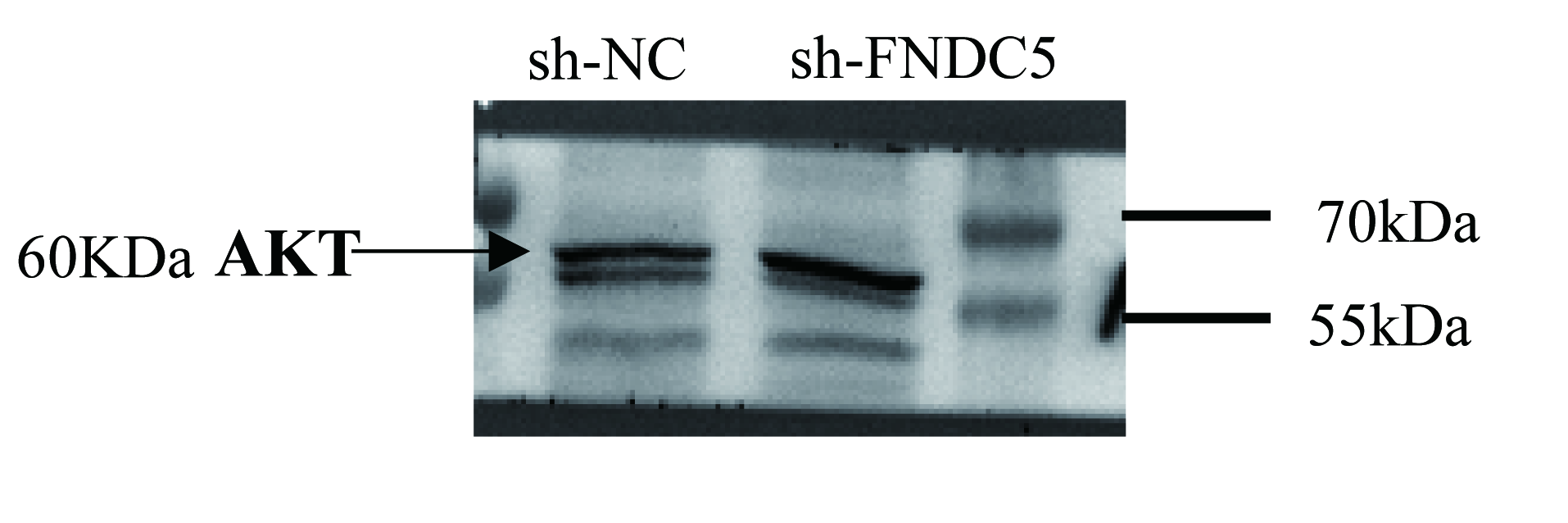

Supplement: Supplementary file 5 — Supplementary Material 5. [file 13395_2026_420_MOESM5_ESM.zip › Supplementary Material 5/Fig2/Fig2D/sh-FNDC5/AKT/AKT-2.tif]

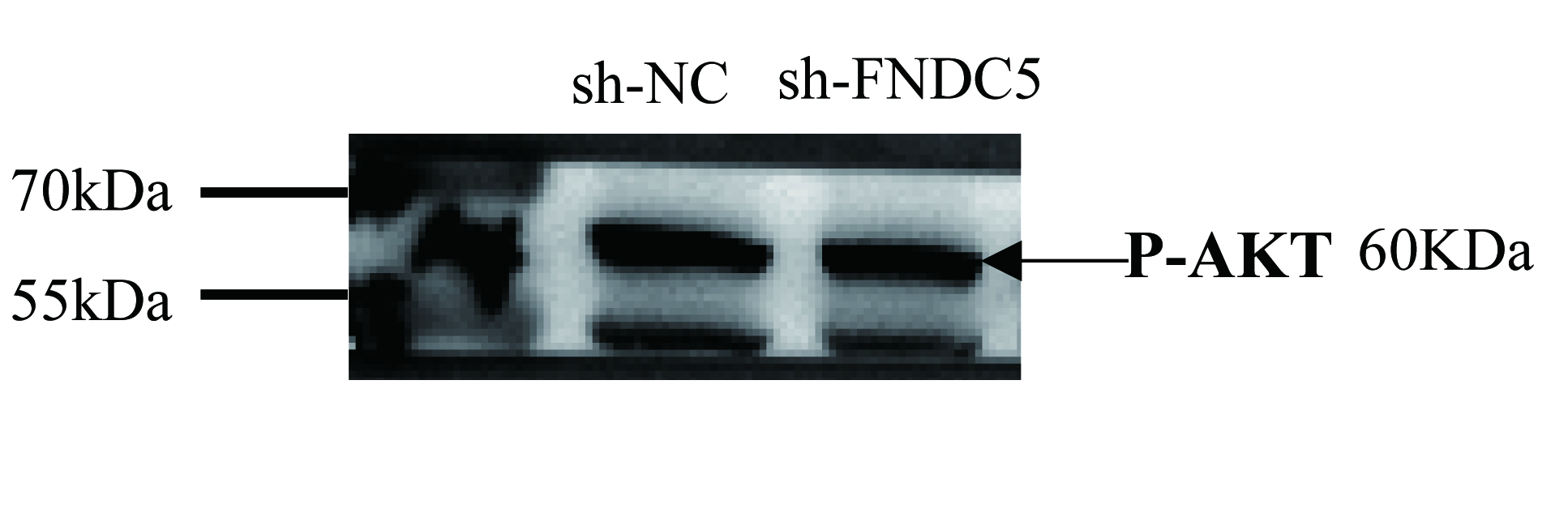

Supplement: Supplementary file 5 — Supplementary Material 5. [file 13395_2026_420_MOESM5_ESM.zip › Supplementary Material 5/Fig2/Fig2D/sh-FNDC5/AKT/AKT-3.tif]
