# Supplementary figures and images for: Integrin αVβ5 regulates myoblast proliferation and differentiation in sarcopenia mice treated with FNDC5 gene delivery: Original article (part 2 of 2)
Source: Skelet Muscle. 2026 Mar 17;16:28. doi: 10.1186/s13395-026-00420-x (PMC13347998; doi:10.1186/s13395-026-00420-x)

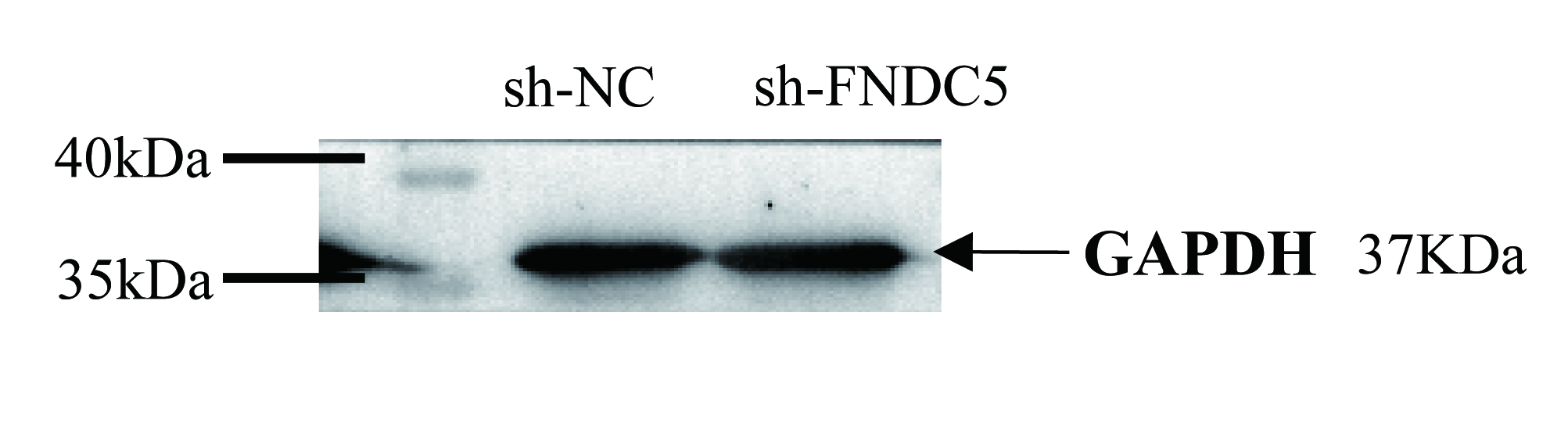

Supplement: Supplementary file 5 — Supplementary Material 5. [file 13395_2026_420_MOESM5_ESM.zip › Supplementary Material 5/Fig2/Fig2D/sh-FNDC5/GAPDH/GAPDH-1.tif]

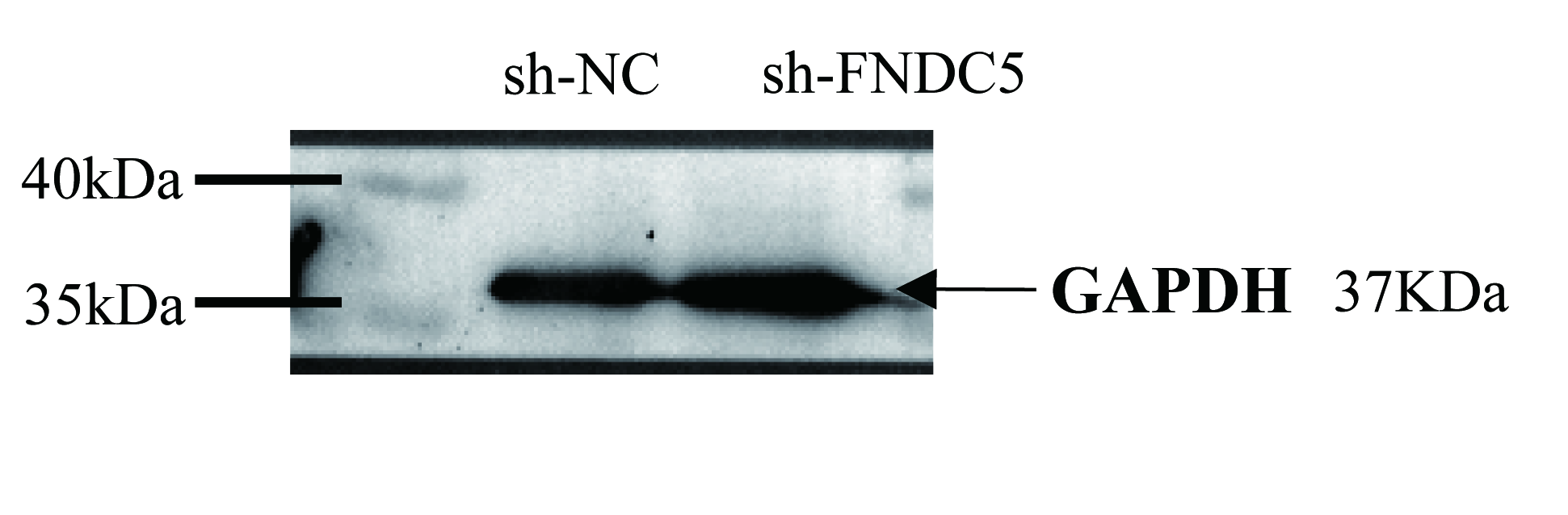

Supplement: Supplementary file 5 — Supplementary Material 5. [file 13395_2026_420_MOESM5_ESM.zip › Supplementary Material 5/Fig2/Fig2D/sh-FNDC5/GAPDH/GAPDH-2.tif]

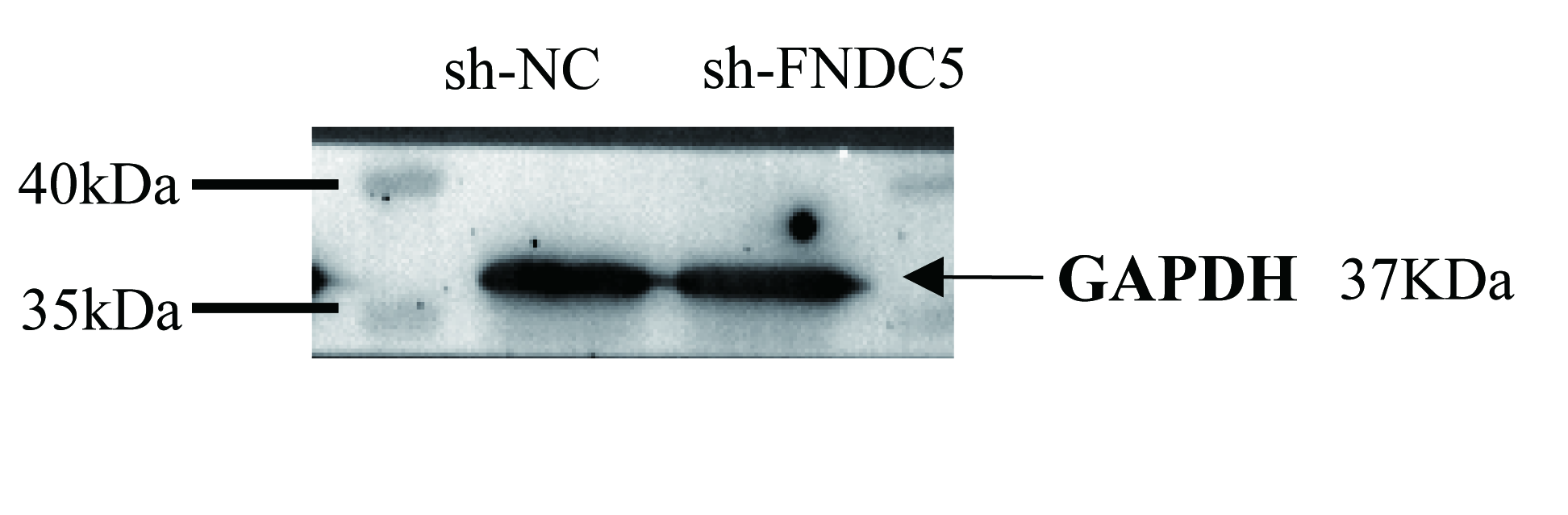

Supplement: Supplementary file 5 — Supplementary Material 5. [file 13395_2026_420_MOESM5_ESM.zip › Supplementary Material 5/Fig2/Fig2D/sh-FNDC5/GAPDH/GAPDH-3.tif]

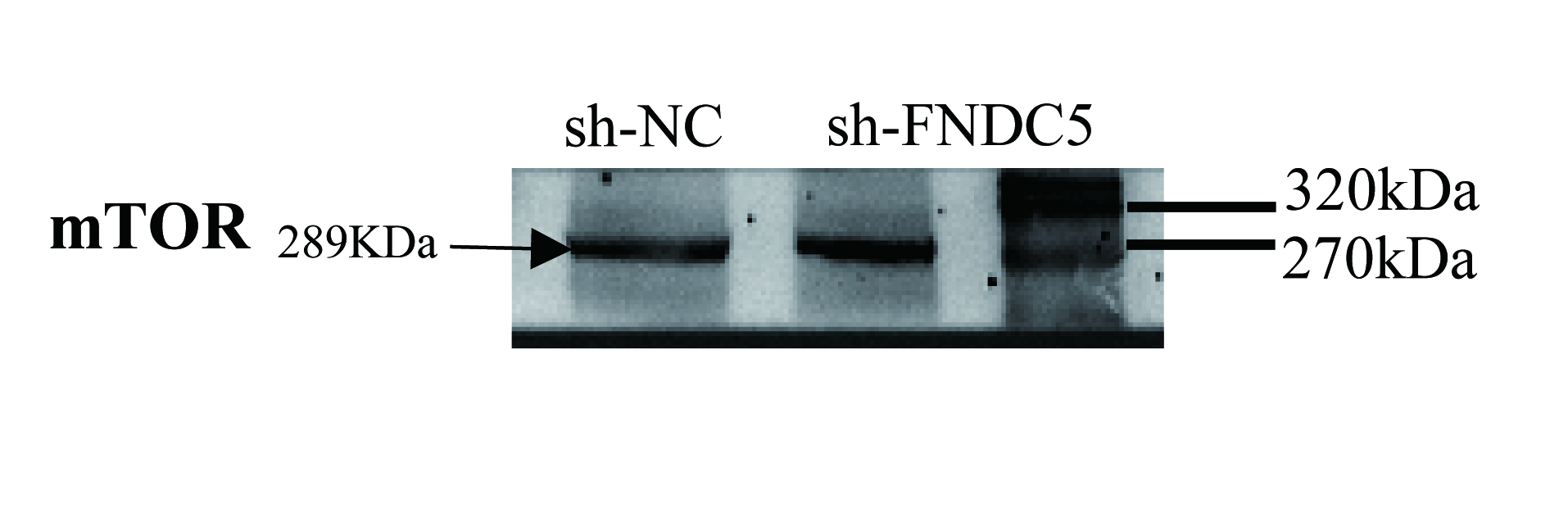

Supplement: Supplementary file 5 — Supplementary Material 5. [file 13395_2026_420_MOESM5_ESM.zip › Supplementary Material 5/Fig2/Fig2D/sh-FNDC5/mTOR/mTOR-1.tif]

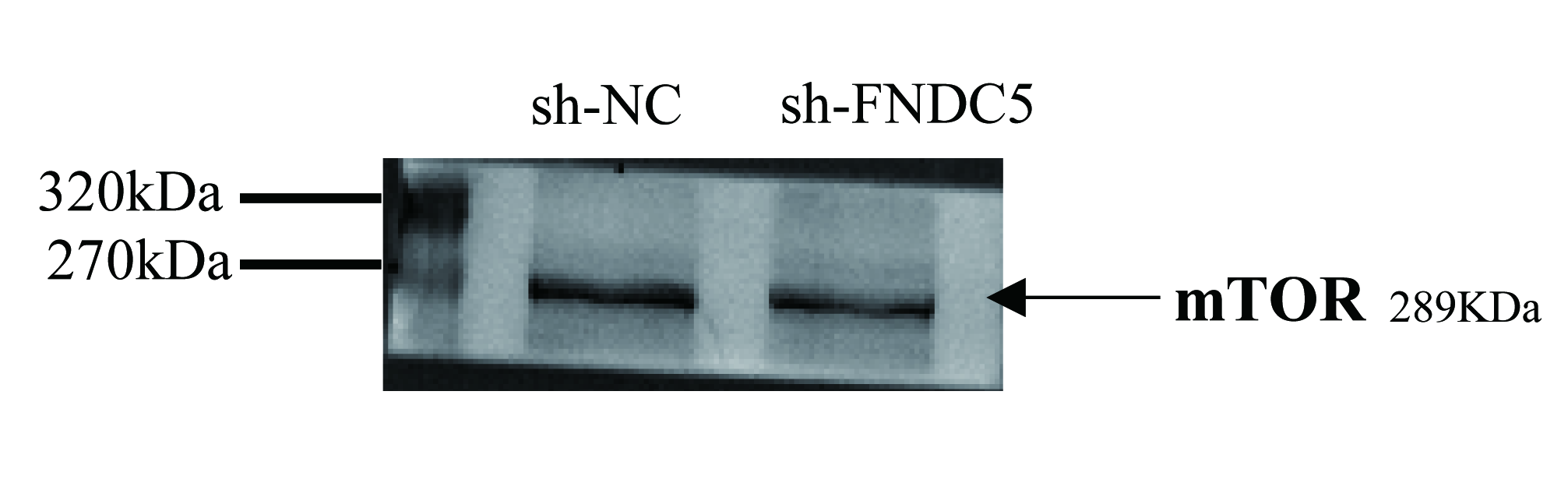

Supplement: Supplementary file 5 — Supplementary Material 5. [file 13395_2026_420_MOESM5_ESM.zip › Supplementary Material 5/Fig2/Fig2D/sh-FNDC5/mTOR/mTOR-2.tif]

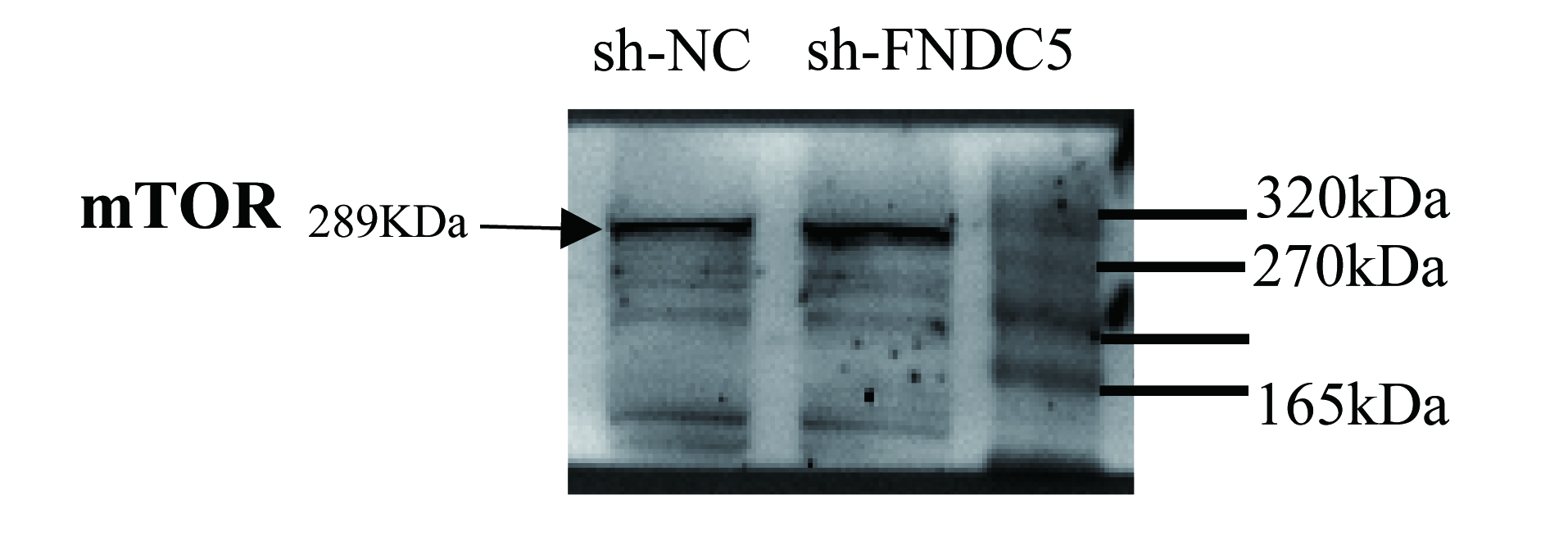

Supplement: Supplementary file 5 — Supplementary Material 5. [file 13395_2026_420_MOESM5_ESM.zip › Supplementary Material 5/Fig2/Fig2D/sh-FNDC5/mTOR/mTOR-3.tif]

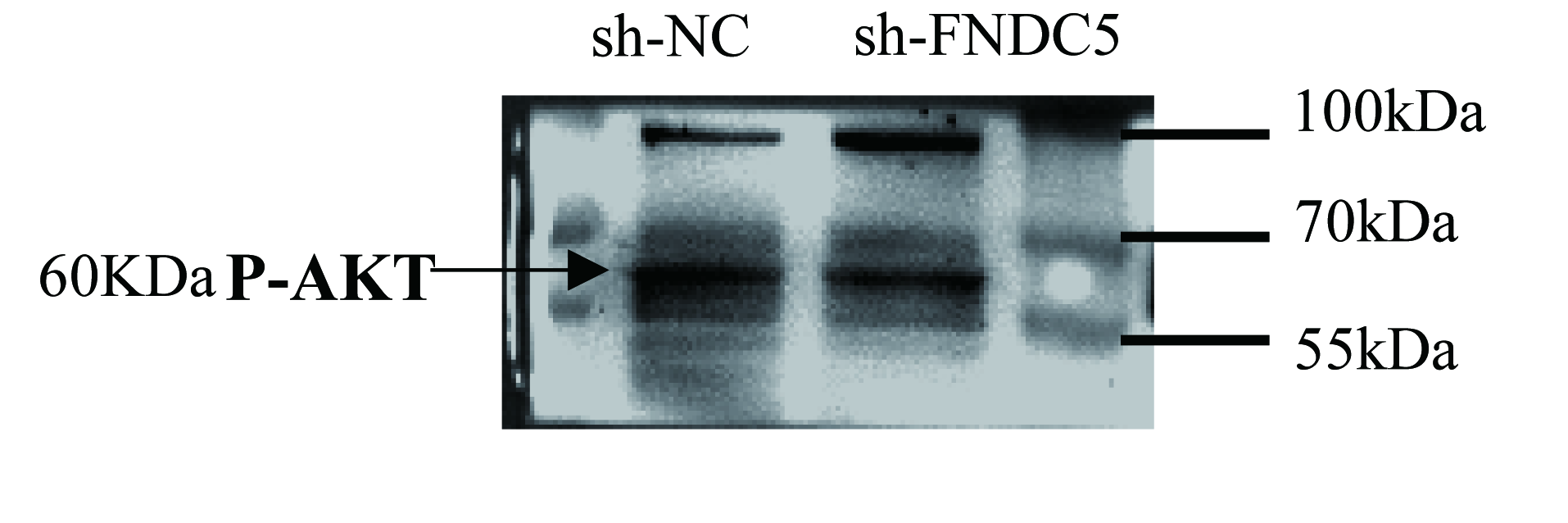

Supplement: Supplementary file 5 — Supplementary Material 5. [file 13395_2026_420_MOESM5_ESM.zip › Supplementary Material 5/Fig2/Fig2D/sh-FNDC5/P-AKT/P-AKT-1.tif]

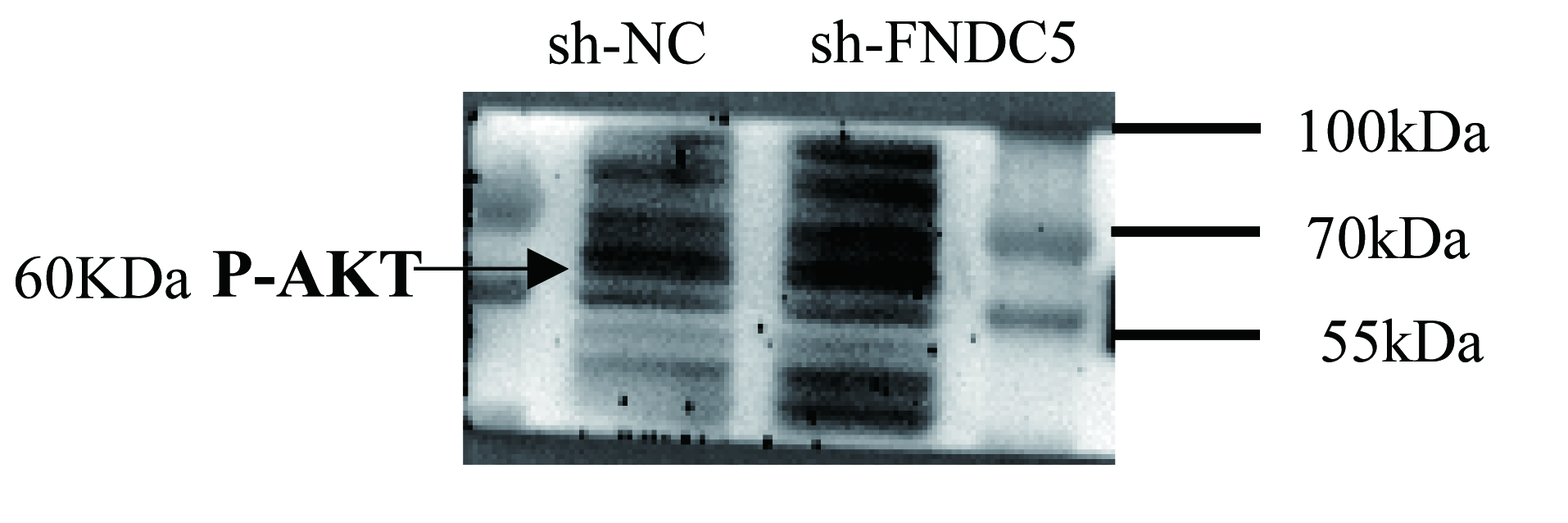

Supplement: Supplementary file 5 — Supplementary Material 5. [file 13395_2026_420_MOESM5_ESM.zip › Supplementary Material 5/Fig2/Fig2D/sh-FNDC5/P-AKT/P-AKT-2.tif]

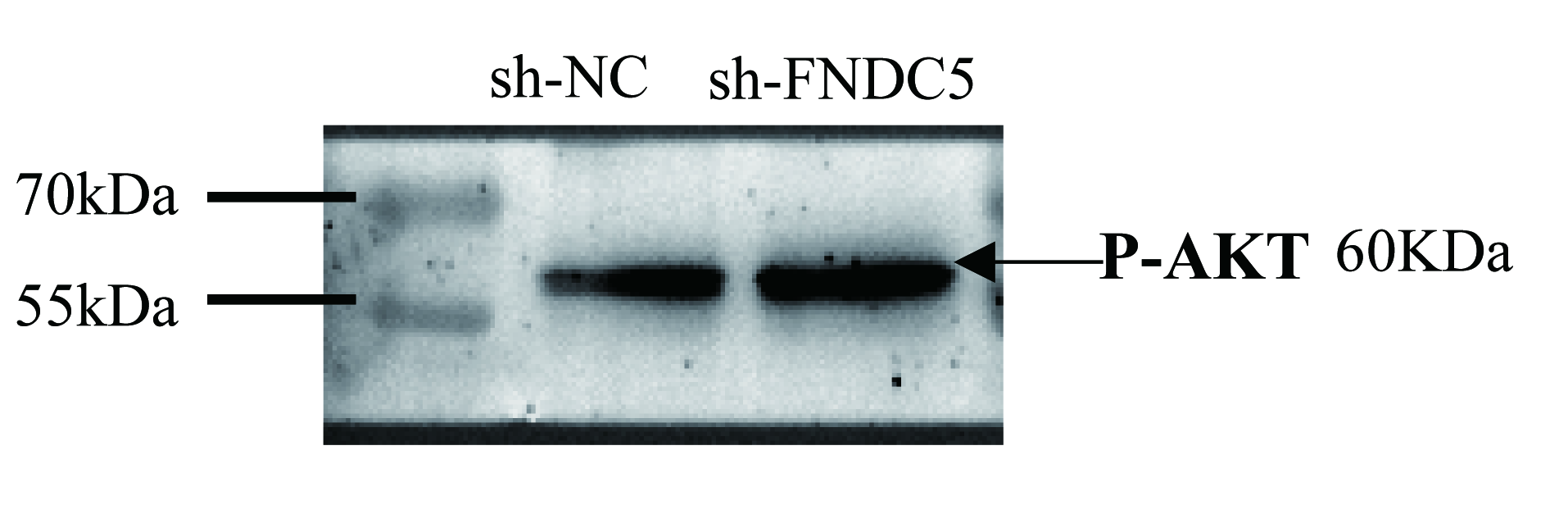

Supplement: Supplementary file 5 — Supplementary Material 5. [file 13395_2026_420_MOESM5_ESM.zip › Supplementary Material 5/Fig2/Fig2D/sh-FNDC5/P-AKT/P-AKT-3.tif]

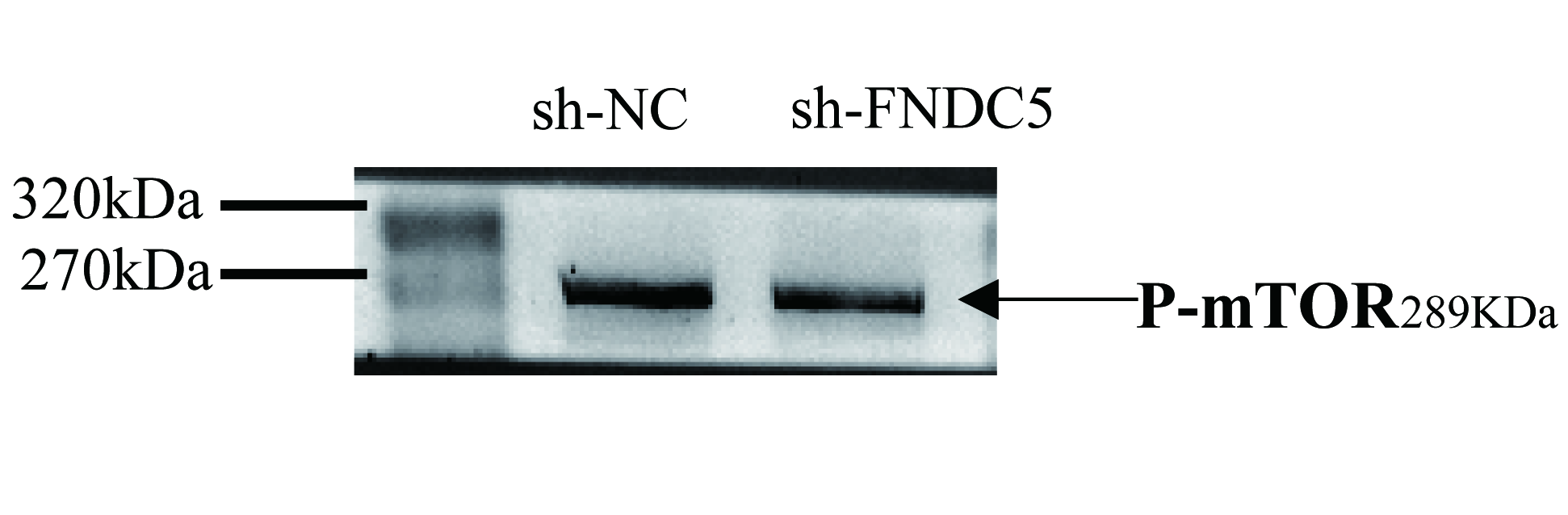

Supplement: Supplementary file 5 — Supplementary Material 5. [file 13395_2026_420_MOESM5_ESM.zip › Supplementary Material 5/Fig2/Fig2D/sh-FNDC5/P-mTOR/P-mTOR-1.tif]

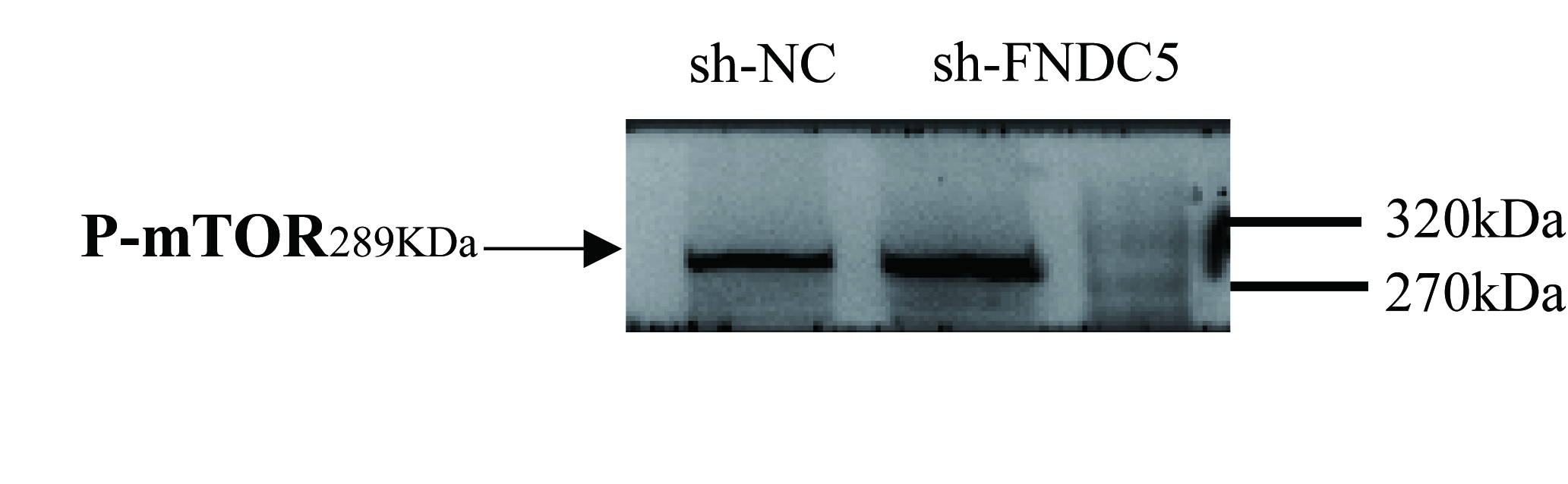

Supplement: Supplementary file 5 — Supplementary Material 5. [file 13395_2026_420_MOESM5_ESM.zip › Supplementary Material 5/Fig2/Fig2D/sh-FNDC5/P-mTOR/P-mTOR-2.tif]

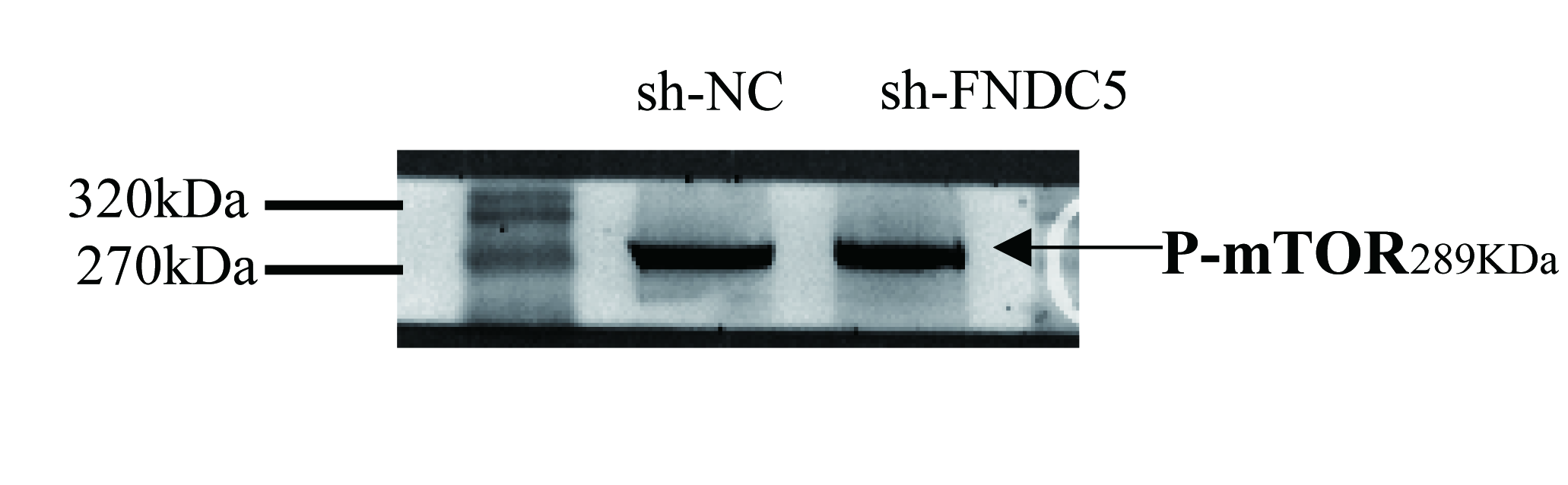

Supplement: Supplementary file 5 — Supplementary Material 5. [file 13395_2026_420_MOESM5_ESM.zip › Supplementary Material 5/Fig2/Fig2D/sh-FNDC5/P-mTOR/P-mTOR-3.tif]

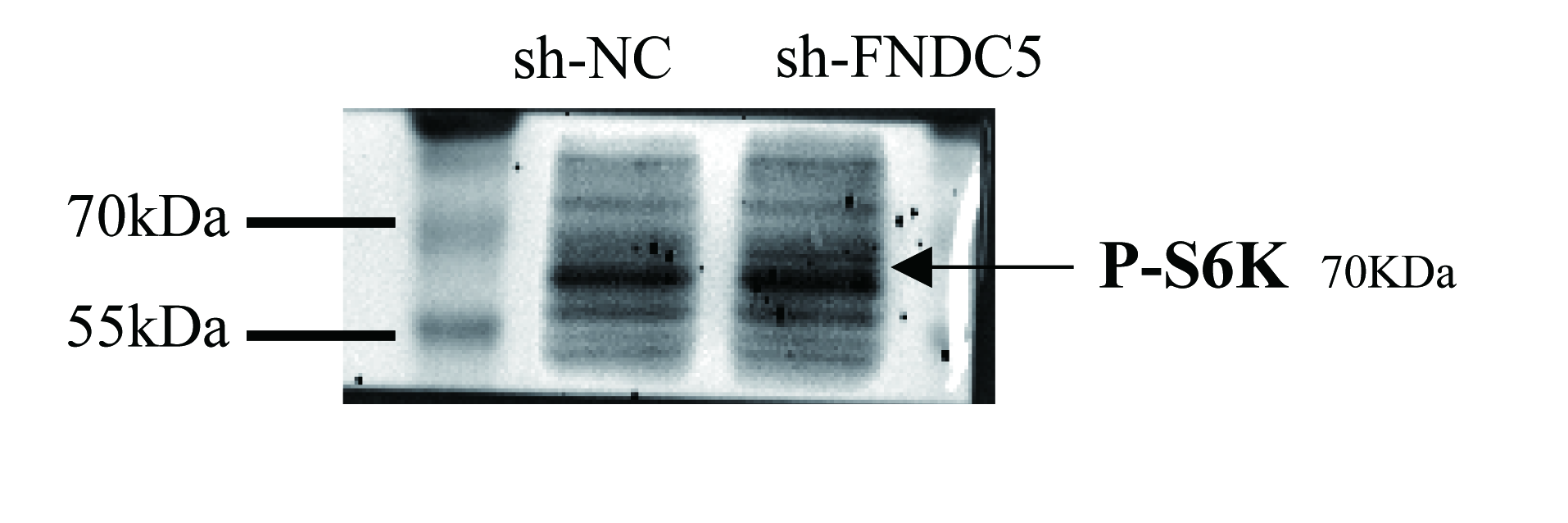

Supplement: Supplementary file 5 — Supplementary Material 5. [file 13395_2026_420_MOESM5_ESM.zip › Supplementary Material 5/Fig2/Fig2D/sh-FNDC5/P-S6K/P-S6K-1.tif]

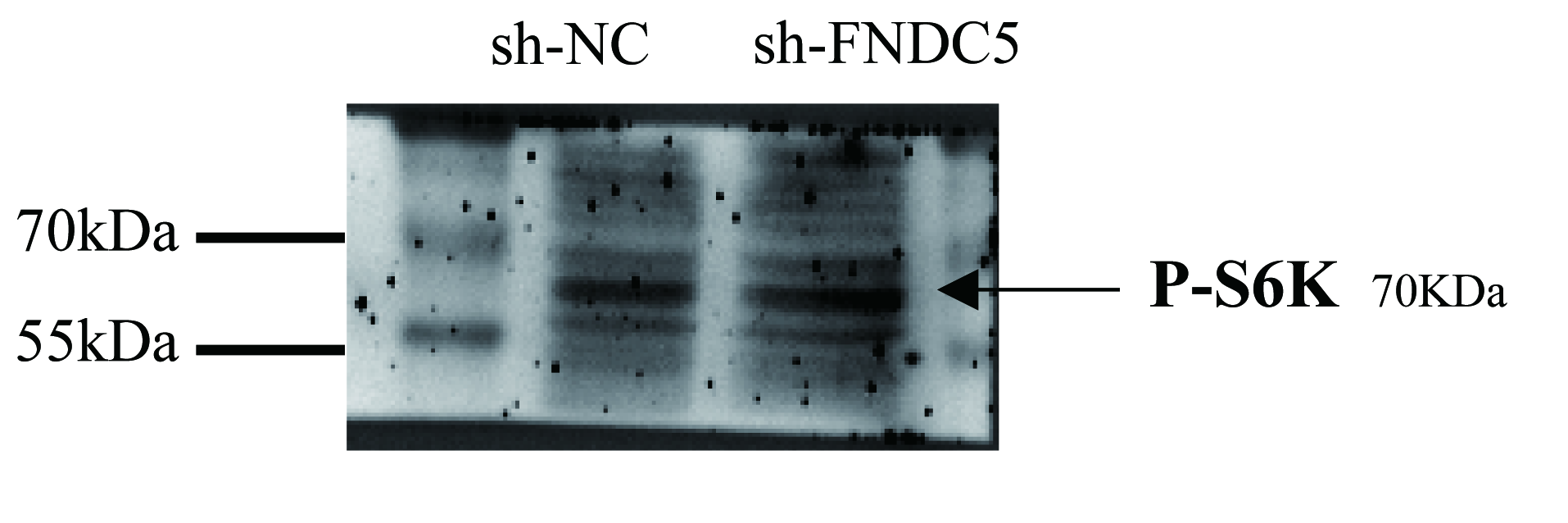

Supplement: Supplementary file 5 — Supplementary Material 5. [file 13395_2026_420_MOESM5_ESM.zip › Supplementary Material 5/Fig2/Fig2D/sh-FNDC5/P-S6K/P-S6K-2.tif]

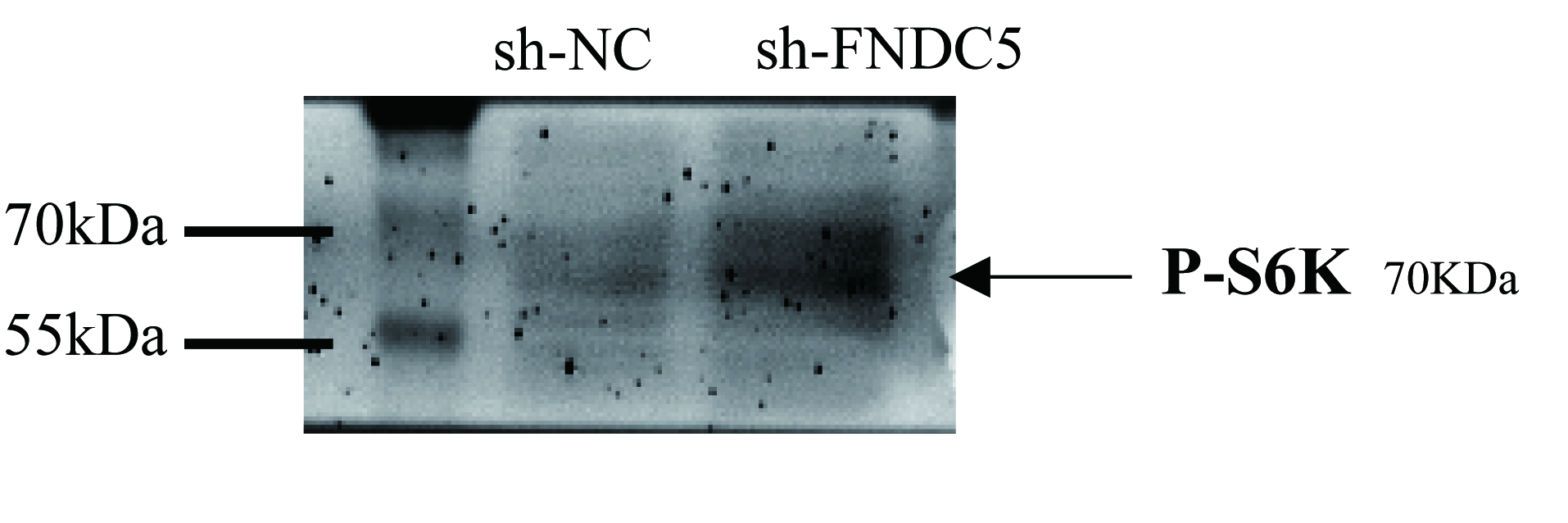

Supplement: Supplementary file 5 — Supplementary Material 5. [file 13395_2026_420_MOESM5_ESM.zip › Supplementary Material 5/Fig2/Fig2D/sh-FNDC5/P-S6K/P-S6K-3.tif]

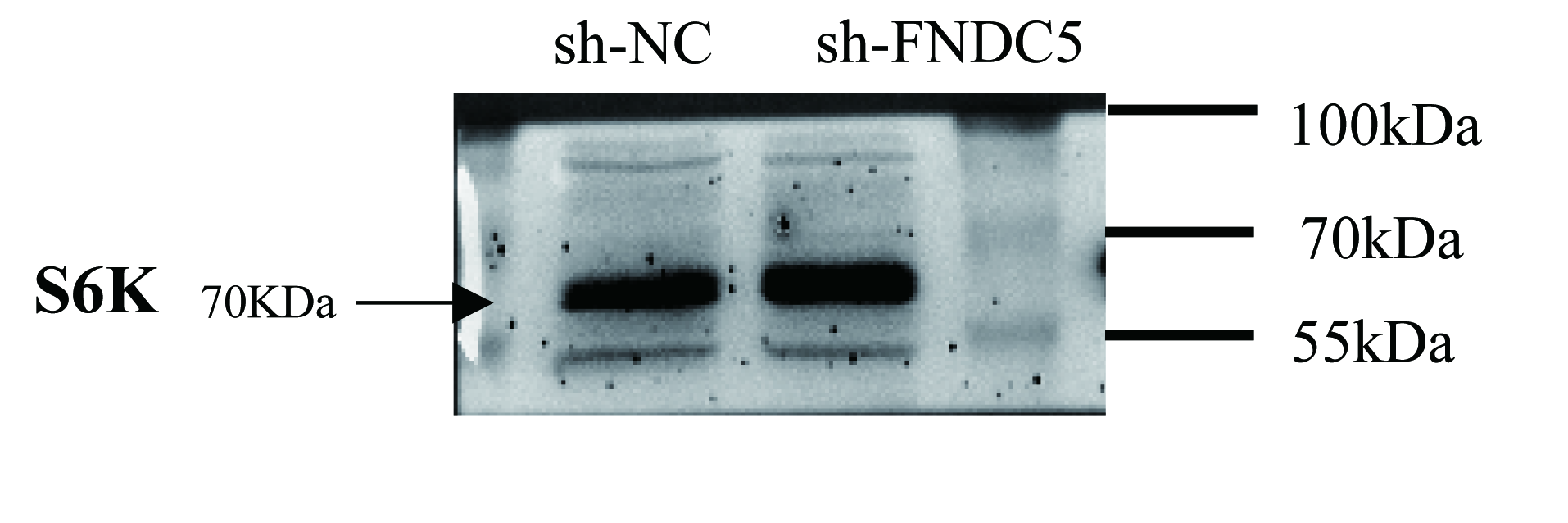

Supplement: Supplementary file 5 — Supplementary Material 5. [file 13395_2026_420_MOESM5_ESM.zip › Supplementary Material 5/Fig2/Fig2D/sh-FNDC5/S6K/S6K-1.tif]

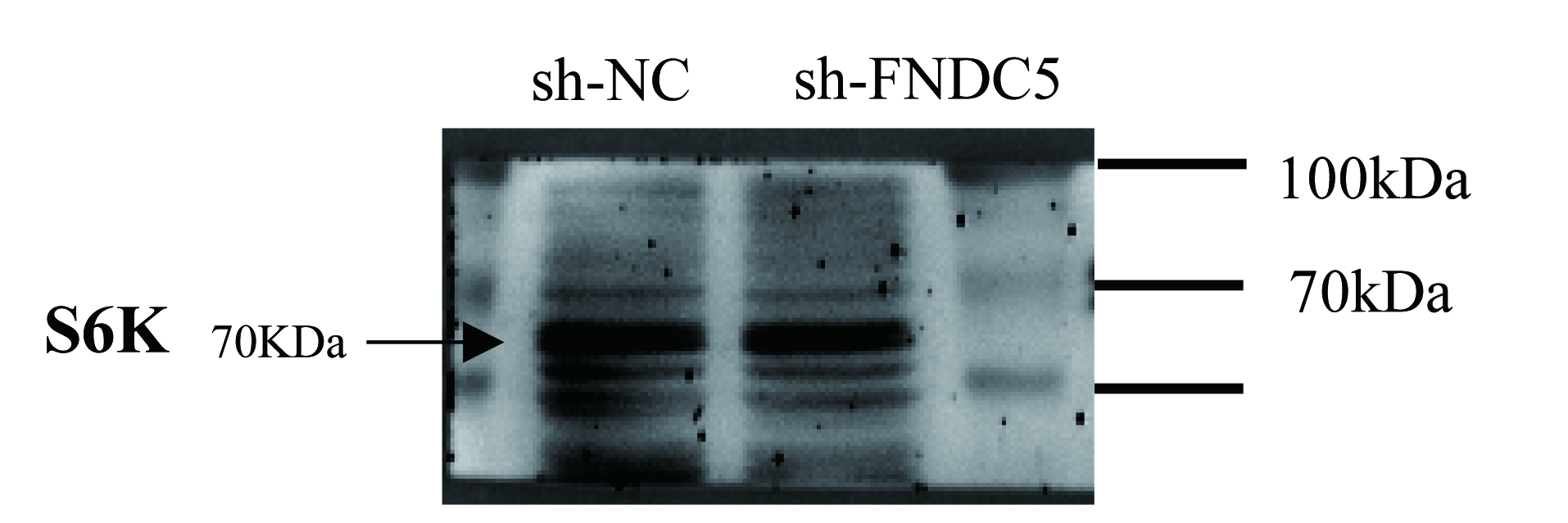

Supplement: Supplementary file 5 — Supplementary Material 5. [file 13395_2026_420_MOESM5_ESM.zip › Supplementary Material 5/Fig2/Fig2D/sh-FNDC5/S6K/S6K-2.tif]

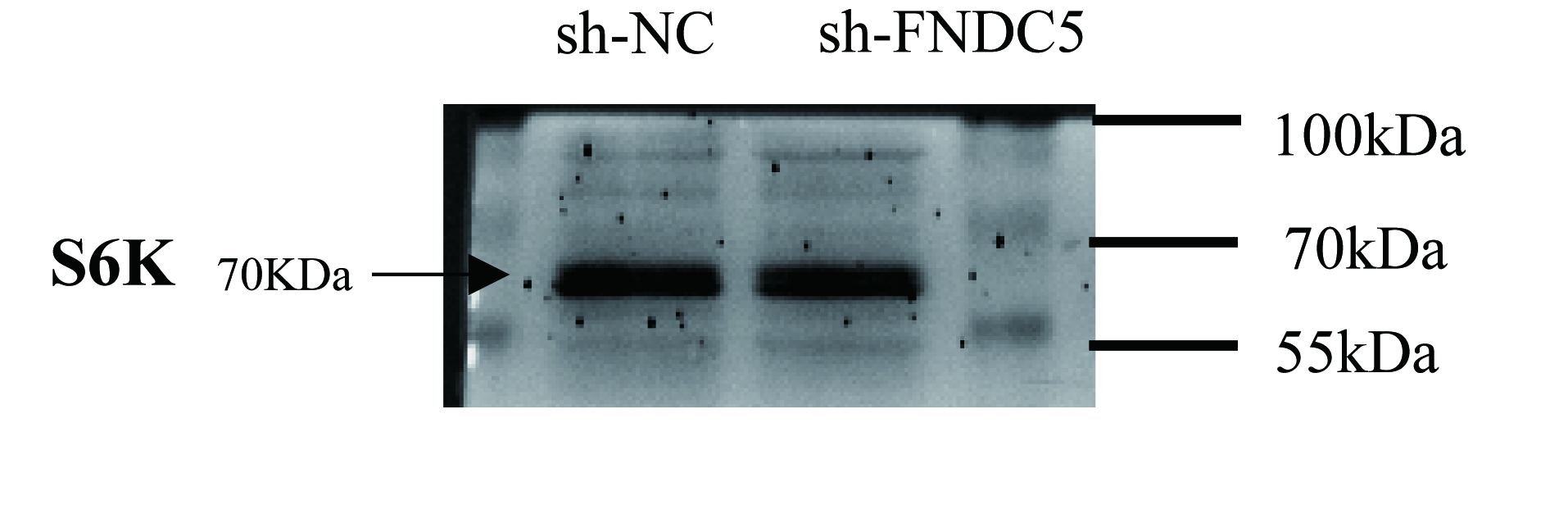

Supplement: Supplementary file 5 — Supplementary Material 5. [file 13395_2026_420_MOESM5_ESM.zip › Supplementary Material 5/Fig2/Fig2D/sh-FNDC5/S6K/S6K-3.tif]

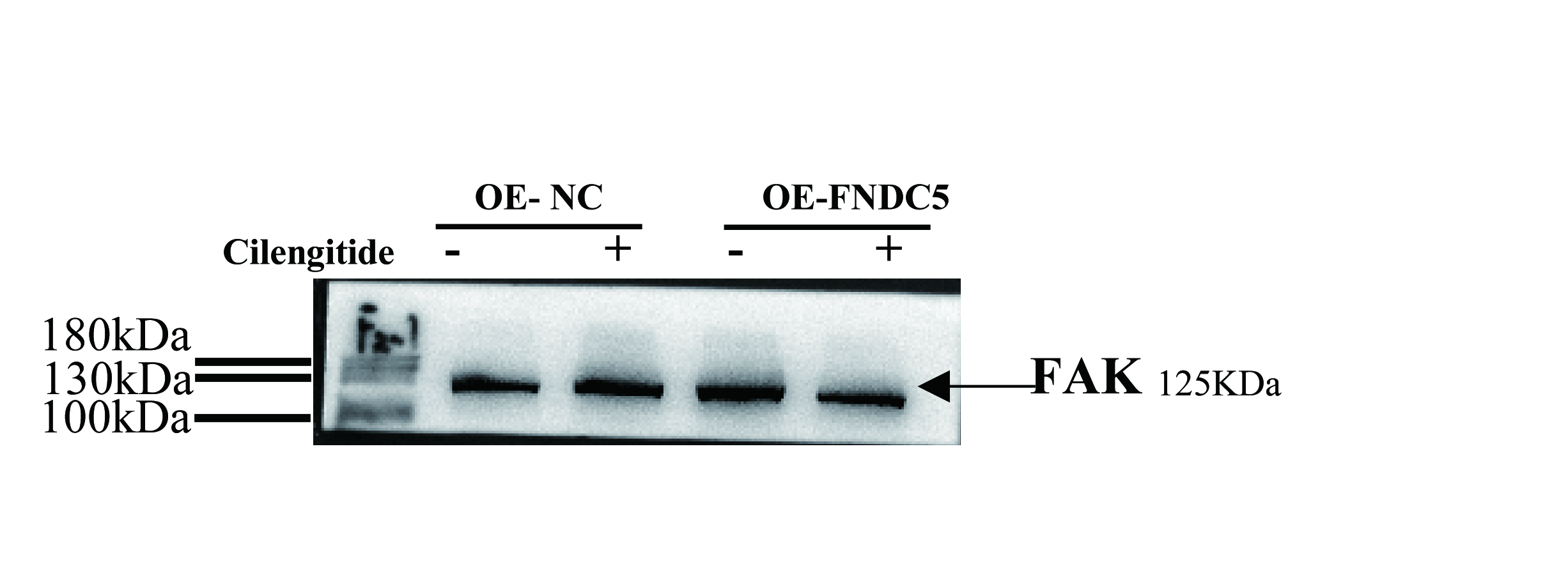

Supplement: Supplementary file 5 — Supplementary Material 5. [file 13395_2026_420_MOESM5_ESM.zip › Supplementary Material 5/Fig3/Fig3D/FAK/FAK-1.tif]

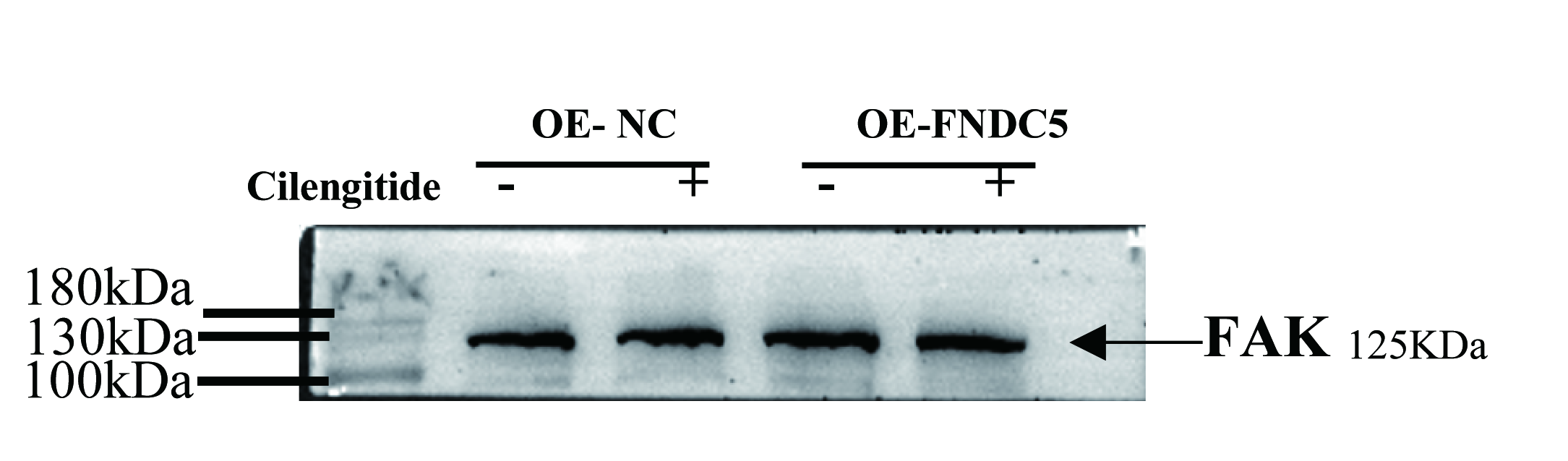

Supplement: Supplementary file 5 — Supplementary Material 5. [file 13395_2026_420_MOESM5_ESM.zip › Supplementary Material 5/Fig3/Fig3D/FAK/FAK-2.tif]

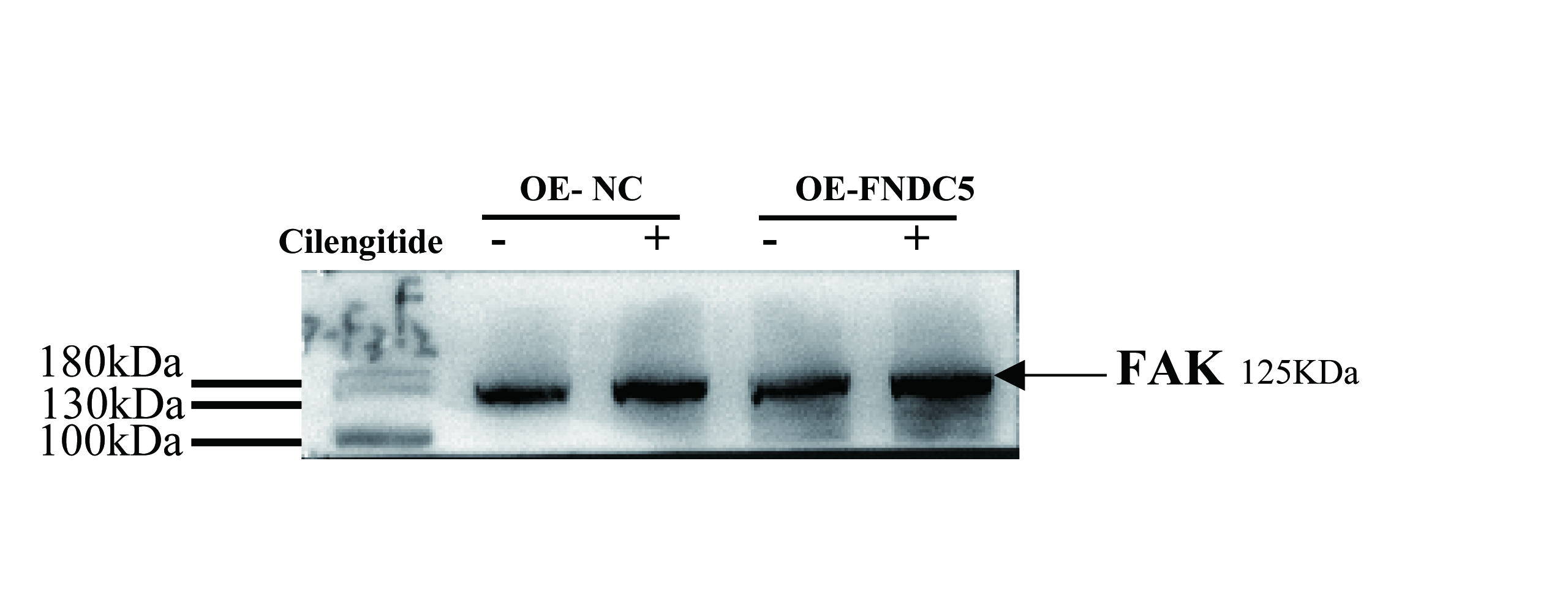

Supplement: Supplementary file 5 — Supplementary Material 5. [file 13395_2026_420_MOESM5_ESM.zip › Supplementary Material 5/Fig3/Fig3D/FAK/FAK-3.tif]

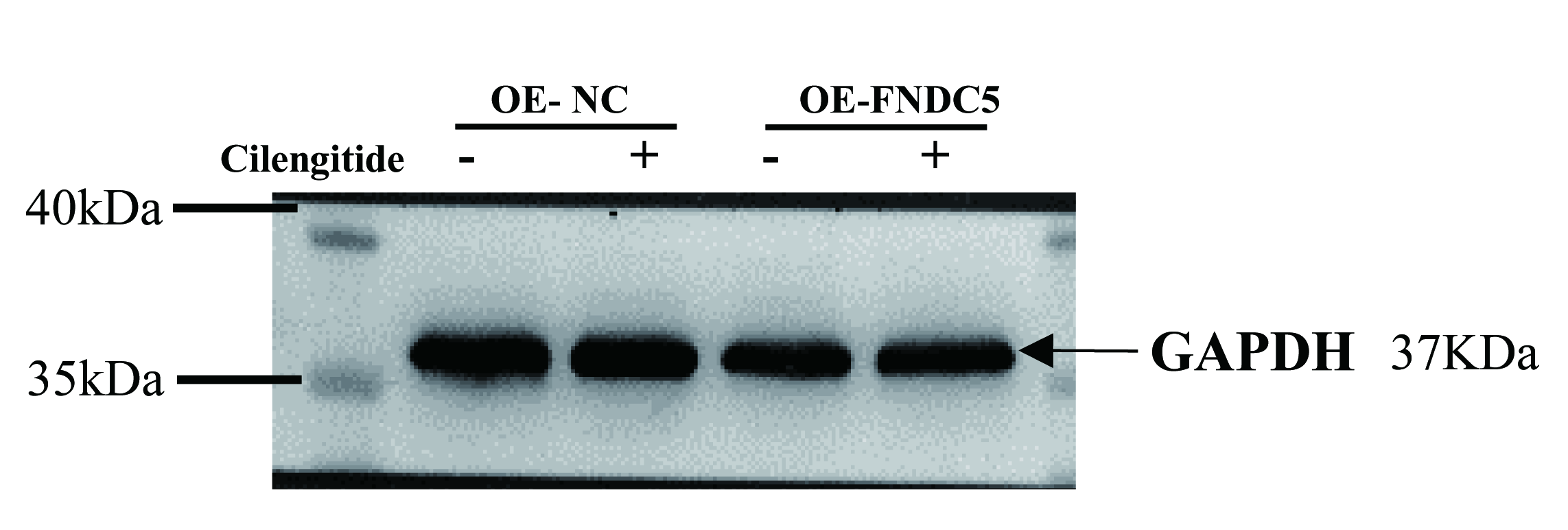

Supplement: Supplementary file 5 — Supplementary Material 5. [file 13395_2026_420_MOESM5_ESM.zip › Supplementary Material 5/Fig3/Fig3D/GAPDH/GAPDH-1.tif]

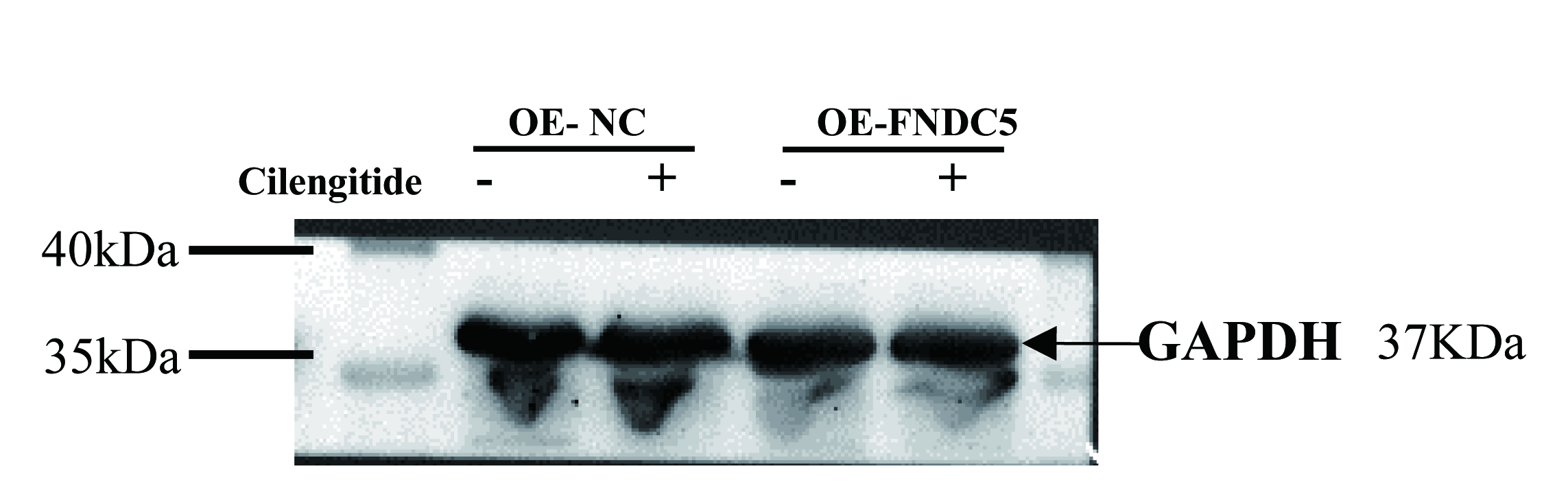

Supplement: Supplementary file 5 — Supplementary Material 5. [file 13395_2026_420_MOESM5_ESM.zip › Supplementary Material 5/Fig3/Fig3D/GAPDH/GAPDH-2.tif]

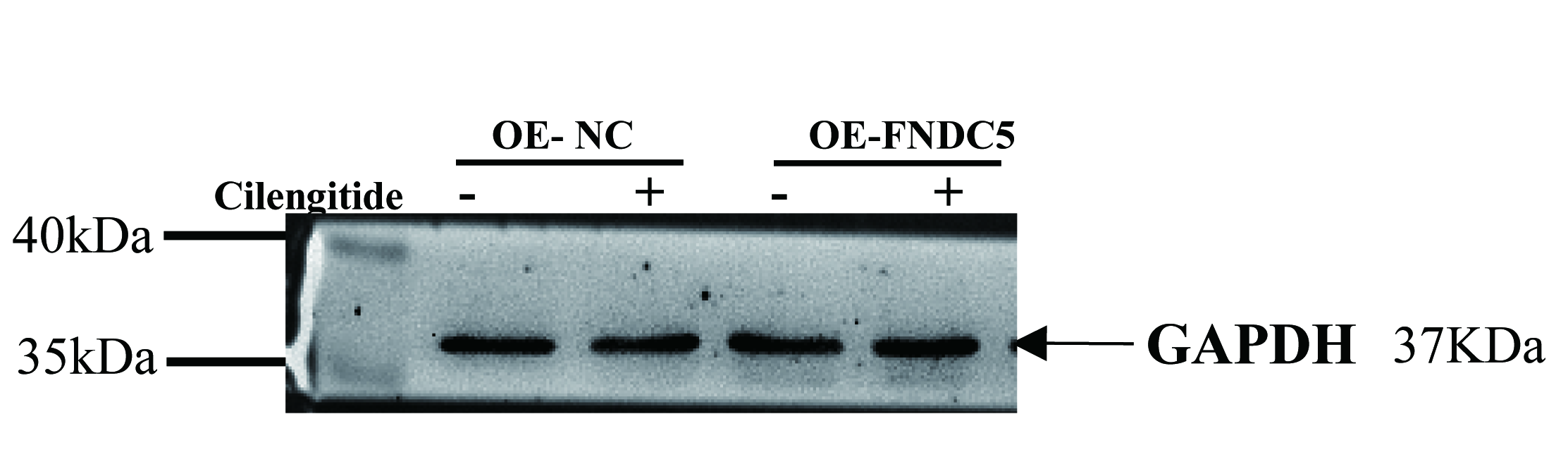

Supplement: Supplementary file 5 — Supplementary Material 5. [file 13395_2026_420_MOESM5_ESM.zip › Supplementary Material 5/Fig3/Fig3D/GAPDH/GAPDH-3.tif]

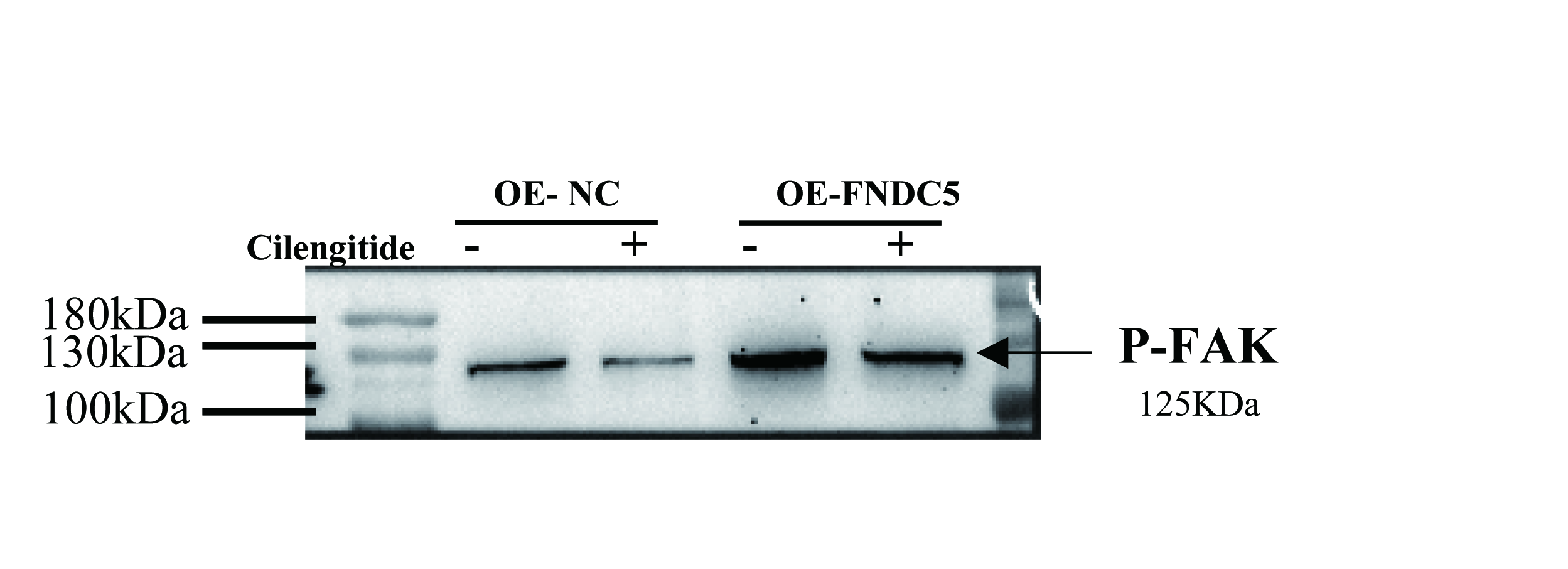

Supplement: Supplementary file 5 — Supplementary Material 5. [file 13395_2026_420_MOESM5_ESM.zip › Supplementary Material 5/Fig3/Fig3D/P-FAK/P-FAK-1.tif]

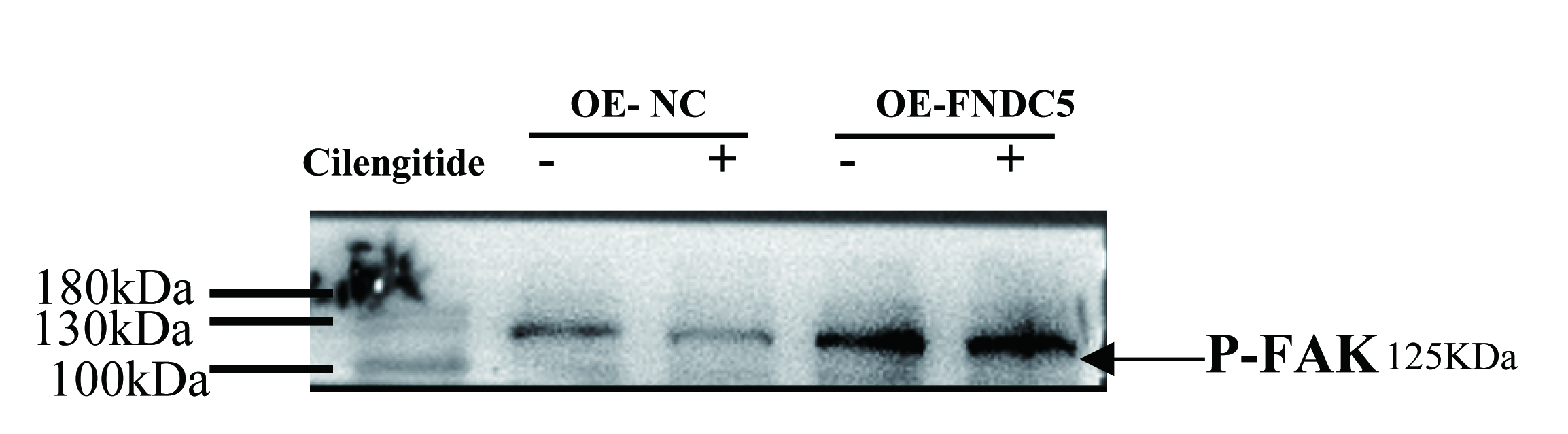

Supplement: Supplementary file 5 — Supplementary Material 5. [file 13395_2026_420_MOESM5_ESM.zip › Supplementary Material 5/Fig3/Fig3D/P-FAK/P-FAK-2.tif]

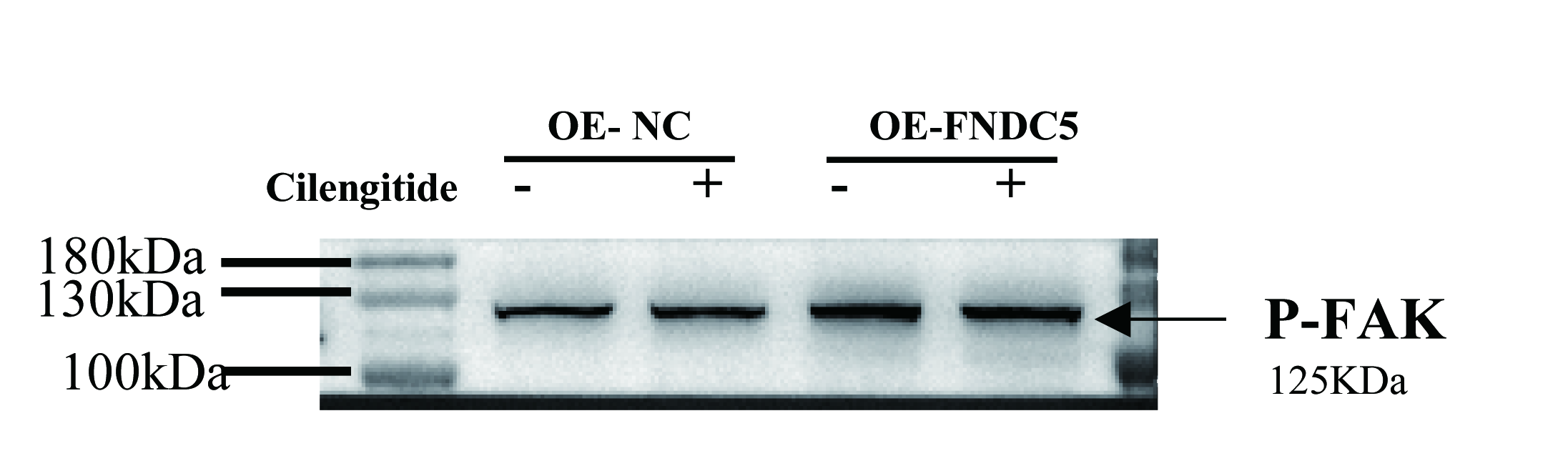

Supplement: Supplementary file 5 — Supplementary Material 5. [file 13395_2026_420_MOESM5_ESM.zip › Supplementary Material 5/Fig3/Fig3D/P-FAK/P-FAK-3.tif]

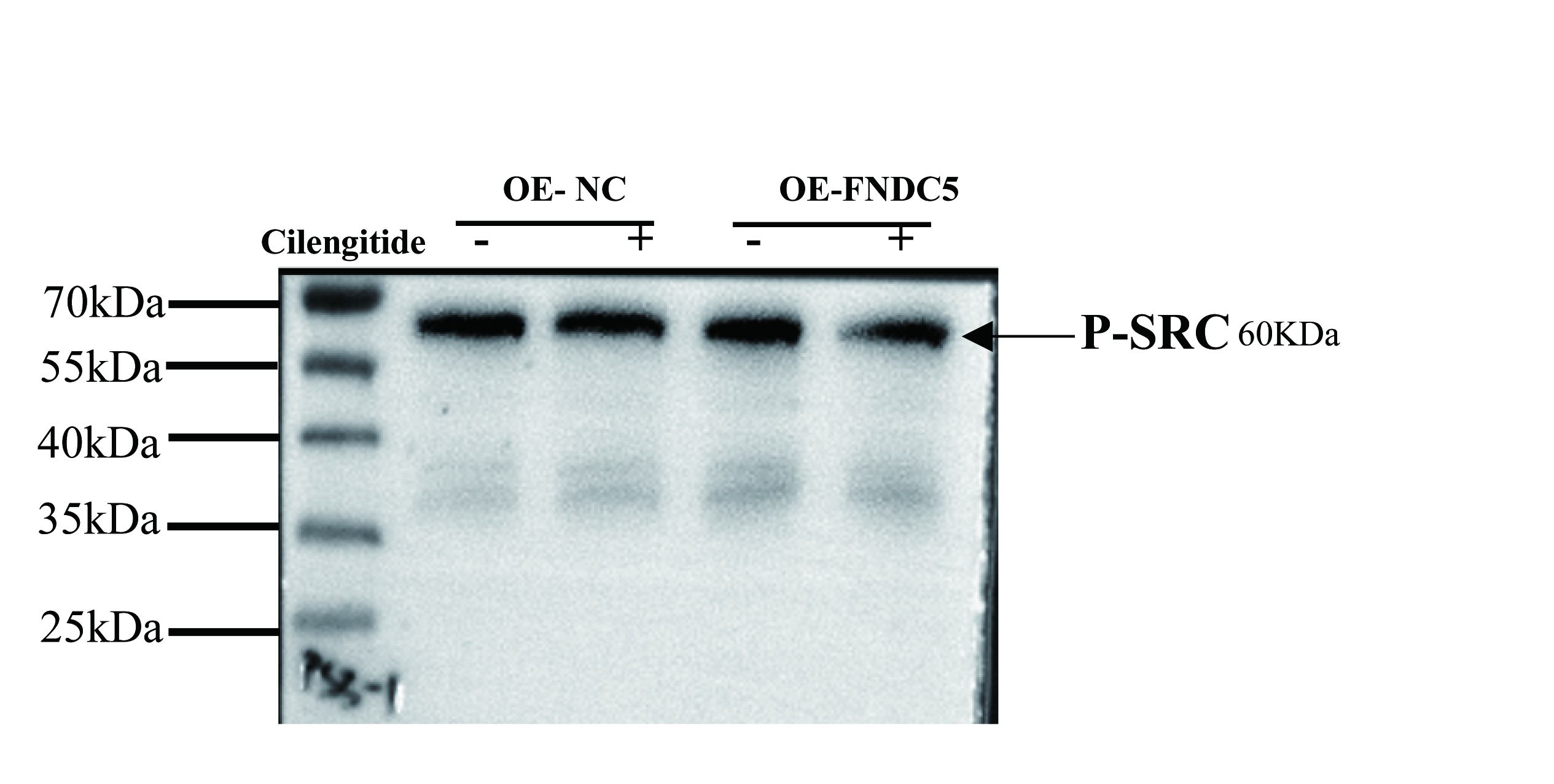

Supplement: Supplementary file 5 — Supplementary Material 5. [file 13395_2026_420_MOESM5_ESM.zip › Supplementary Material 5/Fig3/Fig3D/P-SRC/P-SRC-1.tif]

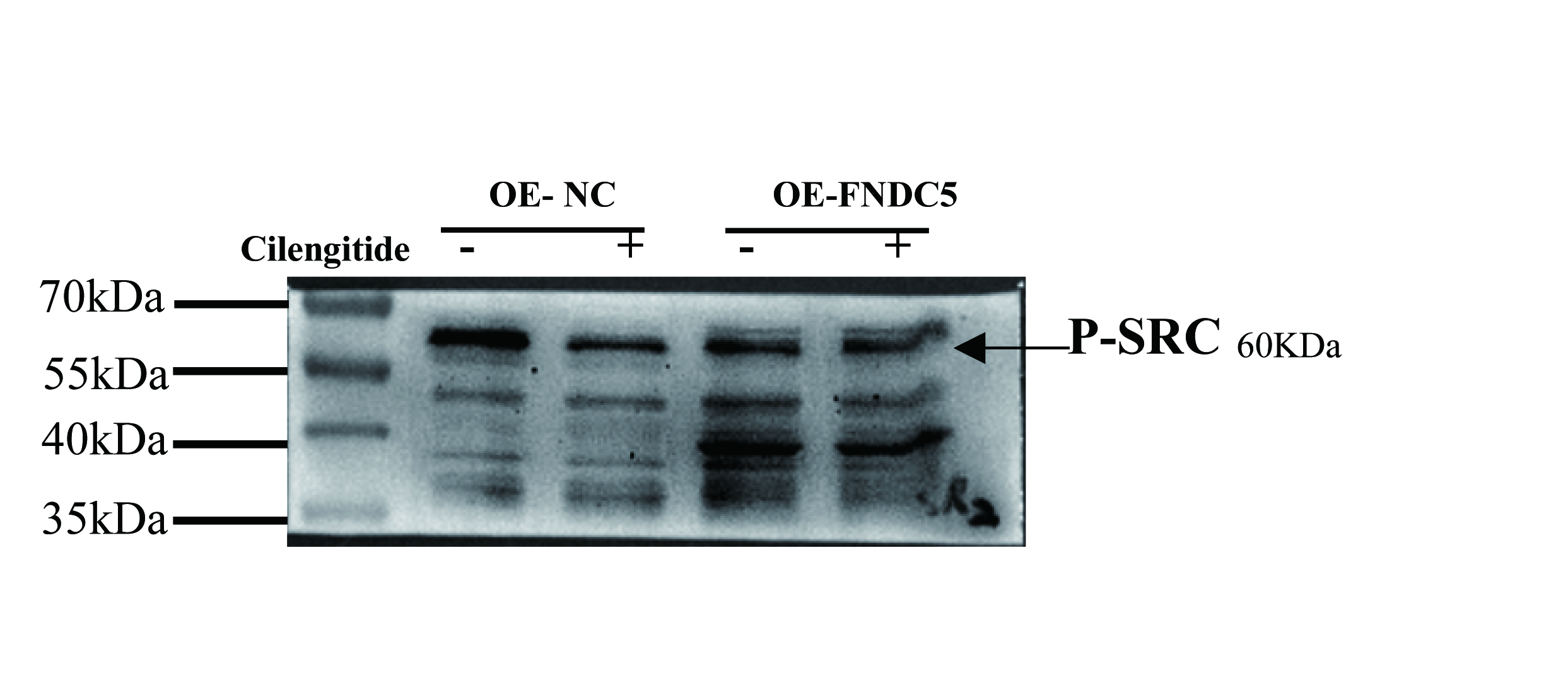

Supplement: Supplementary file 5 — Supplementary Material 5. [file 13395_2026_420_MOESM5_ESM.zip › Supplementary Material 5/Fig3/Fig3D/P-SRC/P-SRC-2.tif]

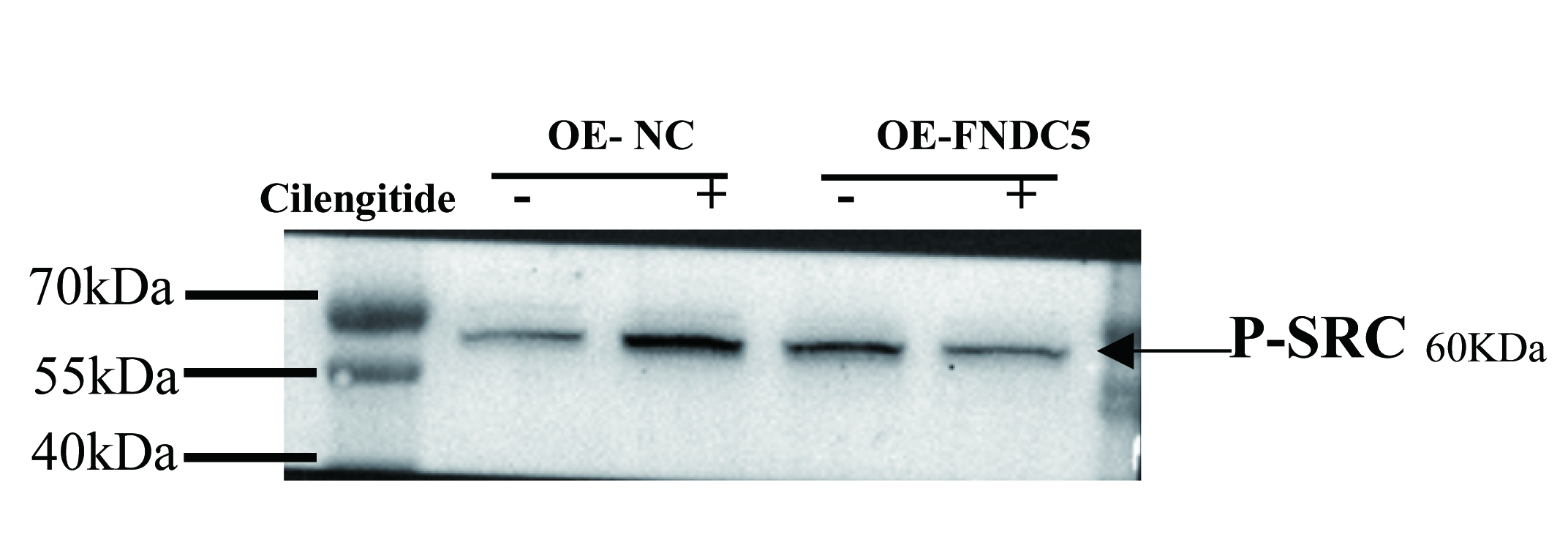

Supplement: Supplementary file 5 — Supplementary Material 5. [file 13395_2026_420_MOESM5_ESM.zip › Supplementary Material 5/Fig3/Fig3D/P-SRC/P-SRC-3.tif]

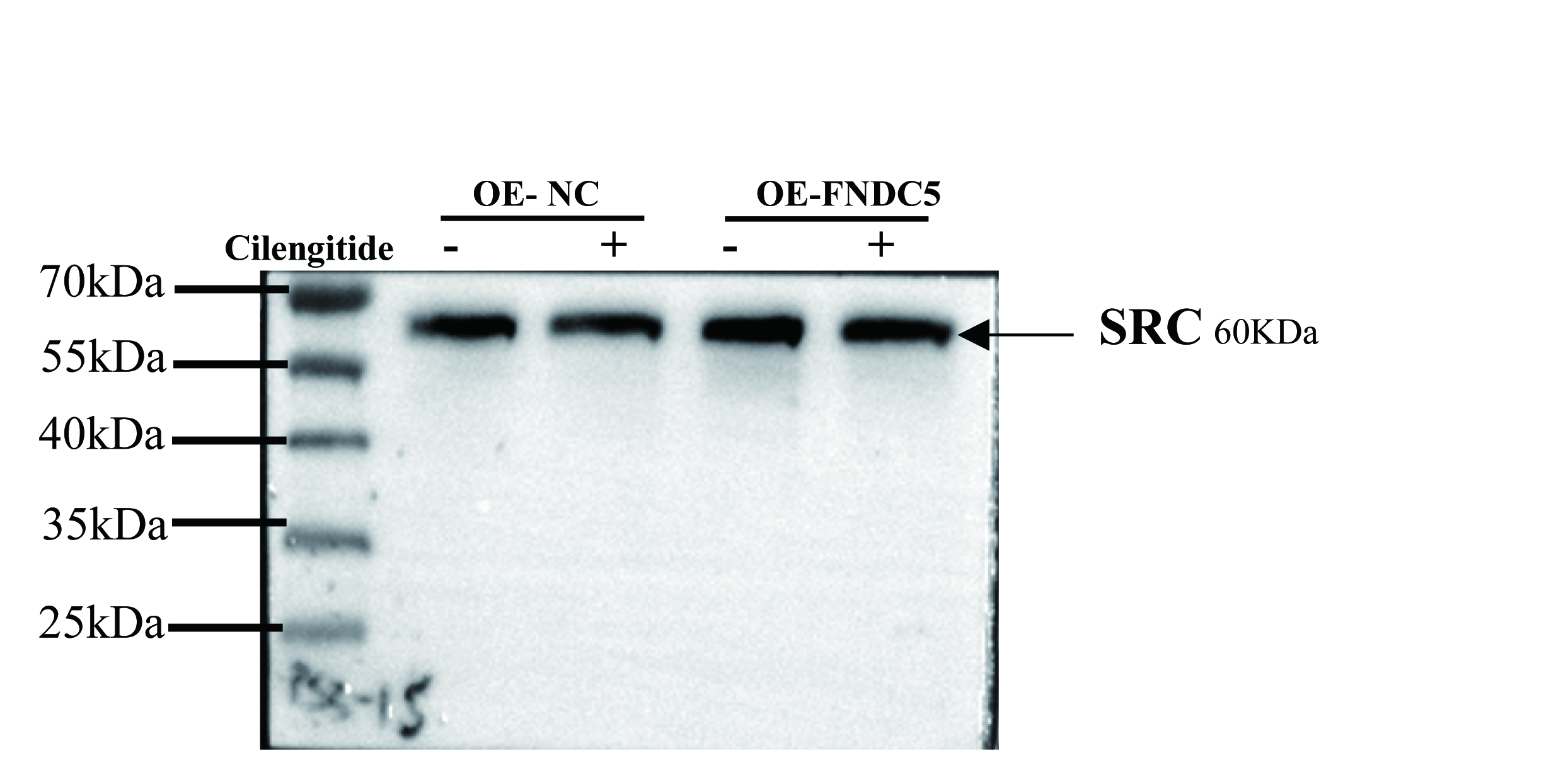

Supplement: Supplementary file 5 — Supplementary Material 5. [file 13395_2026_420_MOESM5_ESM.zip › Supplementary Material 5/Fig3/Fig3D/SRC/SRC-1.tif]

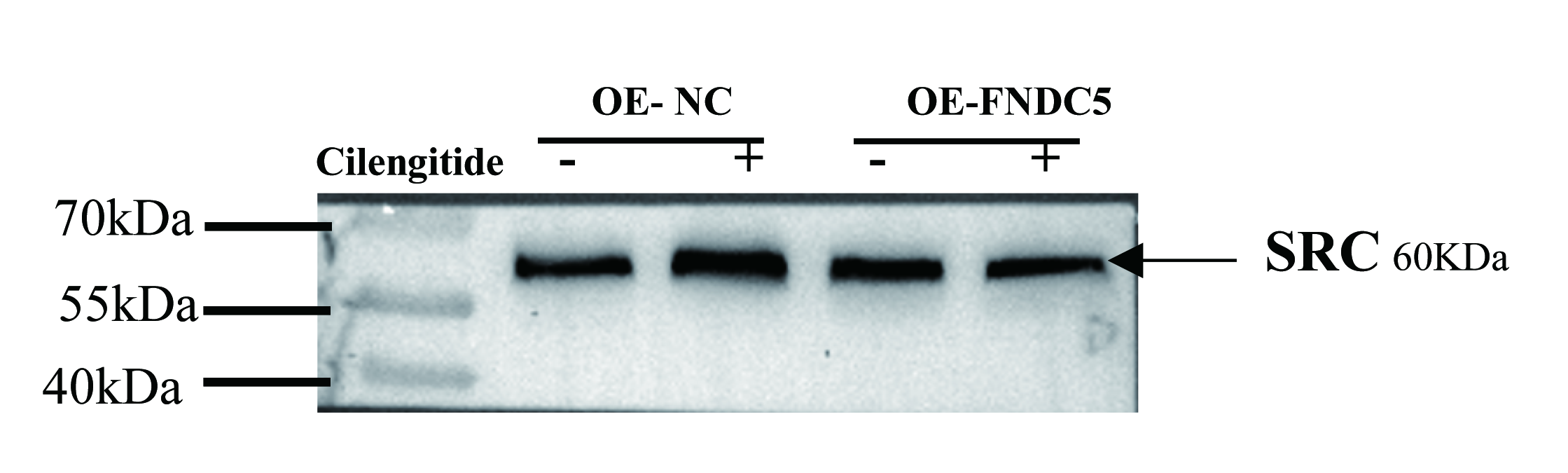

Supplement: Supplementary file 5 — Supplementary Material 5. [file 13395_2026_420_MOESM5_ESM.zip › Supplementary Material 5/Fig3/Fig3D/SRC/SRC-2.tif]

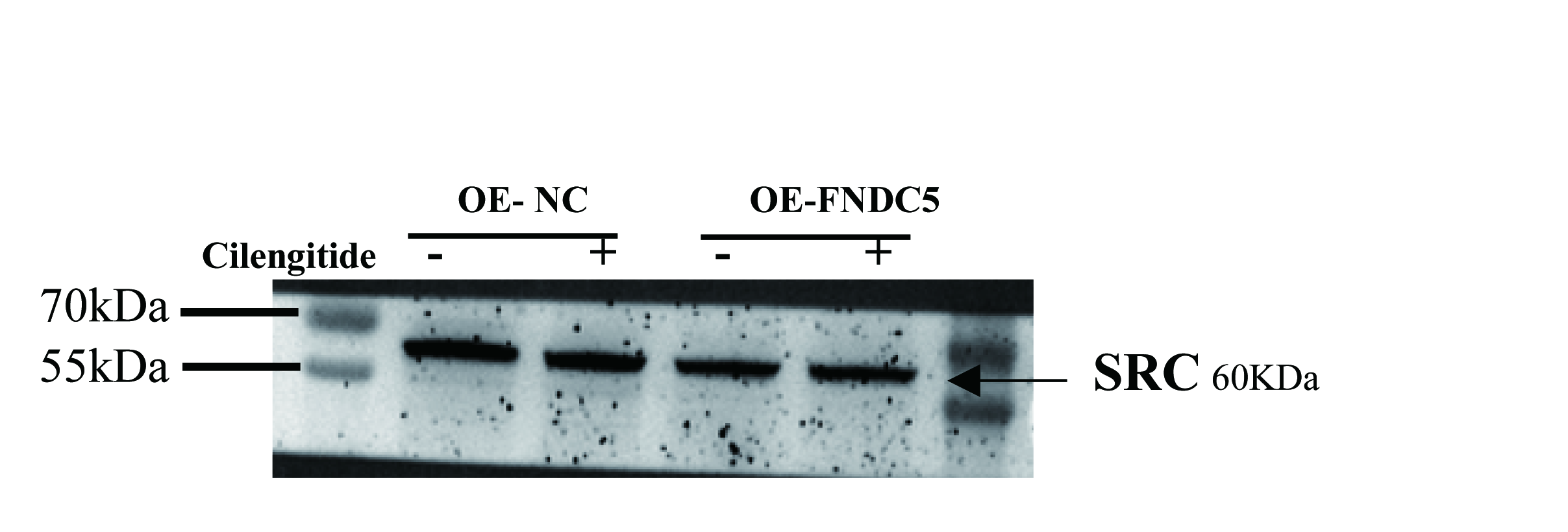

Supplement: Supplementary file 5 — Supplementary Material 5. [file 13395_2026_420_MOESM5_ESM.zip › Supplementary Material 5/Fig3/Fig3D/SRC/SRC-3.tif]

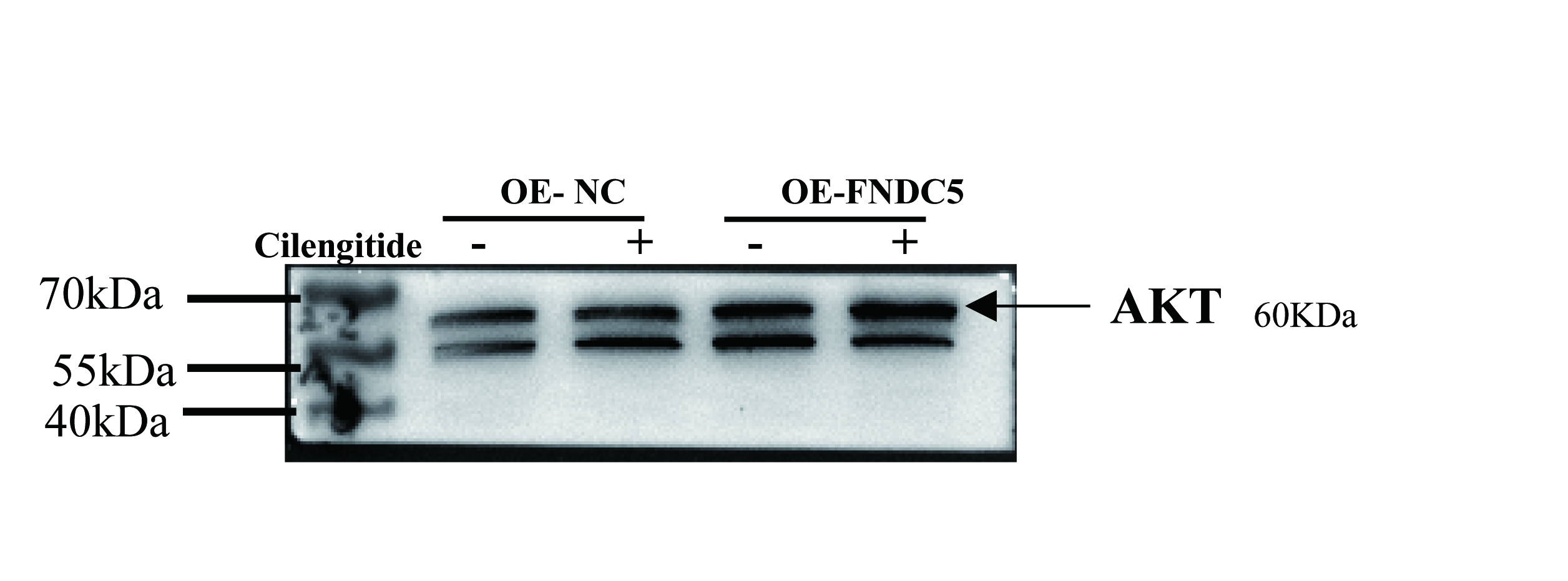

Supplement: Supplementary file 5 — Supplementary Material 5. [file 13395_2026_420_MOESM5_ESM.zip › Supplementary Material 5/Fig3/Fig3H/AKT/AKT-1.tif]

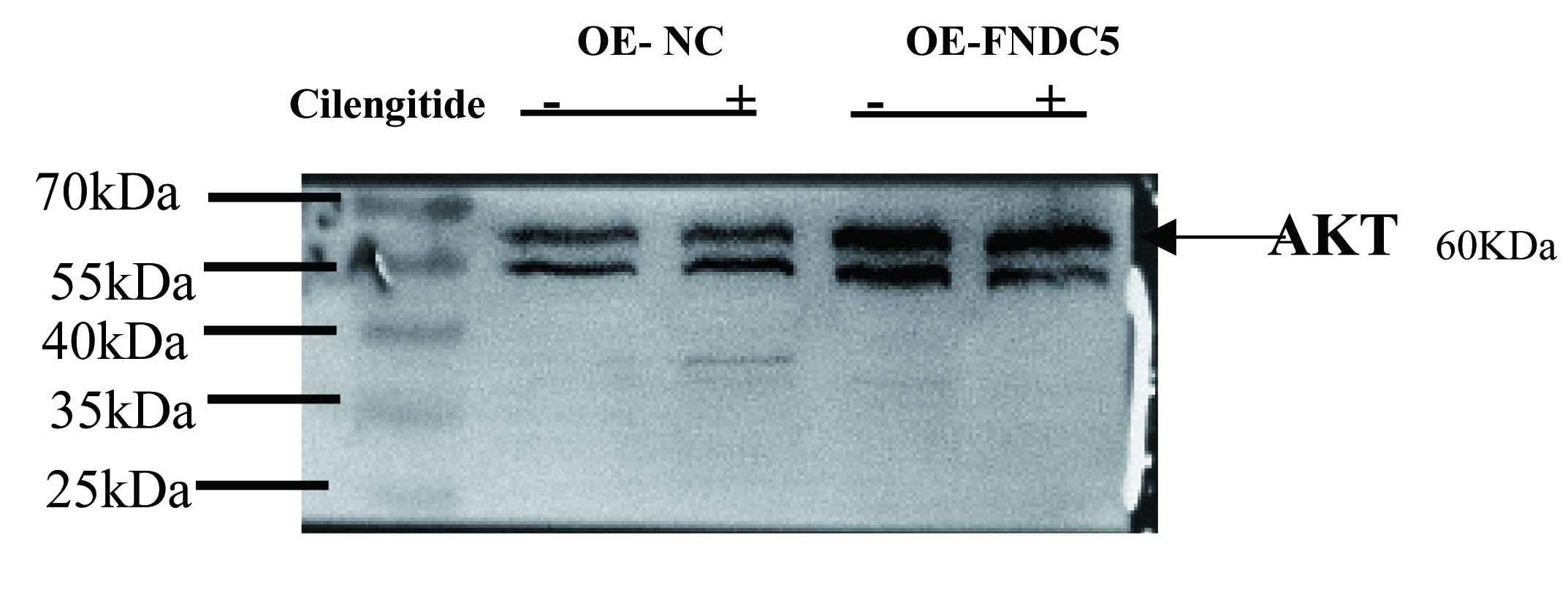

Supplement: Supplementary file 5 — Supplementary Material 5. [file 13395_2026_420_MOESM5_ESM.zip › Supplementary Material 5/Fig3/Fig3H/AKT/AKT-2.tif]

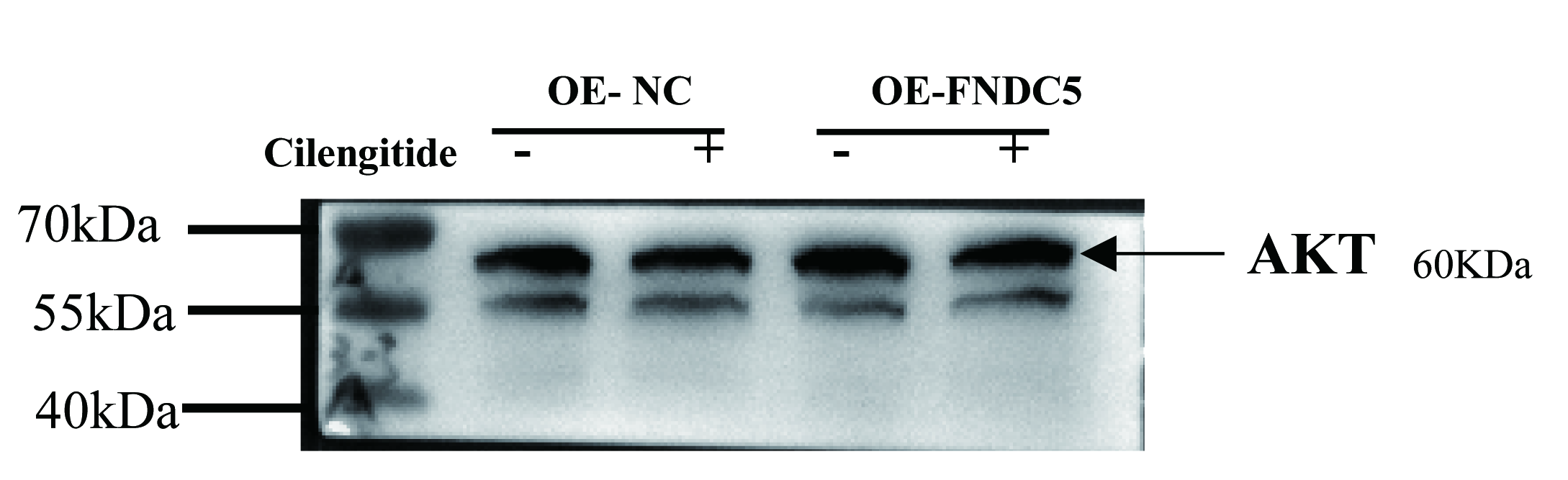

Supplement: Supplementary file 5 — Supplementary Material 5. [file 13395_2026_420_MOESM5_ESM.zip › Supplementary Material 5/Fig3/Fig3H/AKT/AKT-3.tif]

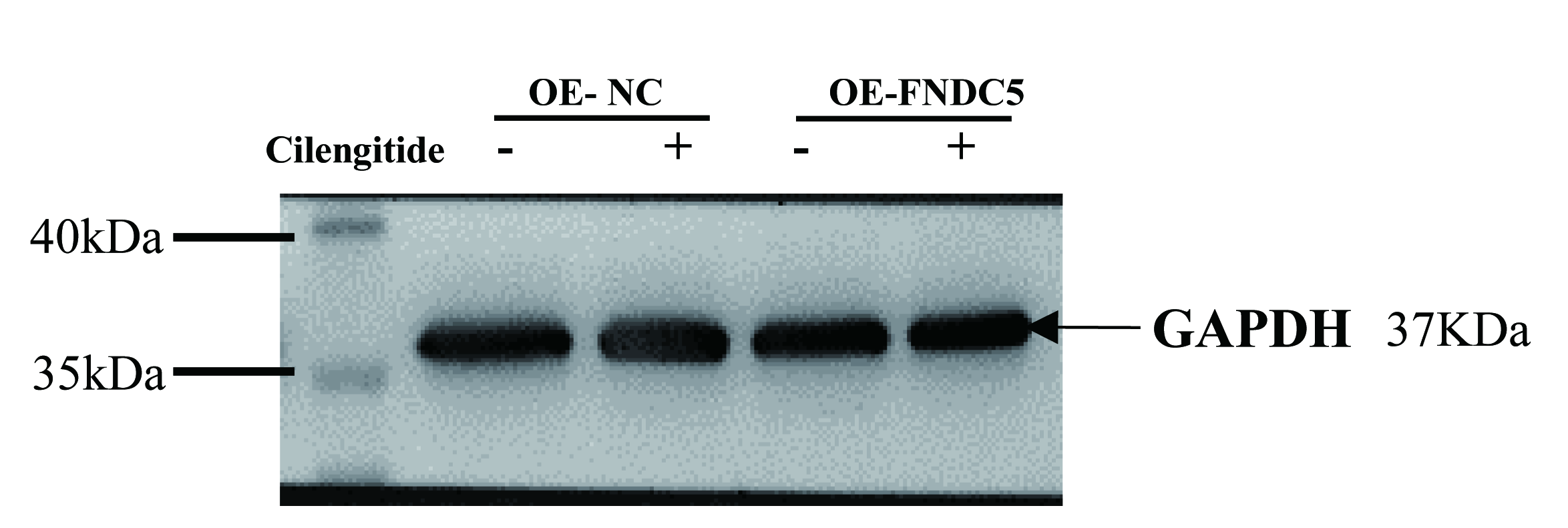

Supplement: Supplementary file 5 — Supplementary Material 5. [file 13395_2026_420_MOESM5_ESM.zip › Supplementary Material 5/Fig3/Fig3H/GAPDH/GAPDH-1.tif]

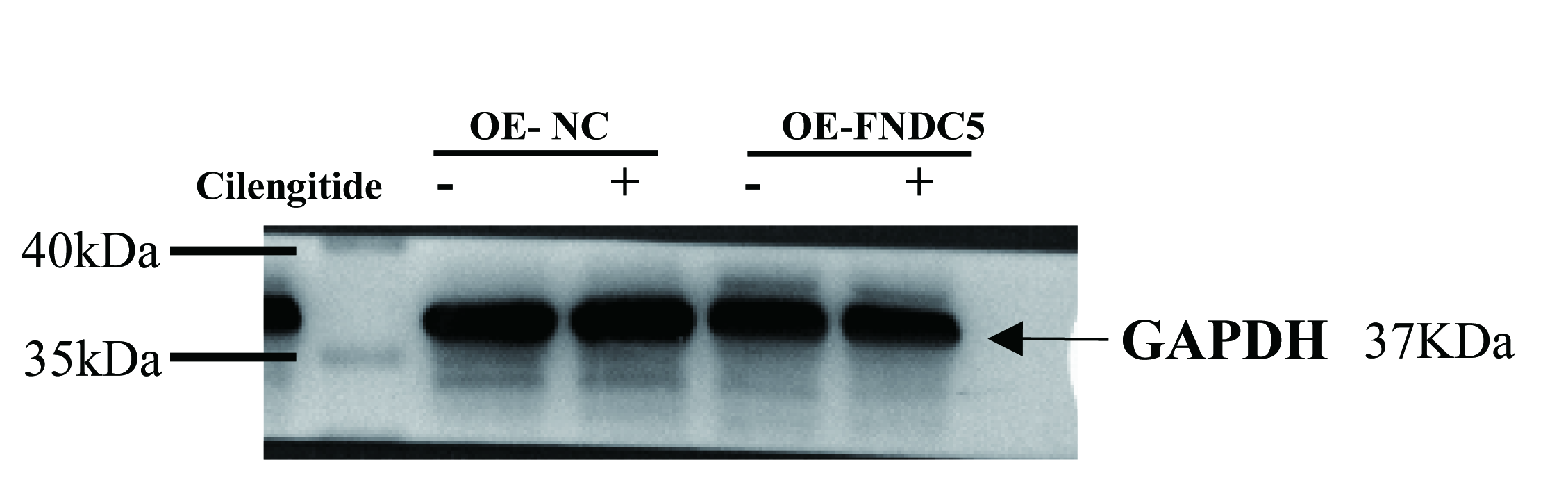

Supplement: Supplementary file 5 — Supplementary Material 5. [file 13395_2026_420_MOESM5_ESM.zip › Supplementary Material 5/Fig3/Fig3H/GAPDH/GAPDH-2.tif]

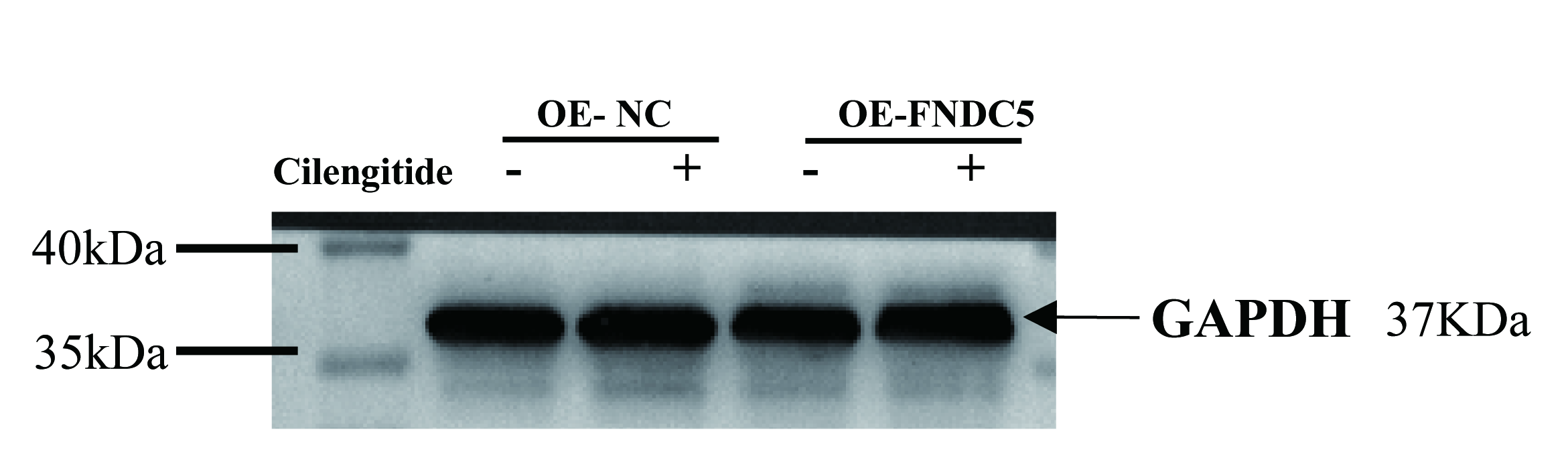

Supplement: Supplementary file 5 — Supplementary Material 5. [file 13395_2026_420_MOESM5_ESM.zip › Supplementary Material 5/Fig3/Fig3H/GAPDH/GAPDH-3.tif]

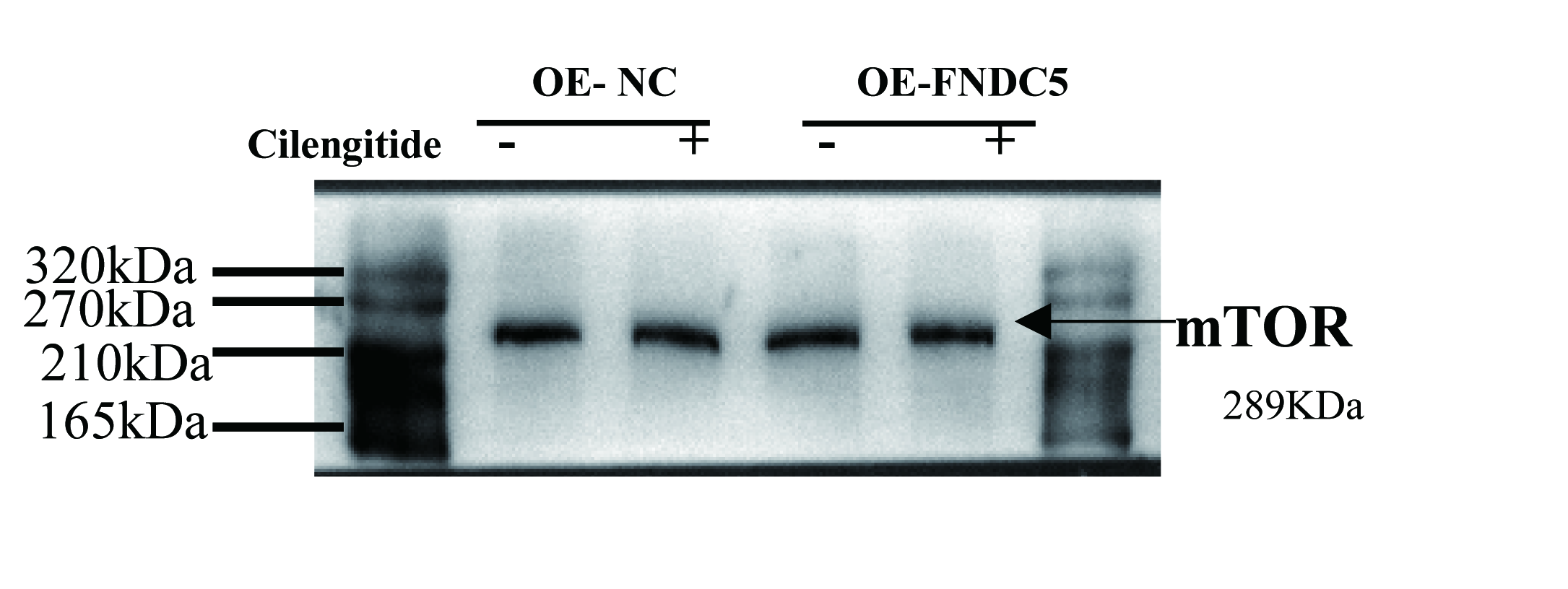

Supplement: Supplementary file 5 — Supplementary Material 5. [file 13395_2026_420_MOESM5_ESM.zip › Supplementary Material 5/Fig3/Fig3H/mTOR/mTOR-1.tif]

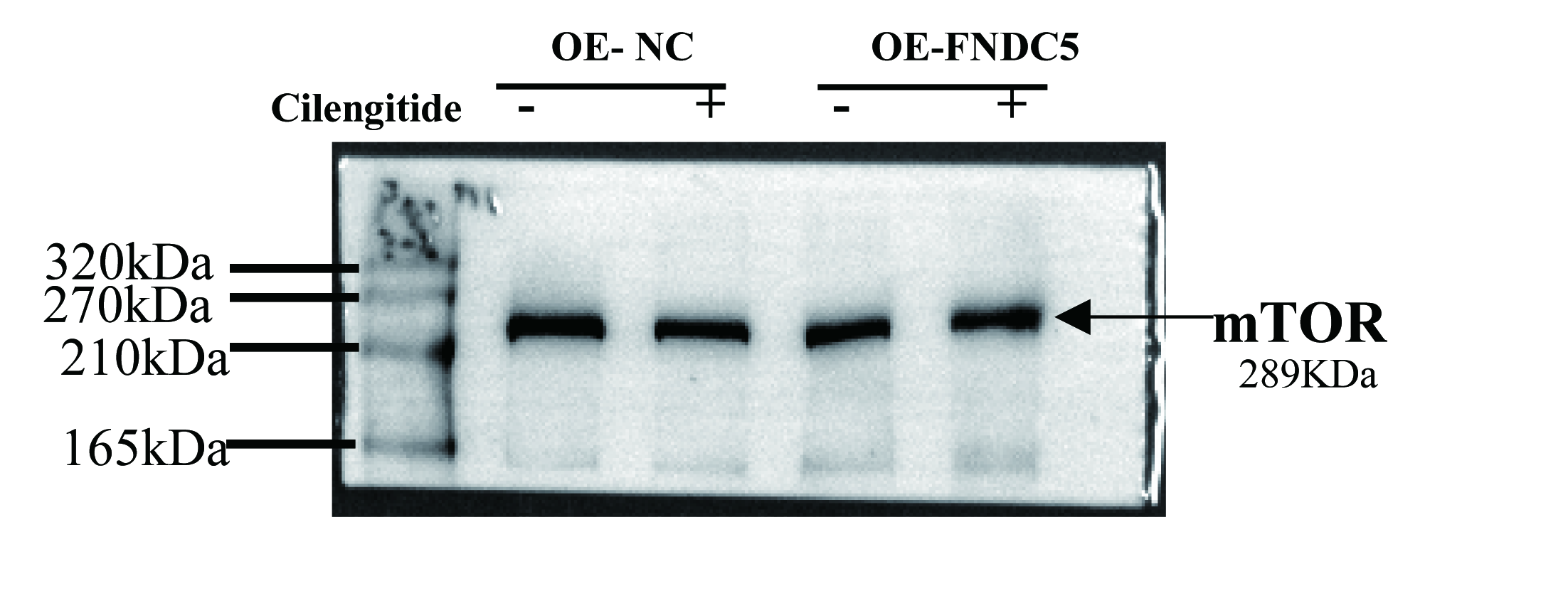

Supplement: Supplementary file 5 — Supplementary Material 5. [file 13395_2026_420_MOESM5_ESM.zip › Supplementary Material 5/Fig3/Fig3H/mTOR/mTOR-2.tif]

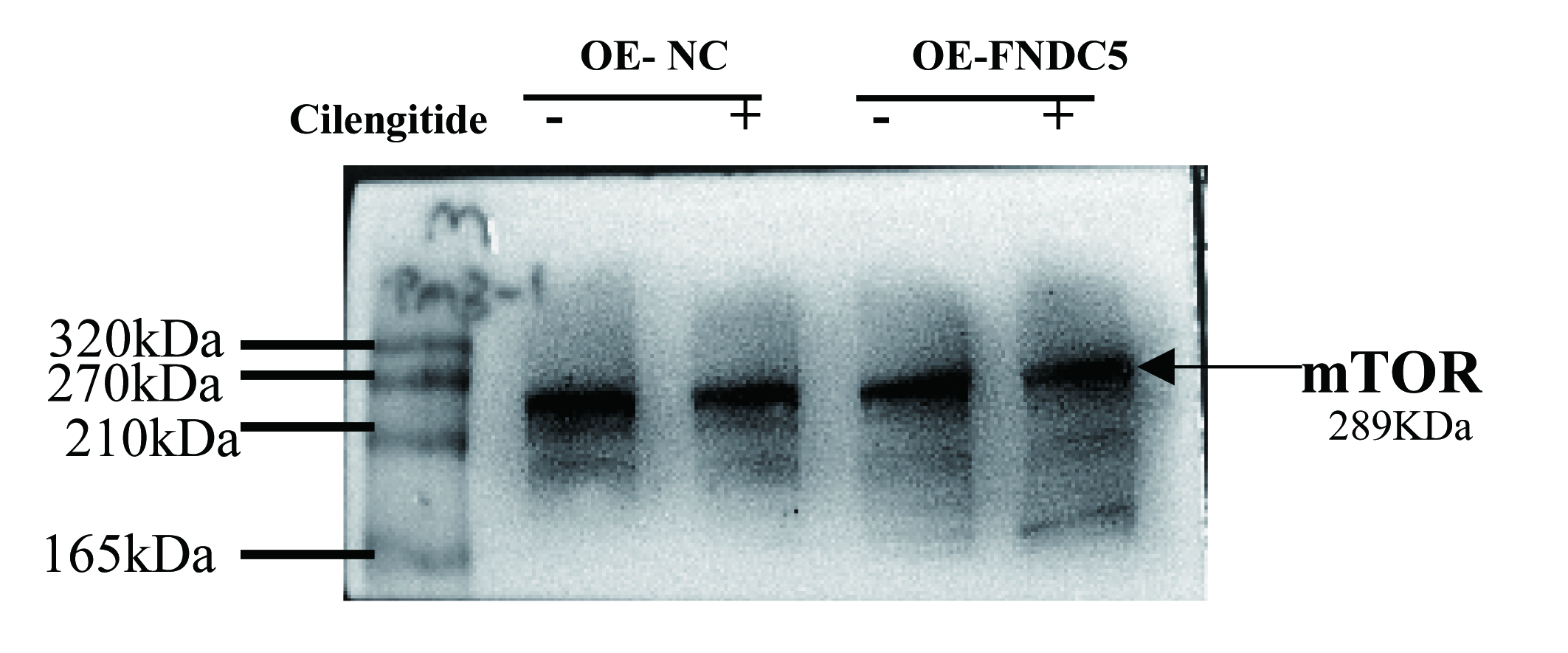

Supplement: Supplementary file 5 — Supplementary Material 5. [file 13395_2026_420_MOESM5_ESM.zip › Supplementary Material 5/Fig3/Fig3H/mTOR/mTOR-3.tif]

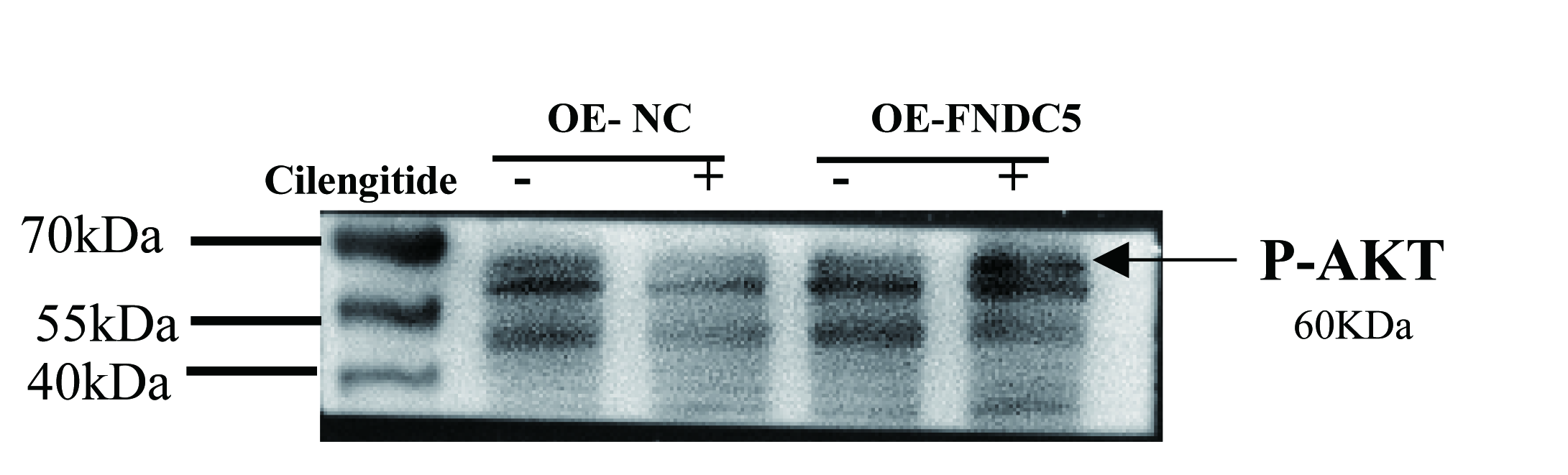

Supplement: Supplementary file 5 — Supplementary Material 5. [file 13395_2026_420_MOESM5_ESM.zip › Supplementary Material 5/Fig3/Fig3H/P-AKT/P-AKT-1.tif]

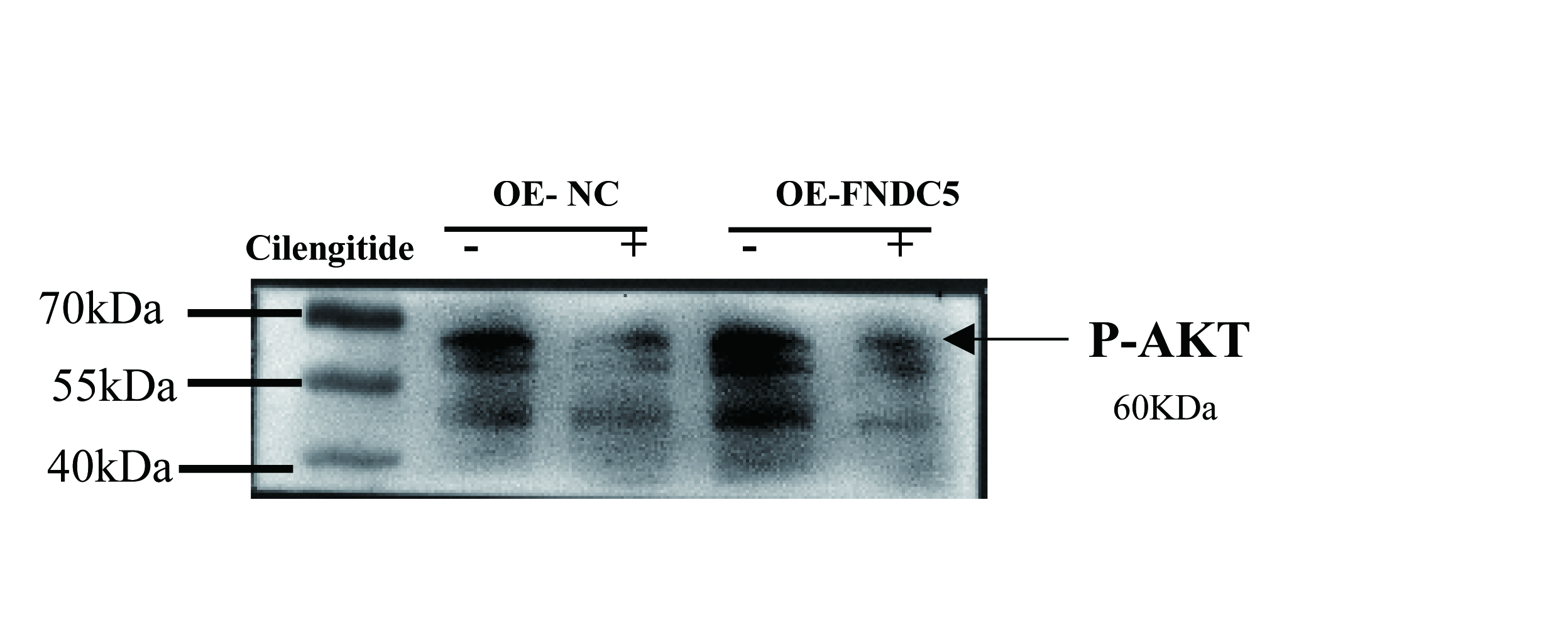

Supplement: Supplementary file 5 — Supplementary Material 5. [file 13395_2026_420_MOESM5_ESM.zip › Supplementary Material 5/Fig3/Fig3H/P-AKT/P-AKT-2.tif]

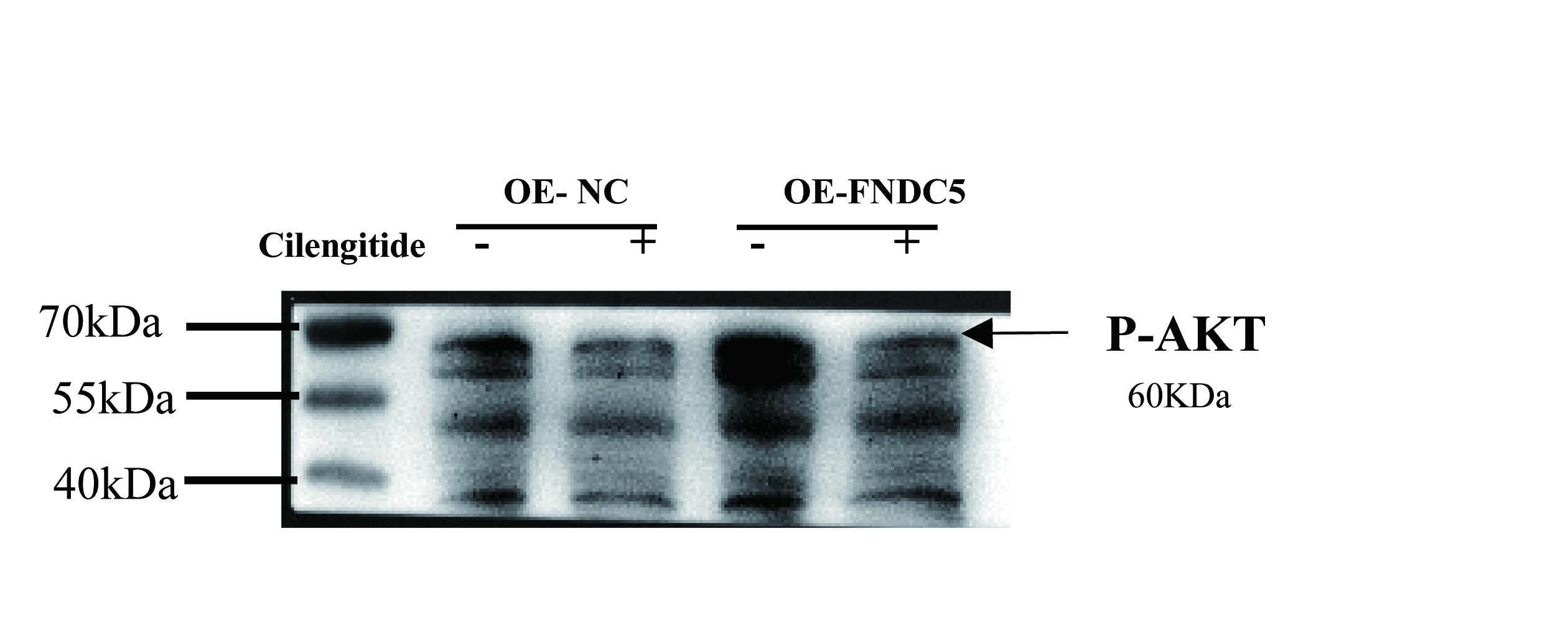

Supplement: Supplementary file 5 — Supplementary Material 5. [file 13395_2026_420_MOESM5_ESM.zip › Supplementary Material 5/Fig3/Fig3H/P-AKT/P-AKT-3.tif]

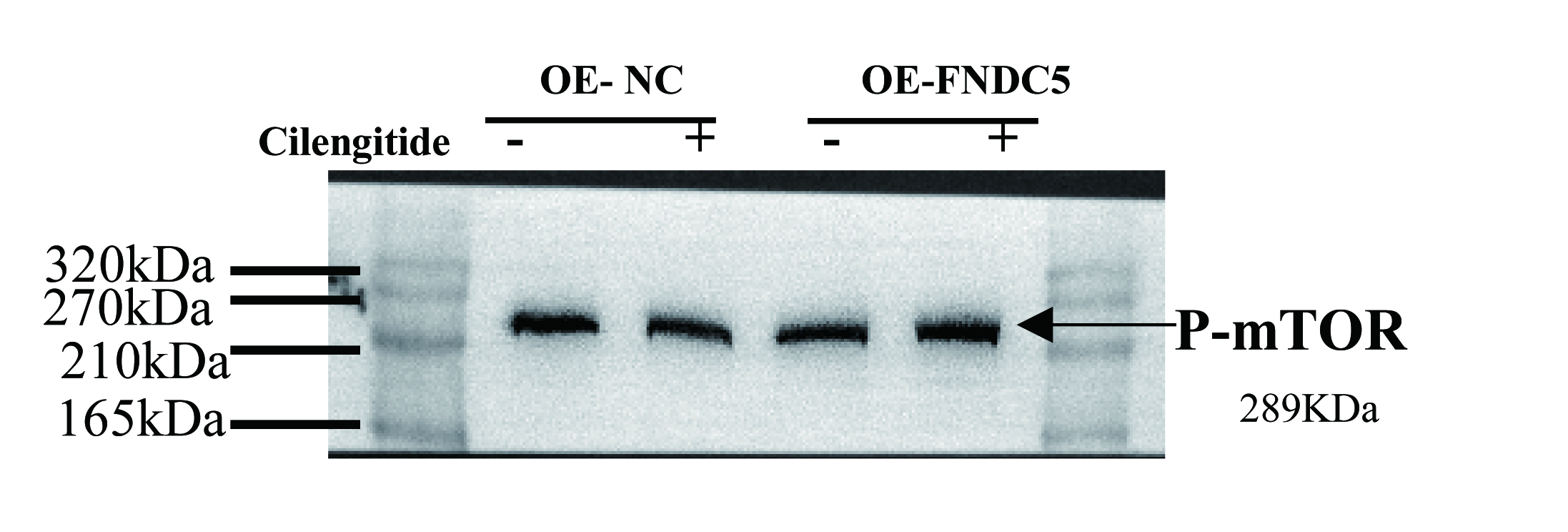

Supplement: Supplementary file 5 — Supplementary Material 5. [file 13395_2026_420_MOESM5_ESM.zip › Supplementary Material 5/Fig3/Fig3H/P-mTOR/P-mTOR-1.tif]

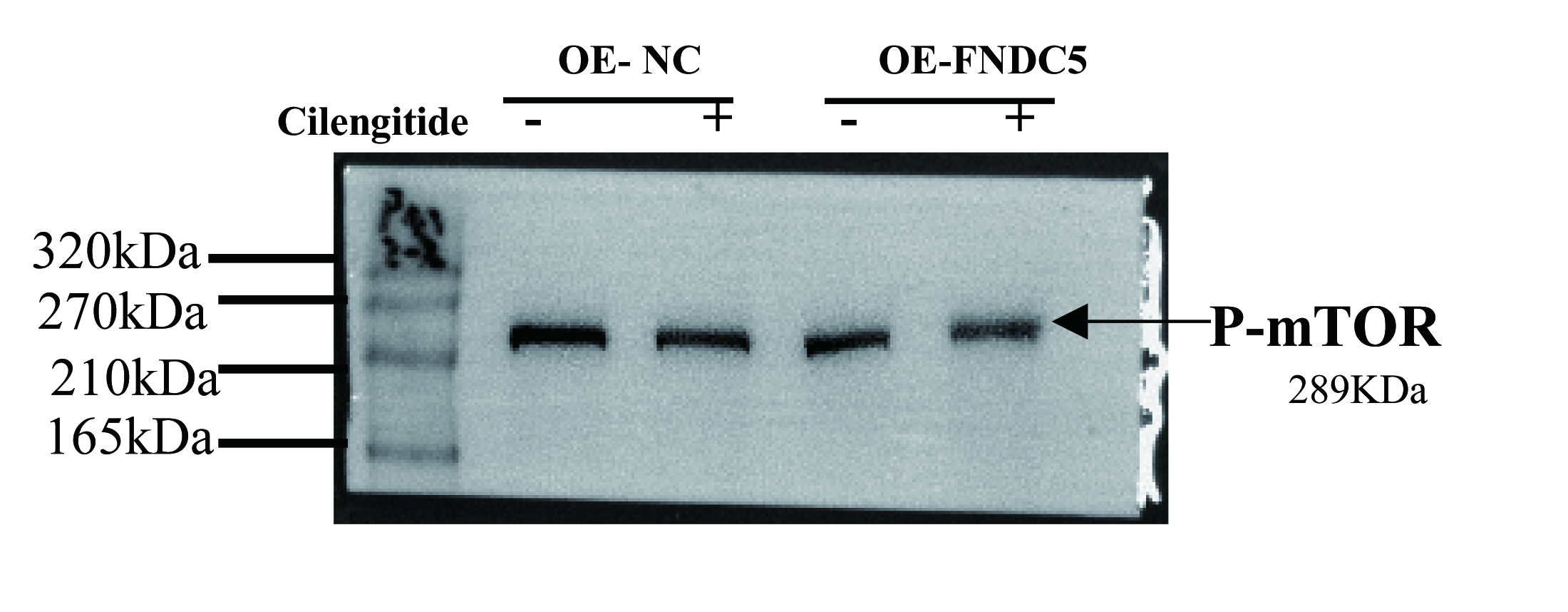

Supplement: Supplementary file 5 — Supplementary Material 5. [file 13395_2026_420_MOESM5_ESM.zip › Supplementary Material 5/Fig3/Fig3H/P-mTOR/P-mTOR-2.tif]

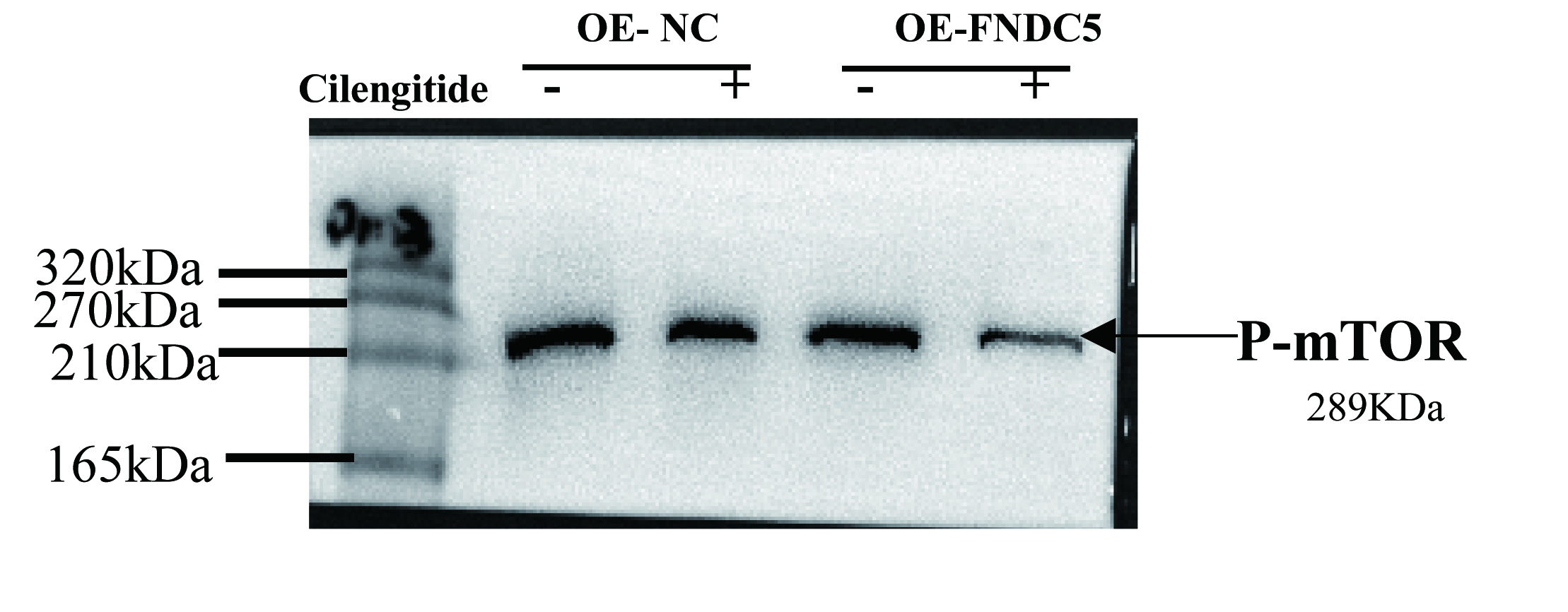

Supplement: Supplementary file 5 — Supplementary Material 5. [file 13395_2026_420_MOESM5_ESM.zip › Supplementary Material 5/Fig3/Fig3H/P-mTOR/P-mTOR-3.tif]

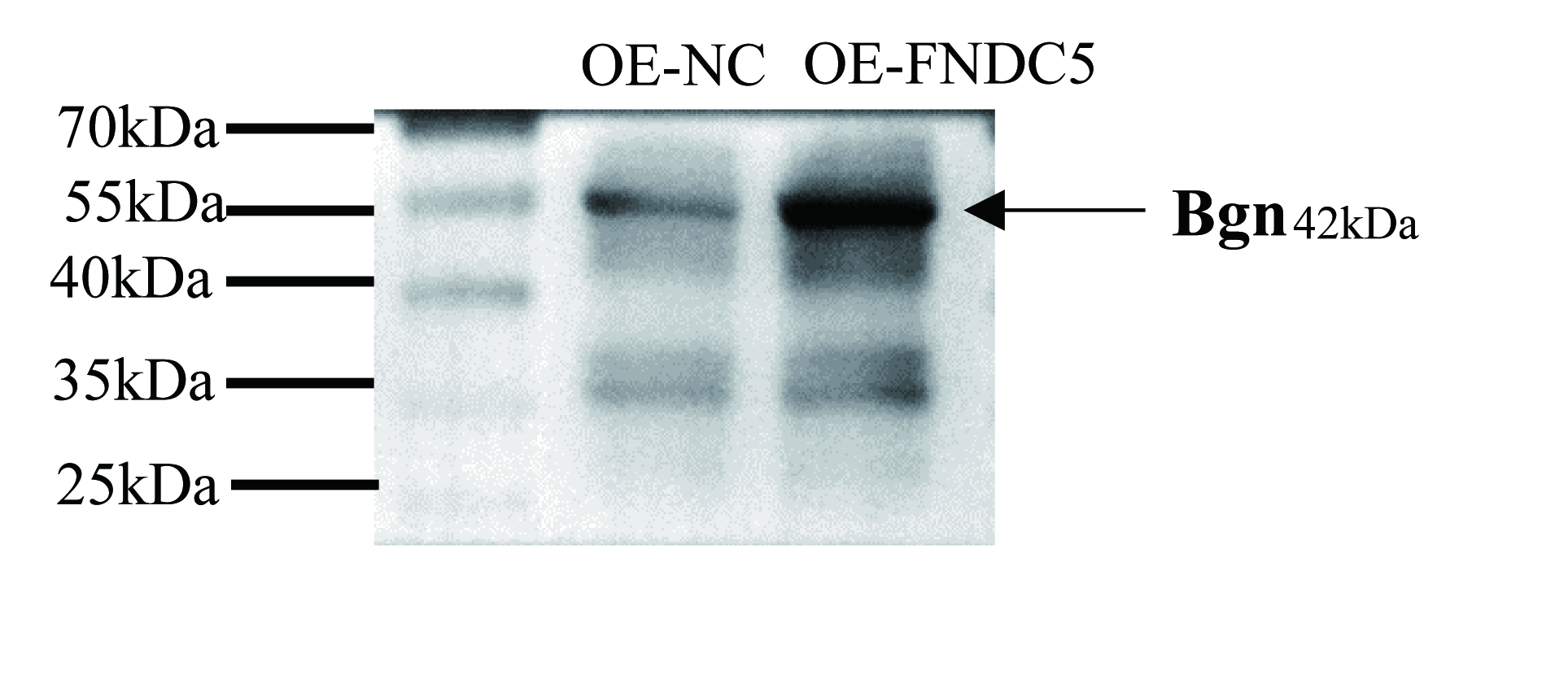

Supplement: Supplementary file 5 — Supplementary Material 5. [file 13395_2026_420_MOESM5_ESM.zip › Supplementary Material 5/Fig4/Fig4G/Bgn/OE-DNDC5/Bgn-1.tif]

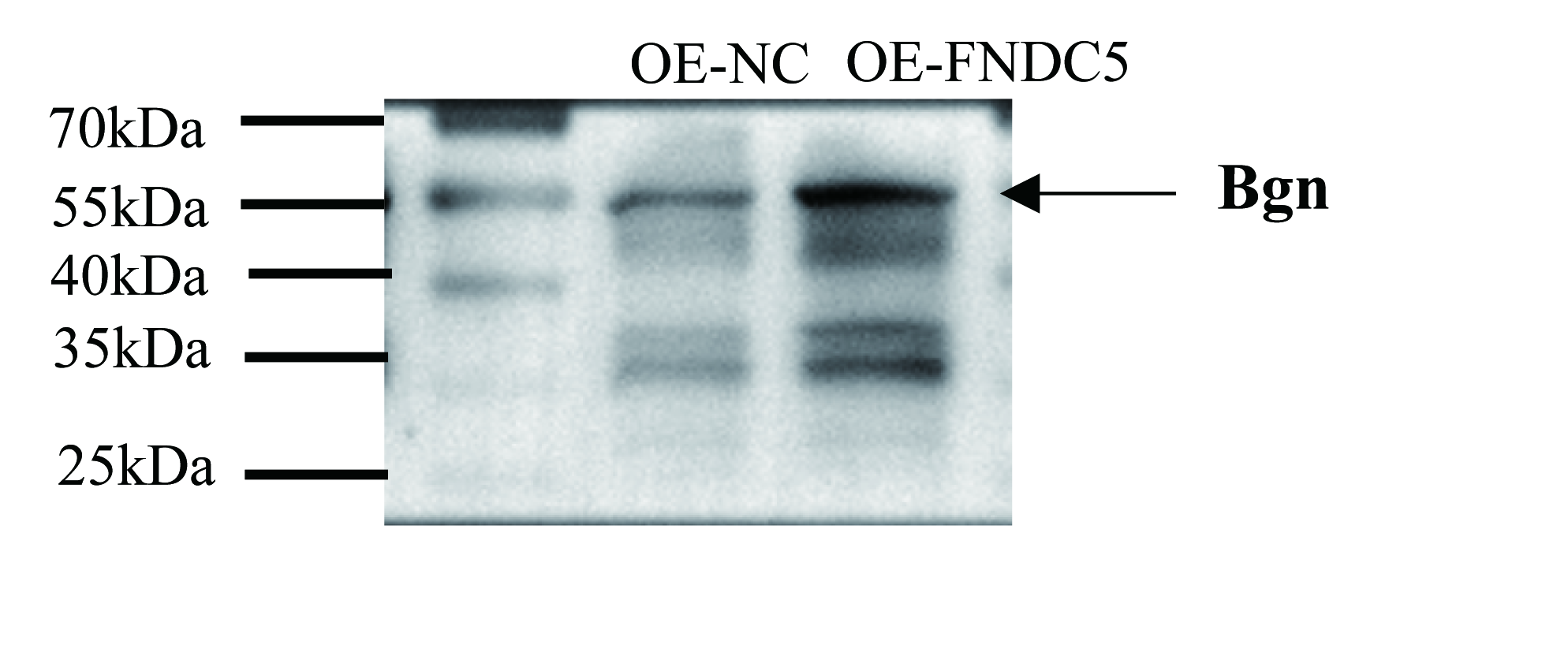

Supplement: Supplementary file 5 — Supplementary Material 5. [file 13395_2026_420_MOESM5_ESM.zip › Supplementary Material 5/Fig4/Fig4G/Bgn/OE-DNDC5/Bgn-2.tif]

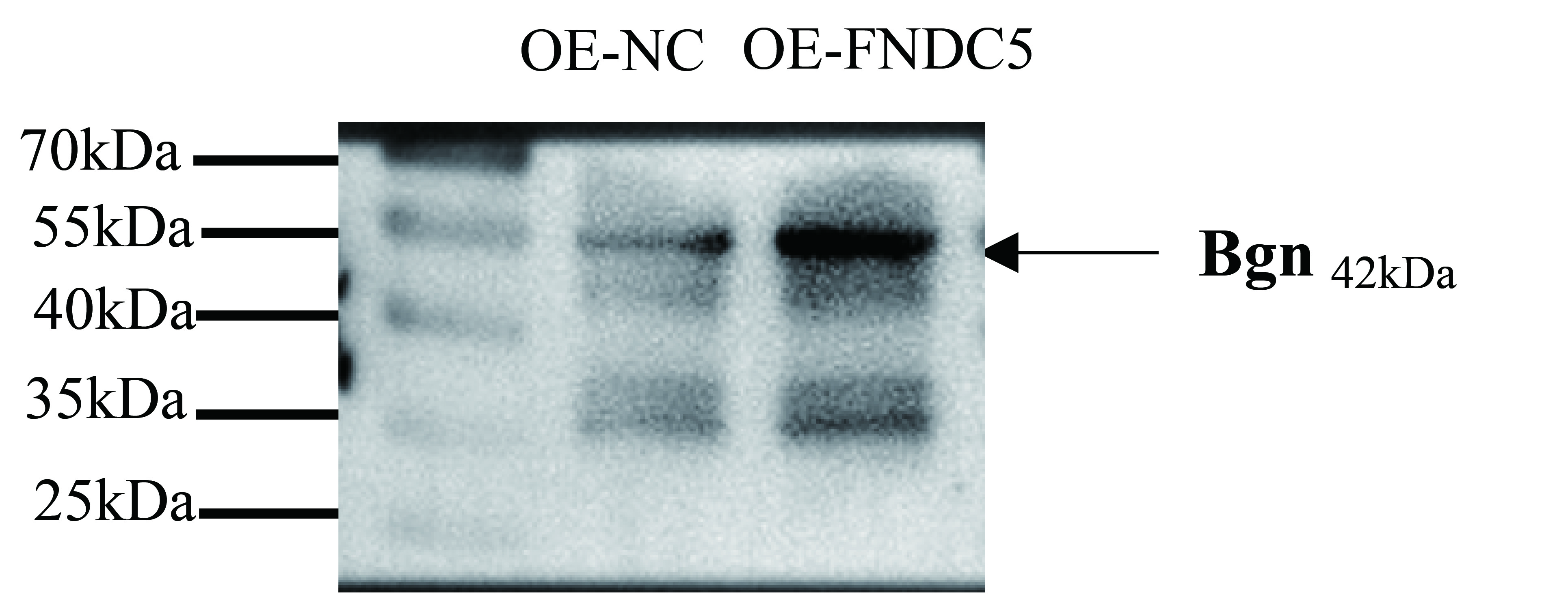

Supplement: Supplementary file 5 — Supplementary Material 5. [file 13395_2026_420_MOESM5_ESM.zip › Supplementary Material 5/Fig4/Fig4G/Bgn/OE-DNDC5/Bgn-3.jpg]

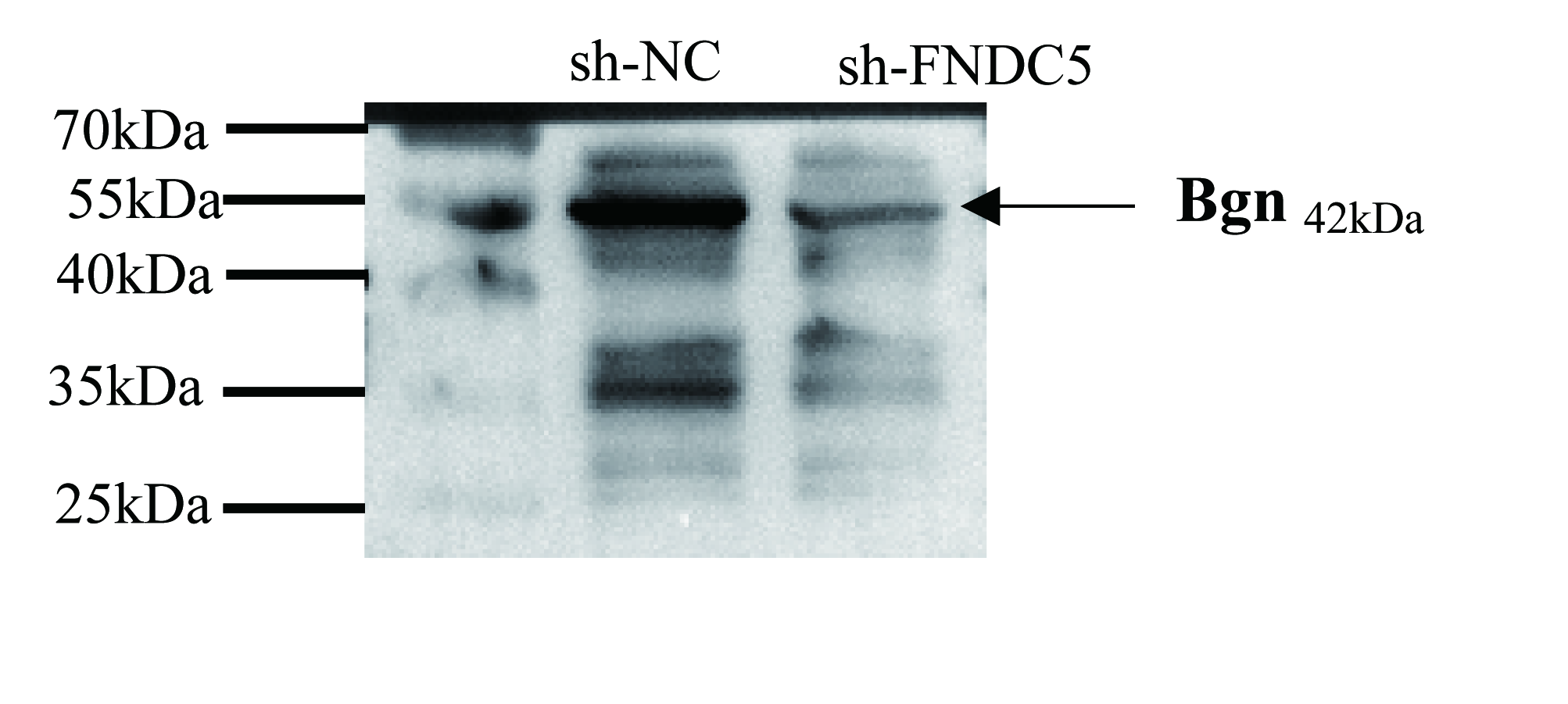

Supplement: Supplementary file 5 — Supplementary Material 5. [file 13395_2026_420_MOESM5_ESM.zip › Supplementary Material 5/Fig4/Fig4G/Bgn/sh-FNDC5/Bgn-1.tif]

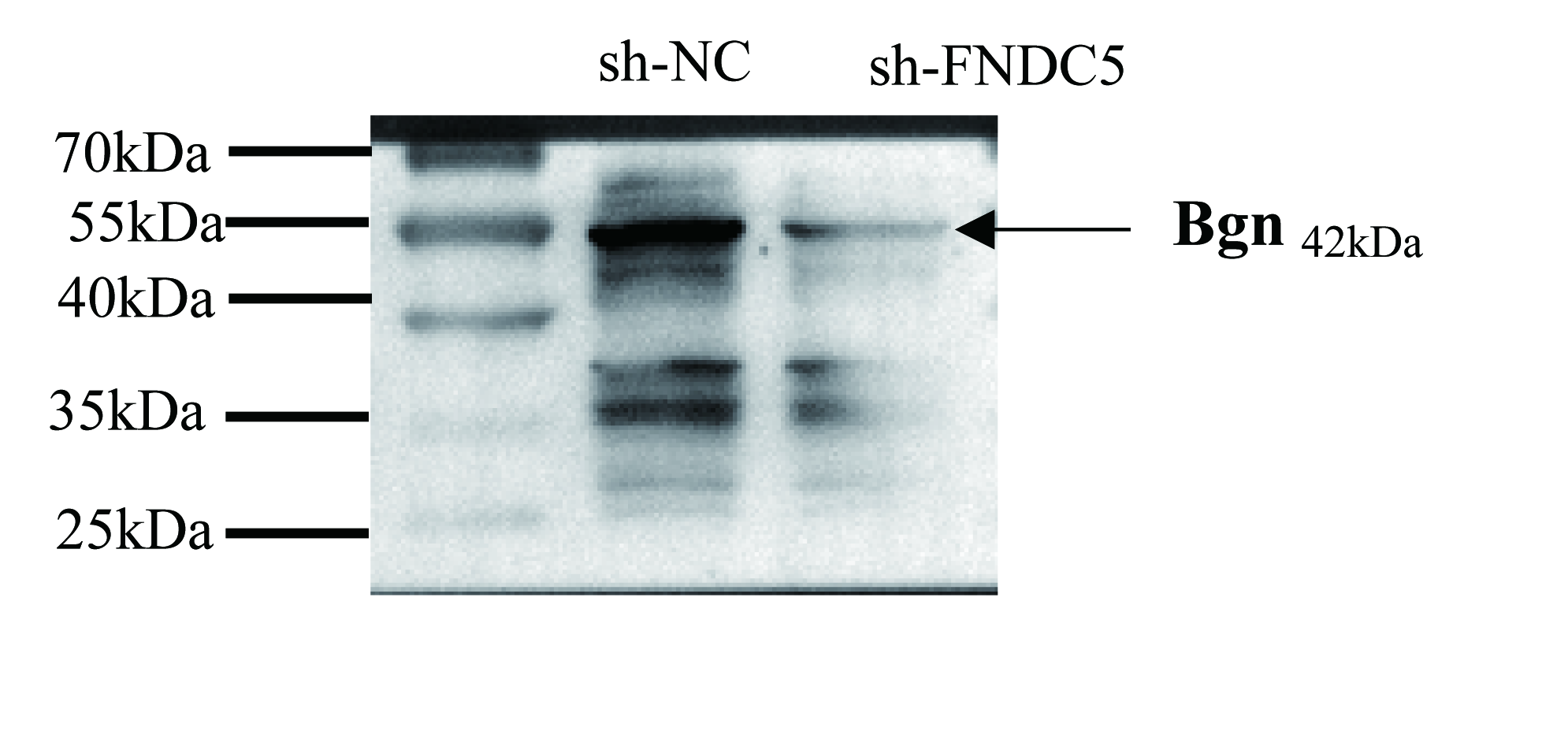

Supplement: Supplementary file 5 — Supplementary Material 5. [file 13395_2026_420_MOESM5_ESM.zip › Supplementary Material 5/Fig4/Fig4G/Bgn/sh-FNDC5/Bgn-2.tif]

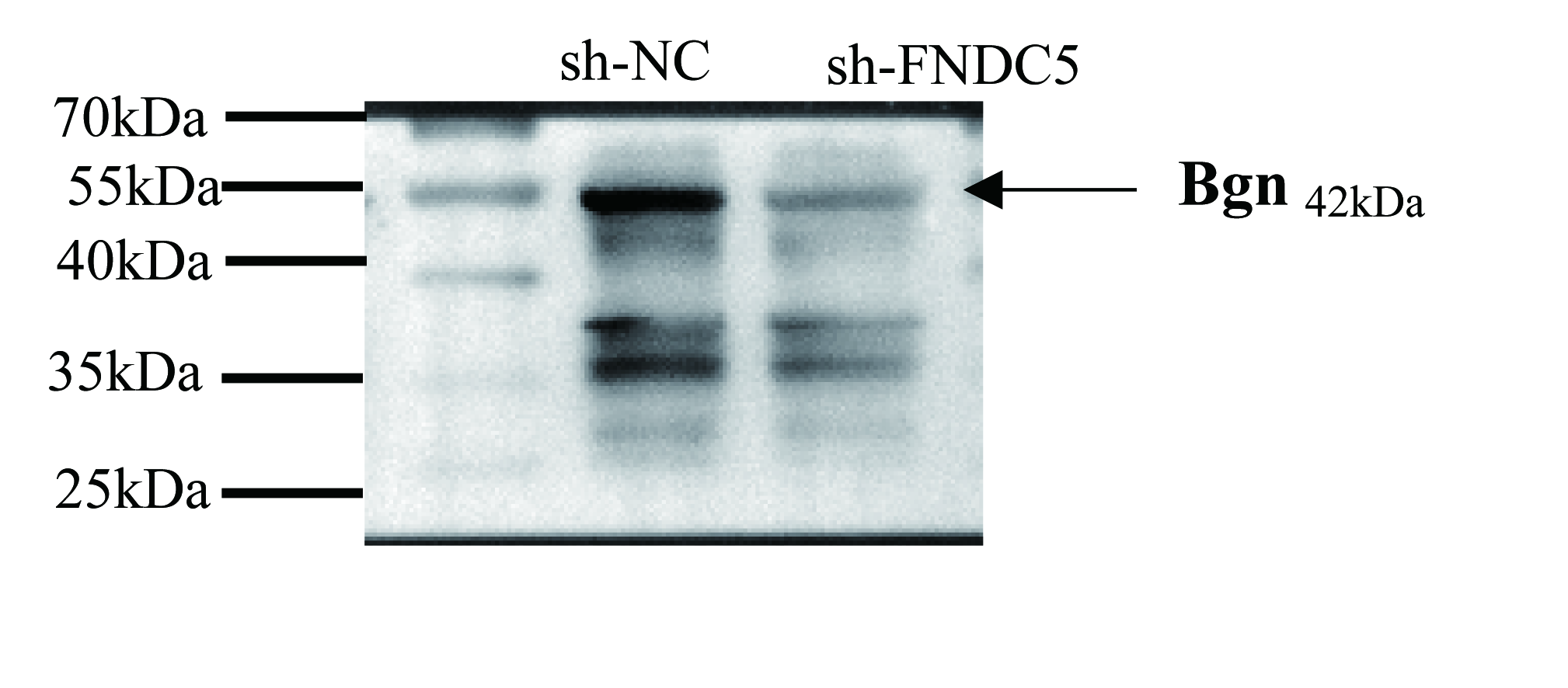

Supplement: Supplementary file 5 — Supplementary Material 5. [file 13395_2026_420_MOESM5_ESM.zip › Supplementary Material 5/Fig4/Fig4G/Bgn/sh-FNDC5/Bgn-3.tif]

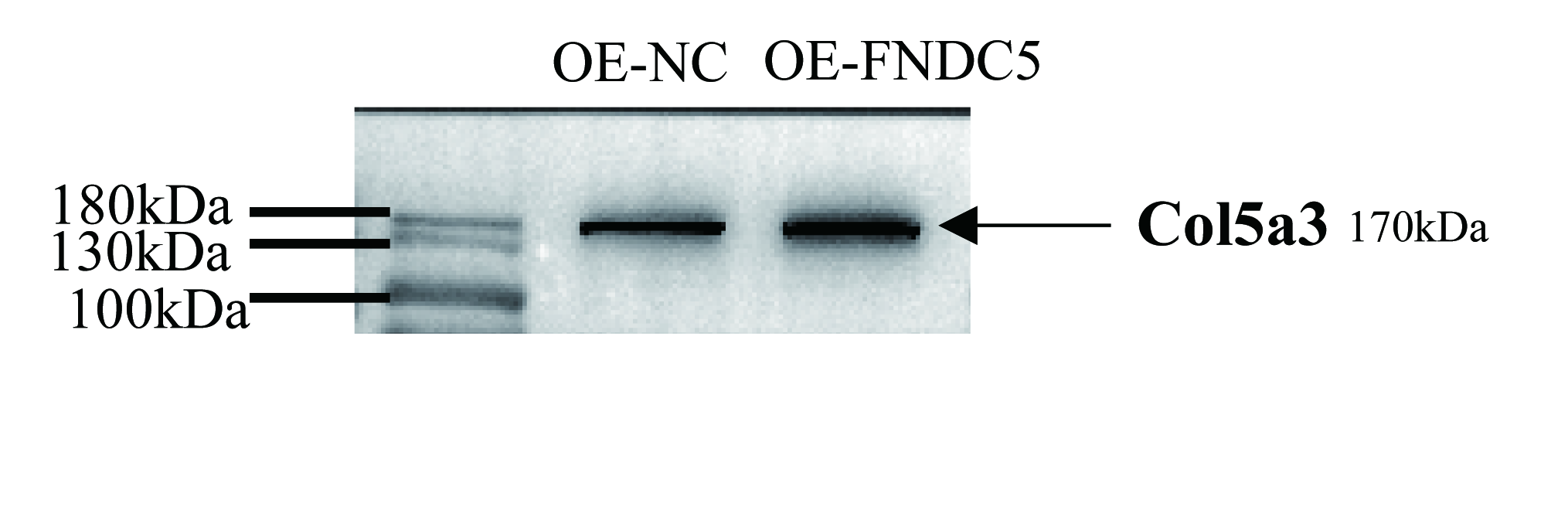

Supplement: Supplementary file 5 — Supplementary Material 5. [file 13395_2026_420_MOESM5_ESM.zip › Supplementary Material 5/Fig4/Fig4G/Col5a3/OE-DNDC5/Col5a3-1.tif]

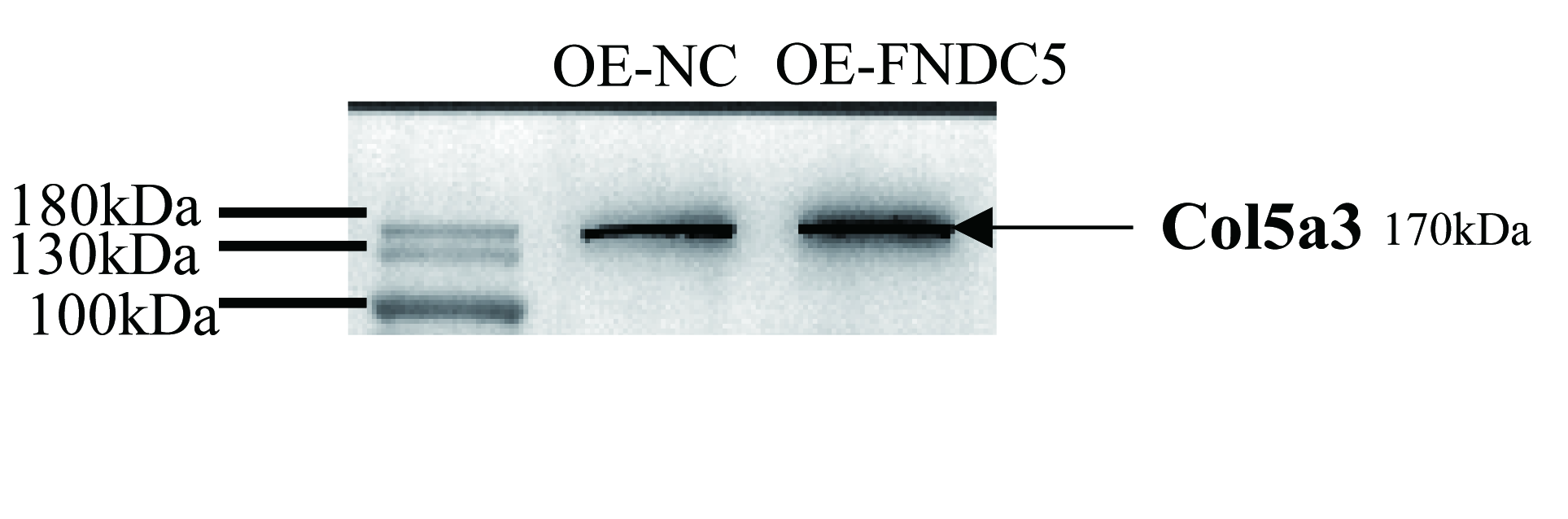

Supplement: Supplementary file 5 — Supplementary Material 5. [file 13395_2026_420_MOESM5_ESM.zip › Supplementary Material 5/Fig4/Fig4G/Col5a3/OE-DNDC5/Col5a3-2.tif]

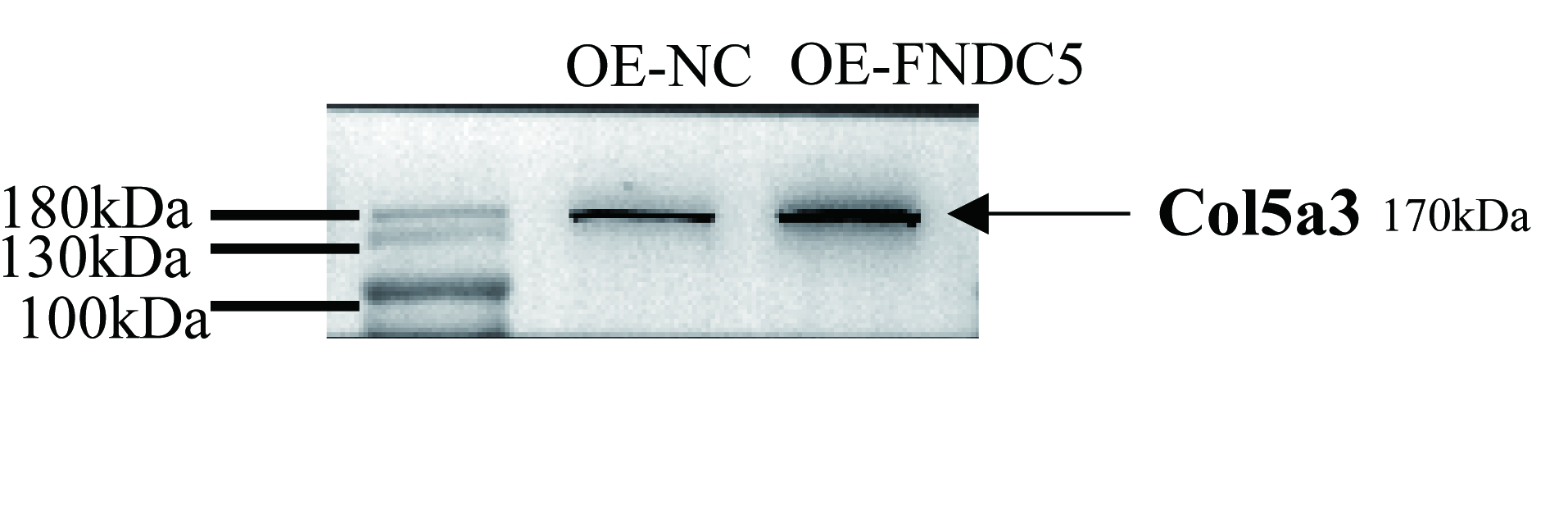

Supplement: Supplementary file 5 — Supplementary Material 5. [file 13395_2026_420_MOESM5_ESM.zip › Supplementary Material 5/Fig4/Fig4G/Col5a3/OE-DNDC5/Col5a3-3.tif]

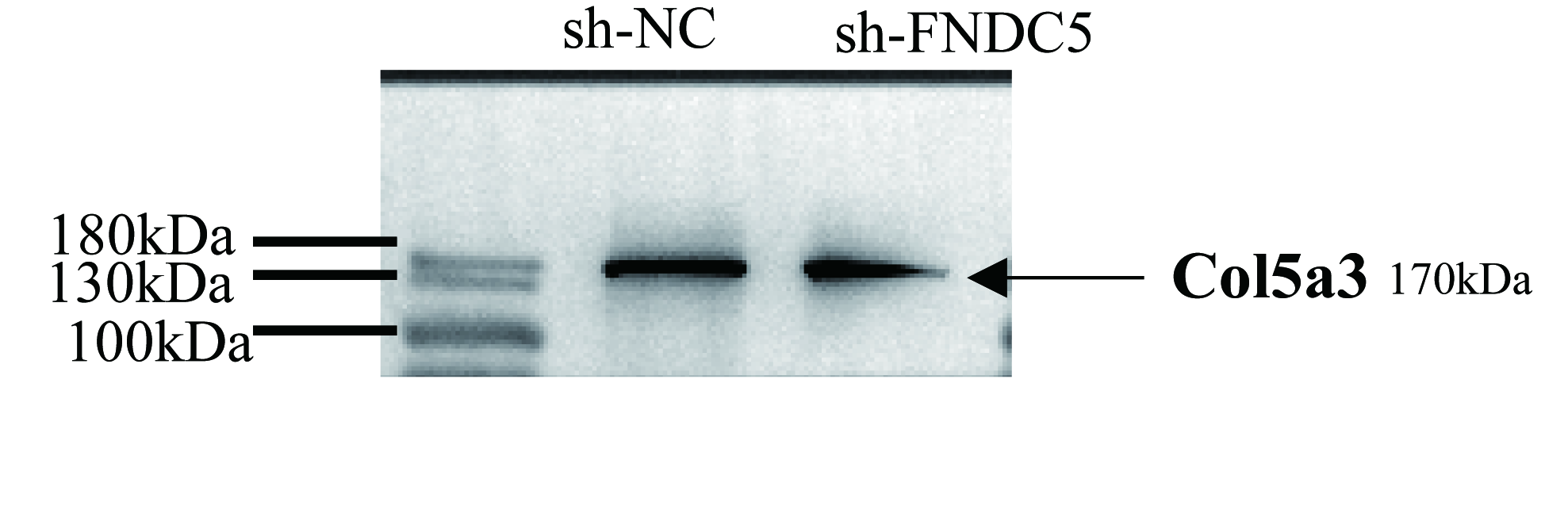

Supplement: Supplementary file 5 — Supplementary Material 5. [file 13395_2026_420_MOESM5_ESM.zip › Supplementary Material 5/Fig4/Fig4G/Col5a3/sh-FNDC5/Col5a3-1.tif]

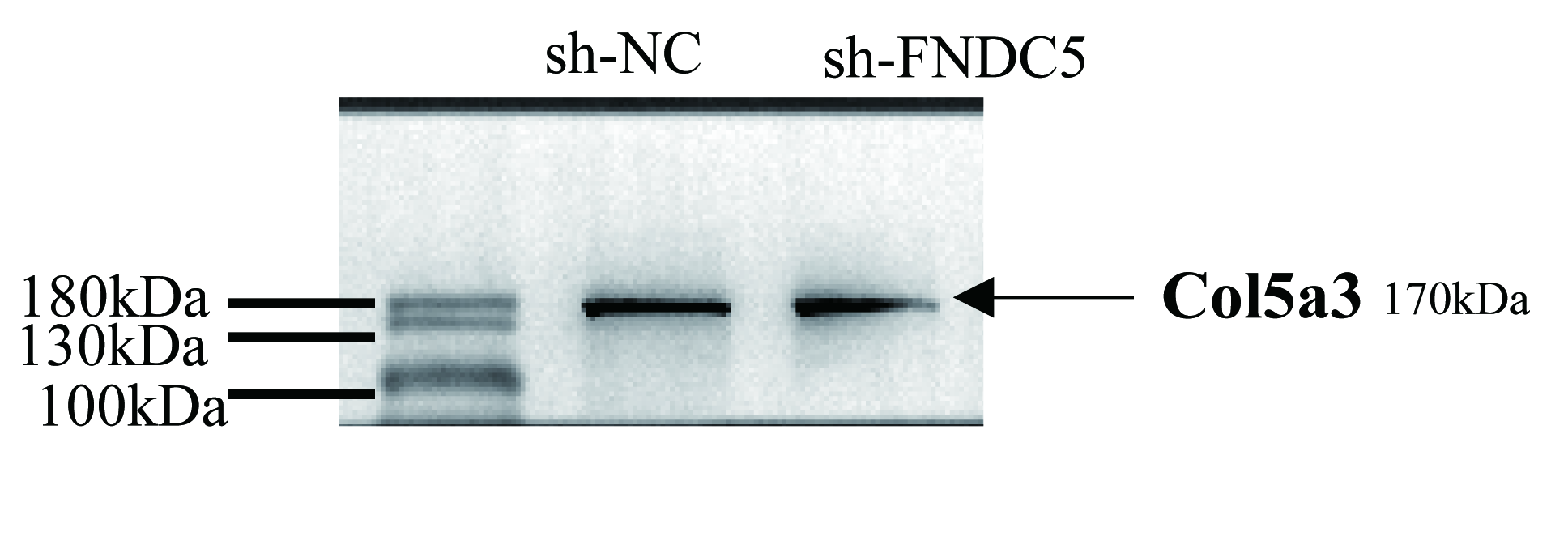

Supplement: Supplementary file 5 — Supplementary Material 5. [file 13395_2026_420_MOESM5_ESM.zip › Supplementary Material 5/Fig4/Fig4G/Col5a3/sh-FNDC5/Col5a3-2.tif]

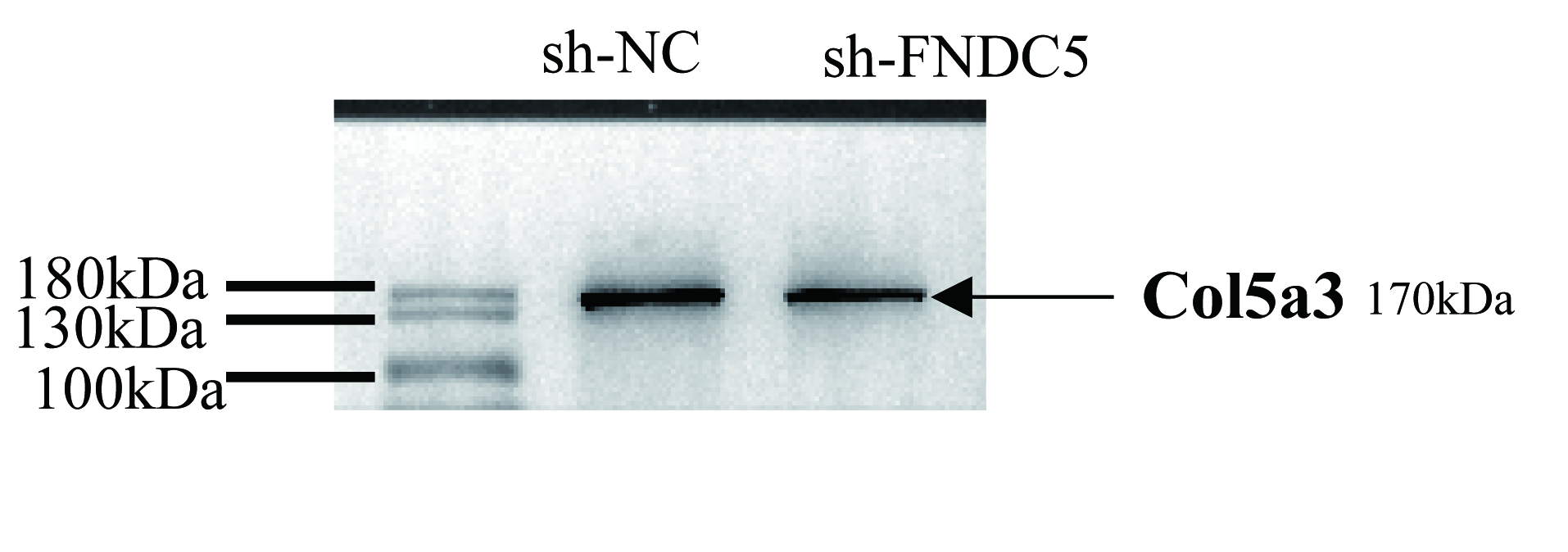

Supplement: Supplementary file 5 — Supplementary Material 5. [file 13395_2026_420_MOESM5_ESM.zip › Supplementary Material 5/Fig4/Fig4G/Col5a3/sh-FNDC5/Col5a3-3.tif]

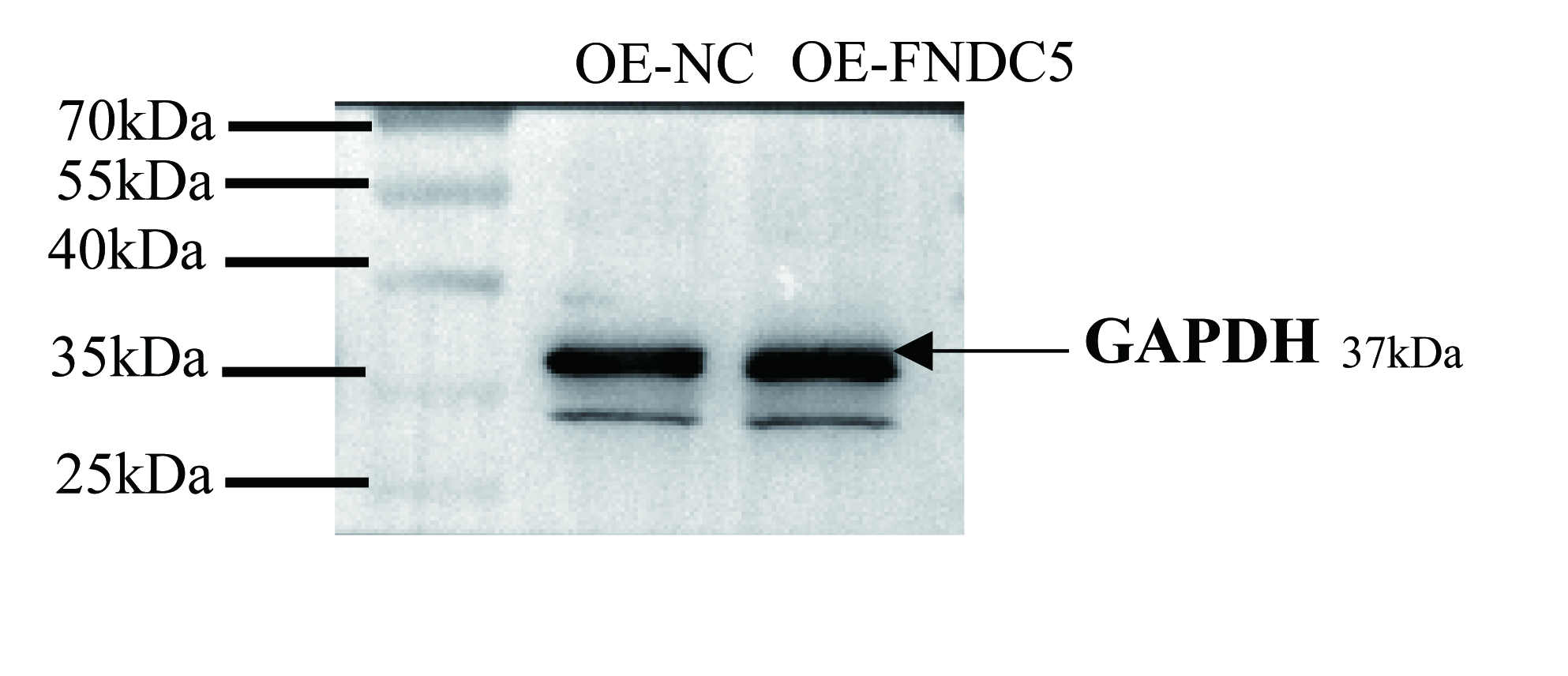

Supplement: Supplementary file 5 — Supplementary Material 5. [file 13395_2026_420_MOESM5_ESM.zip › Supplementary Material 5/Fig4/Fig4G/GAPDH/OE-DNDC5/GAPDH-1.tif]

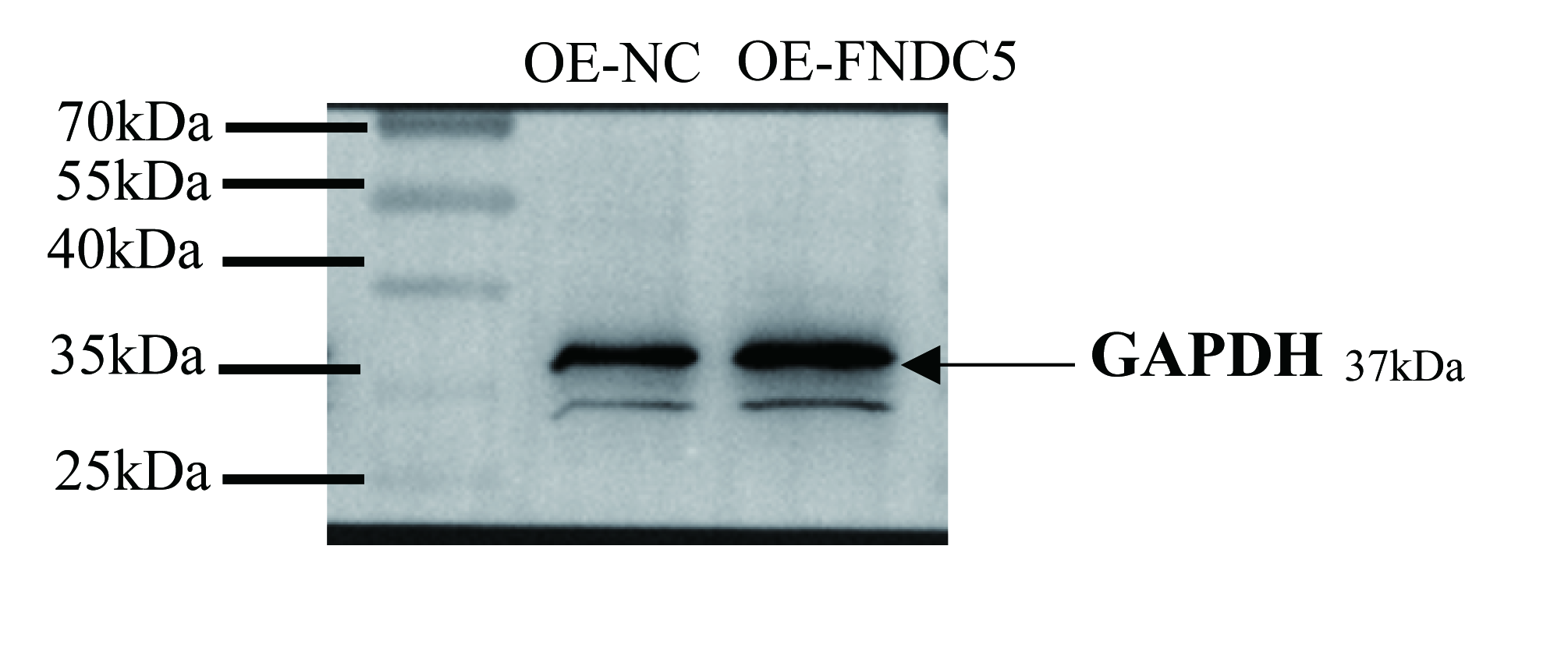

Supplement: Supplementary file 5 — Supplementary Material 5. [file 13395_2026_420_MOESM5_ESM.zip › Supplementary Material 5/Fig4/Fig4G/GAPDH/OE-DNDC5/GAPDH-2.tif]

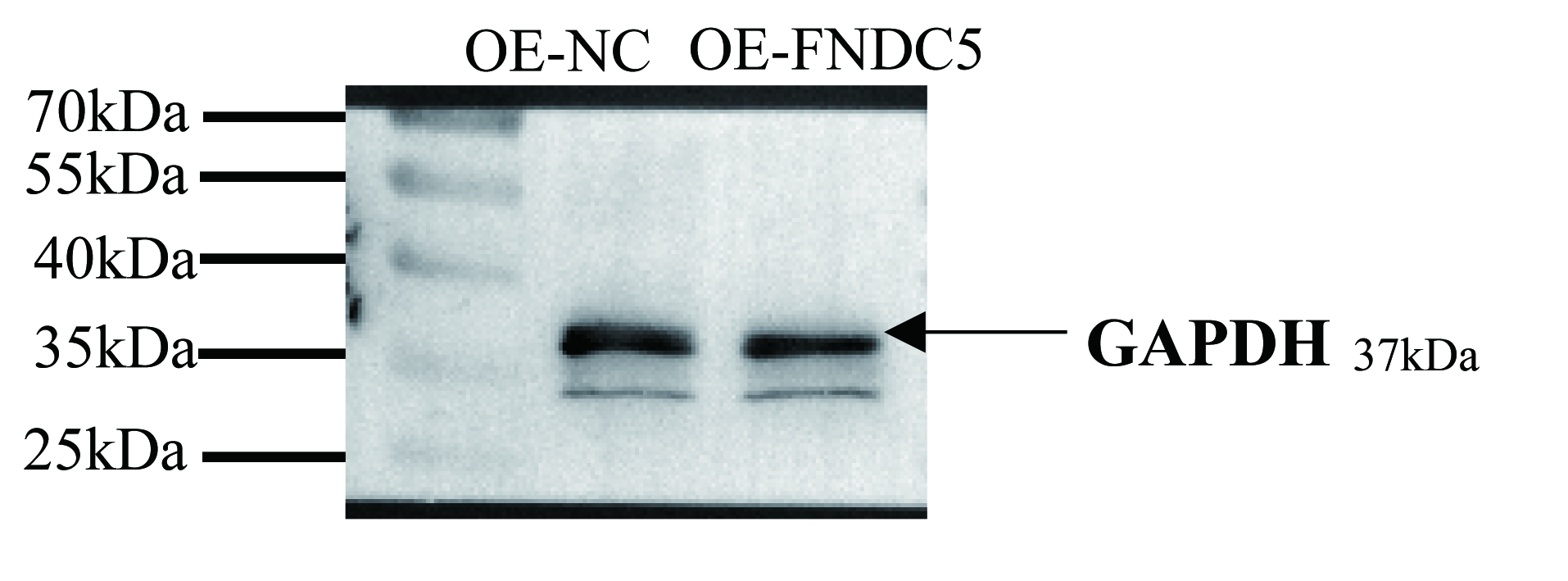

Supplement: Supplementary file 5 — Supplementary Material 5. [file 13395_2026_420_MOESM5_ESM.zip › Supplementary Material 5/Fig4/Fig4G/GAPDH/OE-DNDC5/GAPDH-3.tif]

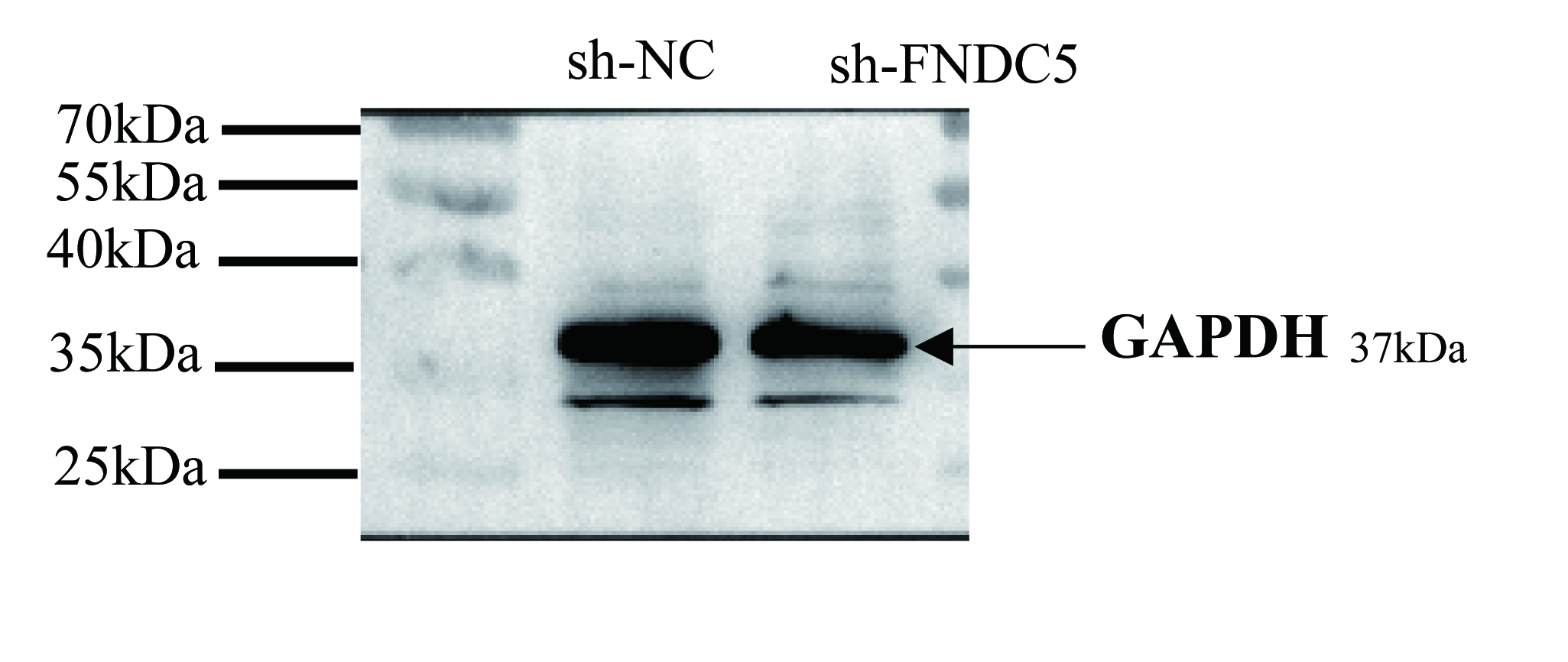

Supplement: Supplementary file 5 — Supplementary Material 5. [file 13395_2026_420_MOESM5_ESM.zip › Supplementary Material 5/Fig4/Fig4G/GAPDH/sh-FNDC5/GAPDH-1.tif]

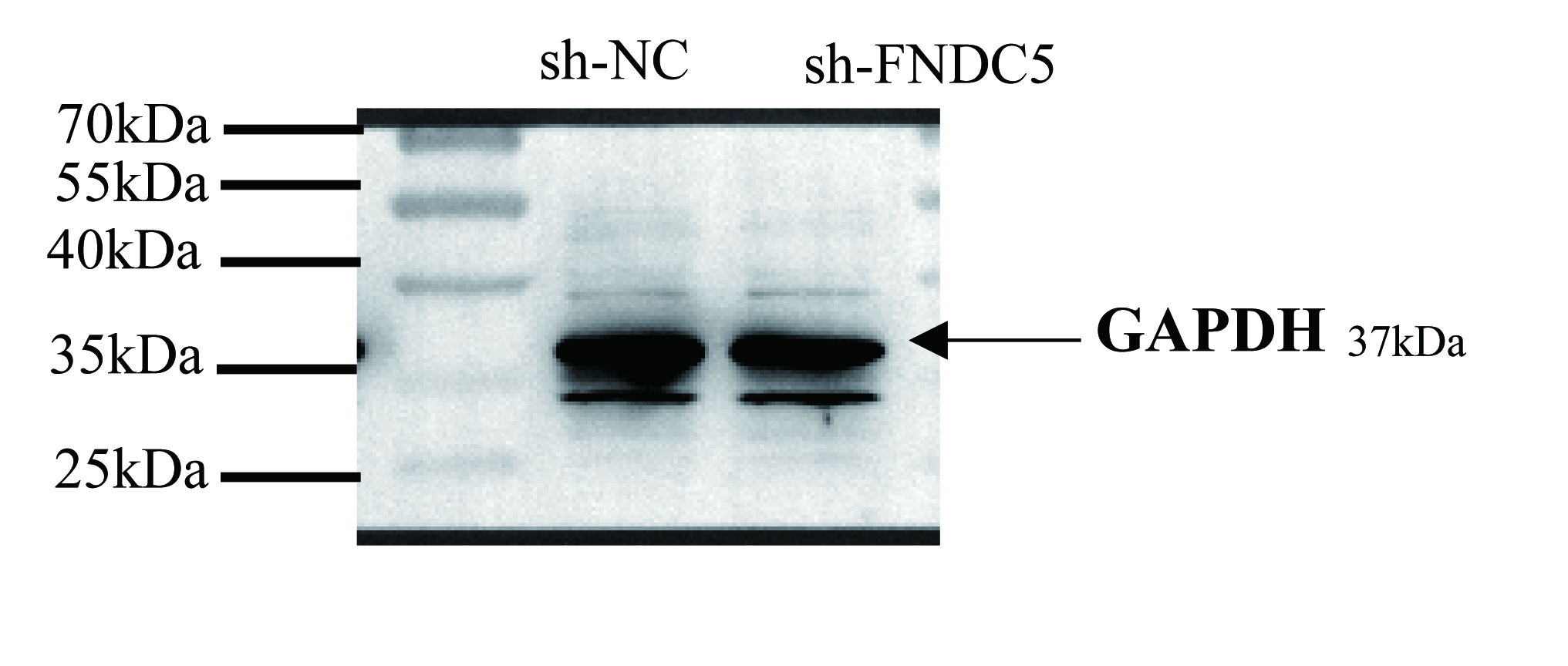

Supplement: Supplementary file 5 — Supplementary Material 5. [file 13395_2026_420_MOESM5_ESM.zip › Supplementary Material 5/Fig4/Fig4G/GAPDH/sh-FNDC5/GAPDH-2.tif]

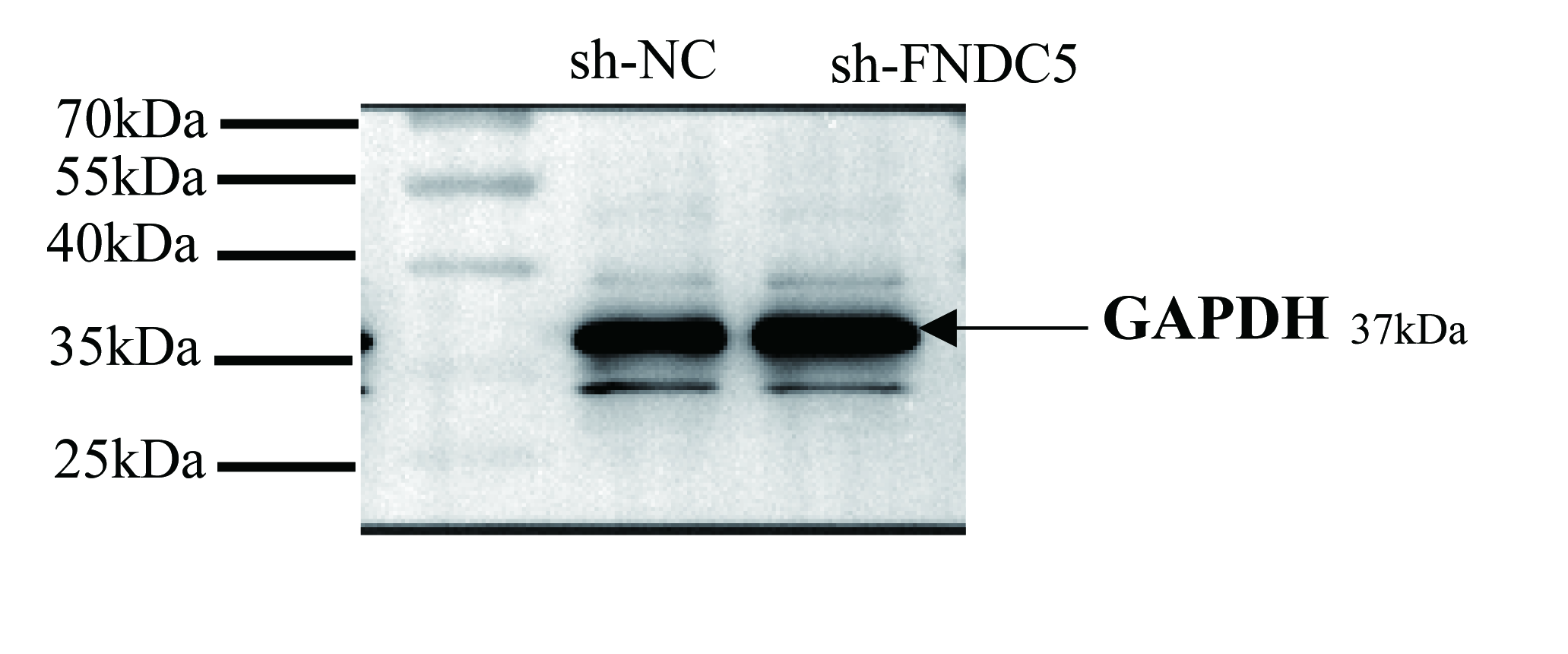

Supplement: Supplementary file 5 — Supplementary Material 5. [file 13395_2026_420_MOESM5_ESM.zip › Supplementary Material 5/Fig4/Fig4G/GAPDH/sh-FNDC5/GAPDH-3.tif]

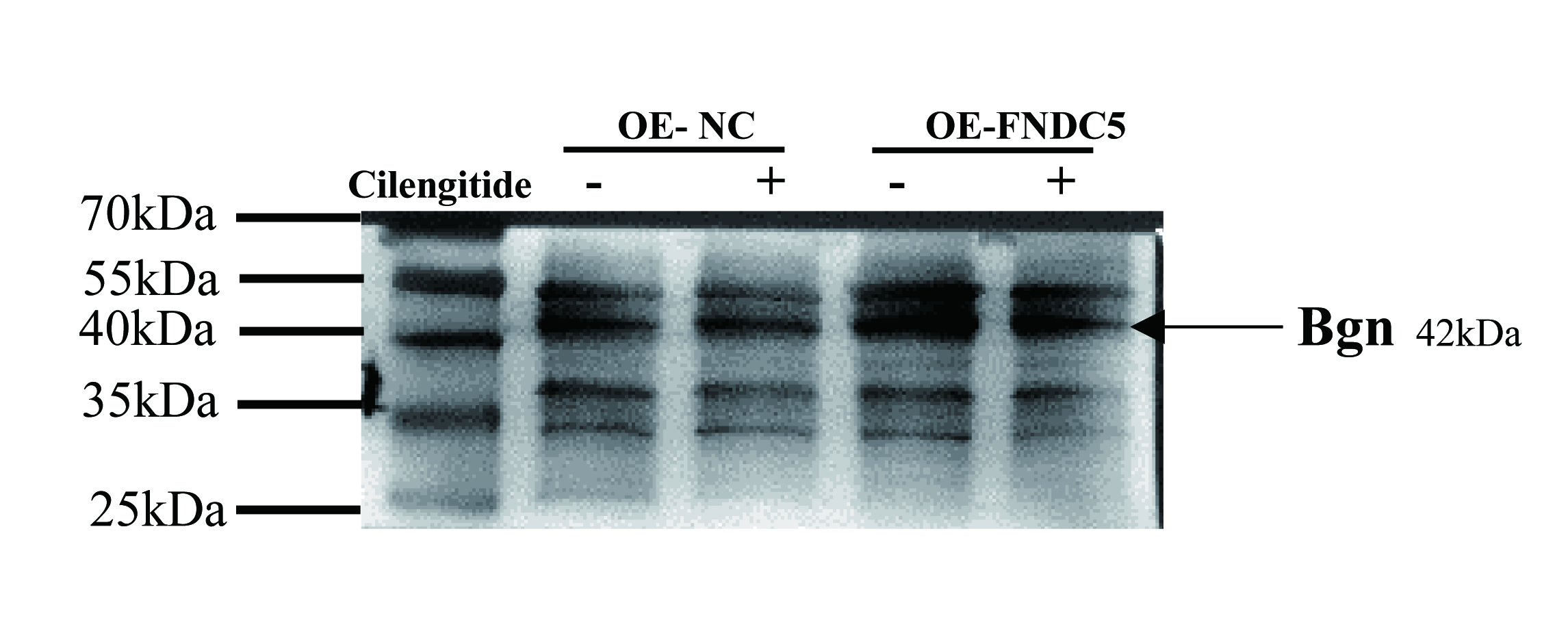

Supplement: Supplementary file 5 — Supplementary Material 5. [file 13395_2026_420_MOESM5_ESM.zip › Supplementary Material 5/Fig4/Fig4H/Bgn/Bgn-1.tif]

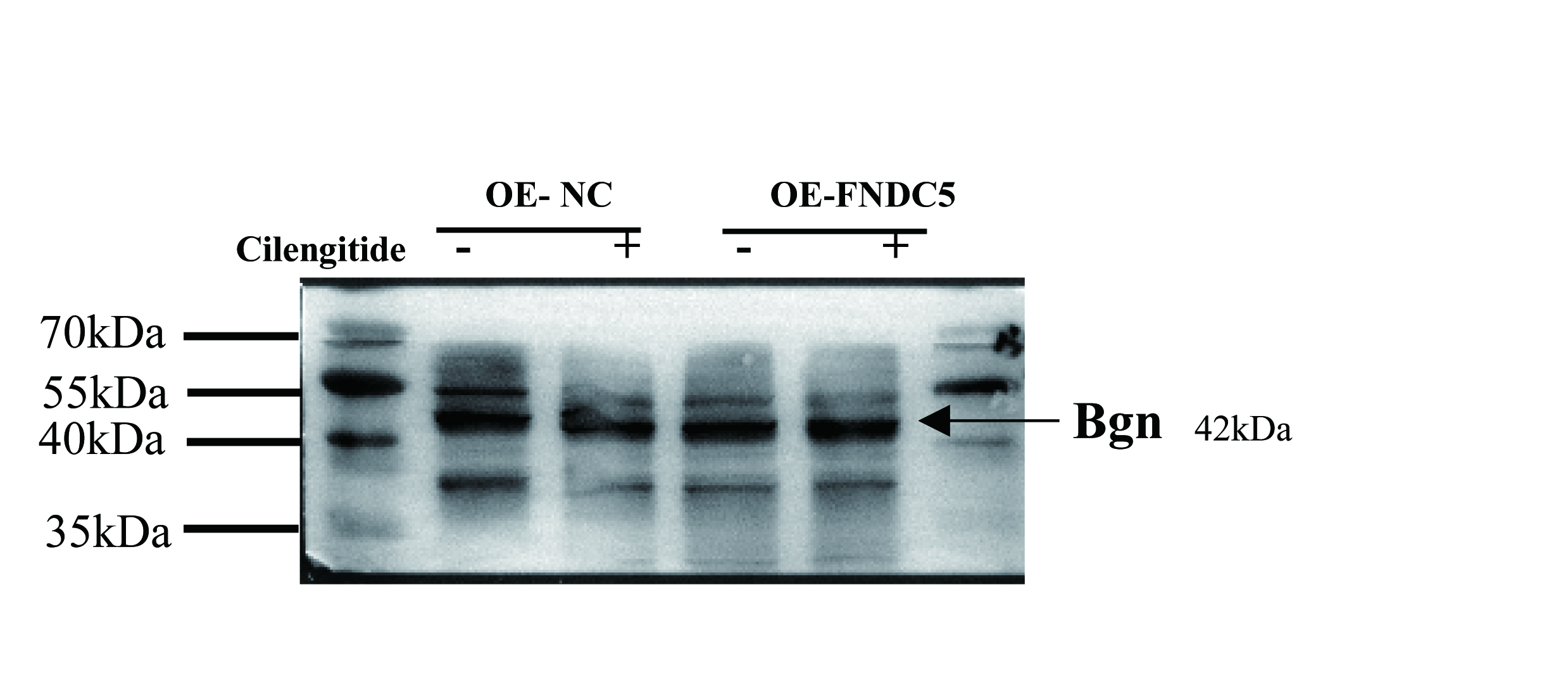

Supplement: Supplementary file 5 — Supplementary Material 5. [file 13395_2026_420_MOESM5_ESM.zip › Supplementary Material 5/Fig4/Fig4H/Bgn/Bgn-2.tif]

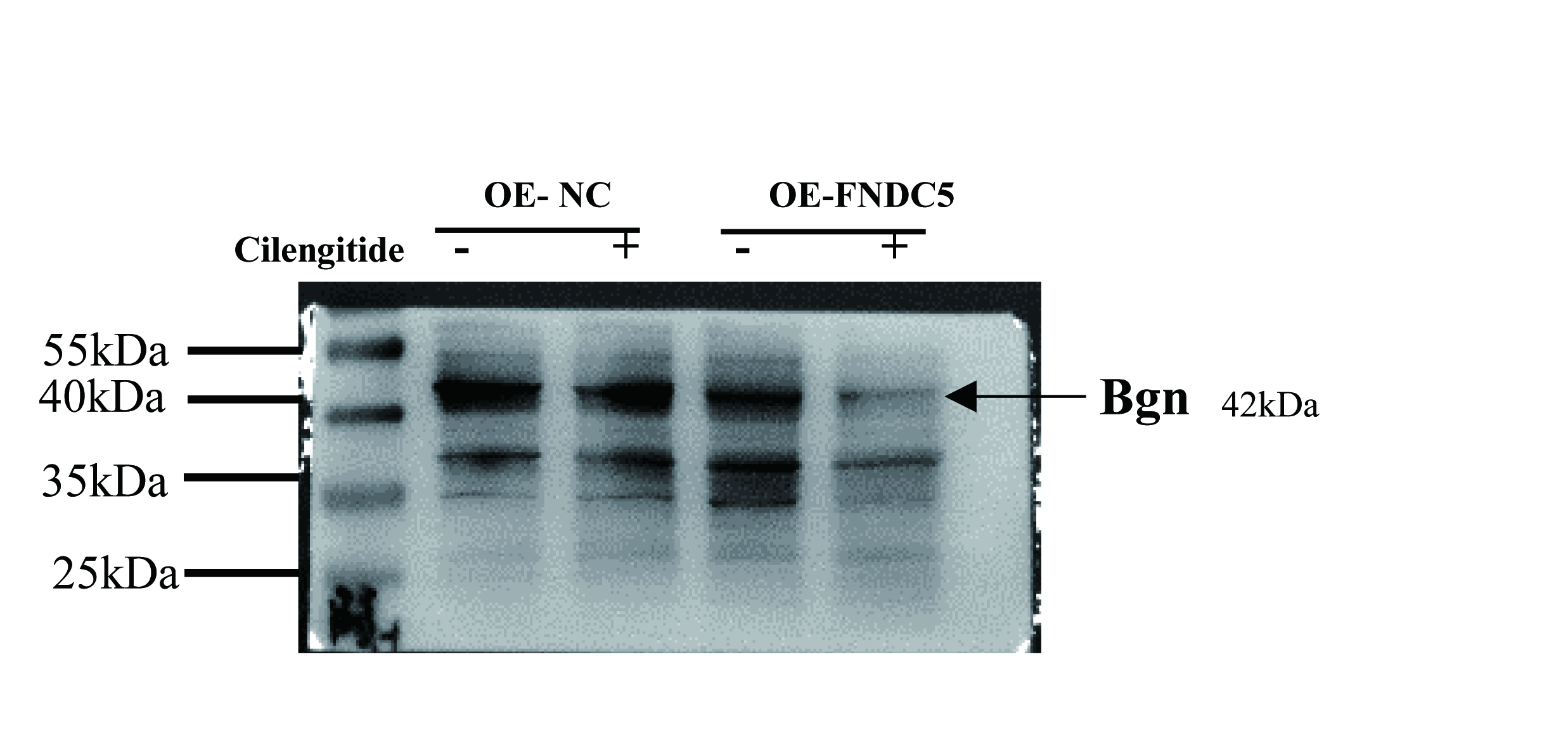

Supplement: Supplementary file 5 — Supplementary Material 5. [file 13395_2026_420_MOESM5_ESM.zip › Supplementary Material 5/Fig4/Fig4H/Bgn/Bgn-3.tif]

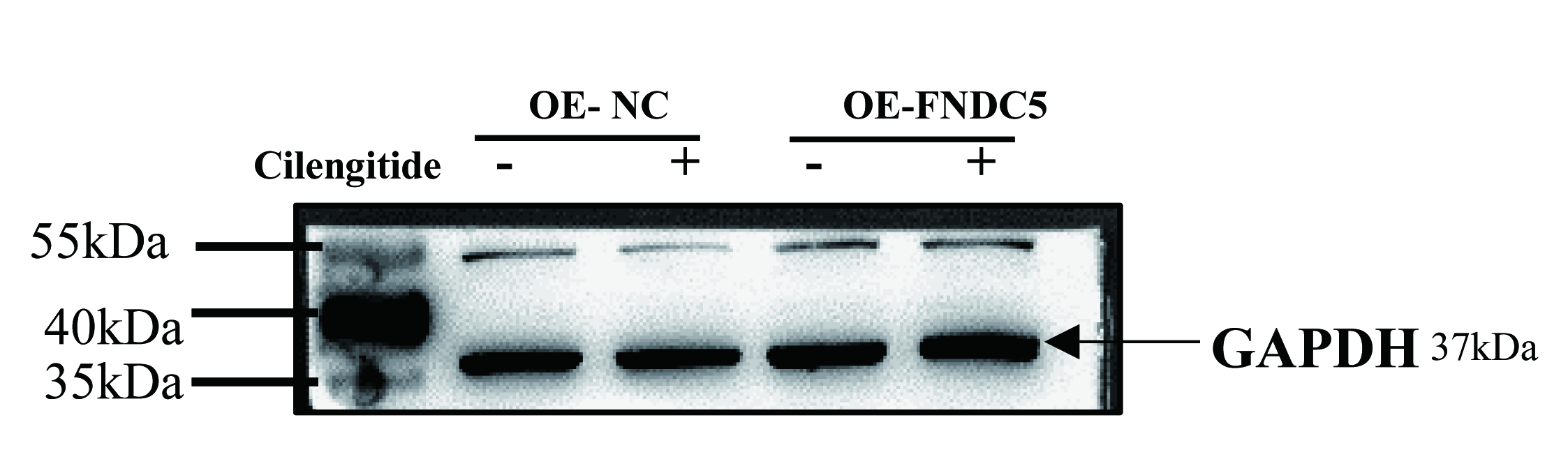

Supplement: Supplementary file 5 — Supplementary Material 5. [file 13395_2026_420_MOESM5_ESM.zip › Supplementary Material 5/Fig4/Fig4H/GAPDH/GAPDH-1.tif]

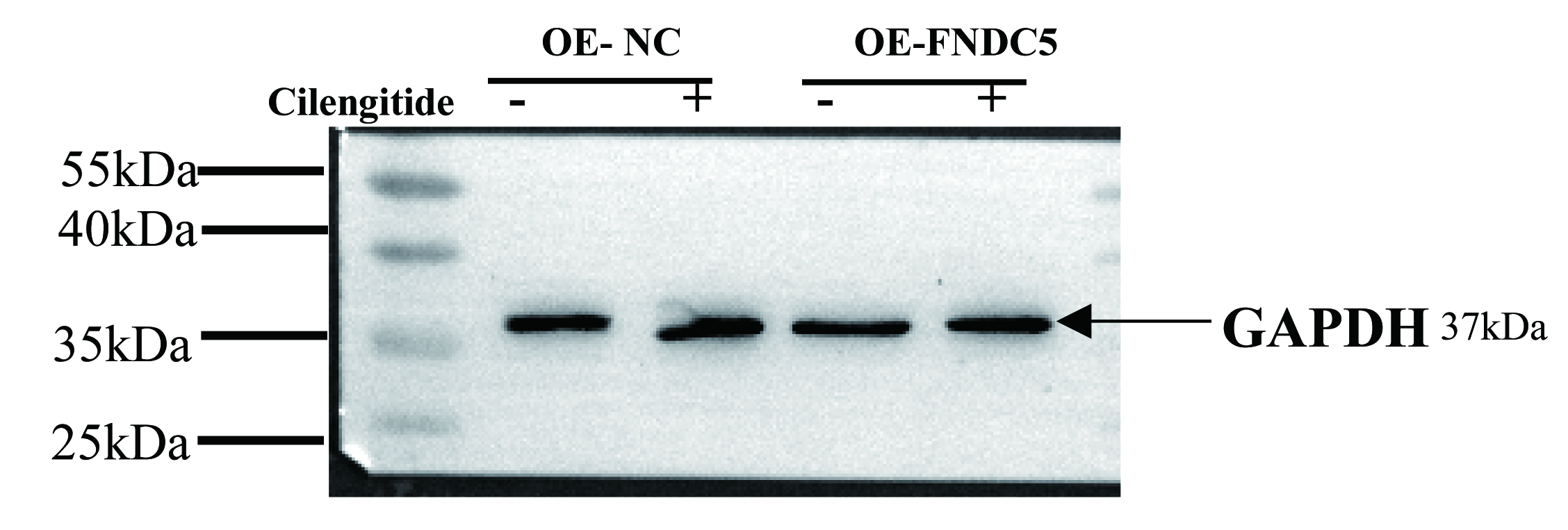

Supplement: Supplementary file 5 — Supplementary Material 5. [file 13395_2026_420_MOESM5_ESM.zip › Supplementary Material 5/Fig4/Fig4H/GAPDH/GAPDH-2.tif]

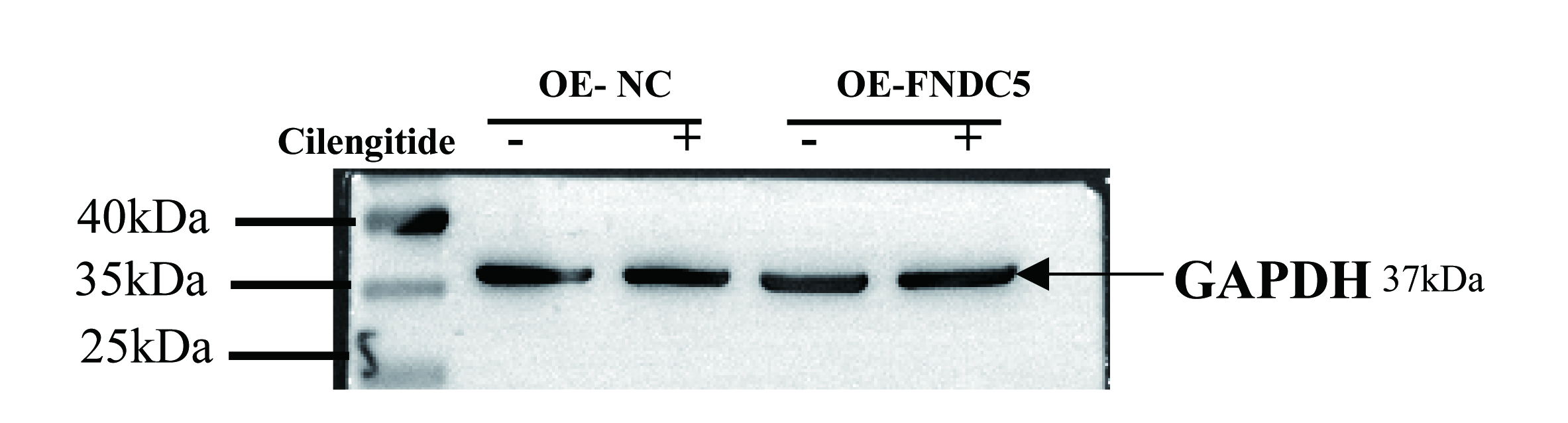

Supplement: Supplementary file 5 — Supplementary Material 5. [file 13395_2026_420_MOESM5_ESM.zip › Supplementary Material 5/Fig4/Fig4H/GAPDH/GAPDH-3.tif]

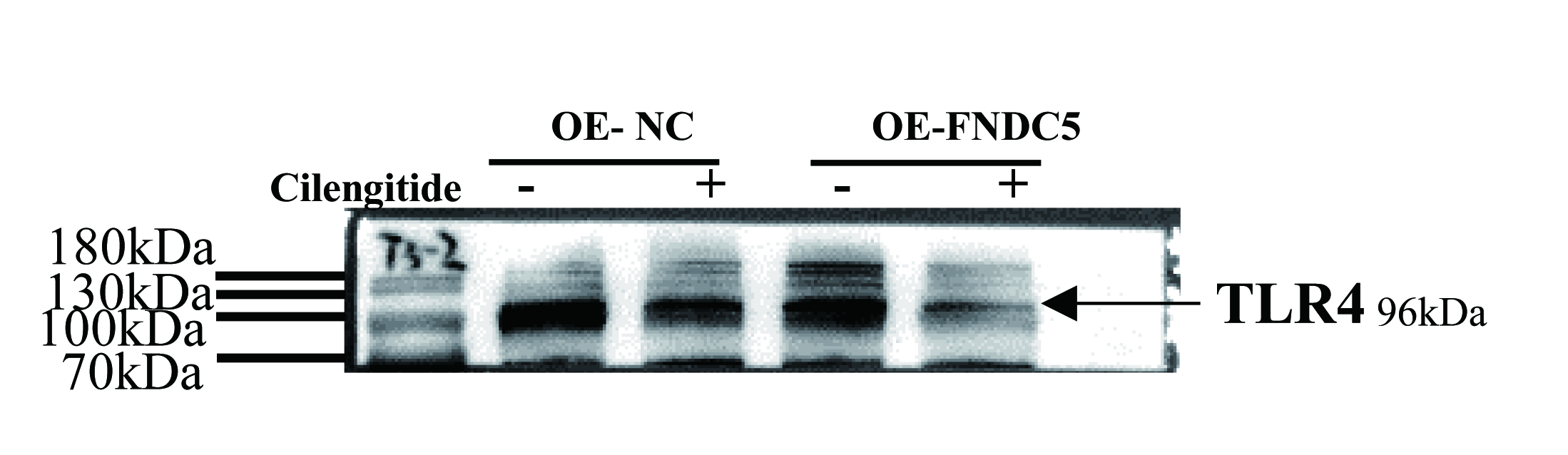

Supplement: Supplementary file 5 — Supplementary Material 5. [file 13395_2026_420_MOESM5_ESM.zip › Supplementary Material 5/Fig4/Fig4H/TLR4/TLR4-1.tif]

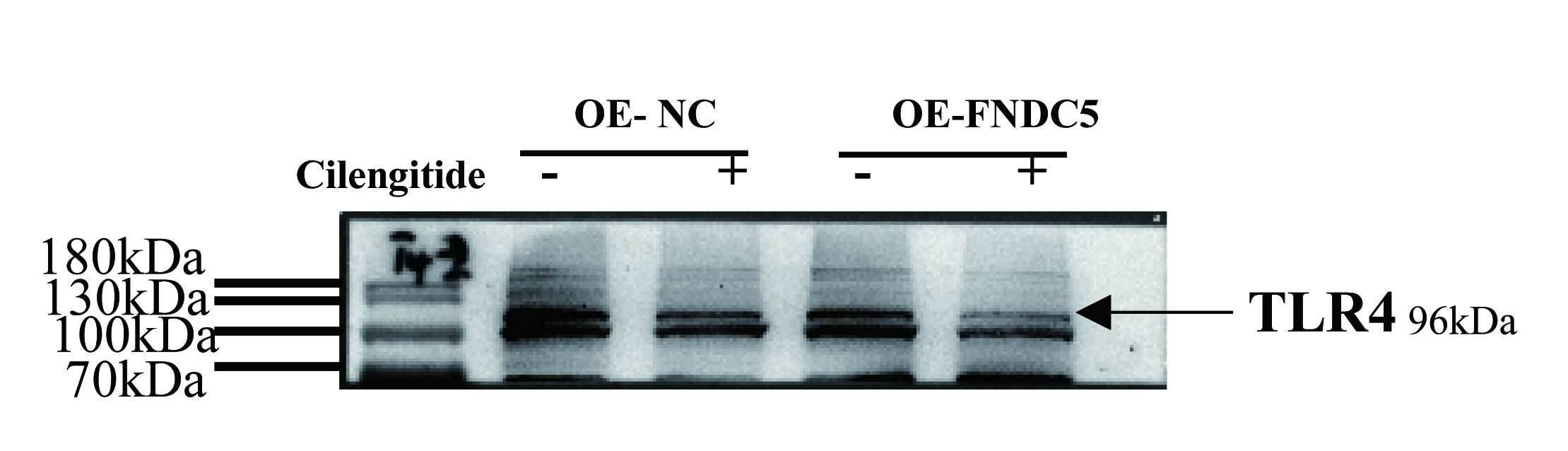

Supplement: Supplementary file 5 — Supplementary Material 5. [file 13395_2026_420_MOESM5_ESM.zip › Supplementary Material 5/Fig4/Fig4H/TLR4/TLR4-2.tif]

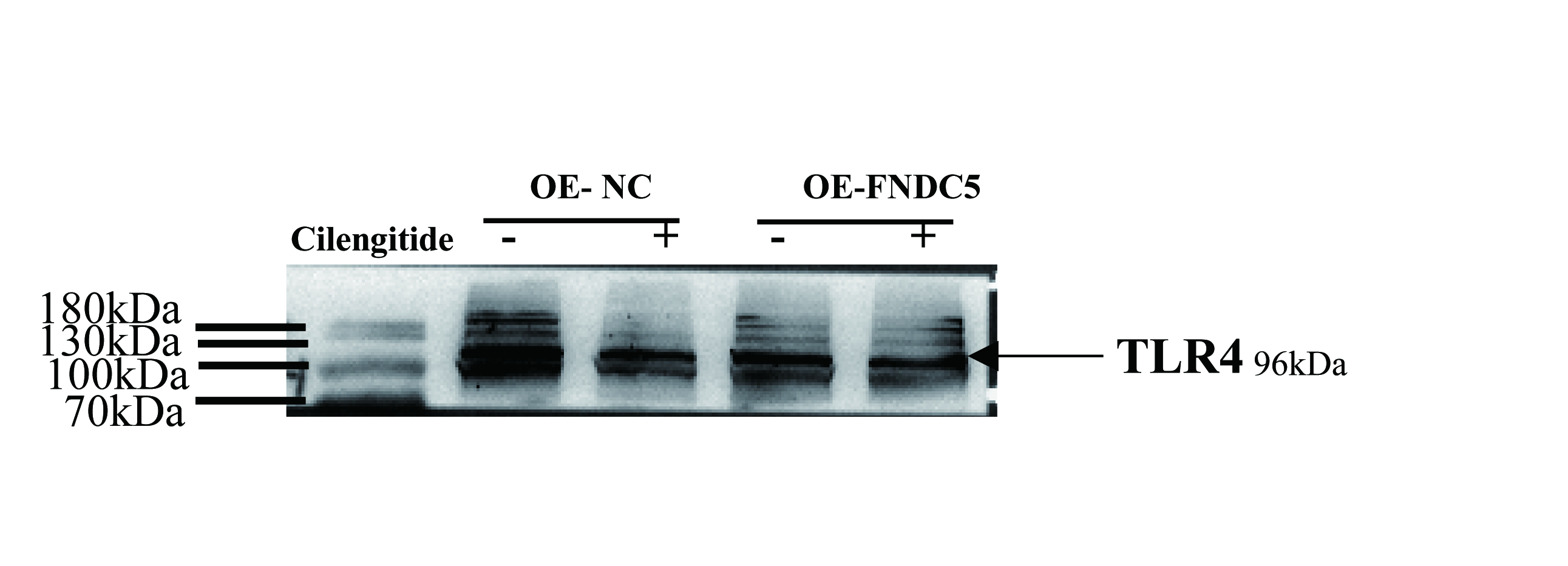

Supplement: Supplementary file 5 — Supplementary Material 5. [file 13395_2026_420_MOESM5_ESM.zip › Supplementary Material 5/Fig4/Fig4H/TLR4/TLR4-3.tif]

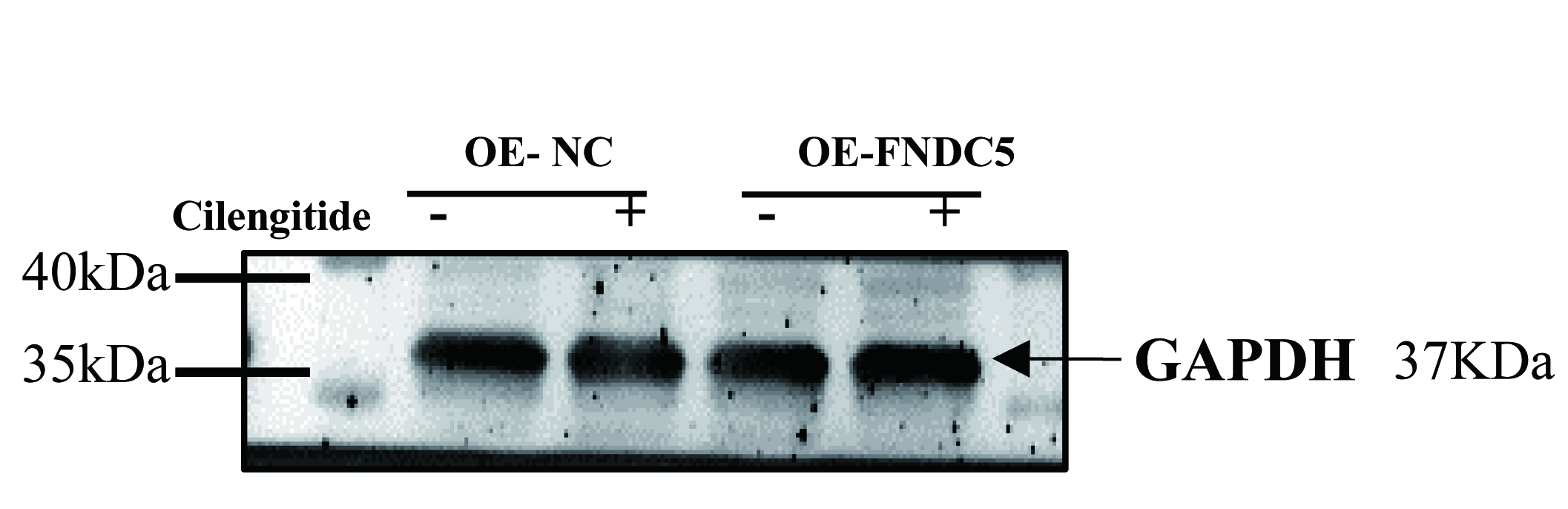

Supplement: Supplementary file 5 — Supplementary Material 5. [file 13395_2026_420_MOESM5_ESM.zip › Supplementary Material 5/supplimentary/GAPDH/GAPDH-1.tif]

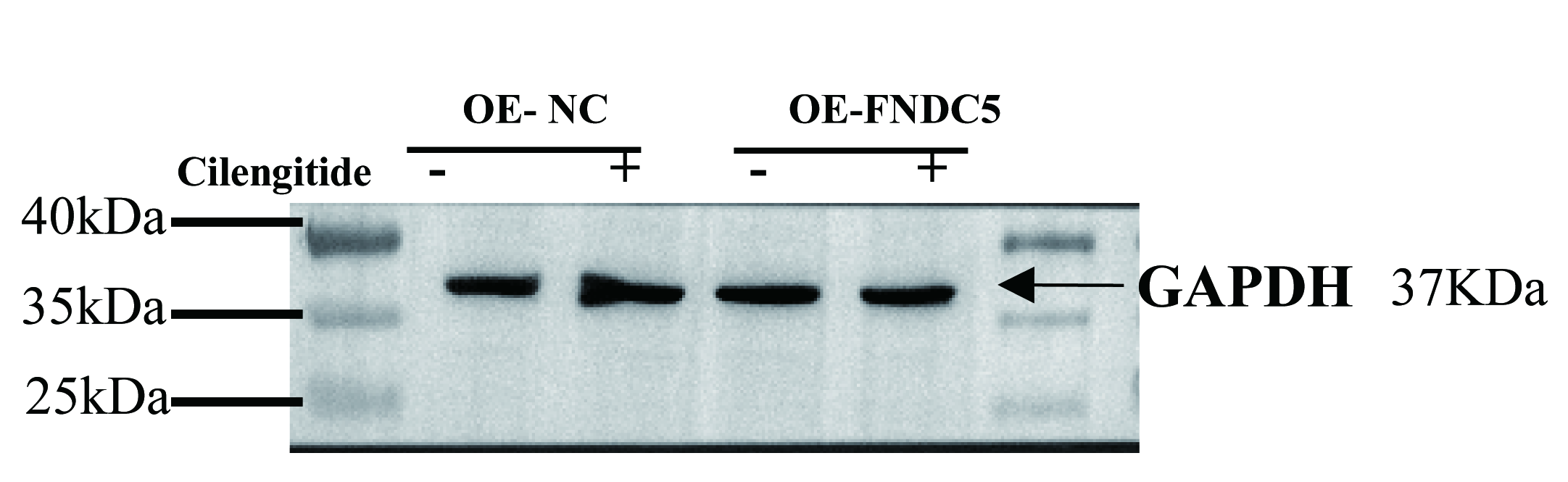

Supplement: Supplementary file 5 — Supplementary Material 5. [file 13395_2026_420_MOESM5_ESM.zip › Supplementary Material 5/supplimentary/GAPDH/GAPDH-2.tif]

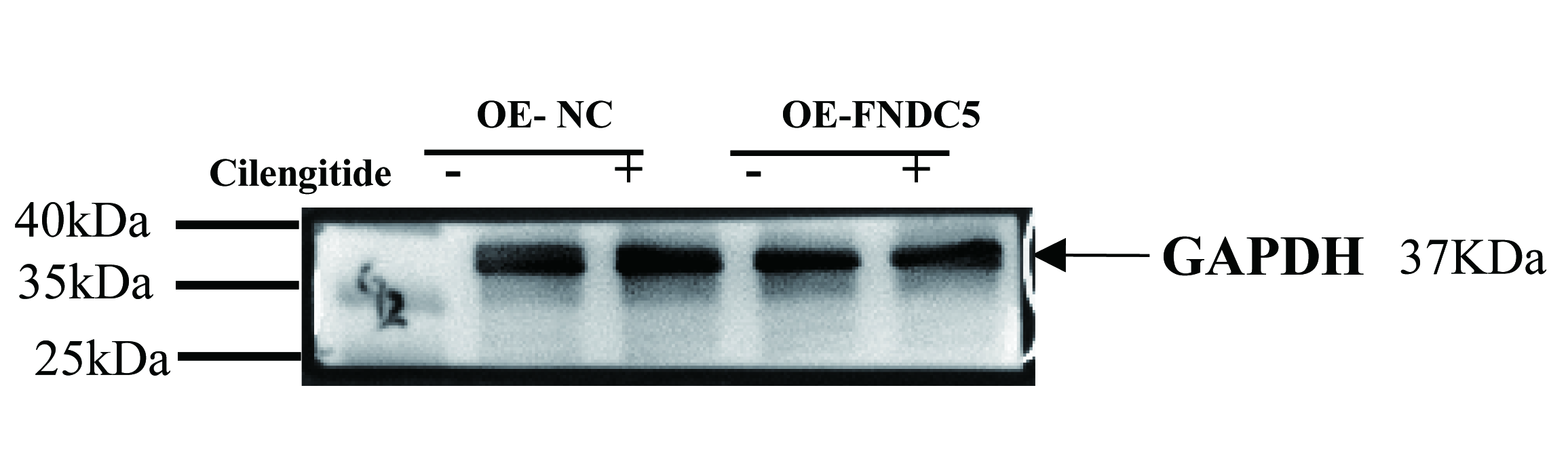

Supplement: Supplementary file 5 — Supplementary Material 5. [file 13395_2026_420_MOESM5_ESM.zip › Supplementary Material 5/supplimentary/GAPDH/GAPDH-3.tif]

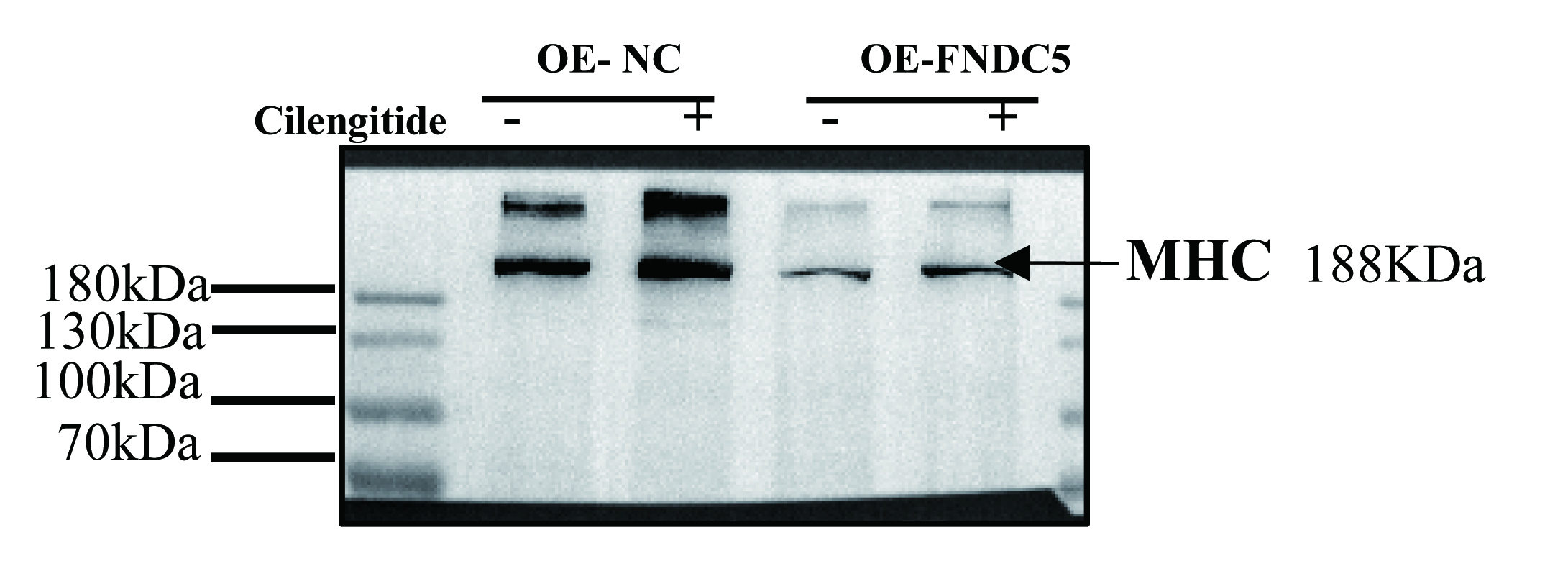

Supplement: Supplementary file 5 — Supplementary Material 5. [file 13395_2026_420_MOESM5_ESM.zip › Supplementary Material 5/supplimentary/MHC/MHC-1.tif]

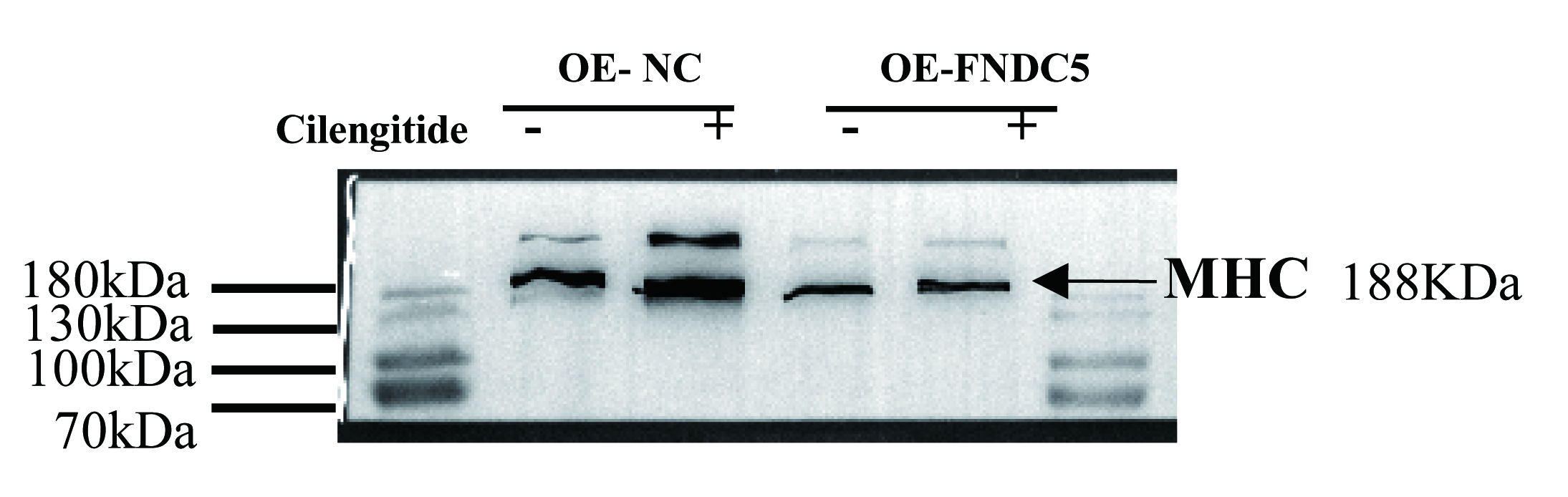

Supplement: Supplementary file 5 — Supplementary Material 5. [file 13395_2026_420_MOESM5_ESM.zip › Supplementary Material 5/supplimentary/MHC/MHC-2.tif]

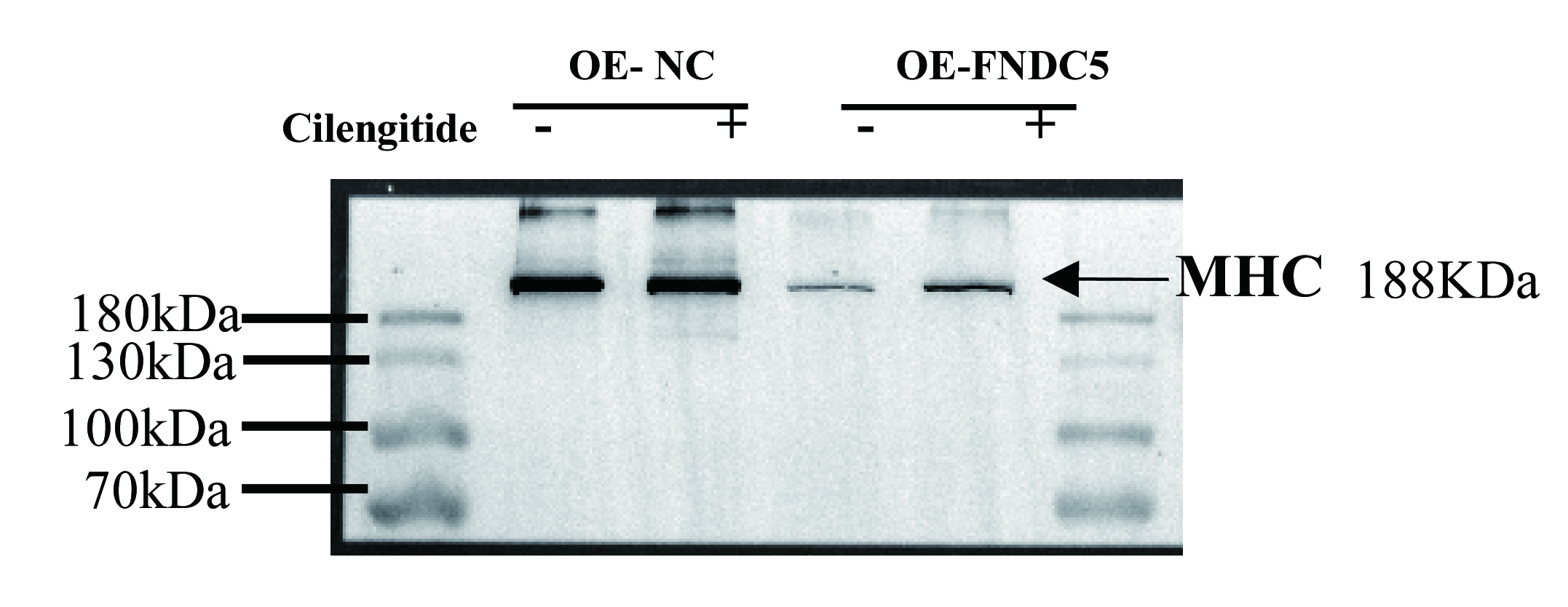

Supplement: Supplementary file 5 — Supplementary Material 5. [file 13395_2026_420_MOESM5_ESM.zip › Supplementary Material 5/supplimentary/MHC/MHC-3.tif]

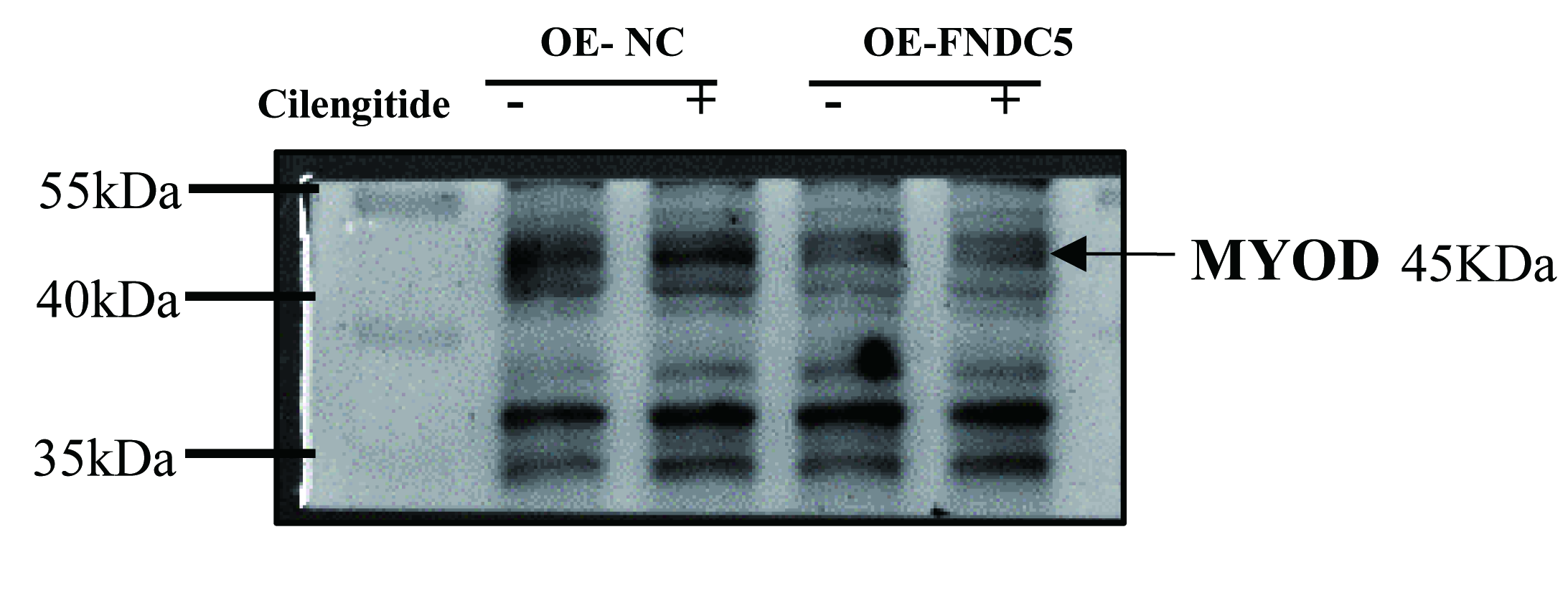

Supplement: Supplementary file 5 — Supplementary Material 5. [file 13395_2026_420_MOESM5_ESM.zip › Supplementary Material 5/supplimentary/MYOD/MYOD-1.tif]

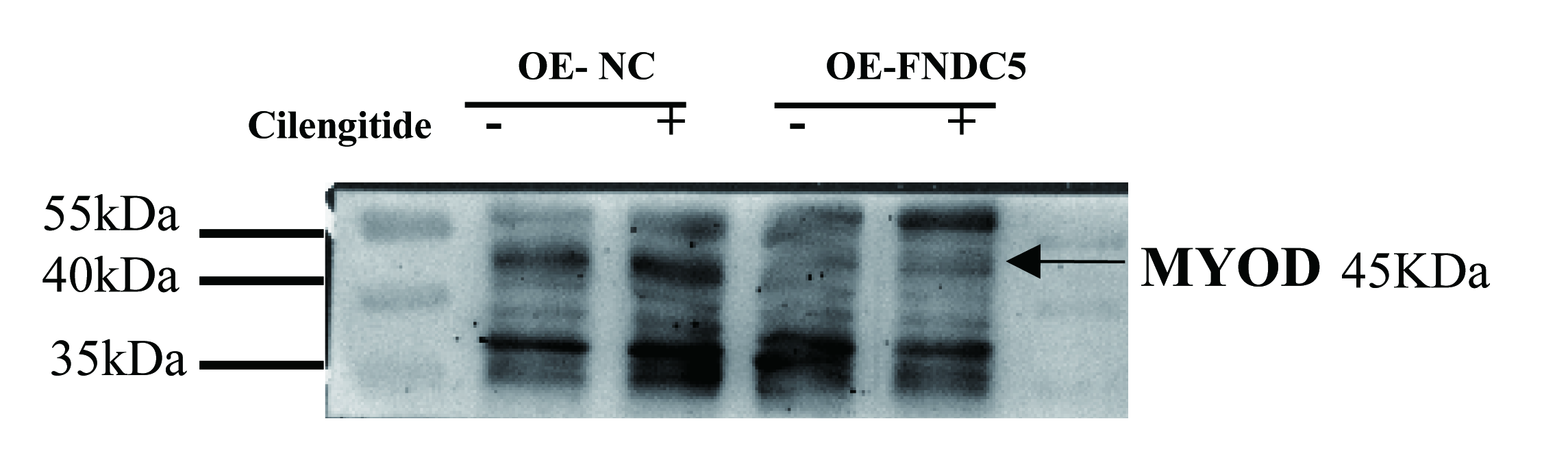

Supplement: Supplementary file 5 — Supplementary Material 5. [file 13395_2026_420_MOESM5_ESM.zip › Supplementary Material 5/supplimentary/MYOD/MYOD-2.tif]

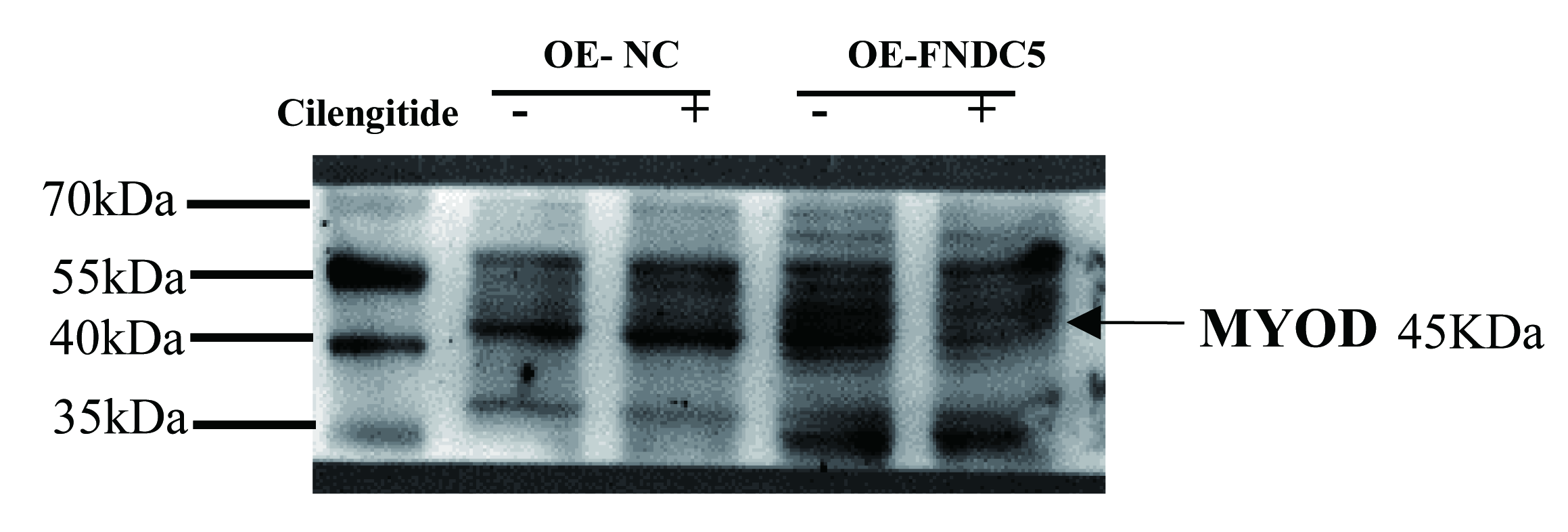

Supplement: Supplementary file 5 — Supplementary Material 5. [file 13395_2026_420_MOESM5_ESM.zip › Supplementary Material 5/supplimentary/MYOD/MYOD-3.tif]

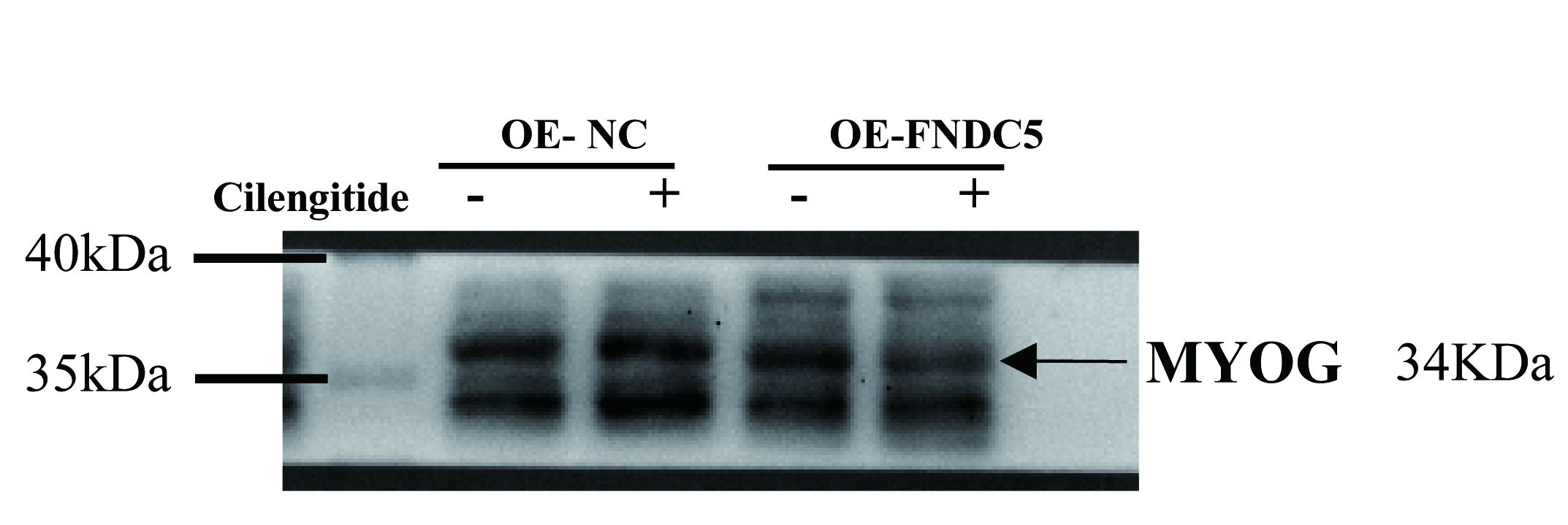

Supplement: Supplementary file 5 — Supplementary Material 5. [file 13395_2026_420_MOESM5_ESM.zip › Supplementary Material 5/supplimentary/MYOG/MYOG-1.tif]

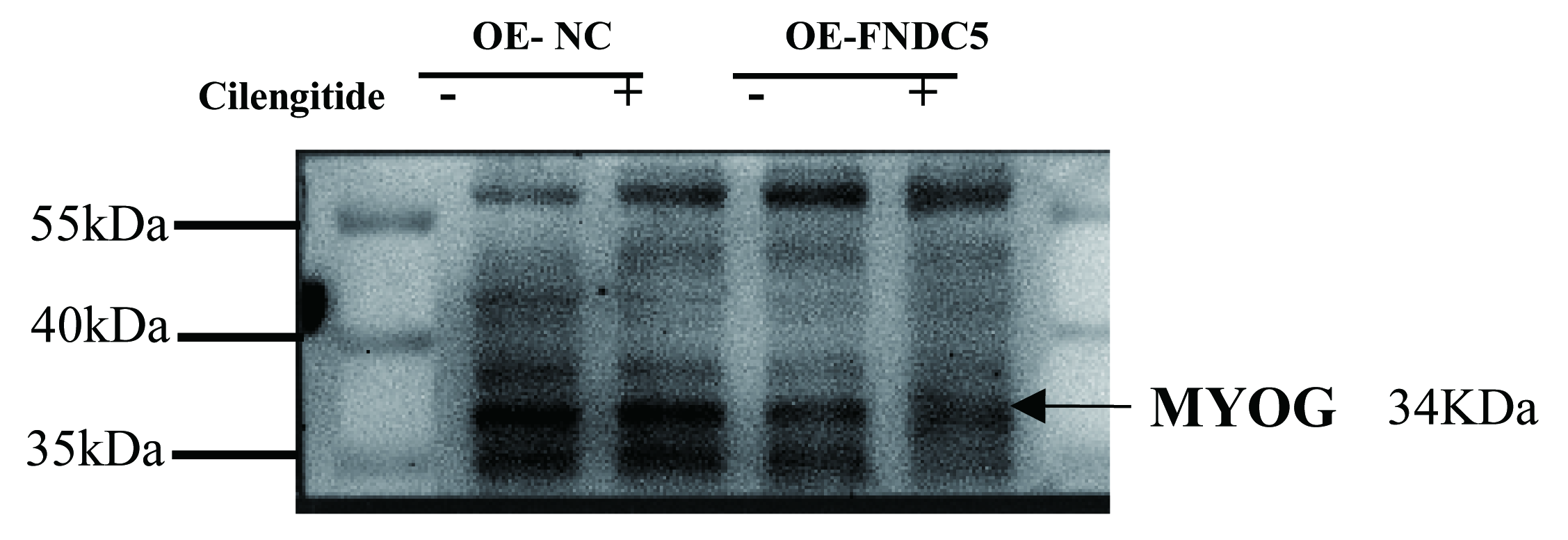

Supplement: Supplementary file 5 — Supplementary Material 5. [file 13395_2026_420_MOESM5_ESM.zip › Supplementary Material 5/supplimentary/MYOG/MYOG-2.tif]

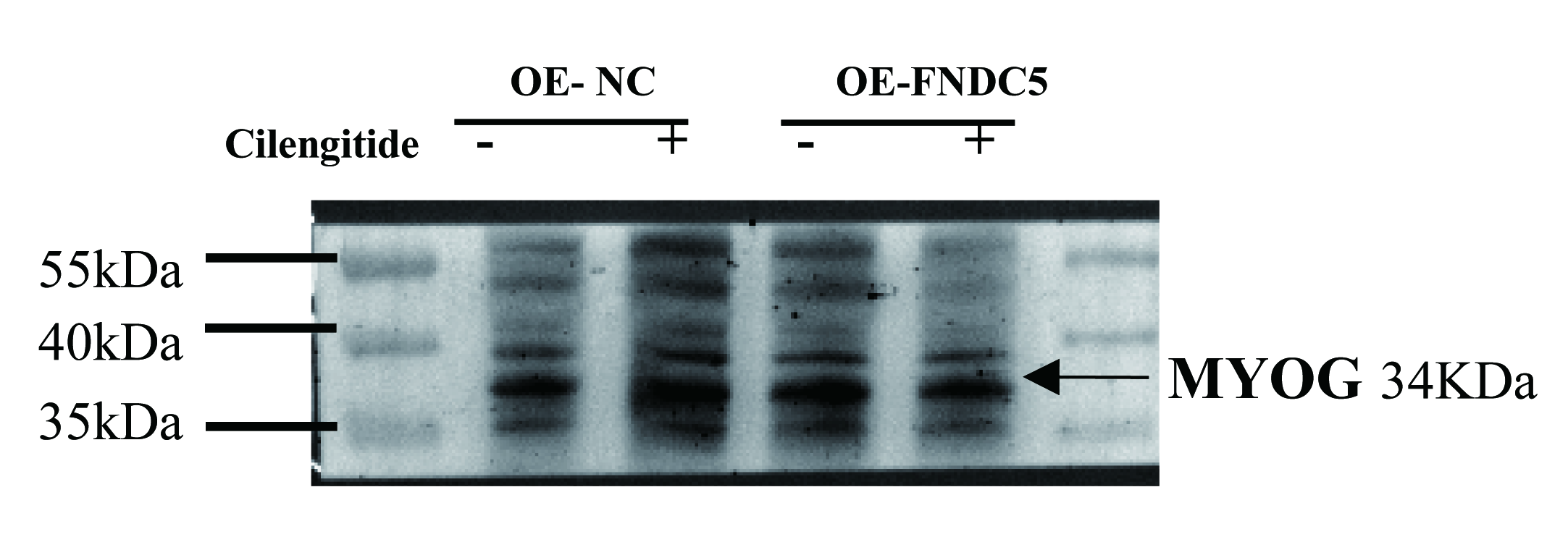

Supplement: Supplementary file 5 — Supplementary Material 5. [file 13395_2026_420_MOESM5_ESM.zip › Supplementary Material 5/supplimentary/MYOG/MYOG-3.tif]
