# Supplementary material for: Integrin αVβ5 regulates myoblast proliferation and differentiation in sarcopenia mice treated with FNDC5 gene delivery: Original article
Source: Skelet Muscle. 2026 Mar 17;16:28. doi: 10.1186/s13395-026-00420-x (PMC13347998; doi:10.1186/s13395-026-00420-x)
Supplement: Supplementary file 6 — Supplementary Material 6. [file 13395_2026_420_MOESM6_ESM.docx]

**Supplementary Fig. 1** Map of Sh-NC expression vector; Information of the vector ， *FNDC5* overexpression sbuttle vector map; Control vector map

Map of Sh-NC expression vector


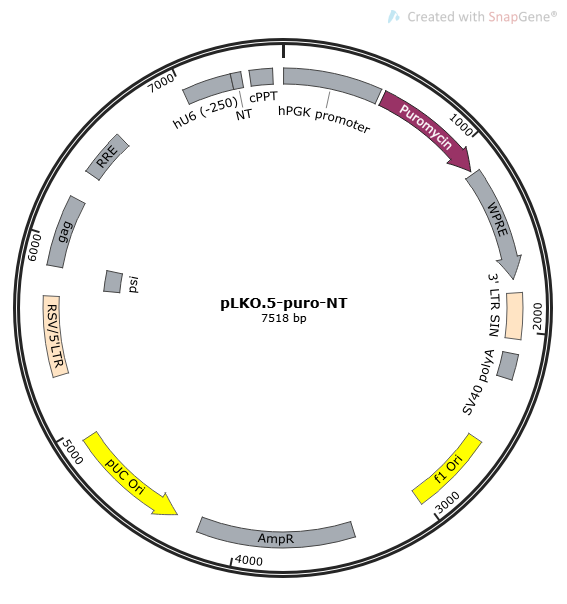


***FNDC5* overexpression sbuttle vector map
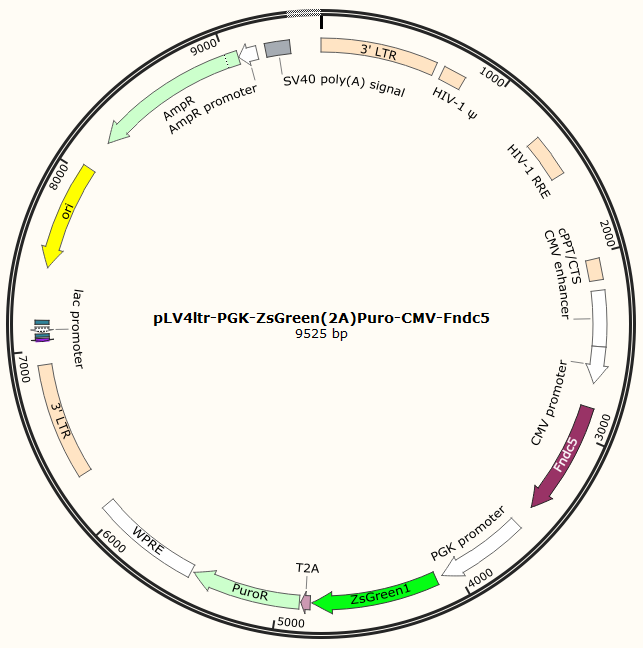
**

**Control vector map**

**
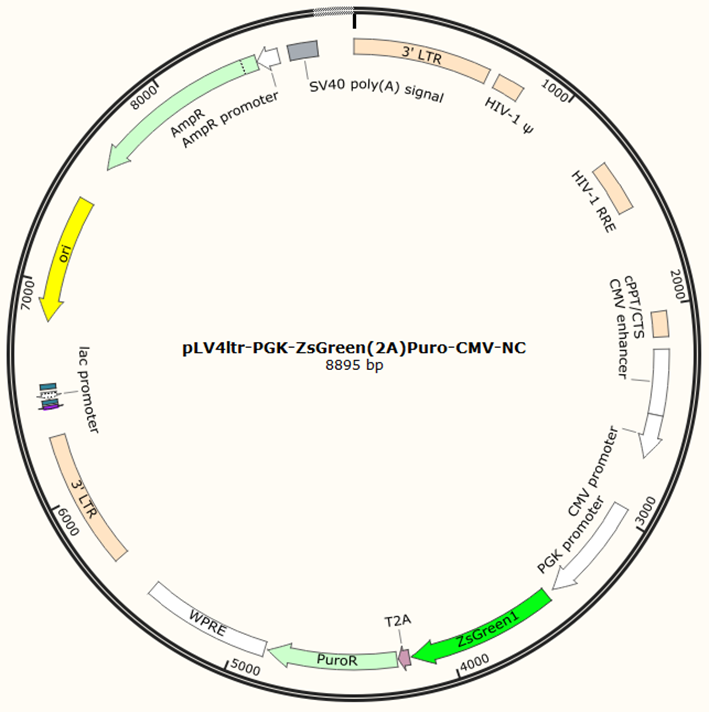
**

**Supplementary Table 1** sequences of shRNA against *FNDC5* and *FNDC5* Gene synthesis sequences;

|  | **Vector Name** | **Sequences** |
| --- | --- | --- |
| **shRNA against FNDC5** | pLKO.5* shControl | CAACAAGATGAAGAGCACCAA |
|  | pLKO.5 shFNDC5-1 | GAGGAGGATACGGAGTACATA |
|  | pLKO.5 shFNDC5-2 | GATGGCCTCCAAGAACAAAGA |
| **fibronectin type III domain containing 5 (FNDC5) (** **NM_027402.4)** |  | TTCGAAGCCACCATGCCCCCAGGGCCGTGCGCCTGGCCGCCCCGCGCCGCGCTCCGCCTGTGGCTAGGCTGCGTCTGCTTCGCGCTGGTGCAGGCGGACAGCCCCTCAGCCCCTGTGAACGTGACCGTCCGGCACCTCAAGGCCAACTCTGCCGTGGTCAGCTGGGATGTCCTGGAGGATGAAGTGGTCATTGGCTTTGCCATCTCTCAGCAGAAGAAGGATGTGCGGATGCTCCGGTTCATTCAGGAGGTGAACACCACCACCCGGTCCTGCGCTCTCTGGGACCTGGAGGAGGACACAGAATATATCGTCCATGTGCAGGCCATCTCCATCCAGGGACAGAGCCCAGCCAGTGAGCCTGTGCTCTTCAAGACCCCACGCGAGGCTGAAAAGATGGCCTCAAAGAACAAAGATGAGGTGACCATGAAGGAGATGGGGAGGAACCAGCAGCTGCGAACGGGGGAGGTGCTGATCATTGTTGTGGTCCTCTTCATGTGGGCAGGTGTTATAGCTCTCTTCTGCCGCCAGTATGATATCATCAAGGACAACGAGCCCAATAACAACAAGGAGAAAACCAAGAGCGCATCAGAAACCAGCACACCGGAGCATCAGGGTGGGGGTCTCCTCCGCAGCAAGATATGACCCGGG |

**FNDC5 Bioss FNDC5，bs-8486R（1:200）**

**Supplementary Table 2** The antibody used for western blot analysis

| **Gene Name** | **Manufacturer** | **Lot. No** | **Source** | **Application** | **Dilution** |
| --- | --- | --- | --- | --- | --- |
| FNDC5 | Abcam | ab174833 | Rabbit | WB | 1:1000 |
|  | Proteintech | 23995-1-AP | Rabbit | IHC | 1:200 |
|  | Affinity | DF13019 | Rabbit | IF | 1:100 |
| MyoD | Santacruz | SC-32758 | Mouse | WB | 1:1000 |
| Myogenin | Santacruz | SC-12732 | Mouse | WB | 1:1000 |
| MHC(MF-20) | DSHB | AB_2147781 | Mouse | WB | 0.5ug/ml |
| anti-mTOR | service bio | GB111839 | Rabbit | WB | 1:1000 |
| Phospho-mTOR (Ser2481) | Cell Signaling Technology | 2974 | Mouse | WB | 1:1000 |
| Phospho-mTOR (Ser2481) | Affinity | 3309 | Rabbit | IHC | 1:200 |
| AKT | Santacruz | SC-8312 | Rabbit | WB | 1:1000 |
| p-Akt(S473) | Affinity | 4060 | Rabbit | WB | 1:1000 |
| p70 S6 kinase | service bio | GB111133-100 | Rabbit | WB | 1:1000 |
| phospho-p70 S6 kinase | Cell Signaling Technology | 34475 | Rabbit | WB | 1:1000 |
|  |  |  |  | IHC | 1:200 |
| FAK | Cell Signaling Technology | 3285S | Rabbit | WB | 1:1000 |
| phospho-FAK(Tyr397) | Cell Signaling Technology | 8556 | Rabbit | WB | 1:1000 |
| phospho-FAK(Tyr397) | Affinity | 3398 | Rabbit | IHC | 1:200 |
| SRC | Cell Signaling Technology | 2109S | Rabbit | WB | 1:1000 |
| p-SRC(Tyr416) | Cell Signaling Technology | 6943 | Rabbit | WB | 1:1000 |
| Integrin αV/β5 | Santacruz | sc-81632 | Mouse | IF | 1:200 |
| Integrin αV | Cell Signaling Technology | 4711 | Rabbit | WB | 1:1000 |
| Integrin β5 | Cell Signaling Technology | 4708 | Rabbit | WB | 1:1000 |
| TLR4 | Affinity | AF7017 | Rabbit | WB | 1:1000 |
| Biglycan | Proteintech | 16409-1-AP | Rabbit | WB | 1:1000 |
| COL5A3 | Affinity | AF9046 | Rabbit | WB | 1:1000 |
| phospho-CDK2(Thr160) | Affinity | AF3237 | Rabbit | WB | 1:1000 |
| CDK2 | Affinity | AF6237 | Rabbit | WB | 1:1000 |
| CDC25A | Affinity | AF6252 | Rabbit | WB | 1:1000 |
| phospho-CDC25A(Ser124) | Affinity | AF3254 | Rabbit | WB | 1:1000 |
| DyLight 488, Goat Anti-Mouse IgG | Abbkine | A23210 | Mouse | WB | 1:500 |
| Dylight 594, Goat Anti-Mouse IgG | Abbkine | A0473 | Mouse | WB | 1:500 |
| HRP conjugated Goat Anti-Rabbit IgG （H+L） | service bio | GB23303 | Goat | WB | 1:1000 |
| HRP conjugated Goat Anti-Mouse IgG （H+L） | service bio | GB23304 | Goat | WB | 1:1000 |
| GAPDH | service bio | GB15002-100 | Mouse | WB | 1:1000 |

**Supplementary Table 3** The primers sequences used for qPCR

| **Gene name** | **Forward (5’-3’)** | **Reverse (5’-3’)** |
| --- | --- | --- |
| *Tnc* | ACGGCTACCACAGAAGCTG | ATGGCTGTTGTTGCTATGGCA |
| *Col5a3* | CGGGGTACTCCTGGTCCTAC | GCATCCCTACTTCCCCCTTG |
| *Bgn* | TGCCATGTGTCCTTTCGGTT | CAGGTCTAGCAGTGTGGTGTC |
| *Matn2* | CTATGTATGCCGTTGGGGTAGG | AGCTTTTCACTTATTTCGCCCAT |
